# Informing Rift Valley Fever preparedness by mapping seasonally varying environmental suitability

**DOI:** 10.1016/j.ijid.2020.07.043

**Published:** 2020-10

**Authors:** Austin N. Hardcastle, Joshua C.P. Osborne, Rebecca E. Ramshaw, Erin N. Hulland, Julia D. Morgan, Molly K. Miller-Petrie, Julia Hon, Lucas Earl, Peter Rabinowitz, Judith N. Wasserheit, Marius Gilbert, Timothy P. Robinson, G.R. William Wint, Shreya Shirude, Simon I. Hay, David M. Pigott

**Affiliations:** a Institute for Health Metrics and Evaluation, University of Washington, Seattle, WA, USA; b Department of Global Health, University of Washington, Seattle, WA, USA; c Spatial Epidemiology Lab (SpELL), Université Libre de Bruxelles, Brussels, Belgium; d Fonds National de la Recherche Scientifique (FNRS), Brussels, Belgium; e Animal Production and Health Division (AGA), Food and Agriculture Organization of the United Nations, Italy; f Environmental Research Group Oxford (ERGO), c/o Department of Zoology, Oxford, UK; g Department of Health Metrics Sciences, School of Medicine, University of Washington, Seattle, WA, USA

**Keywords:** Rift Valley Fever, Preparedness, Environmental suitability, Spillover potential, Outbreak, Mapping

## Abstract

**Background:**

Rift Valley Fever (RVF) poses a threat to human and animal health throughout much of Africa and the Middle East and has been recognized as a global health security priority and a key preparedness target.

**Methods:**

We combined RVF occurrence data from a systematic literature review with animal notification data from an online database. Using boosted regression trees, we made monthly environmental suitability predictions from January 1995 to December 2016 at a 5 × 5-km resolution throughout regions of Africa, Europe, and the Middle East. We calculated the average number of months per year suitable for transmission, the mean suitability for each calendar month, and the “spillover potential,” a measure incorporating suitability with human and livestock populations.

**Results:**

Several countries where cases have not yet been reported are suitable for RVF. Areas across the region of interest are suitable for transmission at different times of the year, and some areas are suitable for multiple seasons each year. Spillover potential results show areas within countries where high populations of humans and livestock are at risk for much of the year.

**Conclusions:**

The widespread environmental suitability of RVF highlights the need for increased preparedness, even in countries that have not previously experienced cases. These maps can aid in prioritizing long-term RVF preparedness activities and determining optimal times for recurring preparedness activities. Given an outbreak, our results can highlight areas often at risk for subsequent transmission that month, enabling decision-makers to target responses effectively.

## Highlights

• Database of Rift Valley Fever occurrences from 46 countries over 22 years.
• Predictions of Rift Valley Fever suitability for every month over 1995–2016.
• Identifies areas at-risk by synthesizing time-series of environmental predictions.
• We use human and livestock data to identify possible hotspots of disease spillover.
• We identify places where long-term and routine preparation efforts should be focused.

## Introduction

Rift Valley Fever (RVF) is an environmentally-driven mosquito-borne disease that affects human and animal health throughout Africa and the Middle East, with the potential to affect additional regions. Since the first case was reported in livestock in Kenya in 1910, the virus has caused large outbreaks throughout sub-Saharan Africa and, more recently, become endemic in parts of the Arabian Peninsula (Linthicum et al., 2016). Outbreaks in livestock herds have caused multimillion-dollar shocks to many pastoral economies (Nanyingi et al., 2015, Peyre et al., 2015).

Human cases of RVF often occur in proximity to livestock outbreaks, and the most affected human populations typically rely financially on livestock ownership (Peyre et al., 2015). Humans can be infected through the bite of an infected mosquito or contact with the bodily fluids of infected mammals (Daubney and Hudson, 1933). Most people infected with the virus are asymptomatic or have a mild, self-limited illness; up to ten percent of those infected develop a more severe disease that can result in permanent vision loss, meningoencephalitis, or hemorrhagic fever. While past outbreaks have reported overall case-fatality ratios of under one percent, the fatality ratio among people who develop hemorrhagic fever is approximately 50 percent (Mandell and Bennett, 1994, WHO, 2019b). The disease is also associated with abortion rates of up to 100 percent in sheep and cows (Bouloy, 2005) and can cause miscarriages and neonatal disease through trans-placental transmission in humans (Arishi et al., 2006, Adam and Karsany, 2008, Baudin et al., 2016).

RVF outbreaks typically occur after prolonged rainfall (Davies et al., 1985, Caminade et al., 2014, Williams et al., 2016), which may drive transmission by creating mosquito habitats or hatching previously infected mosquito eggs (Linthicum et al., 1983, Linthicum et al., 1985). Mosquitoes and other RVF vectors are present on every continent except Antarctica (Linthicum et al., 2016). International trade in livestock (Carroll et al., 2011, Gür et al., 2017, Lancelot et al., 2017) can bring RVF to new regions, with the potential to trigger a new transmission cycle or sustained endemicity under suitable environmental conditions. Given the vector’s reliance on these transmission conditions, one way to estimate RVF risk is to estimate environmental suitability (Walz et al., 2015, Eneanya et al., 2018, Messina et al., 2019).

The impacts of RVF on human health and economic production, and lack of effective clinical countermeasures, motivated the World Health Organization (WHO) to classify it as a Research and Development Blueprint Priority Pathogen (WHO, 2019a). Existing livestock vaccines for RVF have not proven feasible for large-scale rollout because of multi-dose regimens or limited effectiveness (Faburay et al., 2017). No antiviral drugs are licensed for the prevention or treatment of RVF in humans; clinical management of severe cases is limited to non-specific supportive care. To disrupt and prevent transmission, the Coalition for Epidemic Preparedness Innovations recently launched a search for a human RVF vaccine (CEPI, 2019).

Many RVF preparedness activities focus on early response, with increasing intensity as warning signs amplify, as outlined in the Rift Valley Fever Decision Support Framework following the 2006–2007 East African outbreak (Consultative Group for RVF Decision Support, 2010). Existing RVF response and control techniques can be improved by putting in place the necessary infrastructure, such as stocked labs and trained staff before a case is reported.

Existing forecasting tools, such as NASA’s Rift Valley Fever Monitor (Anyamba et al., 2009), identify climatic patterns associated with RVF, such as those caused by El Niño Southern Oscillation events (Anyamba et al., 2012), providing warnings several months in advance. Preparation efforts such as the construction of health facilities, education of livestock owners, and distribution of vaccines if they became available, may require more lead-time than existing tools afford. Risk maps with a broad geographic scope can prioritize where to focus interventions both across and within countries.

Existing mapping efforts show regions at risk for RVF based on environmental conditions. Some maps stratify this risk in space by comparing climatic signals, representative of year-round climate conditions, from specific locations to the same signals in places that have experienced RVF outbreaks (Anyamba et al., 2002, Redding et al., 2017, Walsh et al., 2017). This approach, using synoptic, year-round data, does not fully capture the seasonal nature of climatic factors that drive RVF transmission. In contrast, forecasting efforts provide such precise seasonal information that they may not give enough lead-time for long-term preparation. Our work provides a synoptic framework for long-term RVF preparedness driven by season-specific data.

Given the seasonality of environmental suitability for RVF, we stratify risk at a monthly level, estimating environmental suitability from corresponding monthly climate data. Monthly predictions over a large area build on previous regional work (Anyamba et al., 2002, Soti et al., 2012, Sindato et al., 2014, Cavalerie et al., 2015) and explore patterns on a large scale that some farmers have already perceived locally (Owange et al., 2014, Alhaji et al., 2018). Holding other factors constant, areas suitable for more months out of the year are more likely to experience outbreaks. We combine suitability analyses with human and livestock population data to estimate the “spillover potential”. Combined with local considerations, these resources can provide guidance on where and when to focus RVF preparedness efforts.

## Methods

### Data intake

To obtain dates and locations of past RVF cases, we used two datasets. We compiled the first dataset by conducting a systematic literature review, using a similar protocol to those described elsewhere (Pigott et al., 2014, Messina et al., 2015), and outlined in the appendix. We included 892 papers for full-text review and found RVF reports with geographic information in 250, yielding 2,813 occurrence records. The second dataset came from the Global Animal Disease Information System (EMPRES-i) database from the United Nations Food and Agriculture Organization (FAO) (FAO, 2019). This database contains reports of animal diseases, including RVF, confirmed at regional partner laboratories. We downloaded 961 confirmed RVF occurrences from the EMPRES-i database on Oct 11, 2018, and removed duplicate entries from our literature database.

When available, we recorded exact GPS coordinates associated with occurrences as “point” data. When point information was not available, we extracted locations as two-dimensional bounded regions, or “polygons”. These polygons were often administrative units, such as districts or states, to which the paper referred, though some were custom sampling regions.

### Pre-modeling data processing

To represent the timing of disease transmission, we only included RVF occurrences in our model that were confirmed with PCR or detected in samples from symptomatic individuals diagnosed with serological tests, as these conditions best represent active infection. Occurrences in the EMPRES-i dataset are confirmed once they are tested at regional FAO partner laboratories; these results were included in the model, though the type of test used was not specified. Our modeling dataset contained 1,381 occurrence records. To obtain associated environmental information from an occurrence, we needed point data associated with a specific month and year. If occurrence data did not adhere to this format—for instance, if the location of a case was reported as a polygon, or the timing was reported as a range of dates—we stochastically sampled one point from each polygon or one month-year combination from each date range, as described in the appendix.

### Environmental suitability modeling

We used our occurrence data in a boosted regression tree model to predict RVF suitability in areas based on their environmental similarity to places and times that have experienced outbreaks (Mylne et al., 2015, Pigott et al., 2016). We also used absence data in the model to represent an environmental contrast between places with and without cases. Given the difficulty of determining disease absence, we simulated the same number of absence, or “background,” points as occurrence points in each bootstrap (Zaniewski et al., 2002). As described in the appendix, we defined a region around occurrence data, which encompassed much of Africa, Europe, and the Middle East. We sampled the coordinates of background points from this region, which is also the region for which we show predictions, and sampled the month-year combinations from a distribution based on the time trends of all occurrence data. Sampling background data from a region near previous RVF occurrence locations, provides more environmental contrast than if we sampled globally from regions with vastly different climates.

Our data on climatic signals, or covariates, driving RVF suitability came from globally complete datasets containing values for each 5 × 5-km pixel (further details on these datasets are presented in Appendix Table 4). We selected model covariates based on past modeling work and current understanding of RVF epidemiology (Table 1), with further justification detailed in the appendix.

**Table 1:** Covariates used for modelling.

1. Monthly rainfall
2. Monthly rainfall (one month prior)
3. Monthly rainfall (two months prior)
4. Distance to the closest floodplain
5. Saturated water content of soil
6. Bulk density of soil
7. Monthly mean temperature
8. Average enhanced vegetation index over all months and years
9. Standard deviation of monthly precipitation over the years 1995-2018

The parameters used to tune the boosted regression trees model were optimized to minimize the absolute fit error using a non-parametric Bayesian method over a finite space (Shahriari et al., 2016), as described further in the appendix (Appendix Table 3). We calculated the area under the curve (AUC) for each bootstrap, based on the bootstrap’s predictions for the occurrence and background points with which it was provided. This measure, “AUC_boot_,” provides validation for the internal machinery of the model.

### Data aggregation

We averaged 100 model bootstrap predictions for every month between January 1995 and December 2016 to create 264 month-year maps. For each of these, the difference between percentiles 2.5 and 97.5 of each pixel across all bootstraps was used to measure uncertainty (Appendix Figures 43–66). We calculated a suitability threshold for each monthly map, which optimized the combination of sensitivity and specificity, to turn continuous predictions in the monthly maps into binary maps, where pixels could be either suitable or not suitable. We used these binary maps to determine if we correctly predicted cases specific to their month and year and calculated the average number of suitable months per year for each pixel from 1995 to 2016.

For each calendar month, the maps specific to that month across all years were averaged to provide mean monthly suitability predictions. For each mean monthly map, we calculated AUC using the predictions associated with occurrences and backgrounds pooled from that month across all years. This statistic (“AUC_syn_”) measured the predictive validity of the mean monthly maps we provide.

### Spillover potential

We used binary suitability maps, livestock population estimates (Gilbert et al., 2018), and WorldPop, 2019 human population estimates (WorldPop, 2019) to calculate the number of humans and livestock (cattle, sheep, and goats) at risk in each second administrative level unit, hereafter referred to as “districts”. We calculated a composite measure for both humans and livestock, detailed in the appendix, based on absolute populations at risk and proportions of populations at risk in each district (Pigott et al., 2017). We then combined these measures to calculate spillover potential for each district in each month and year. We ranked all values across districts, months, and years and binned these values into quintiles. For each calendar month, for each district, we report the average quintile bin across all years.

This study follows the Guidelines for Accurate and Transparent Health Estimates Reporting (GATHER, Stevens et al., 2016) (Appendix Table 1).

### Role of the funding source

This work was supported by the Bill & Melinda Gates Foundation, Seattle, WA [grant number OPP1181128]. The sponsor of the study had no role in study design, data collection, data analysis, data interpretation, or writing of the report. The corresponding author had full access to all the data in the study and had final responsibility for the decision to submit for publication.

## Results

### Data extraction

**Figure 1:**
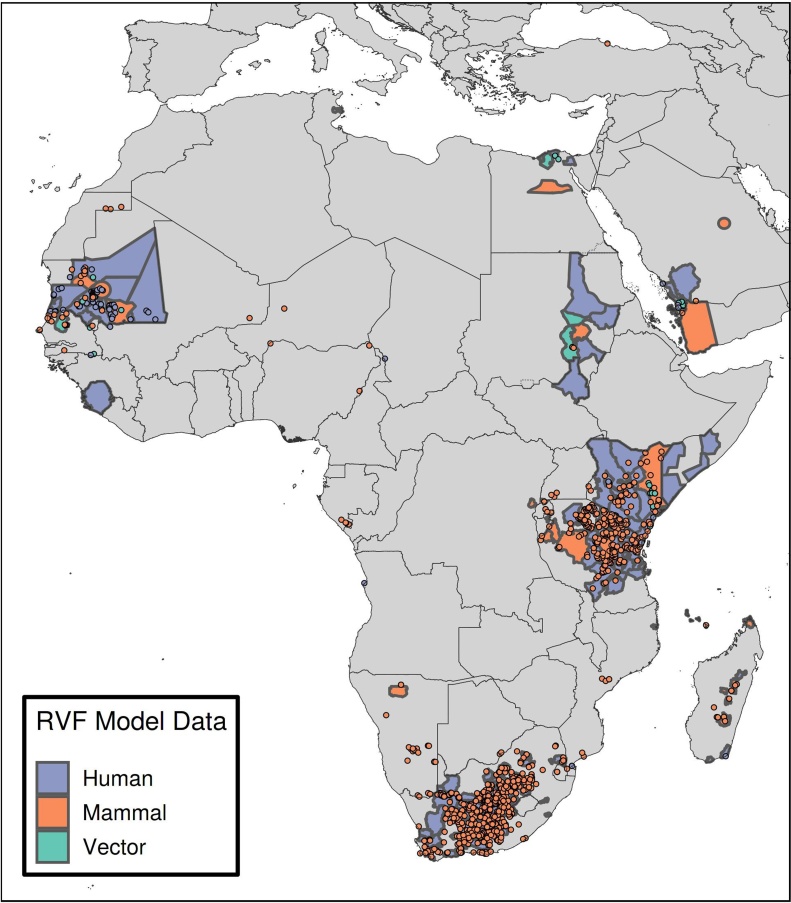
Rift Valley Fever detections used for modelling. Caption: Data from the EMPRES-i database and our literature extraction are shown. These occurrence data represent cases in humans (blue), mammals (red), and vectors (green) that were detected either by PCR or by a serological test on a symptomatic subject. They were used as input into the environmental suitability model.

We included 1,381 reports of symptomatic or PCR-confirmed RVF cases in humans, as well as cases in mammals and vectors, from 32 countries in our model (Figure 1, Appendix Table 2), the majority from countries that had experienced large outbreaks. Some countries, including Zambia (Samui et al., 1997) and Togo (Zeller et al., 1995), have serological evidence of infection but no active or symptomatic case reports.

### Model performance

**Figure 2:**
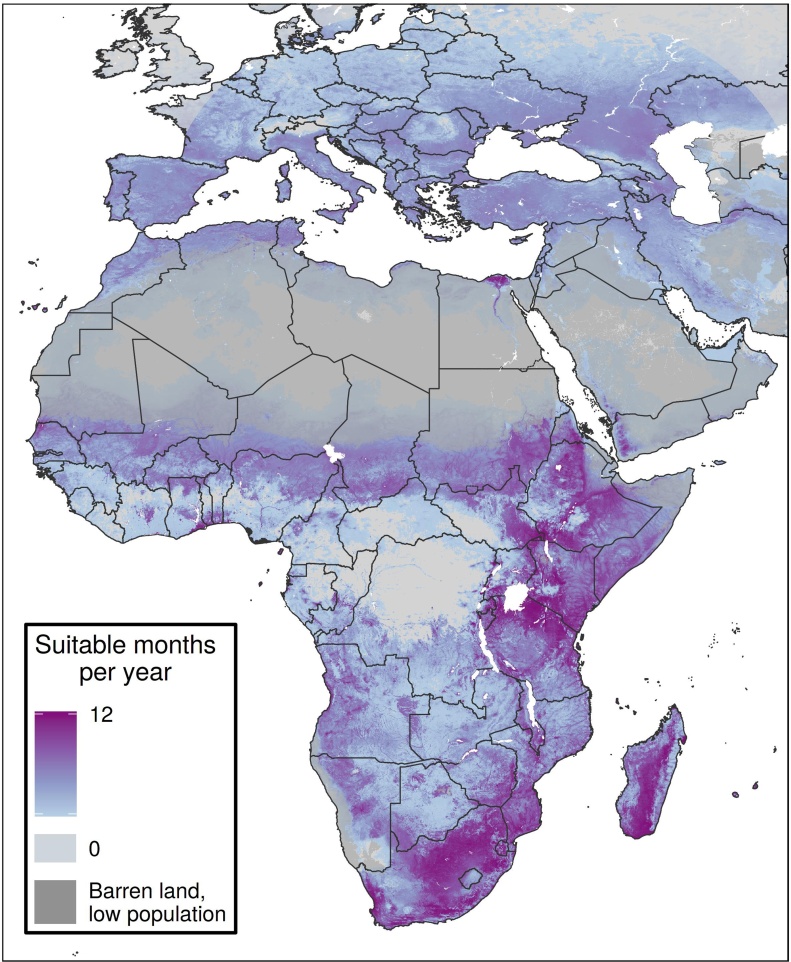
Average number of suitable months per year. Caption: The average number of suitable months per year across years 1995-2016 is shown for each 5 × 5-km pixel. A pixel was considered suitable in a month-year combination if its predicted suitability value was above an optimized threshold for that month-year. Places in darker purple were suitable for more months per year, on average.

The model had high AUC_boot_ and AUC_syn_ values (0.983, 0.899) and correctly classified 97.8% of point cases as suitable in the month and year of their occurrence (Appendix Table 5). We predicted areas to be at risk that have only reported asymptomatic serological evidence (Figure 2). Our model also predicted environmental suitability in countries with no previously reported cases, such as Ethiopia and Ghana.

### Spatio-temporal variation

In some countries, such as Uganda and Namibia, spatial variation was evident in the frequency of risk (Figure 2). In countries such as Côte d’Ivoire and Benin, some areas were never suitable for RVF, and some were frequently suitable, while in countries like Kenya and South Africa, almost all areas were suitable for much of the year. In Sudan, an RVF outbreak has been reported in the country’s north-eastern region (Hassan et al., 2011), but our model suggests that a larger area could be suitable for the disease.

Our results also show the specific months of the year when places are most suitable for RVF (Figure 3, Appendix Figures 31–42). These maps can yield temporal insights in regions that appear spatially homogenous. For example, much of southern Niger is suitable for transmission the same number of months per year (Figure 2). The monthly maps show that these months are typically August to November (Figure 3, Appendix Figures 38–41), which align with the timeline of the country’s 2016 outbreak (Lagare et al., 2019).

**Figure 3:**
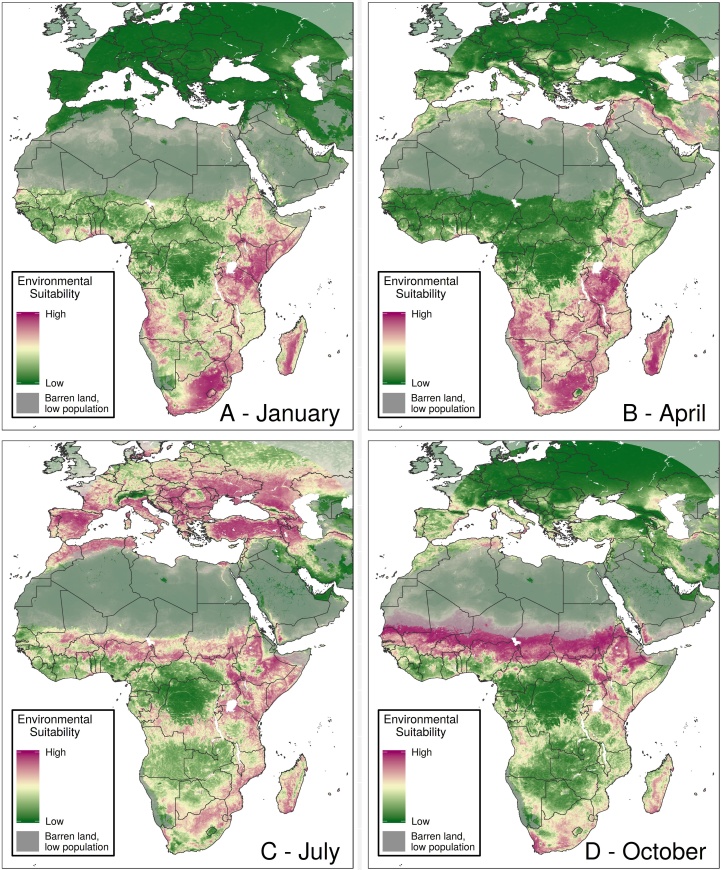
Mean monthly suitability predictions. Caption: Mean RVF suitability is shown throughout the year for January (A), April (B), July (C), and October (D). The pixel values in each monthly map represent the average suitability in that month across the years 1995-2016. Places in purple were more suitable, on average, than places in green. Maps for all 12 months are shown in Appendix Figures 31-42.

Some countries exhibit different patterns in temporal suitability. For example, in Ghana, the northern part of the country is suitable from June through October, while the south shows higher suitability from November through January. Northern Algeria and southern Côte d’Ivoire have similar average months of suitability (Figure 2), but northern Algeria experiences suitability for one long season, while southern Côte d’Ivoire is suitable for two shorter seasons during the year (Figure 3, Appendix Figures 31–42).

Our model also shows simultaneous suitability in RVF-endemic countries and those in which importation events from those endemic countries are suspected of having occurred. We show parts of Saudi Arabia that were impacted by the 2000 outbreak (Madani et al., 2003) to be suitable during June through September, which aligns with the temporal suitability of much of Turkey, where subsequent RVF detections have occurred and are suspected of having been imported via livestock from Saudi Arabia during the outbreak (Gür et al., 2017). Similarly, cases were detected in Madagascar that are suspected of having come from the December 2006-March 2007 East African outbreak (Lancelot et al., 2017), during which time both geographic regions showed environmental suitability.

### Spillover potential analysis

**Figure 4:**
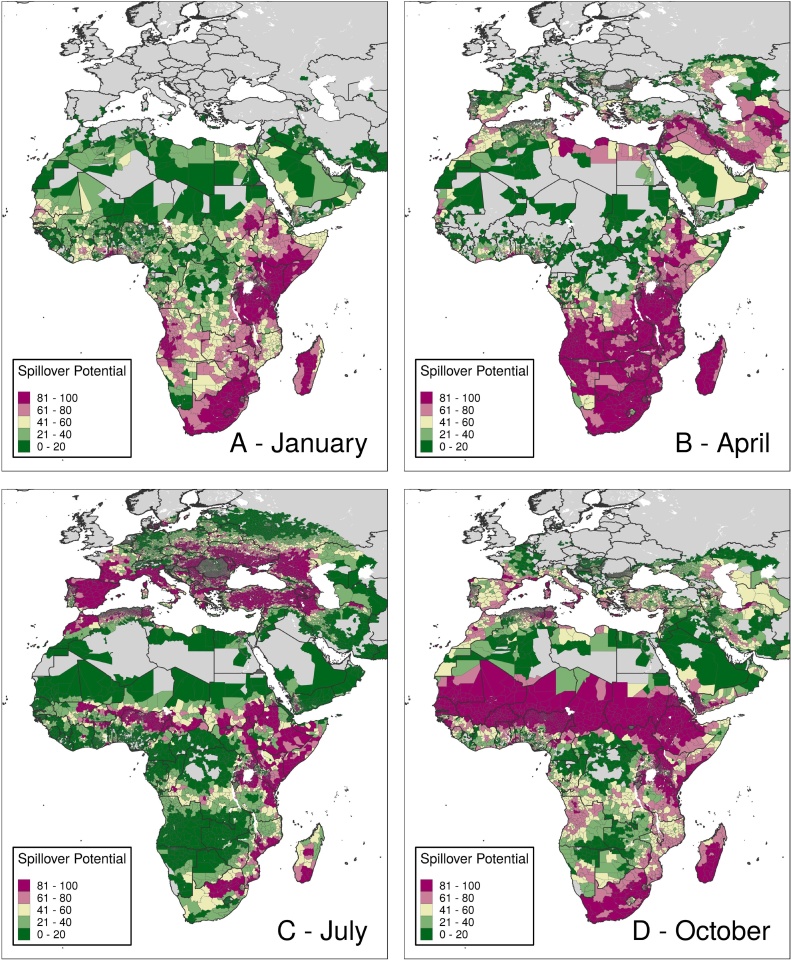
Monthly spillover value. Caption: These maps show districts’ average spillover potential during January (A), April (B), July (C), and October (D). Values in these maps represent average spillover values in that month across all years of analysis. Districts in dark purple represent districts with the highest spillover potential. Maps for all 12 months are provided in Appendix Figures 67-78.

Within individual months, spillover potential analysis provides further stratification for decision-making. For example, although Angola has fairly spatially homogenous suitability during April (Figure 3), in the south-west, suitability overlaps with high populations of humans and livestock (Figure 4). In Sudan, though most of the southern part of the country has high suitability during the latter half of the year (Figure 3), several south-eastern districts also show high spillover potential in January (Figure 4).

**Figure 5:**
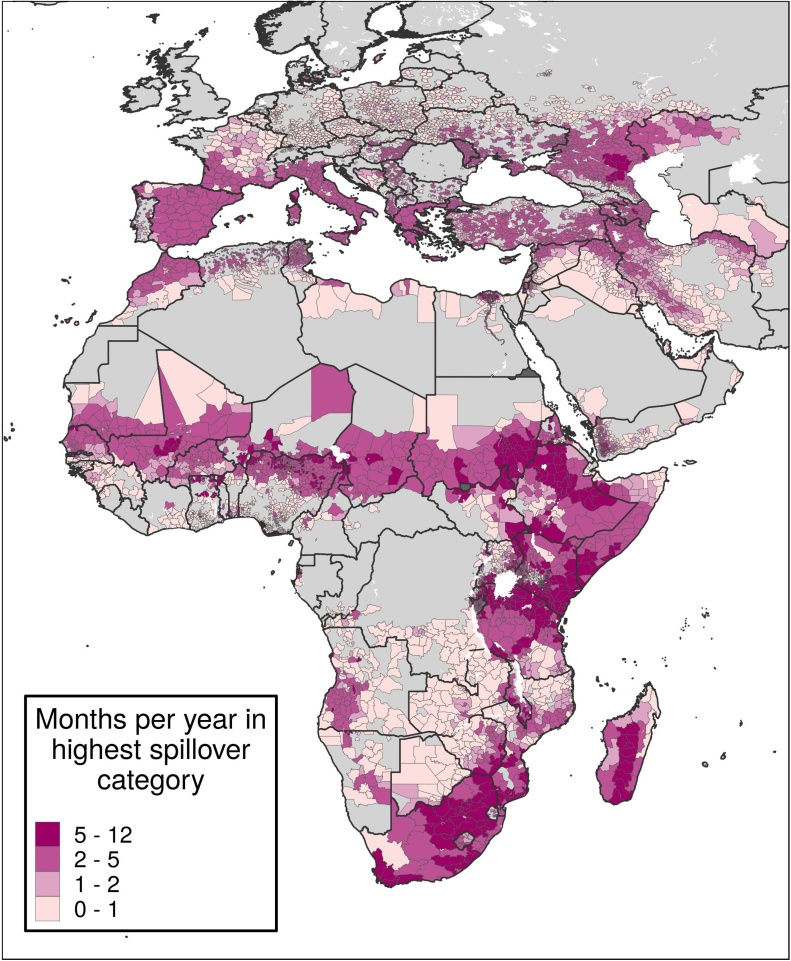
Average number of months per year in the highest spillover category. Caption: Each district is colored by how many months per year, on average, it was in the top spillover quintile of districts at risk of RVF. Districts in darker purple were in the top quintile most often. Districts in grey were never in the top quintile.

Figure 5 shows the frequency at which districts have been in the top spillover category since 1995. In some districts, such as many in south-central Africa, though suitability is rare, large populations mean that many livestock and people are at risk when areas are suitable. Elsewhere, like parts of northern Africa, areas are frequently suitable but rarely have high spillover potential due to small populations. Some countries, like Mozambique, are homogenous in terms of months per year of suitability but show high contrast in spillover potential.

## Discussion

Our results indicate that many areas throughout Africa, Europe, and the Middle East are suitable for RVF transmission, but these regions show substantial variation in when and how often they are suitable. It is critical that such spatio-temporal variation be integrated into preparedness plans for RVF. Additionally, some suitable areas have larger and denser populations of livestock and humans, increasing susceptibility to outbreaks and potential losses to human and animal life if an outbreak were to occur.

Detecting RVF in livestock and humans before outbreaks begin is critical for disease prevention (FAO, 2018). Due to finite resources, surveillance efforts must be prioritized in space and time, and our results, along with tools like the RVF Decision Support Framework (Consultative Group for RVF Decision Support, 2010) and the RVF Monitor (Anyamba et al., 2009), can inform when and where to look for cases. Our study suggests that suitable countries like Togo, which has had serological RVF detections but no symptomatic detections, could benefit from increased surveillance to enable real-time detection and response to cases, considering the virus may already be circulating. Our results could be further used by national decision-makers to identify subnational areas, such as the northern part of Togo, that are frequently suitable and in the top spillover category, which might benefit from the establishment of long-term surveillance efforts like sentinel herds (Lichoti et al., 2014) or participatory surveillance (Alhaji et al., 2018, FAO, 2018). For this specific region in Togo, March and April have little to no suitability, and these are more effective times to retrain surveillance workers and restock diagnostic labs ahead of the season of suitability. Other countries could use our results to conduct similarly tailored analyses for surveillance strengthening.

Local leaders could strengthen prevention efforts by observing where and when periods of suitability intersect with the timing of cultural behaviors that could put people and animals at risk, such as those that involve large-scale killing and transportation of livestock, as takes place in preparation for the Eid al-Adha festival. In Senegal in 2013, preparation for this festival started in August, when Senegal was highly suitable for RVF. An outbreak began and, in the following months, spread to other suitable parts of the country (Sow et al., 2016). Our results allow leaders to identify high-risk times such as these and tailor context-specific education and prevention efforts, such as those encouraging people to avoid contact with bodily fluids from potentially infected livestock.

Our spillover maps show how preparedness activities like surveillance and education might be further prioritized by highlighting where RVF could affect the largest populations of livestock, humans, or both. In the event that human or livestock vaccines are eventually made available for widespread uptake, monthly spillover maps could help countries with high spillover heterogeneity, such as Morocco, determine how best to distribute vaccines over space and time. Districts that are rarely suitable but can have high spillover potential, like many in south-central Africa, should monitor RVF forecasts and anomalous weather events like those caused by El Niño Southern Oscillations (Anyamba et al., 2012), especially during the months preceding those predicted to be most suitable, and intensify preparation accordingly.

For RVF transmission to occur in a previously unaffected area, even when the area is suitable, a disease introduction is necessary. Gilbert and colleagues described the risks associated with exporting livestock from environmentally suitable areas (Gilbert et al., 2017); seasonality could be included in this consideration. Simultaneous environmental suitability in both the exporting and importing location would be necessary for an RVF transmission cycle to begin in the new location. Our maps can help countries previously unaffected by RVF identify times when they and their trade partners are suitable for the disease, as these are the times when precautions are most necessary.

### Limitations

This study is subject to several limitations, outlined here and presented in full in the appendix (Appendix Section 6). Environmental suitability, while necessary for disease transmission, is not the only component of RVF risk. Previously unaffected countries should consider geographic proximity to previous cases and the possibility of disease importation.

Unless a source stated that a case was imported, we assumed the environment at the location of detection was similar to the environment at the point of infection. The 175 occurrence records from literature sources in our database detected by serology were all confirmed by studies detecting active RVF infection, and while IgM serology best represents active infection, the type of serological test used was not always specified. Additionally, diagnoses from serological tests may yield false positives.

Our modeling framework could not account for local factors affecting RVF epidemiology, such as vector population dynamics, watering point distributions, or livestock movements, though these should be considered. Although our estimates provide critical information for RVF preparation, the implementation of parallel surveys of vector populations – mosquitos, in particular, for RVF – in space and time is needed to provide complementary information to further stratify times and locations that are suitable for RVF. While agricultural and veterinary professionals are likely among those at the highest risk for RVF infection, due to the lack of data on their geographic distribution, our relative spillover rankings only considered total human and livestock populations. While this approach does not precisely integrate the number of livestock-related professionals in a region, it approximates the number of humans and livestock truly at risk.

Our modeling approach does not account for reporting bias; much of our input data were clustered in specific locations, although sensitivity analyses suggest the effect of this was minimal in our models (Appendix Figures 11–22; Appendix Figures 79–94). Background point sampling could also have introduced bias, though without a comprehensive understanding of which places could not experience or detect cases, weighting the sampling based on another covariate would have also introduced bias.

Finally, while we believe our model captures RVF’s climatic relationships well, further research could be done into regional, seasonal suitability patterns. Public health and veterinary health workers can triangulate our maps with other types of risk assessments and engage with local experts to make the most informed decisions for RVF prevention (Judson et al., 2018). More studies that use and produce season-specific data and results would help decision-makers to best prepare for RVF.

### Conclusion

By systematically illuminating spatial and temporal patterns in environmental suitability and analyzing how those patterns can intersect with human and livestock populations, this study can support RVF preparedness efforts. The information provided here can add value to discussions of where and when to focus RVF preparedness resources, ultimately preventing further losses to health and economies in places that have already suffered from the impacts of RVF as well as in those that have not yet detected a case.

## Author contributions

ANH, RR, and ENH extracted all occurrence data from the literature and vetted it along with SS and DMP. JDM de-duplicated literature occurrence data with the EMPRES-i database. JH cataloged all extracted data. LE prepared all covariate data for use. JCPO wrote the initial modeling code for statistical analysis, modified by ANH with input from DMP. ANH and JDM made all figures. ANH wrote the initial manuscript with assistance from JCPO, MKMP, SIH, and DMP, and all authors contributed to final revisions.

## Declaration of interests

Dr. Rabinowitz receives funding from a CDC Cooperative Agreement to study Rift Valley Fever in Kenya.

## Data sharing

Estimates can be explored using custom online data visualization tools (upon publication: http://vizhub.healthdata.org/lbd/pandemics), and are publicly available at the Global Health Data Exchange (GHDx; upon publication: http://ghdx.healthdata.org/). All data sources are indicated in Supplementary Table 2.

## Funding

This work was supported by the Bill & Melinda Gates Foundation, Seattle, WA [grant number OPP1181128].

## Ethical approval

Ethical approval was not required in this research.
