# Supplementary material for: Informing Rift Valley Fever preparedness by mapping seasonally varying environmental suitability
Source: Int J Infect Dis. 2020 Oct;99:362–72. doi: 10.1016/j.ijid.2020.07.043 (PMC7562817; doi:10.1016/j.ijid.2020.07.043)
Supplement: Supplementary file 1 [file mmc1.docx]

Supplementary Appendix for “Informing Rift Valley Fever preparedness by mapping seasonally varying environmental suitability”

Table of Contents

[List of Supplementary Figures 2](#_Toc35555543)

[List of Supplementary Tables 5](#_Toc35555544)

[1.0 GATHER compliance 6](#_Toc35555545)

[2.0 Data collection and processing 7](#_Toc35555546)

[2.1 Literature data collection and processing 7](#_Toc35555547)

[2.1.1 Extraction process 7](#_Toc35555548)

[2.1.2 Information collected from extraction 28](#_Toc35555549)

[2.1.3 Point and polygon data 28](#_Toc35555550)

[2.1.4 Date sampling 29](#_Toc35555551)

[2.2 EMPRES-i processing 31](#_Toc35555552)

[2.2.1 Locality quality processing 31](#_Toc35555553)

[2.2.2 Date sampling 31](#_Toc35555554)

[2.3 Deduplication of literature and EMPRES-i data 31](#_Toc35555555)

[2.4 Model-specific data subsets 31](#_Toc35555556)

[3.0 Modelling framework and techniques 31](#_Toc35555557)

[3.1 Background data simulation 31](#_Toc35555558)

[3.1.1 Spatial sampling 31](#_Toc35555559)

[3.1.2 Date sampling 34](#_Toc35555560)

[3.2 Modelling with boosted regression trees 34](#_Toc35555561)

[3.2.1 BRT methodology 34](#_Toc35555562)

[3.2.2 Tuning BRT hyper-parameters 35](#_Toc35555563)

[3.2.3 Model covariates 35](#_Toc35555564)

[3.2.4 Multivariate environmental similarity surface (MESS) analysis 40](#_Toc35555565)

[3.2.5 Evaluating model performance 53](#_Toc35555566)

[4.0 Results aggregation 53](#_Toc35555567)

[4.1 Suitable months per year 53](#_Toc35555568)

[4.2 Mean suitability maps 54](#_Toc35555569)

[4.3 Mean uncertainty 54](#_Toc35555570)

[4.4 Synoptic binary maps, confidence intervals, and synoptic AUC 55](#_Toc35555571)

[4.5 Spillover potential 57](#_Toc35555572)

[4.5 Mean spillover quintile 62](#_Toc35555573)

[4.6 Months per year in top spillover quintile 62](#_Toc35555574)

[5.0 Supplementary results 62](#_Toc35555575)

[5.1 Monthly mean suitability maps 62](#_Toc35555576)

[5.2 Monthly binary maps 75](#_Toc35555577)

[5.3 Monthly mean uncertainty maps 80](#_Toc35555578)

[5.4 Monthly spillover maps 96](#_Toc35555579)

[5.5 Supplementary experiments 109](#_Toc35555580)

[5.5.1 Occurrence data from detections in humans 109](#_Toc35555581)

[5.5.2 Occurrence data from detections diagnosed with PCR 112](#_Toc35555582)

[5.5.3 Occurrence data with exact date information 115](#_Toc35555583)

[6.0 Supplementary Limitations 119](#_Toc35555584)

[7.0 Supplementary references 119](#_Toc35555585)

# List of Supplementary Figures

[Appendix Figure 1: All extracted data, literature and EMPRES-i 28](#_Toc27394817)

[Appendix Figure 2: Example of year sampling distribution 30](#_Toc27394818)

[Appendix Figure 3: Example of month sampling distribution 30](#_Toc27394819)

[Appendix Figure 4: Global Burden of Disease (GBD) region definitions 32](#_Toc27394820)

[Appendix Figure 5: Datapoints geographically clustered with hierarchical clustering 33](#_Toc27394821)

[Appendix Figure 6: Buffered cluster centroids 33](#_Toc27394822)

[Appendix Figure 7: Example of single classification tree 34](#_Toc27394823)

[Appendix Figure 8: Frequency of interactions between covariates in classification trees 35](#_Toc27394824)

[Appendix Figure 9: Covariate effects (part 1 of 2) 37](#_Toc27394825)

[Appendix Figure 10: Covariate effects (part 2 of 2) 38](#_Toc27394826)

[Appendix Figure 11: January multivariate environmental similarity surface analysis 41](#_Toc27394827)

[Appendix Figure 12: February multivariate environmental similarity surface 42](#_Toc27394828)

[Appendix Figure 13: March multivariate environmental similarity surface analysis 43](#_Toc27394829)

[Appendix Figure 14: April multivariate environmental similarity surface analysis 44](#_Toc27394830)

[Appendix Figure 15: May multivariate environmental similarity surface analysis 45](#_Toc27394831)

[Appendix Figure 16: June multivariate environmental similarity surface analysis 46](#_Toc27394832)

[Appendix Figure 17: July multivariate environmental similarity surface analysis 47](#_Toc27394833)

[Appendix Figure 18: August multivariate environmental similarity surface analysis 48](#_Toc27394834)

[Appendix Figure 19: September multivariate environmental similarity surface analysis 49](#_Toc27394835)

[Appendix Figure 20: October multivariate environmental similarity surface analysis 50](#_Toc27394836)

[Appendix Figure 21: November multivariate environmental similarity surface analysis 51](#_Toc27394837)

[Appendix Figure 22: December multivariate environmental similarity surface analysis 52](#_Toc27394838)

[Appendix Figure 23: Suitable months per year aggregation 54](#_Toc27394839)

[Appendix Figure 24: Monthly suitability aggregation 54](#_Toc27394840)

[Appendix Figure 25: Mean uncertainty aggregation 55](#_Toc27394841)

[Appendix Figure 26: Occurrence data counts by month 56](#_Toc27394842)

[Appendix Figure 27: Optimised thresholds for monthly synoptic maps 57](#_Toc27394843)

[Supplemental Figure 28: Cattle population data 59](#_Toc27394844)

[Supplemental Figure 29: Sheep population data 60](#_Toc27394845)

[Supplemental Figure 30: Goat population data 61](#_Toc27394846)

[Appendix Figure 31: January mean environmental suitability 63](#_Toc27394847)

[Appendix Figure 32: February mean environmental suitability 64](#_Toc27394848)

[Appendix Figure 33: March mean environmental suitability 65](#_Toc27394849)

[Appendix Figure 34: April mean environmental suitability 66](#_Toc27394850)

[Appendix Figure 35: May mean environmental suitability 67](#_Toc27394851)

[Appendix Figure 36: June mean environmental suitability 68](#_Toc27394852)

[Appendix Figure 37: July mean environmental suitability 69](#_Toc27394853)

[Appendix Figure 38: August mean environmental suitability 70](#_Toc27394854)

[Appendix Figure 39: September mean environmental suitability 71](#_Toc27394855)

[Appendix Figure 40: October mean environmental suitability 72](#_Toc27394856)

[Appendix Figure 41: November mean environmental suitability 73](#_Toc27394857)

[Appendix Figure 42: December mean environmental suitability 74](#_Toc27394858)

[Appendix Figure 43: January binary prediction map with confidence intervals 75](#_Toc27394859)

[Appendix Figure 44: February binary prediction map with confidence intervals 75](#_Toc27394860)

[Appendix Figure 45: March binary prediction map with confidence intervals 76](#_Toc27394861)

[Appendix Figure 46: April binary prediction map with confidence intervals 76](#_Toc27394862)

[Appendix Figure 47: May binary prediction map with confidence intervals 77](#_Toc27394863)

[Appendix Figure 48: June binary prediction map with confidence intervals 77](#_Toc27394864)

[Appendix Figure 49: July binary prediction map with confidence intervals 78](#_Toc27394865)

[Appendix Figure 50: August binary prediction map with confidence intervals 78](#_Toc27394866)

[Appendix Figure 51: September binary prediction map with confidence intervals 79](#_Toc27394867)

[Appendix Figure 52: October binary prediction map with confidence intervals 79](#_Toc27394868)

[Appendix Figure 53: November binary prediction map with confidence intervals 80](#_Toc27394869)

[Appendix Figure 54: December binary prediction map with confidence intervals 80](#_Toc27394870)

[Appendix Figure 55: January mean uncertainty map 81](#_Toc27394871)

[Appendix Figure 56: February mean uncertainty map 82](#_Toc27394872)

[Appendix Figure 57: March mean uncertainty map 83](#_Toc27394873)

[Appendix Figure 58: April mean uncertainty map 84](#_Toc27394874)

[Appendix Figure 59: May mean uncertainty map 85](#_Toc27394875)

[Appendix Figure 60: June mean uncertainty map 86](#_Toc27394876)

[Appendix Figure 61: July mean uncertainty map 87](#_Toc27394877)

[Appendix Figure 62: August mean uncertainty map 88](#_Toc27394878)

[Appendix Figure 63: September mean uncertainty map 89](#_Toc27394879)

[Appendix Figure 64: October mean uncertainty map 90](#_Toc27394880)

[Appendix Figure 65: November mean uncertainty map 91](#_Toc27394881)

[Appendix Figure 66: December mean uncertainty map 92](#_Toc27394882)

[Appendix Figure 67: January mean spillover map 93](#_Toc27394883)

[Appendix Figure 68: February mean spillover map 94](#_Toc27394884)

[Appendix Figure 69: March mean spillover map 95](#_Toc27394885)

[Appendix Figure 70: April mean spillover map 96](#_Toc27394886)

[Appendix Figure 71: May mean spillover map 97](#_Toc27394887)

[Appendix Figure 72: June mean spillover map 98](#_Toc27394888)

[Appendix Figure 73: July mean spillover map 99](#_Toc27394889)

[Appendix Figure 74: August mean spillover map 100](#_Toc27394890)

[Appendix Figure 75: September mean spillover map 101](#_Toc27394891)

[Appendix Figure 76: October mean spillover map 102](#_Toc27394892)

[Appendix Figure 77: November mean spillover map 103](#_Toc27394893)

[Appendix Figure 78: December mean spillover map 104](#_Toc27394894)

[Appendix Figure 79: Map of symptomatic occurrences of Rift Valley Fever in humans 105](#_Toc27394895)

[Appendix Figure 80: January binary suitability estimates and confidence intervals modelled with only human occurrence data 106](#_Toc27394896)

[Appendix Figure 81: April binary suitability estimates and confidence intervals modelled with only human occurrence data 106](#_Toc27394897)

[Appendix Figure 82: July binary suitability estimates and confidence intervals modelled with only human occurrence data 107](#_Toc27394898)

[Appendix Figure 83: October binary suitability estimates and confidence intervals modelled with only human occurrence data 107](#_Toc27394899)

[Appendix Figure 84: Map of Rift Valley Fever occurrences diagnosed with PCR 108](#_Toc27394900)

[Appendix Figure 85: January binary suitability estimates and confidence intervals modelled with only occurrence data detected using PCR 109](#_Toc27394901)

[Appendix Figure 86: April binary suitability estimates and confidence intervals modelled with only occurrence data detected using PCR 109](#_Toc27394902)

[Appendix Figure 87: July binary suitability estimates and confidence intervals modelled with only occurrence data detected using PCR 110](#_Toc27394903)

[Appendix Figure 88: October binary suitability estimates and confidence intervals modelled with only occurrence data detected using PCR 110](#_Toc27394904)

[Appendix Figure 89: Map of Rift Valley Fever occurrences reported with exact date information 111](#_Toc27394905)

[Appendix Figure 90: January binary suitability estimates and confidence intervals modelled with only occurrence data reported with exact date information 112](#_Toc27394906)

[Appendix Figure 91: April binary suitability estimates and confidence intervals modelled with only occurrence data reported with exact date information 112](#_Toc27394907)

[Appendix Figure 92: July binary suitability estimates and confidence intervals modelled with only occurrence data reported with exact date information 113](#_Toc27394908)

[Appendix Figure 93: October binary suitability estimates and confidence intervals modelled with only occurrence data reported with exact date information 113](#_Toc27394909)

[Appendix Figure 94: Counts by month of records with exact date information 114](#_Toc27394910)

# List of Supplementary Tables

[Appendix Table 1: Guidelines for Accurate and Transparent Health Estimates Reporting (GATHER) checklist 7](#_Toc44931978)

[Appendix Table 2: Citations with geographically resolved Rift Valley Fever occurrences 9](#_Toc44931979)

[Appendix Table 3: Model hyperparameters 36](#_Toc44931980)

[Appendix Table 4: Model covariates and citations 40](#_Toc44931981)

[Appendix Table 5: Synoptic area under the curve values for monthly maps 54](#_Toc44931982)

# 1.0 GATHER compliance

Appendix Table 1: Guidelines for Accurate and Transparent Health Estimates Reporting (GATHER) checklist

| **Item #** | **Checklist item** | **Reported on page #** |
| --- | --- | --- |
| **Objectives and Funding** | | |
| **1** | Define the indicator(s), populations (including age, sex, and geographic entities), and time period(s) for which estimates were made. | Main text: Methods (overview section) |
| **2** | List the funding sources for the work. | Main text: Acknowledgments |
| **Data Inputs** | | |
| *For all data inputs from multiple sources that are synthesized as part of the study:* | | |
| **3** | Describe how the data were identified and how the data were accessed. | Main text: Methods (data intake section)  Appendix Information: Section 2.0 |
| **4** | Specify the inclusion and exclusion criteria. Identify all ad-hoc exclusions. | Main text: Methods (data intake, pre-modelling data processing sections)  Appendix Information: Section 2.0 |
| **5** | Provide information on all included data sources and their main characteristics. For each data source used, report reference information or contact name/institution, population represented, data collection method, year(s) of data collection, sex and age range, diagnostic criteria or measurement method, and sample size, as relevant. | Appendix Information: Section 2.0, Appendix Table 2 |
| **6** | Identify and describe any categories of input data that have potentially important biases (e.g., based on characteristics listed in item 5). | Main text: Discussion (limitations section) |
| *For data inputs that contribute to the analysis but were not synthesized as part of the study:* | | |
| **7** | Describe and give sources for any other data inputs. | N/A |
| *For all data inputs:* | | |
| **8** | Provide all data inputs in a file format from which data can be efficiently extracted (e.g., a spreadsheet rather than a PDF), including all relevant meta-data listed in item 5. For any data inputs that cannot be shared because of ethical or legal reasons, such as third-party ownership, provide a contact name or the name of the institution that retains the right to the data. | Available through: <http://ghdx.healthdata.org/> (upon publication) |
| **9** | Provide a conceptual overview of the data analysis method. A diagram may be helpful. | Main text: Methods (environmental suitability modelling section)  Appendix Information: Section 3.0 |
| **10** | Provide a detailed description of all steps of the analysis, including mathematical formulae. This description should cover, as relevant, data cleaning, data pre-processing, data adjustments and weighting of data sources, and mathematical or statistical model(s). | Main text: Methods (data intake, pre-modelling data processing, and environmental suitability modelling sections)  Appendix Information: Section 3.0 |
| **11** | Describe how candidate models were evaluated and how the final model(s) were selected. | Main text: Methods (environmental suitability modelling section)  Appendix Information: Section 3.0 |
| **12** | Provide the results of an evaluation of model performance, if done, as well as the results of any relevant sensitivity analysis. | Main text: Results (model performance section)  Appendix Information: Appendix Table 4 |
| **13** | Describe methods for calculating uncertainty of the estimates. State which sources of uncertainty were, and were not, accounted for in the uncertainty analysis. | Main text: Methods (pre-modelling data processing, data aggregation sections) |
| **14** | State how analytic or statistical source code used to generate estimates can be accessed. | Available through: <http://ghdx.healthdata.org/> (upon publication) |
| **Results and Discussion** | | |
| **15** | Provide published estimates in a file format from which data can be efficiently extracted. | Available through: <http://ghdx.healthdata.org/> (upon publication) |
| **16** | Report a quantitative measure of the uncertainty of the estimates (e.g. uncertainty intervals). | Available through: <http://ghdx.healthdata.org/> (upon publication) |
| **17** | Interpret results in light of existing evidence. If updating a previous set of estimates, describe the reasons for changes in estimates. | Main text: Discussion |
| **18** | Discuss limitations of the estimates. Include a discussion of any modelling assumptions or data limitations that affect interpretation of the estimates. | Main text: Discussion (limitations section) |

# 2.0 Data collection and processing

## 2.1 Literature data collection and processing

### 2.1.1 Extraction process

We searched PubMed, Scopus, Web of Science, and Embase on July 3, 2018, with the keywords "Rift Valley Fever,” “RVFV,” and “RVF”. Our search returned 17,284 citations. After removing duplicates, 9,715 citations remained. We included those published from 1995 to 2018, which were 8,273 citations. We conducted title-abstract screening and included 892 articles that had potential for geographical information about Rift Valley Fever (RVF) occurrences. We conducted full-text screening on these and found geographical information in 250 articles. We catalogued these articles in the Global Health Data Exchange (GHDx), where they are associated with an NID value. The articles from which we extracted data are listed in Table 1, and the geographical data we extracted from them are shown in Appendix Figure 1.

Appendix Table 2: Citations with geographically resolved Rift Valley Fever occurrences

The results of our systematic literature review are shown. Modelled data did not include data from every citation because some citations reported occurrences that were detected by serology after the time of infection, which is not sufficiently representative for informing the model of time of infection. These citations are catalogued in the GHDx and can be accessed using their NIDs.

| NID | Geography | Suggested citation |
| --- | --- | --- |
| 403729 | Mozambique | Muianga A, Falk K, Oludele J, Pinto G, Ali S, Tivane AT, Galano G, Gudo ES, Lagerqvist N. Serological and molecular investigation of dengue, chikungunya and rift valley fever in febrile and non-febrile patients from northern Mozambique during Dengue outbreak, 2014. Int J Infect Dis. 2016; 45: 1845. |
| 403392 | Yemen | World Health Organization. Outbreak news - Rift Valley fever, Yemen - update. WHO Wkly Epidemiol Rec. 2000; 75(40): 321. |
| 403386 | Saudi Arabia | Arishi H, Ageel A, Rahman, MA, et al. Outbreak of Rift Valley Fever - Saudi Arabia, August - October 2000. MMWR Morb Mortal Wkly Rep. 2000; 59(43): 982-986. |
| 403384 | Saudi Arabia, Yemen | Arishi H, Ageel A, Rahman, MA, et al. Outbreak of Rift Valley Fever - Saudi Arabia, August - October 2000. MMWR Morb Mortal Wkly Rep. 2000; 59(40): 905-908. |
| 403376 | Kenya | Nguku P, Sharif S, Omar A, et al. Rift Valley fever outbreak - Kenya, November 2006-January 2007. MMWR Morb Mortal Wkly Rep. 2007; 56(4): 73-75. |
| 403370 | Kenya, Somalia, Tanzania | World Health Organization. An outbreak of Rift Valley Fever, Eastern Africa, 1997-1998. WHO Wkly Epidemiol Rec. 2000; 73(15): 105-109. |
| 403366 | Yemen | World Health Organization. Outbreak of Rift Valley fever, Yemen, August-October 2000. WHO Wkly Epidemiol Rec. 2000; 75(48): 392-395. |
| 403355 | Burundi | Cooke FJ, Shapiro DS. ProMED update - Rift Valley Fever in East Africa. Int J Infect Dis. 2007; 11(4): 2878. |
| 403354 | Kenya, Somalia, Tanzania | World Health Organization. Outbreaks of Rift Valley fever in Kenya, Somalia and United Republic of Tanzania, December 2006-April 2007. WHO Wkly Epidemiol Rec. 2007; 82(20): 169-178. |
| 403352 | Tanzania | World Health Organization. Outbreak news - Rift Valley fever, United Republic of Tanzania. WHO Wkly Epidemiol Rec. 2007; 82(6): 117. |
| 403349 | Sudan | World Health Organization. Outbreak news - Rift Valley fever, Sudan - update. WHO Wkly Epidemiol Rec. 2007; 82(48): 417. |
| 403347 | Kenya | World Health Organization. Outbreak news - Rift Valley fever, Kenya. WHO Wkly Epidemiol Rec. 2007; 82(3): 17. |
| 403335 | Sudan | Cook FJ, Shapiro DS. ProMED update - Rift Valley Fever in Sudan. Int J Infect Dis. 2008; 12(1): 12. |
| 403328 | Madagascar | World Health Organization. Outbreak news - Rift Valley Fever, Madagascar. WHO Wkly Epidemiol Rec. 2008; 83(18): 157. |
| 402867 | Guinea | Konstantinov OK, Diallo SM, Inapogi AP, Ba A, Kamara SK. The mammals of Guinea as reservoirs and carriers of arboviruses. Med Parazitol (Mosk). 2006; 1: 34-9. |
| 402863 | Guinea | Butenko AM. Arbovirus circulation in the Republic of Guinea. Med Parazitol (Mosk). 1996; 2: 40-5. |
| 402861 | Democratic Republic of the Congo | Georges TM, Justin M, Victor M, Marie KJ, Mark R, Léopold MMK. Seroprevalence and Virus Activity of Rift Valley Fever in Cattle in Eastern Region of Democratic Republic of the Congo. J Vet Med. 2018; 2018: 4956378. |
| 402857 | Democratic Republic of the Congo | Tshilenge GM, Dundon WG, De Nardi M, Mulumba Mfumu LK, Rweyemamu M, Kayembe-Ntumba JM, Masumu J. Seroprevalence of Rift Valley fever virus in cattle in the Democratic Republic of the Congo. Trop Anim Health Prod. 2019; 51(3): 537-543. |
| 402850 | Madagascar | Andriamandimby SF, Randrianarivo-Solofoniaina AE, Jeanmaire EM, Ravololomanana L, Razafimanantsoa LT, Rakotojoelinandrasana T, Razainirina J, Hoffmann J, Ravalohery JP, Rafisandratantsoa JT, Rollin PE, Reynes JM. Rift Valley fever during rainy seasons, Madagascar, 2008 and 2009. Emerg Infect Dis. 2010; 16(6): 963-70. |
| 402848 | Senegal | Clements AC, Pfeiffer DU, Martin V, Pittliglio C, Best N, Thiongane Y. Spatial risk assessment of Rift Valley fever in Senegal. Vector Borne Zoonotic Dis. 2007; 7(2): 203-16. |
| 402846 | Tanzania | Mohamed M, Mosha F, Mghamba J, Zaki SR, Shieh WJ, Paweska J, Omulo S, Gikundi S, Mmbuji P, Bloland P, Zeidner N, Kalinga R, Breiman RF, Njenga MK. Epidemiologic and clinical aspects of a Rift Valley fever outbreak in humans in Tanzania, 2007. Am J Trop Med Hyg. 2010; 83(2 Suppl): 22-7. |
| 402844 | Kenya, Tanzania | Jost CC, Nzietchueng S, Kihu S, Bett B, Njogu G, Swai ES, Mariner JC. Epidemiological assessment of the Rift Valley fever outbreak in Kenya and Tanzania in 2006 and 2007. Am J Trop Med Hyg. 2010; 83(2 Suppl): 65-72. |
| 402842 | Sudan | Salim RW, Khairalla KMS, Eljamal AA, Karrar AE, Aradaib IE. A Single-Tube RT-PCR Amplification for Detection of Rift Valley Fever Virus. Res J Med Sci. 2010; 4(3): 14651. |
| 402838 | South Africa | Paweska JT, Barnard BJ, Williams R. The use of sucrose-acetone-extracted Rift Valley fever virus antigen derived from cell culture in an indirect enzyme-linked immunosorbent assay and haemagglutination-inhibition test. Onderstepoort J Vet Res. 1995; 62(4): 227-33. |
| 402834 | Mauritania | Zeller HG, Akakpo AJ, Ba MM. Rift Valley fever epizootic in small ruminants in southern Mauritania (October 1993): risk of extensive outbreaks. Ann Soc Belg Med Trop. 1995; 75(2): 135-40. |
| 402832 | Senegal | Fontenille D, Traore-Lamizana M, Zeller H, Mondo M, Diallo M, Digoutte JP. Short report: Rift Valley fever in western Africa: isolations from Aedes mosquitoes during an interepizootic period. Am J Trop Med Hyg. 1995; 52(5): 403-4. |
| 402828 | Benin, Cameroon, Côte d’Ivoire, Senegal, Togo | Zeller HG, Bessin R, Thiongane Y, Bapetel I, Teou K, Ala MG, Atse AN, Sylla R, Digoutte JP, Akakpo JA. Rift Valley fever antibody prevalence in domestic ungulates in Cameroon and several west African countries (1989-1992) following the 1987 Mauritanian outbreak. Res Virol. 1995; 146(1): 81-5. |
| 402826 | Niger | Mariner JC, Morrill J, Ksiazek TG. Antibodies to hemorrhagic fever viruses in domestic livestock in Niger: Rift Valley fever and Crimean-Congo hemorrhagic fever. Am J Trop Med Hyg. 1995; 53(3): 217-21. |
| 402492 | Madagascar | Jeanmaire EM, Rabenarivahiny R, Biarmann M, Rabibisoa L, Ravaomanana F, Randriamparany T, Andriamandimby SF, Diaw CS, Fenozara P, de La Rocque S, Reynes JM. Prevalence of Rift Valley fever infection in ruminants in Madagascar after the 2008 outbreak. Vector Borne Zoonotic Dis. 2011; 11(4): 395-402. |
| 402488 | Nigeria | Olaleye OD, Tomori O, Ladipo MA, Schmitz H. Rift Valley fever in Nigeria: infections in humans. Rev Sci Tech. 1996; 15(3): 923-35. |
| 402484 | Nigeria | Olaleye OD, Tomori O, Schmitz H. Rift Valley fever in Nigeria: infections in domestic animals. Rev Sci Tech. 1996; 15(3): 937-46. |
| 402482 | Kenya, Tanzania | House C, Alexander KA, Kat PW, O'Brien SJ, Mangiafico J. Serum antibody to Rift Valley fever virus in African carnivores. Ann N Y Acad Sci. 1996; 791: 345-9. |
| 402478 | Central African Republic | Guilherme JM, Gonella-Legall C, Legall F, Nakoume E, Vincent J. Seroprevalence of five arboviruses in Zebu cattle in the Central African Republic. Trans R Soc Trop Med Hyg. 1996; 90(1): 31-3. |
| 402466 | Egypt | Abu-Elyazeed R, el-Sharkawy S, Olson J, Botros B, Soliman A, Salib A, Cummings C, Arthur R. Prevalence of anti-Rift-Valley-fever IgM antibody in abattoir workers in the Nile delta during the 1993 outbreak in Egypt. Bull World Health Organ. 1996; 74(2): 155-8. |
| 402464 | Sudan | McCarthy MC, Haberberger RL, Salib AW, Soliman BA, El-Tigani A, Khalid IO, Watts DM. Evaluation of arthropod-borne viruses and other infectious disease pathogens as the causes of febrile illnesses in the Khartoum Province of Sudan. J Med Virol. 1996; 48(2): 141-6. |
| 402462 | Zambia | Samui KL, Inoue S, Mweene AS, Nambota AM, Mlangwa JE, Chilonda P, Onuma M, Morita C. Distribution of Rift Valley fever among cattle in Zambia. Jpn J Med Sci Biol. 1997; 50(2): 73-7. |
| 402460 | Guinea, Mauritania, Senegal | Sall AA, de A Zanotto PM, Zeller HG, Digoutte JP, Thiongane Y, Bouloy M. Variability of the NS(S) protein among Rift Valley fever virus isolates. J Gen Virol. 1997; 78(Pt 11): 2853-8. |
| 402458 | Egypt | Elwan MS, Sharaf MH, Gameel K, El-Hadi E, Arthur RR. Rift valley fever retinopathy: Observations in a new outbreak. Ann Saudi Med. 1997; 17(3): 377-80. |
| 402456 | Senegal | Zeller HG, Fontenille D, Traore-Lamizana M, Thiongane Y, Digoutte JP. Enzootic activity of Rift Valley fever virus in Senegal. Am J Trop Med Hyg. 1997; 56(3): 265-72. |
| 402454 | Burkina Faso, Central African Republic, Guinea, Mauritania, Nigeria, Senegal | Fontenille D, Traore-Lamizana M, Diallo M, Thonnon J, Digoutte JP, Zeller HG. New vectors of Rift Valley fever in West Africa. Emerg Infect Dis. 1998; 4(2): 289-93. |
| 402452 | Senegal | Thonnon J, Picquet M, Thiongane Y, Lo M, Sylla R, Vercruysse J. Rift valley fever surveillance in the lower Senegal river basin: update 10 years after the epidemic. Trop Med Int Health. 1999; 4(8): 580-5. |
| 402450 | Egypt | Abd el-Rahim IH, Abd el-Hakim U, Hussein M. An epizootic of Rift Valley fever in Egypt in 1997. Rev Sci Tech. 1999; 18(3): 741-8. |
| 402448 | Burkina Faso, Central African Republic, Egypt, Guinea, Kenya, Madagascar, Mauritania, Senegal, Uganda | Sall AA, Zanotto PM, Sene OK, Zeller HG, Digoutte JP, Thiongane Y, Bouloy M. Genetic reassortment of Rift Valley fever virus in nature. J Virol. 1999; 73(10): 8196-200. |
| 402446 | Louga | Gora D, Yaya T, Jocelyn T, Didier F, Maoulouth D, Amadou S, Ruel TD, Gonzalez JP. The potential role of rodents in the enzootic cycle of Rift Valley fever virus in Senegal. Microbes Infect. 2000; 2(4): 343-6. |
| 402444 | Central African Republic | Nakounné E, Selekon B, Morvan J. Microbiological surveillance: viral hemorrhagic fever in Central African Republic: current serological data in man. Bull Soc Pathol Exot. 2000; 93(5): 340-7. |
| 402442 | Saudi Arabia, Yemen | Ahmad K. More deaths from Rift Valley fever in Saudi Arabia and Yemen. Lancet. 2000; 356(9239): 1422. |
| 402417 | Egypt | El-Esnawy, Nagwa A. Infection by certain arboviruses among workers potentially at risk of infection. J Egypt Public Health Assoc. 2001; 76(3-4): 169-82. |
| 402415 | Egypt | Youssef BZ. Application of reverse transcriptase-polymerase chain reaction for detection of Rift Valley Fever viral antigen from mosquito. J Egypt Public Health Assoc. 2001; 76(3-4): 297-308. |
| 402410 | Senegal | Diallo M, Lochouarn L, Ba K, Sall AA, Mondo M, Girault L, Mathiot C. First isolation of the Rift Valley fever virus from Culex poicilipes (Diptera: Culicidae) in nature. Am J Trop Med Hyg. 2000; 62(6): 702-4. |
| 402408 | Saudi Arabia | Turkistany AH, Mohamed AG, Al-Hamdan N. Seroprevalence of Rift Valley Fever among slaughterhouse personnel in Makkah during Hajj 1419h (1999). J Fam Community Med. 2001; 8(3): 53-7. |
| 402406 | Mauritania | Nabeth P, Kane Y, Abdalahi MO, Diallo M, Ndiaye K, Ba K, Schneegans F, Sall AA, Mathiot C. Rift Valley fever outbreak, Mauritania, 1998: seroepidemiologic, virologic, entomologic, and zoologic investigations. Emerg Infect Dis. 2001; 7(6): 1052-4. |
| 402404 | Senegal | Traoré-Lamizana M, Fontenille D, Diallo M, Bâ Y, Zeller HG, Mondo M, Adam F, Thonon J, Maïga A. Arbovirus surveillance from 1990 to 1995 in the Barkedji area (Ferlo) of Senegal, a possible natural focus of Rift Valley fever virus. J Med Entomol. 2001; 38(4): 480-92. |
| 401797 | Egypt | Youssef BZ, Donia HA. The potential role of rattus rattus in enzootic cycle of Rift Valley Fever in Egypt 2-application of reverse transcriptase polymerase chain reaction (RT-PCR) in blood samples of Rattus rattus. J Egypt Public Health Assoc. 2002; 77(1-2): 133-41. |
| 401771 | Saudi Arabia | Miller BR, Godsey MS, Crabtree MB, Savage HM, Al-Mazrao Y, Al-Jeffri MH, Abdoon AM, Al-Seghayer SM, Al-Shahrani AM, Ksiazek TG. Isolation and genetic characterization of Rift Valley fever virus from Aedes vexans arabiensis, Kingdom of Saudi Arabia. Emerg Infect Dis. 2002; 8(12): 1492-4. |
| 401769 | Saudi Arabia | Jupp PG, Kemp A, Grobbelaar A, Lema P, Burt FJ, Alahmed AM, Al Mujalli D, Al Khamees M, Swanepoel R. The 2000 epidemic of Rift Valley fever in Saudi Arabia: mosquito vector studies. Med Vet Entomol. 2002; 16(3): 245-52. |
| 401767 | Kenya | Woods CW, Karpati AM, Grein T, McCarthy N, Gaturuku P, Muchiri E, Dunster L, Henderson A, Khan AS, Swanepoel R, Bonmarin I, Martin L, Mann P, Smoak BL, Ryan M, Ksiazek TG, Arthur RR, Ndikuyeze A, Agata NN, Peters CJ, World Health Organization Hemorrhagic Fever Task Force. An outbreak of Rift Valley fever in Northeastern Kenya, 1997-98. Emerg Infect Dis. 2002; 8(2): 138-44. |
| 401764 | Chad | Durand JP, Bouloy M, Richecoeur L, Peyrefitte CN, Tolou H. Rift Valley fever virus infection among French troops in Chad. Emerg Infect Dis. 2003; 9(6): 751-2. |
| 401027 | Saudi Arabia | Madani TA, Al-Mazrou YY, Al-Jeffri MH, Mishkhas AA, Al-Rabeah AM, Turkistani AM, Al-Sayed MO, Abodahish AA, Khan AS, Ksiazek TG, Shobokshi O. Rift Valley fever epidemic in Saudi Arabia: epidemiological, clinical, and laboratory characteristics. Clin Infect Dis. 2003; 37(8): 1084-92. |
| 401025 | Saudi Arabia | Al-Khuwaitir TS, Al-Moghairi AM, Sherbeeni SM, Al-Ghamdi AS. Rift Valley fever hepatitis complicated by disseminated intravascular coagulation and hepatorenal syndrome. Saudi Med J. 2004; 25(4): 528-31. |
| 401022 | Chad | Ringot D, Durand JP, Toulou H, Boutin JP, Davoust B. Rift Valley fever in Chad. Emerg Infect Dis. 2004; 10(5): 945-7. |
| 401020 | Saudi Arabia | Alrajhi AA, Al-Semari A, Al-Watban J. Rift Valley fever encephalitis. Emerg Infect Dis. 2004; 10(3): 554-5. |
| 401018 | Senegal | Chevalier V, Lancelot R, Thiongane Y, Sall B, Diaité A, Mondet B. Rift Valley fever in small ruminants, Senegal, 2003. Emerg Infect Dis. 2005; 11(11): 1693-700. |
| 401016 | Saudi Arabia | Al-Hazmi A, Al-Rajhi AA, Abboud EB, Ayoola EA, Al-Hazmi M, Saadi R, Ahmed N. Ocular complications of Rift Valley fever outbreak in Saudi Arabia. 2005; 112(2): 313-8. |
| 401014 | Cameroon | LeBreton M, Umlauf S, Djoko CF, Daszak P, Burke DS, Kwenkam PY, Wolfe ND. Rift Valley fever in goats, Cameroon. Emerg Infect Dis. 2006; 12(4): 702-3. |
| 401012 | Senegal | Marrama L, Spiegel A, Ndiaye K, Sall AA, Gomes E, Diallo M, Thiongane Y, Mathiot C, Gonzalez JP. Domestic transmission of Rift Valley Fever virus in Diawara (Senegal) in 1998. Southeast Asian J Trop Med Public Health. 2005; 36(6): 1487-95. |
| 401010 | Saudi Arabia | Arishi HM, Aqeel AY, Al Hazmi MM. Vertical transmission of fatal Rift Valley fever in a newborn. Ann Trop Paediatr. 2006; 26(3): 251-3. |
| 401008 | Saudi Arabia | Elfadil AA, Hasab-Allah KA, Dafa-Allah OM, Elmanea AA. The persistence of rift valley fever in the Jazan region of Saudi Arabia. Rev Sci Tech. 2006; 25(3): 1131-6. |
| 401001 | Kenya | LaBeaud AD, Peters CJ, Muchiri EM, King CH. Late outcomes of Rift Valley Fever in Kenya: Ijara clinical survey. Am J Trop Med Hyg. 2006; 75(5 Suppl): 192. |
| 400999 | Saudi Arabia | Elfadil AAM, Musa SM, Elmujalli DM, Elkhamis MH, Elahmed KS. Epidemiologic Study On Rift Valley Fever In The South-West Of Kingdom Of Saudi Arabia. Sudan Journal of Science and Technology. 2004; 5(1): 110-19. |
| 400996 | Saudi Arabia | Elfadil AA, Hasab-Allah KA, Dafa-Allah OM. Factors associated with rift valley fever in south-west Saudi Arabia. Rev Sci Tech. 2006; 25(3): 1137-45. |
| 400994 | Yemen | Abdo-Salem S, Gerbier G, Bonnet P, Al-Qadasi M, Tran A, Thiry E, Al-Eryni G, Roger F. Descriptive and spatial epidemiology of Rift valley fever outbreak in Yemen 2000-2001. Ann N Y Acad Sci. 2006; 1081: 240-2. |
| 400992 | South Africa | LaBeaud AD, Cross PC, Getz WM, Glinka A, King CH. Rift Valley fever virus infection in African buffalo (Syncerus caffer) herds in rural South Africa: evidence of interepidemic transmission. Am J Trop Med Hyg. 2011; 84(4): 641-6. |
| 400990 | Saudi Arabia | Al-Afaleq AI, Abu-Elzein EME, Hegazy AA, Al-Naeem A. Serosurveillance of camels (Camelus Dromedarius) to detect antibodies against viral diseases in Saudi Arabia. J Camel Pract Res. 2007; 14(2): 9196. |
| 400987 | Kenya | LaBeaud AD, Ochiai Y, Peters CJ, Muchiri EM, King CH. Spectrum of Rift Valley fever virus transmission in Kenya: insights from three distinct regions. Am J Trop Med Hyg. 2007; 76(5): 795-800. |
| 400984 | Somalia | Soumare B, Tempia S, Cagnolati V, Mohamoud A, Van Huylenbroeck G, Berkvens D. Screening for Rift Valley fever infection in northern Somalia: a GIS based survey method to overcome the lack of sampling frame. Vet Microbiol. 2007; 121(3-4): 249-56. |
| 400804 | Mauritania, Senegal | Faye O, Diallo M, Diop D, Bezeid OE, Bâ H, Niang M, Dia I, Mohamed SA, Ndiaye K, Diallo D, Ly PO, Diallo B, Nabeth P, Simon F, Lô B, Diop OM. Rift Valley fever outbreak with East-Central African virus lineage in Mauritania, 2003. Emerg Infect Dis. 2007; 13(7): 1016-23. |
| 400802 | United Arab Emirates | Wernery U, Thomas R, Raghavan R, Syriac G, Joseph S, Georgy N. Seroepidemiological studies for the detection of antibodies against 8 infectious diseases in dairy dromedaries of the United Arab Emirates using modern laboratory techniques - Part II. J Camel Pract Res. 2008; 15(2): 13945. |
| 400800 | Kenya | Fabiansen C, Thybo S. Hemorrhagic Rift Valley fever. Ugeskr Laeger. 2007; 169(26): 2537-8. |
| 400798 | Kenya | Evans A, Gakuya F, Paweska JT, Rostal M, Akoolo L, Van Vuren PJ, Manyibe T, Macharia JM, Ksiazek TG, Feikin DR, Breiman RF, Kariuki Njenga M. Prevalence of antibodies against Rift Valley fever virus in Kenyan wildlife. Epidemiol Infect. 2008; 136(9): 1261-9. |
| 400796 | Kenya | Bird BH, Githinji JW, Macharia JM, Kasiiti JL, Muriithi RM, Gacheru SG, Musaa JO, Towner JS, Reeder SA, Oliver JB, Stevens TL, Erickson BR, Morgan LT, Khristova ML, Hartman AL, Comer JA, Rollin PE, Ksiazek TG, Nichol ST. Multiple virus lineages sharing recent common ancestry were associated with a Large Rift Valley fever outbreak among livestock in Kenya during 2006-2007. J Virol. 2008; 82(22): 11152-66. |
| 400794 | Kenya | LaBeaud AD, Muchiri EM, Ndzovu M, Mwanje MT, Muiruri S, Peters CJ, King CH. Interepidemic Rift Valley fever virus seropositivity, northeastern Kenya. Emerg Infect Dis. 2008; 14(8): 1240-6. |
| 400792 | Egypt | Youssef BZ. The potential role of pigs in the enzootic cycle of rift valley Fever at Alexandria governorate, Egypt. J Egypt Public Health Assoc. 2009; 84(3-4): 331-44. |
| 400686 | Germany, Kenya | Oltmann A, Kämper S, Staeck O, Schmidt-Chanasit J, Günther S, Berg T, Frank C, Krüger DH, Hofmann J. Fatal outcome of hepatitis A virus (HAV) infection in a traveler with incomplete HAV vaccination and evidence of Rift Valley Fever virus infection. J Clin Microbiol. 2008; 46(11): 3850-2. |
| 400684 | Sudan | Adam I, Karsany MS. Case report: Rift Valley Fever with vertical transmission in a pregnant Sudanese woman. J Med Virol. 2008; 80(5): 929. |
| 400682 | Mayotte | Sissoko D, Giry C, Gabrie P, Tarantola A, Pettinelli F, Collet L, D’Ortenzio E, Renault P, Pierre V. Rift Valley fever, Mayotte, 2007-2008. Emerg Infect Dis. 2009; 15(4): 568-70. |
| 400680 | Tanzania | Swai ES, Schoonman L. Prevalence of Rift Valley fever immunoglobulin G antibody in various occupational groups before the 2007 outbreak in Tanzania. Vector Borne Zoonotic Dis. 2009; 9(6): 579-82. |
| 400678 | Kenya | Sutherland LJ, Muiruri S, Muchiri EM, Gray LR, Zimmerman PA, Hise AG, King CH, LaBeaud AD. Prevalence and fine-scale distribution of rift valley fever virus and West Nile virus in mosquitoes during a rift valley fever outbreak in Northeastern Province, Kenya. Am J Trop Med Hyg. 2009; 81(5): 13. |
| 400676 | Kenya, Somalia, Tanzania | Anyamba A, Chretien JP, Small J, Tucker CJ, Formenty PB, Richardson JH, Britch SC, Schnabel DC, Erickson RL, Linthicum KJ. Prediction of a Rift Valley fever outbreak. Proc Natl Acad Sci U S A. 2009; 106(3): 955-9. |
| 400674 | Mayotte | Cêtre-Sossah C, Billecocq A, Lancelot R, Defernez C, Favre J, Bouloy M, Martinez D, Albina E. Evaluation of a commercial competitive ELISA for the detection of antibodies to Rift Valley fever virus in sera of domestic ruminants in France. Prev Vet Med. 2009; 90(1-2): 146-9. |
| 400672 | South Africa | Wolhuter J, Bengis RG, Reilly BK, Cross PC. Clinical demodicosis in African buffalo (Syncerus caffer) in the Kruger National Park. J Wildl Dis. 2009; 45(2): 502-4. |
| 400670 | Sudan | El Imam M, El Sabiq M, Omran M, Abdalkareem A, El Gaili Mohamed MA, Elbashir A, Khalafala O. Acute renal failure associated with the Rift Valley fever: a single center study. Saudi J Kidney Dis Transpl. 2009; 20(6): 1047-52. |
| 400668 | Kenya | Kahlon SS, Peters CJ, Leduc J, Muchiri EM, Muiruri S, Njenga MK, Breiman RF, White AC Jr, King CH. Severe Rift Valley fever may present with a characteristic clinical syndrome. Am J Trop Med Hyg. 2010; 82(3): 371-5. |
| 400666 | Sudan | Seufi AM, Galal FH. Role of Culex and Anopheles mosquito species as potential vectors of rift valley fever virus in Sudan outbreak, 2007. BMC Infect Dis. 2010; 10: 65. |
| 400664 | Sudan | Abdelgadir DM, Bashab HMM, Mohamed RAE, Abuelmaali SA. Risk Factor Analysis for Outbreak of Rift Valley Fever in Khartoum State of Sudan. J Entomol Sci. 2010; 45(3): 239-51. |
| 400478 | South Africa | World Health Organization. Outbreak news - Rift Valley fever, South Africa - update. WHO Wkly Epidemiol Rec. 2010; 85(21): 185-86. |
| 400474 | Kenya | Munyua P, Murithi RM, Wainwright S, Githinji J, Hightower A, Mutonga D, Macharia J, Ithondeka PM, Musaa J, Breiman RF, Bloland P, Njenga MK. Rift Valley fever outbreak in livestock in Kenya, 2006-2007. Am J Trop Med Hyg. 2010; 83(2 Suppl): 58-64. |
| 400472 | Kenya | Rostal MK, Evans AL, Sang R, Gikundi S, Wakhule L, Munyua P, Macharia J, Feikin DR, Breiman RF, Njenga MK. Identification of potential vectors of and detection of antibodies against Rift Valley fever virus in livestock during interepizootic periods. Am J Vet Res. 2010; 71(5): 522-6. |
| 400470 | Sudan | Hassanain AM, Noureldien W, Karsany MS, Saeed el NS, Aradaib IE, Adam I. Rift Valley Fever among febrile patients at New Halfa hospital, eastern Sudan. Virol J. 2010; 7: 97. |
| 400424 | Sudan | Adam AA, Karsany MS, Adam I. Manifestations of severe Rift Valley fever in Sudan. Int J Infect Dis. 2010; 14(2): e179-80. |
| 400417 | Saudi Arabia | Al-Qabati AG, Al-Afaleq AI. Cross-Sectional, Longitudinal and Prospective Epidemiological Studies of Rift Valley Fever in Al-Hasa Oasis, Saudi Arabia. Journal of Animal and Veterinary Advances. 2010; 9(2): 25865. |
| 400413 | Kenya | Nguku PM, Sharif SK, Mutonga D, Amwayi S, Omolo J, Mohammed O, Farnon EC, Gould LH, Lederman E, Rao C, Sang R, Schnabel D, Feikin DR, Hightower A, Njenga MK, Breiman RF. An investigation of a major outbreak of Rift Valley fever in Kenya: 2006-2007. Am J Trop Med Hyg. 2010; 83(2 Suppl): 5-13. |
| 400411 | Madagascar | Raveloson NE, Ramorasata JC, Rasolofohanitrininosy R, Rakotoativony ST, Andrianjatovo JJ, Sztark F. Fatal haemorrhagic Rift valley fever: a case at Madagascar. Med Trop (Mars). 2010; 70(2): 177-9. |
| 400407 | Mauritania | El Mamy AB, Baba MO, Barry Y, Isselmou K, Dia ML, El Kory MO, Diop M, Lo MM, Thiongane Y, Bengoumi M, Puech L, Plee L, Claes F, de La Rocque S, Doumbia B. Unexpected Rift Valley fever outbreak, northern Mauritania. Emerg Infect Dis. 2011; 17(10): 1894-6. |
| 400404 | Comoros, Djibouti, Mauritania | Fernandez JC, Billecocq A, Durand JP, Cêtre-Sossah C, Cardinale E, Marianneau P, Pépin M, Tordo N, Bouloy M. The nonstructural protein NSs induces a variable antibody response in domestic ruminants naturally infected with Rift Valley fever virus. Clin Vaccine Immunol. 2012; 19(1): 5-10. |
| 400402 | South Africa | Miller M, Buss P, Joubert J, Maseko N, Hofmeyr M, Gerdes T. Serosurvey for selected viral agents in white rhinoceros (Ceratotherium simum) in Kruger National Park, 2007. J Zoo Wildl Med. 2011; 42(1): 29-32. |
| 400400 | Saudi Arabia | Memish ZA, Albarrak A, Almazroa MA, Al-Omar I, Alhakeem R, Assiri A, Fagbo S, MacNeil A, Rollin PE, Abdullah N, Stephens G. Seroprevalence of Alkhurma and other hemorrhagic fever viruses, Saudi Arabia. Emerg Infect Dis. 2011; 17(12): 2316-8. |
| 400398 | Kenya | Mease LE, Coldren RL, Musila LA, Prosser T, Ogolla F, Ofula VO, Schoepp RJ, Rossi CA, Adungo N. Seroprevalence and distribution of arboviral infections among rural Kenyan adults: a cross-sectional study. Virol J. 2011; 8: 371. |
| 400358 | Kenya | Grossi-Soyster EN, Banda T, Teng CY, Muchiri EM, Mungai PL, Mutuku FM, Gildengorin G, Kitron U, King CH, Desiree Labeaud A. Rift Valley Fever Seroprevalence in Coastal Kenya. Am J Trop Med Hyg. 2017; 97(1): 115-120. |
| 399720 | Kenya, Somalia, Tanzania | Nderitu L, Lee JS, Omolo J, Omulo S, O’Guinn ML, Hightower A, Mosha F, Mohamed M, Munyua P, Nganga Z, Hiett K, Seal B, Feikin DR, Breiman RF, Njenga MK. Sequential Rift Valley fever outbreaks in eastern Africa caused by multiple lineages of the virus. J Infect Dis. 2011; 203(5): 655-65. |
| 399716 | Morocco, Western Sahara | El-Harrak M, Martín-Folgar R, Llorente F, Fernández-Pacheco P, Brun A, Figuerola J, Jiménez-Clavero MA. Rift Valley and West Nile virus antibodies in camels, North Africa. Emerg Infect Dis. 2011; 17(12): 2372-4. |
| 399712 | Kenya | LaBeaud AD, Muiruri S, Sutherland LJ, Dahir S, Gildengorin G, Morrill J, Muchiri EM, Peters CJ, King CH. Postepidemic analysis of Rift Valley fever virus transmission in northeastern Kenya: a village cohort study. PLoS Negl Trop Dis. 2011; 5(8): e1265. |
| 399708 | Kenya | LaBeaud AD, Sutherland LJ, Muiruri S, Muchiri EM, Gray LR, Zimmerman PA, Hise AG, King CH. Arbovirus prevalence in mosquitoes, Kenya. Emerg Infect Dis. 2011; 17(2): 233-41. |
| 399704 | Madagascar | Chevalier V, Rakotondrafara T, Jourdan M, Heraud JM, Andriamanivo HR, Durand B, Ravaomanana J, Rollin PE, Rakotondravao R. An unexpected recurrent transmission of Rift Valley fever virus in cattle in a temperate and mountainous area of Madagascar. PLoS Negl Trop Dis. 2011; 5(12): e1423. |
| 399702 | Madagascar | Schwarz NG, Girmann M, Randriamampionona N, Bialonski A, Maus D, Krefis AC, Njarasoa C, Rajanalison JF, Ramandrisoa HD, Randriarison ML, May J, Schmidt-Chanasit J, Rakotozandrindrainy R. Seroprevalence of antibodies against Chikungunya, Dengue, and Rift Valley fever viruses after febrile illness outbreak, Madagascar. Emerg Infect Dis. 2012; 18(11): 1780-6. |
| 399698 | Saudi Arabia | Al-Afaleq AI, Hussein MF, Al-Naeem AA, Housawi F, Kabati AG. Seroepidemiological study of Rift Valley fever (RVF) in animals in Saudi Arabia. Trop Anim Health Prod. 2012; 44(7): 1535-9. |
| 399694 | Saudi Arabia | Al-Azraqi TA, El Mekki AA, Mahfouz AA. Rift Valley Fever in Southwestern Saudi Arabia: a sero-epidemiological study seven years after the outbreak of 2000-2001. Acta Trop. 2012; 123(2): 111-6. |
| 399693 | Mauritania | World Health Organization. Outbreak news. Rift Valley fever, Mauritania. WHO Wkly Epidemiol Rec. 2012; 45(87): 438. |
| 399691 | Kenya | Grolla A, Mehedi M, Lindsay R, Bosio C, Duse A, Feldmann H. Enhanced detection of Rift Valley fever virus using molecular assays on whole blood samples. J Clin Virol. 2012; 54(4): 313-7. |
| 399689 | Kenya | Mwaengo D, Lorenzo G, Iglesias J, Warigia M, Sang R, Bishop RP, Brun A. Detection and identification of Rift Valley fever virus in mosquito vectors by quantitative real-time PCR. Virus Res. 2012; 169(1): 137-43. |
| 399686 | Uganda | de St Maurice A, Harmon J, Nyakarahuka L, Balinandi S, Tumusiime A, Kyondo J, Mulei S, Namutebi A, Knust B, Shoemaker T, Nichol ST, McElroy AK, Spiropoulou CF. Rift valley fever viral load correlates with the human inflammatory response and coagulation pathway abnormalities in humans with hemorrhagic manifestations. PLoS Negl Trop Dis. 2018; 12(5): e0006460. |
| 399684 | Tanzania | Sindato C, Karimuribo ED, Pfeiffer DU, Mboera LE, Kivaria F, Dautu G, Bernard B, Paweska JT. Spatial and temporal pattern of Rift Valley fever outbreaks in Tanzania; 1930 to 2007. PLoS One. 2014; 9(2): e88897. |
| 399682 | Egypt | Horton KC, Wasfy M, Samaha H, Abdel-Rahman B, Safwat S, Abdel Fadeel M, Mohareb E, Dueger E. Serosurvey for zoonotic viral and bacterial pathogens among slaughtered livestock in Egypt. Vector Borne Zoonotic Dis. 2014; 14(9): 633-9. |
| 399680 | Chad | Abakar MF, Naré NB, Schelling E, Hattendorf J, Alfaroukh IO, Zinsstag J. Seroprevalence of Rift Valley fever, Q fever, and brucellosis in ruminants on the southeastern shore of Lake Chad. Vector Borne Zoonotic Dis. 2014; 14(10): 757-62. |
| 399678 | South Africa | Fagbo S, Coetzer JA, Venter EH. Seroprevalence of Rift Valley fever and lumpy skin disease in African buffalo (Syncerus caffer) in the Kruger National Park and Hluhluwe-iMfolozi Park, South Africa. J S Afr Vet Assoc. 2014; 85(1): e1-e7. |
| 399676 | Uganda | Crockett R, Gilbert A, Kityo R, Ledermann J, Borland E, Powers A, Panella N, Crabtree M, Nakayiki T, Kuzmin I, Niezgoda M, Agwanda B, Markotter W, Malekani J, Kalemba L, Akaibe D, Rupprecht C, Lutwama J, Miller B. Arbovirus surveillance and virus isolations from bats in Uganda, Kenya, and the Democratic Republic of the Congo. Am J Trop Med Hyg. 2012; 87(5 Suppl. 1): 170. |
| 399674 | South Africa | Mapaco LP, Coetzer JA, Paweska JT, Venter EH. An investigation into an outbreak of Rift Valley fever on a cattle farm in Bela-Bela, South Africa, in 2008. J S Afr Vet Assoc. 2012; 83(1): 132. |
| 399672 | Mozambique | Fafetine J, Neves L, Thompson PN, Paweska JT, Rutten VP, Coetzer JA. Serological evidence of Rift Valley fever virus circulation in sheep and goats in Zambézia Province, Mozambique. PLoS Negl Trop Dis. 2013; 7(2): e2065. |
| 399670 | South Sudan, Sudan | Aradaib IE, Erickson BR, Elageb RM, Khristova ML, Carroll SA, Elkhidir IM, Karsany ME, Karrar AE, Elbashir MI, Nichol ST. Rift Valley fever, Sudan, 2007 and 2010. Emerg Infect Dis. 2013; 19(2): 246-53. |
| 399668 | Kenya | La Beaud AD, Banda T, Teng CY, Pfeil S, Muchiri EM, Mungai P, Mutuku FM, Bustinduy AL, Gildengorin G, Kitron U, King CH. Rift valley fever seroprevalence in coastal Kenya [poster presentation]. Am J Trop Med Hyg. 2013; 89(5 Suppl. 1): 136. |
| 399647 | Kenya | Britch SC, Binepal YS, Ruder MG, Kariithi HM, Linthicum KJ, Anyamba A, Small JL, Tucker CJ, Ateya LO, Oriko AA, Gacheru S, Wilson WC. Rift Valley fever risk map model and seroprevalence in selected wild ungulates and camels from Kenya. PLoS One. 2013; 8(6): e66626. |
| 399645 | Uganda | Magona JW, Galiwango T, Walubengo J, Mukiibi G. Rift Valley fever in Uganda: Seroprevalence and risk factor surveillance vis-à-vis mosquito vectors, anti-RVF virus IgG and RVF virus neutralizing antibodies in goats. Small Ruminant Research. 2013; 114(1): 17681. |
| 399421 | Namibia | Monaco F, Pinoni C, Cosseddu GM, Khaiseb S, Calistri P, Molini U, Bishi A, Conte A, Scacchia M, Lelli R. Rift Valley fever in Namibia, 2010. Emerg Infect Dis. 2013; 19(12): 2025-7. |
| 399419 | Saudi Arabia | Lernout T, Cardinale E, Jego M, Desprès P, Collet L, Zumbo B, Tillard E, Girard S, Filleul L. Rift valley fever in humans and animals in Mayotte, an endemic situation?. PLoS One. 2013; 8(9): e74192. |
| 399417 | Saudi Arabia | Al Azraqi TA, El Mekki AA, Mahfouz AA. Rift Valley fever among children and adolescents in southwestern Saudi Arabia. J Infect Public Health. 2013; 6(3): 230-5. |
| 399415 | Zimbabwe | Caron A, Miguel E, Gomo C, Makaya P, Pfukenyi DM, Foggin C, Hove T, de Garine-Wichatitsky M. Relationship between burden of infection in ungulate populations and wildlife/livestock interfaces. Epidemiol Infect. 2013; 141(7): 1522-35. |
| 399413 | Kenya | Newman-Gerhardt S, Muiruri S, Muchiri E, Peters CJ, Morrill J, Lucas AH, King CH, Kazura J, LaBeaud AD. Potential for autoimmune pathogenesis of Rift Valley Fever virus retinitis. Am J Trop Med Hyg. 2013; 89(3): 495-7. |
| 399411 | Mauritania | Jäckel S, Eiden M, El Mamy BO, Isselmou K, Vina-Rodriguez A, Doumbia B, Groschup MH. Molecular and serological studies on the Rift Valley fever outbreak in Mauritania in 2010. Transbound Emerg Dis. 2013; 60 Suppl 2: 31-9. |
| 399409 | Tanzania | Sumaye RD, Geubbels E, Mbeyela E, Berkvens D. Inter-epidemic transmission of Rift Valley fever in livestock in the Kilombero River Valley, Tanzania: a cross-sectional survey. PLoS Negl Trop Dis. 2013; 7(8): e2356. |
| 399407 | South Africa | Ellis CE, Mareledwane VE, Williams R, Wallace DB, Majiwa PA. Validation of an ELISA for the concurrent detection of total antibodies (IgM and IgG) to Rift Valley fever virus. Onderstepoort J Vet Res. 2014; 81(1). |
| 399405 | Mozambique | Lagerqvist N, Moiane B, Mapaco L, Fafetine J, Vene S, Falk KI. Antibodies against Rift Valley fever virus in cattle, Mozambique. Emerg Infect Dis. 2013; 19(7): 1177-9. |
| 399403 | Saudi Arabia | Al-Hamdan NA, Panackal AA, Al Bassam TH, Alrabea A, Al Hazmi M, Al Mazroa Y, Al Jefri M, Khan AS, Ksiazek TG. The Risk of Nosocomial Transmission of Rift Valley Fever. PLoS Negl Trop Dis. 2015; 9(12): e0004314. |
| 399401 | Kenya | Lwande OW, Paul GO, Chiyo PI, Ng'ang'a E, Otieno V, Obanda V, Evander M. Spatio-temporal variation in prevalence of Rift Valley fever: a post-epidemic serum survey in cattle and wildlife in Kenya. Infect Ecol Epidemiol. 2015; 5: 30106. |
| 399399 | Saudi Arabia | Boshra H, Truong T, Babiuk S, Hemida MG. Seroprevalence of Sheep and Goat Pox, Peste Des Petits Ruminants and Rift Valley Fever in Saudi Arabia. PLoS One. 2015; 10(10): e0140328. |
| 399397 | Tanzania | Swai ES, Sindato C. Seroprevalence of Rift Valley fever virus infection in camels (dromedaries) in northern Tanzania. Trop Anim Health Prod. 2015; 47(2): 347-52. |
| 399248 | Egypt | Selim A, Kamel I, Ibrahim E-SM. Seroprevalence and economic impact of rift valley fever among small ruminants. Asian Journal of Animal and Veterinary Advances. 2015; 10(11): 781-8. |
| 399246 | Botswana | Jori F, Alexander KA, Mokopasetso M, Munstermann S, Moagabo K, Paweska JT. Serological Evidence of Rift Valley Fever Virus Circulation in Domestic Cattle and African Buffalo in Northern Botswana (2010-2011). Front Vet Sci. 2015; 2: 63. |
| 399244 | Kenya | Tigoi C, Lwande O, Orindi B, Irura Z, Ongus J, Sang R. Seroepidemiology of selected arboviruses in febrile patients visiting selected health facilities in the lake/river basin areas of Lake Baringo, Lake Naivasha, and Tana River, Kenya. Vector Borne Zoonotic Dis. 2015; 15(2): 124-32. |
| 399242 | Madagascar | Gray GC, Anderson BD, LaBeaud AD, Heraud JM, Fèvre EM, Andriamandimby SF, Cook EAJ, Dahir S, de Glanville WA, Heil GL, Khan SU, Muiruri S, Olive MM, Thomas LF, Merrill HR, Merrill MLM, Richt JA. Seroepidemiological Study of Interepidemic Rift Valley Fever Virus Infection Among Persons with Intense Ruminant Exposure in Madagascar and Kenya. Am J Trop Med Hyg. 2015; 93(6): 1364-1370. |
| 399240 | Senegal | Lo MM, Mbao V, Sierra P, Thiongane Y, Diop M, Donadeu M, Dungu B. Safety and immunogenicity of Onderstepoort Biological Products' Rift Valley fever Clone 13 vaccine in sheep and goats under field conditions in Senegal. Onderstepoort J Vet Res. 2015; 82(1): 857. |
| 399231 | Tanzania | Heinrich N, Mangu CD, Ntinginya NE, Dobler G, Saathoff E, Maboko LL, Hoelscher M. Rift Valley fever, Chikungunya and rickettsioses: Synopsis of four seroprevalence studies on arthropod-borne diseases from South-Western Tanzania on geographic distribution and risk factors. Trop Med Int Health. 2015; 20(51): 162. |
| 399229 | Mauritania | Boushab BM, Savadogo M, Sow SM, Soufiane S. Survey of investigation around cases of Rift Valley Fever at Tagant, Mauritania. Rev Epidemiol Sante Publique. 2015; 63(3): 213-6. |
| 399224 | Kenya | Hise AG, Traylor Z, Hall NB, Sutherland LJ, Dahir S, Ermler ME, Muiruri S, Muchiri EM, Kazura JW, LaBeaud AD, King CH, Stein CM. Association of symptoms and severity of rift valley fever with genetic polymorphisms in human innate immune pathways. PLoS Negl Trop Dis. 2015; 9(3): e0003584. |
| 399222 | China | Liu J, Sun Y, Shi W, Tan S, Pan Y, Cui S, Zhang Q, Dou X, Lv Y, Li X, Li X, Chen L, Quan C, Wang Q, Zhao Y, Lv Q, Hua W, Zeng H, Chen Z, Xiong H, Jiang C, Pang X, Zhang F, Liang M, Wu G, Gao GF, Liu WJ, Li A, Wang Q. The first imported case of Rift Valley fever in China reveals a genetic reassortment of different viral lineages. Emerg Microbes Infect. 2017; 6(1): e4. |
| 399218 | Côte d’Ivoire | Kanouté YB, Gragnon BG, Schindler C, Bonfoh B, Schelling E. Neglected zoonoses at the human and livestock interface in Northern Côte d’Ivoire. Trop Med Int Health. 2015; 20(Suppl. 1): 108. |
| 399215 | Saudi Arabia | Memish ZA, Masri MA, Anderson BD, Heil GL, Merrill HR, Khan SU, Alsahly A, Gray GC. Elevated antibodies against Rift Valley fever virus among humans with exposure to ruminants in Saudi Arabia. Am J Trop Med Hyg. 2015; 92(4): 739-43. |
| 399212 | Madagascar | Olive MM, Grosbois V, Tran A, Nomenjanahary LA, Rakotoarinoro M, Andriamandimby SF, Rogier C, Heraud JM, Chevalier V. Reconstruction of Rift Valley fever transmission dynamics in Madagascar: estimation of force of infection from seroprevalence surveys using Bayesian modelling. Sci Rep. 2017; 7: 39870. |
| 399210 | Mozambique | Moiane B, Mapaco L, Thompson P, Berg M, Albihn A, Fafetine J. High seroprevalence of Rift Valley fever phlebovirus in domestic ruminants and African Buffaloes in Mozambique shows need for intensified surveillance. Infect Ecol Epidemiol. 2017; 7(1): 1416248. |
| 399204 | Senegal | Ba Y, Sall AA, Diallo D, Mondo M, Girault L, Dia I, Diallo M. Re-emergence of Rift Valley fever virus in Barkedji (Senegal, West Africa) in 2002-2003: identification of new vectors and epidemiological implications. J Am Mosq Control Assoc. 2012; 28(3): 170-8. |
| 399195 | Mayotte | Cêtre-Sossah C, Pédarrieu A, Guis H, Defernez C, Bouloy M, Favre J, Girard S, Cardinale E, Albina E. Prevalence of Rift Valley Fever among ruminants, Mayotte. Emerg Infect Dis. 2012; 18(6): 972-5. |
| 399192 | Mauritania, Senegal | Soumaré PO, Freire CC, Faye O, Diallo M, de Oliveira JV, Zanotto PM, Sall AA. Phylogeography of Rift Valley Fever virus in Africa reveals multiple introductions in Senegal and Mauritania. PLoS One. 2012; 7(4): e35216. |
| 399064 | Cameroon | Sadeuh-Mba SA, Yonga Wansi GM, Demanou M, Gessain A, Njouom R. Serological evidence of rift valley fever Phlebovirus and Crimean-Congo hemorrhagic fever orthonairovirus infections among pygmies in the east region of Cameroon. Virol J. 2018; 15(1): 63. |
| 399062 | Uganda | Sternberg Lewerin S, Wolff C, Masembe C, Ståhl K, Boqvist S, Franko MA. Methodological aspects of serosurveillance in resource-poor settings. Vet Rec Open. 2018; 5(1): e000273. |
| 399060 | Sierra Leone | O’Hearn AE, Voorhees MA, Fetterer DP, Wauquier N, Coomber MR, Bangura J, Fair JN, Gonzalez JP, Schoepp RJ. Serosurveillance of viral pathogens circulating in West Africa. Virol J. 2016; 13(1): 163. |
| 399058 | Senegal | Thiongane Y, Thonnon J, Zeller H, Lo MM, Faty A, Diagne F, Gonzalez J, Akakpo JA, Fontenille D, Digoutte JP. Recent data on Rift Valley Fever epidemiology in Senegal. Dakar Med. 1996; Spec No: 1-6. |
| 399056 | Senegal | Sow A, Faye O, Ba Y, Diallo D, Fall G, Faye O, Bob NS, Loucoubar C, Richard V, Dia AT, Diallo M, Malvy D, Sall AA. Widespread Rift Valley Fever Emergence in Senegal in 2013-2014. Open Forum Infect Dis. 2016; 3(3): ofw149. |
| 399054 | Saudi Arabia, Somalia, Sudan | Mohamed AM, Ashshi AM, Asghar AH, Abd El-Rahim IH, El-Shemi AG, Zafar T. Seroepidemiological survey on Rift Valley fever among small ruminants and their close human contacts in Makkah, Saudi Arabia, in 2011. Rev Sci Tech. 2014; 33(3): 903-15. |
| 399052 | Sudan | Elhassan TMA, Mansour MEA, Ibrahim NAM, Elhussein AM. Risk factors of Rift Valley fever in central Sudan. Int J Infect Dis. 2014; 21(Suppl 1): 23. |
| 399050 | Mauritania | Sow A, Faye O, Ba Y, Ba H, Diallo D, Faye O, Loucoubar C, Boushab M, Barry Y, Diallo M, Sall AA. Rift Valley fever outbreak, southern Mauritania, 2012. Emerg Infect Dis. 2014; 20(2): 296-9. |
| 399048 | Senegal | Sow A, Faye O, Faye O, Diallo D, Sadio BD, Weaver SC, Diallo M, Sall AA. Rift Valley fever in Kedougou, southeastern Senegal, 2012. Emerg Infect Dis. 2014; 20(3): 504-6. |
| 399046 | Mozambique | Lesko B, Gudo ES, Vene S, Candido SI, Deus ND, Pinto F, Pinto G, Manhica I, Falk KI. Sero-epidemiological findings of zoonotic infections in Maputo suburban residents. Int J Infect Dis. 2014; 21: 2312. |
| 399044 | Mauritania | Faye O, Ba H, Ba Y, Freire CC, Faye O, Ndiaye O, Elgady IO, Zanotto PM, Diallo M, Sall AA. Reemergence of Rift Valley fever, Mauritania, 2010. Emerg Infect Dis. 2014; 20(2): 300-3. |
| 399042 | Burkina Faso | Boussini H, Lamien CE, Nacoulma OG, Kaboré A, Poda G, Viljoen G. Prevalence of Rift Valley fever in domestic ruminants in the central and northern regions of Burkina Faso. Rev Sci Tech. 2014; 33(3): 893-901. |
| 399010 | Kenya | Owange NO, Ogara WO, Affognon H, Peter GB, Kasiiti J, Okuthe S, Onyango-Ouma W, Landmann T, Sang R, Mbabu M. Occurrence of rift valley fever in cattle in Ijara district, Kenya. Prev Vet Med. 2014; 117(1): 121-8. |
| 399008 | Mozambique | Gudo ES, Pinto G, Weyer J, le Roux C, Mandlaze A, José AF, Muianga A, Paweska JT. Serological evidence of rift valley fever virus among acute febrile patients in Southern Mozambique during and after the 2013 heavy rainfall and flooding: implication for the management of febrile illness. Virol J. 2016; 13: 96. |
| 399006 | Nigeria | Bukbuk DN, Fukushi S, Tani H, Yoshikawa T, Taniguchi S, Iha K, Fukuma A, Shimojima M, Morikawa S, Saijo M, Kasolo F, Baba SS. Development and validation of serological assays for viral hemorrhagic fevers and determination of the prevalence of Rift Valley fever in Borno State, Nigeria. Trans R Soc Trop Med Hyg. 2014; 108(12): 768-73. |
| 399000 | Sudan | Baudin M, Jumaa AM, Jomma HJE, Karsany MS, Bucht G, Näslund J, Ahlm C, Evander M, Mohamed N. Association of Rift Valley fever virus infection with miscarriage in Sudanese women: a cross-sectional study. Lancet Glob Health. 2016; 4(11): e864-e871. |
| 398998 | Djibouti | Andayi F, Charrel RN, Kieffer A, Richet H, Pastorino B, Leparc-Goffart I, Ahmed AA, Carrat F, Flahault A, de Lamballerie X. A sero-epidemiological study of arboviral fevers in Djibouti, Horn of Africa. PLoS Negl Trop Dis. 2014; 8(12): e3299. |
| 398995 | Madagascar | Rakotoarivelo RA, Andrianasolo R, Razafimahefa SH, Randremandranto Razafimbelo NS, Randria MJ. Severe presentations of Rift Valley Fever in Madagascar. Med Mal Infect. 2011; 41(6): 318-21. |
| 398782 | Kenya | Lutomiah J, Omondi D, Masiga D, Mutai C, Mireji PO, Ongus J, Linthicum KJ, Sang R. Blood meal analysis and virus detection in blood-fed mosquitoes collected during the 2006-2007 Rift Valley fever outbreak in Kenya. Vector Borne Zoonotic Dis. 2014; 14(9): 656-64. |
| 398780 | Mauritania | Boushab MB, Savadogo M, Sow MS, Fall-Malick FZ, Seydi M. Severe hemorrhagic form of Rift Valley Fever in Mauritania. Bull Soc Pathol Exot. 2015; 108(2): 102-6. |
| 398778 | Tanzania | Sumaye RD, Abatih EN, Thiry E, Amuri M, Berkvens D, Geubbels E. Inter-epidemic acquisition of Rift Valley fever virus in humans in Tanzania. PLoS Negl Trop Dis. 2015; 9(2): e0003536. |
| 398774 | Kenya | LaBeaud AD, Pfeil S, Muiruri S, Dahir S, Sutherland LJ, Traylor Z, Gildengorin G, Muchiri EM, Morrill J, Peters CJ, Hise AG, Kazura JW, King CH. Factors associated with severe human Rift Valley fever in Sangailu, Garissa County, Kenya. PLoS Negl Trop Dis. 2015; 9(3): e0003548. |
| 398749 | Kenya | Muiruri S, Kabiru EW, Muchiri EM, Hussein H, Kagondu F, LaBeaud AD, King CH. Cross-sectional survey of Rift Valley fever virus exposure in Bodhei village located in a transitional coastal forest habitat in Lamu county, Kenya. Am J Trop Med Hyg. 2015; 92(2): 394-400. |
| 398745 | Iran | Fakour S, Naserabadi S, Ahmadi E. The first positive serological study on rift valley fever in ruminants of Iran. J Vector Borne Dis. 2017; 54(4): 348-352. |
| 398743 | Mauritania | Rissmann M, Eiden M, El Mamy BO, Isselmou K, Doumbia B, Ziegler U, Homeier-Bachmann T, Yahya B, Groschup MH. Serological and genomic evidence of Rift Valley fever virus during inter-epidemic periods in Mauritania. Epidemiol Infect. 2017; 145(5): 1058-1068. |
| 398741 | Saudi Arabia | Mohamed RAEH, Mohamed N, Aleanizy FS, Alqahtani FY, Khalaf AA, Al-Keridis LA. Investigation of hemorrhagic fever viruses inside wild populations of ticks: One of the pioneer studies in Saudi Arabia. Asian Pac J Trop Dis. 2017; 7(5): 299303. |
| 398737 | South Africa | Beechler BR, Bengis R, Swanepoel R, Paweska JT, Kemp A, van Vuren PJ, Joubert J, Ezenwa VO, Jolles AE. Rift valley Fever in Kruger national park: do buffalo play a role in the inter-epidemic circulation of virus?. Transbound Emerg Dis. 2015; 62(1): 24-32. |
| 398575 | Kenya | Cook EAJ, Grossi-Soyster EN, de Glanville WA, Thomas LF, Kariuki S, Bronsvoort BMC, Wamae CN, LaBeaud AD, Fèvre EM. The sero-epidemiology of Rift Valley fever in people in the Lake Victoria Basin of western Kenya. PLoS Negl Trop Dis. 2017; 11(7): e0005731. |
| 398573 | Saudi Arabia | Shraim MA, Eid R, Radad K, Saeed N. Ultrastructural pathology of human liver in Rift Valley fever. BMJ Case Rep. 2016; 2016. |
| 398571 | Angola, China | Wiwanitkit V. Emerging Rift Valley fever in China: What should be known?. Asian Pac J Trop Biomed. 2016; 6(9): 727-9. |
| 398565 | Namibia | Capobianco Dondona A, Aschenborn O, Pinoni C, Di Gialleonardo L, Maseke A, Bortone G, Polci A, Scacchia M, Molini U, Monaco F. Rift Valley Fever Virus among Wild Ruminants, Etosha National Park, Namibia, 2011. Emerg Infect Dis. 2016; 22(1): 128-30. |
| 398063 | Tanzania | Heinrich N, Saathoff E, Weller N, Clowes P, Kroidl I, Ntinginya E, Machibya H, Maboko L, Löscher T, Dobler G, Hoelscher M. High seroprevalence of Rift Valley FEVER AND EVIDENCE FOR ENDEMIC circulation in Mbeya region, Tanzania, in a cross-sectional study. PLoS Negl Trop Dis. 2012; 6(3): e1557. |
| 398061 | Tanzania | Ahmed A, Makame J, Robert F, Julius K, Mecky M. Sero-prevalence and spatial distribution of Rift Valley fever infection among agro-pastoral and pastoral communities during Interepidemic period in the Serengeti ecosystem, northern Tanzania. BMC Infect Dis. 2018; 18(1): 276. |
| 398059 | Uganda | Nyakarahuka L, de St Maurice A, Purpura L, Ervin E, Balinandi S, Tumusiime A, Kyondo J, Mulei S, Tusiime P, Lutwama J, Klena JD, Brown S, Knust B, Rollin PE, Nichol ST, Shoemaker TR. Prevalence and risk factors of Rift Valley fever in humans and animals from Kabale district in Southwestern Uganda, 2016. PLoS Negl Trop Dis. 2018; 12(5): e0006412. |
| 398057 | Uganda | Kading RC, Kityo RM, Mossel EC, Borland EM, Nakayiki T, Nalikka B, Nyakarahuka L, Ledermann JP, Panella NA, Gilbert AT, Crabtree MB, Peterhans JK, Towner JS, Amman BR, Sealy TK, Nichol ST, Powers AM, Lutwama JJ, Miller BR. Neutralizing antibodies against flaviviruses, Babanki virus, and Rift Valley fever virus in Ugandan bats. Infect Ecol Epidemiol. 2018; 8(1): 1439215. |
| 398055 | Zambia | Saasa N, Kajihara M, Dautu G, Mori-Kajihara A, Fukushi S, Sinkala Y, Morikawa S, Mweene A, Takada A, Yoshimatsu K, Arikawa J. Expression of a Recombinant Nucleocapsid Protein of Rift Valley Fever Virus in Vero Cells as an Immunofluorescence Antigen and Its Use for Serosurveillance in Traditional Cattle Herds in Zambia. Vector Borne Zoonotic Dis. 2018; 18(5): 273-277. |
| 398051 | Algeria, Western Sahara | Di Nardo A, Rossi D, Saleh SM, Lejlifa SM, Hamdi SJ, Di Gennaro A, Savini G, Thrusfield MV. Evidence of Rift Valley fever seroprevalence in the Sahrawi semi-nomadic pastoralist system, Western Sahara. BMC Vet Res. 2014; 10: 92. |
| 398048 | Comoros | Roger M, Beral M, Licciardi S, Soulé M, Faharoudine A, Foray C, Olive MM, Maquart M, Soulaimane A, Madi Kassim A, Cêtre-Sossah C, Cardinale E. Evidence for circulation of the rift valley fever virus among livestock in the union of Comoros. PLoS Negl Trop Dis. 2014; 8(7): e3045. |
| 398039 | Tanzania | Kifaro EG, Nkangaga J, Joshua G, Sallu R, Yongolo M, Dautu G, Kasanga CJ. Epidemiological study of Rift Valley fever virus in Kigoma, Tanzania. Onderstepoort J Vet Res. 2014; 81(2): E1-5. |
| 398037 | Djibouti | Abbas B, Yousif MA, Nur HM. Animal health constraints to livestock exports from the Horn of Africa. Rev Sci Tech. 2014; 33(3): 711-21. |
| 397478 | Kenya | Lichoti JK, Kihara A, Oriko AA, Okutoyi LA, Wauna JO, Tchouassi DP, Tigoi CC, Kemp S, Sang R, Mbabu RM. Detection of rift valley Fever virus interepidemic activity in some hotspot areas of kenya by sentinel animal surveillance, 2009-2012. Vet Med Int. 2014; 2014: 379010. |
| 397475 | Kenya | Sang R, Kioko E, Lutomiah J, Warigia M, Ochieng C, O'Guinn M, Lee JS, Koka H, Godsey M, Hoel D, Hanafi H, Miller B, Schnabel D, Breiman RF, Richardson J. Rift Valley fever virus epidemic in Kenya, 2006/2007: the entomologic investigations. Am J Trop Med Hyg. 2010; 83(2 Suppl): 28-37. |
| 397473 | Madagascar | Ratovonjato J, Olive MM, Tantely LM, Andrianaivolambo L, Tata E, Razainirina J, Jeanmaire E, Reynes JM, Elissa N. Detection, isolation, and genetic characterization of Rift Valley fever virus from Anopheles (Anopheles) coustani, Anopheles (Anopheles) squamosus, and Culex (Culex) antennatus of the Haute Matsiatra region, Madagascar. Vector Borne Zoonotic Dis. 2011; 11(6): 753-9. |
| 397471 | Kenya, Tanzania | Sabirovic M, Raw L, Rogers A, Hall S, Elliott H, Coulson N. International disease monitoring, January to March 2007. Vet Rec. 2007; 160(21): 717-22. |
| 397457 | Uganda | de St Maurice A, Nyakarahuka L, Purpura L, Ervin E, Tumusiime A, Balinandi S, Kayondo J, Mulei S, Namutebi AM, Tusiime P, Wiersma S, Nichol S, Rollin P, Klena J, Knust B, Shoemaker T. Notes from the Field: Rift Valley Fever Response - Kabale District, Uganda, March 2016. MMWR Morb Mortal Wkly Rep. 2016;65(43):1200-1201 |
| 397058 | Kenya | Mbotha D, Bett B, Kairu-Wanyoike S, Grace D, Kihara A, Wainaina M, Hoppenheit A, Clausen PH, Lindahl J. Inter-epidemic Rift Valley fever virus seroconversions in an irrigation scheme in Bura, south-east Kenya. Transbound Emerg Dis. 2018; 65(1): e55-e62. |
| 397056 | Kenya | Hightower A, Kinkade C, Nguku PM, Anyangu A, Mutonga D, Omolo J, Njenga MK, Feikin DR, Schnabel D, Ombok M, Breiman RF. Relationship of climate, geography, and geology to the incidence of Rift Valley fever in Kenya during the 2006-2007 outbreak. Am J Trop Med Hyg. 2012; 86(2): 373-80. |
| 397054 | Central African Republic | Nakouné E, Kamgang B, Berthet N, Manirakiza A, Kazanji M. Rift Valley Fever Virus Circulating among Ruminants, Mosquitoes and Humans in the Central African Republic. PLoS Negl Trop Dis. 2016; 10(10): e0005082. |
| 397052 | Comoros | Dellagi K, Salez N, Maquart M, Larrieu S, Yssouf A, Silaï R, Leparc-Goffart I, Tortosa P, de Lamballerie X. Serological Evidence of Contrasted Exposure to Arboviral Infections between Islands of the Union of Comoros (Indian Ocean). PLoS Negl Trop Dis. 2016; 10(12): e0004840. |
| 397050 | Tanzania | Chengula AA, Kasanga CJ, Mdegela RH, Sallu R, Yongolo M. Molecular detection of Rift Valley fever virus in serum samples from selected areas of Tanzania. Trop Anim Health Prod. 2014; 46(4): 629-34. |
| 397048 | Madagascar | Nicolas G, Durand B, Rakotoarimanana TT, Lacote S, Chevalier V, Marianneau P. A 3-year serological and virological cattle follow-up in Madagascar highlands suggests a non-classical transmission route of Rift Valley fever virus. Am J Trop Med Hyg. 2014; 90(2): 265-6. |
| 397046 | Mauritania | El Mamy AB, Lo MM, Thiongane Y, Diop M, Isselmou K, Doumbia B, Baba MO, El Arbi AS, Lancelot R, Kane Y, Albina E, Cêtre-Sossah C. Comprehensive phylogenetic reconstructions of Rift Valley fever virus: the 2010 northern Mauritania outbreak in the Camelus dromedarius species. Vector Borne Zoonotic Dis. 2014; 14(12): 856-61. |
| 397044 | South Africa | Archer BN, Thomas J, Weyer J, Cengimbo A, Landoh DE, Jacobs C, Ntuli S, Modise M, Mathonsi M, Mashishi MS, Leman PA, le Roux C, Jansen van Vuren P, Kemp A, Paweska JT, Blumberg L. Epidemiologic Investigations into Outbreaks of Rift Valley Fever in Humans, South Africa, 2008-2011. Emerg Infect Dis. 2013; 19(12). |
| 397042 | Saudi Arabia | Taha HA, Shoman SA, Alhadlag NM. Molecular and serological survey of some haemoprotozoan, rickettsial and viral diseases of small ruminants from Al-Madinah Al Munawarah, KSA. Trop Biomed. 2015; 32(3): 511-23. |
| 397040 | Sudan | Abdallah MM, Adam IA, Abdalla TM, Abdelaziz SA, Ahmed ME, Aradaib IE. A survey of rift valley fever and associated risk factors among the one-humped camel (Camelus dromedaries) in Sudan. Ir Vet J. 2015; 69: 6. |
| 396818 | Tanzania | Wensman JJ, Lindahl J, Wachtmeister N, Torsson E, Gwakisa P, Kasanga C, Misinzo G. A study of Rift Valley fever virus in Morogoro and Arusha regions of Tanzania - serology and farmers' perceptions. Infect Ecol Epidemiol. 2015; 5: 30025. |
| 396815 | Tanzania | Sindato C, Pfeiffer DU, Karimuribo ED, Mboera LE, Rweyemamu MM, Paweska JT. A Spatial Analysis of Rift Valley Fever Virus Seropositivity in Domestic Ruminants in Tanzania. PLoS One. 2015; 10(7): e0131873. |
| 396813 | Turkey | Gür S, Kale M, Erol N, Yapici O, Mamak N, Yavru S. The first serological evidence for Rift Valley fever infection in the camel, goitered gazelle and Anatolian water buffaloes in Turkey. Trop Anim Health Prod. 2017; 49(7): 1531-1535. |
| 396810 | Kenya | Nanyingi MO, Muchemi GM, Thumbi SM, Ade F, Onyango CO, Kiama SG, Bett B. Seroepidemiological Survey of Rift Valley Fever Virus in Ruminants in Garissa, Kenya. Vector Borne Zoonotic Dis. 2017; 17(2): 141-146. |
| 396808 | Egypt | Mroz C, Gwida M, El-Ashker M, El-Diasty M, El-Beskawy M, Ziegler U, Eiden M, Groschup MH. Seroprevalence of Rift Valley fever virus in livestock during inter-epidemic period in Egypt, 2014/15. BMC Vet Res. 2017; 13(1): 87. |
| 396806 | Rwanda | Umuhoza T, Berkvens D, Gafarasi I, Rukelibuga J, Mushonga B, Biryomumaisho S. Seroprevalence of Rift Valley fever in cattle along the Akagera-Nyabarongo rivers, Rwanda. J S Afr Vet Assoc. 2017; 88: e1-e5. |
| 396804 | Gabon | Maganga GD, Abessolo Ndong AL, Mikala Okouyi CS, Makiala Mandanda S, N'Dilimabaka N, Pinto A, Agossou E, Cossic B, Akue JP, Leroy EM. Serological Evidence for the Circulation of Rift Valley Fever Virus in Domestic Small Ruminants in Southern Gabon. Vector Borne Zoonotic Dis. 2017; 17(6): 443-446. |
| 396802 | Côte d’Ivoire | Kanouté YB, Gragnon BG, Schindler C, Bonfoh B, Schelling E. Epidemiology of brucellosis, Q Fever and Rift Valley Fever at the human and livestock interface in northern Côte d’Ivoire. Acta Trop. 2017; 165: 66-75. |
| 396800 | Mauritania | Bob NS, Bâ H, Fall G, Ishagh E, Diallo MY, Sow A, Sembene PM, Faye O, El Kouri B, Sidi ML, Sall AA. Detection of the Northeastern African Rift Valley Fever Virus Lineage During the 2015 Outbreak in Mauritania. Open Forum Infect Dis. 2017; 4(2): ofx087. |
| 396798 | Kenya | Fèvre EM, de Glanville WA, Thomas LF, Cook EAJ, Kariuki S, Wamae CN. An integrated study of human and animal infectious disease in the Lake Victoria crescent small-holder crop-livestock production system, Kenya. BMC Infect Dis. 2017; 17(1): 457. |
| 396012 | Saudi Arabia, Somalia, Sudan | Mohamed AM, Ghazi H, Ashshi AM, Faidah HS, Clinical EIA. Serological survey of Rift valley fever among sacrifice animals in holy mecca during pilgrimage season. Int J Trop Med. 2011; 6(4): 85–89. |
| 396010 | Mauritania | Boushab BM, Fall-Malick FZ, Ould Baba SEW, Ould Salem ML, Belizaire MRD, Ledib H, Ould Baba Ahmed MM, Basco LK, Ba H. Severe Human Illness Caused by Rift Valley Fever Virus in Mauritania, 2015. Open Forum Infect Dis. 2016; 3(4): ofw200. |
| 396008 | Mozambique | Gudo ES, Lesko B, Vene S, Lagervist N, Candido SI, de Deus NR, Pinto FD, Pinto G, Monteiro V, Evaristo VL, Bhatt N, Manhica I, Falk KI. Seroepidemiologic Screening for Zoonotic Viral Infections, Maputo, Mozambique. Emerg Infect Dis. 2016; 22(5): 915-917. |
| 395906 | Mali | Subudhi S, Dakouo M, Sloan A, Stein DR, Grolla A, Jones S, Dibernardo A, Rosenke K, Sas M, Traore A, Lindsay R, Groschup MH, Misra V, Feldmann H, Sogoba N, Safronetz D, Niang M. Seroprevalence of Rift Valley Fever Virus Antibodies in Cattle in Mali, 2005-2014. Am J Trop Med Hyg. 2018; 98(3): 872-874. |
| 395904 | Senegal | Sow A, Loucoubar C, Diallo D, Faye O, Ndiaye Y, Senghor CS, Dia AT, Faye O, Weaver SC, Diallo M, Malvy D, Sall AA. Concurrent malaria and arbovirus infections in Kedougou, southeastern Senegal. Malar J. 2016; 15: 47. |
| 395902 | Mauritania | Salem ML, Baba Sel W, Fall-Malick FZ, Boushab BM, Ghaber SM, Mokhtar A. [Severe hemorrhagic forms of Rift Valley fever: about 5 cases]. Pan Afr Med J. 2016; 24: 73. |
| 395900 | Comoros | Roger M, Girard S, Faharoudine A, Halifa M, Bouloy M, Cetre-Sossah C, Cardinale E. Rift valley fever in ruminants, Republic of Comoros, 2009. Emerg Infect Dis. 2011; 17(7): 1319-20. |
| 395898 | South Africa | Pretorius A, Oelofsen MJ, Smith MS, van der Ryst E. Rift Valley fever virus: a seroepidemiologic study of small terrestrial vertebrates in South Africa. Am J Trop Med Hyg. 1997; 57(6): 693-8. |
| 395896 | Gabon | Pourrut X, Nkoghé D, Souris M, Paupy C, Paweska J, Padilla C, Moussavou G, Leroy EM. Rift Valley fever virus seroprevalence in human rural populations of Gabon. PLoS Negl Trop Dis. 2010; 4(7): e763. |
| 395894 | Kenya | Oyas H, Holmstrom L, Kemunto NP, Muturi M, Mwatondo A, Osoro E, Bitek A, Bett B, Githinji JW, Thumbi SM, Widdowson MA, Munyua PM, Njenga MK. Enhanced surveillance for Rift Valley Fever in livestock during El Niño rains and threat of RVF outbreak, Kenya, 2015-2016. PLoS Negl Trop Dis. 2018; 12(4): e0006353. |
| 395892 | France, Mayotte | Métras R, Cavalerie L, Dommergues L, Mérot P, Edmunds WJ, Keeling MJ, Cêtre-Sossah C, Cardinale E. The Epidemiology of Rift Valley Fever in Mayotte: Insights and Perspectives from 11 Years of Data. PLoS Negl Trop Dis. 2016; 10(6): e0004783. |
| 395890 | Angola, China | Liu W, Sun FJ, Tong YG, Zhang SQ, Cao WC. Rift Valley fever virus imported into China from Angola. Lancet Infect Dis. 2016; 16(11): 1226. |
| 395888 | Mali | Haneche F, Leparc-Goffart I, Simon F, Hentzien M, Martinez-Pourcher V, Caumes E, Maquart M. Rift Valley fever in kidney transplant recipient returning from Mali with viral RNA detected in semen up to four months from symptom onset, France, autumn 2015. Euro Surveill. 2016; 21(18). |
| 395886 | Egypt | Hanafi HA, Fryauff DJ, Saad MD, Soliman AK, Mohareb EW, Medhat I, Zayed AB, Szumlas DE, Earhart KC. Virus isolations and high population density implicate Culex antennatus (Becker) (Diptera: Culicidae) as a vector of Rift Valley Fever virus during an outbreak in the Nile Delta of Egypt. Acta Trop. 2011; 119(2-3): 119-24. |
| 395884 | Angola, China | Fu X, Wang L, Fang B, Ma R, Zheng Y, Huang S, Zhou P, Cao Z, Tian J, Li S, Zhang G. Import of Rift Valley fever to China: a potential new threat?. Virol Sin. 2016; 31(5): 454-6. |
| 395882 | Mozambique | Fafetine JM, Coetzee P, Mubemba B, Nhambirre O, Neves L, Coetzer JA, Venter EH. Rift Valley Fever Outbreak in Livestock, Mozambique, 2014. Emerg Infect Dis. 2016; 22(12): 2165-2167. |
| 395880 | Mauritania | Diallo M, Nabeth P, Ba K, Sall AA, Ba Y, Mondo M, Girault L, Abdalahi MO, Mathiot C. Mosquito vectors of the 1998-1999 outbreak of Rift Valley Fever and other arboviruses (Bagaza, Sanar, Wesselsbron and West Nile) in Mauritania and Senegal. Med Vet Entomol. 2005; 19(2): 119-26. |
| 395878 | Madagascar | Carroll SA, Reynes JM, Khristova ML, Andriamandimby SF, Rollin PE, Nichol ST. Genetic evidence for Rift Valley fever outbreaks in Madagascar resulting from virus introductions from the East African mainland rather than enzootic maintenance. J Virol. 2011; 85(13): 6162-7. |
| 395876 | South Africa | Caron A, Cornelis D, Foggin C, Hofmeyr M, de Garine-Wichatitsky M. African Buffalo Movement and Zoonotic Disease Risk across Transfrontier Conservation Areas, Southern Africa. Emerg Infect Dis. 2016; 22(2): 277-80. |
| 395874 | Tunisia | Bosworth A, Ghabbari T, Dowall S, Varghese A, Fares W, Hewson R, Zhioua E, Chakroun M, Tiouiri H, Ben Jemaa M, Znazen A, Letaief A. Serologic evidence of exposure to Rift Valley fever virus detected in Tunisia. New Microbes New Infect. 2015; 9: 1-7. |
| 395872 | Mozambique | Blomström AL, Scharin I, Stenberg H, Figueiredo J, Nhambirre O, Abilio A, Berg M, Fafetine J. Seroprevalence of Rift Valley fever virus in sheep and goats in Zambézia, Mozambique. Infect Ecol Epidemiol. 2016; 6: 31343. |
| 395870 | Kenya | Anyangu AS, Gould LH, Sharif SK, Nguku PM, Omolo JO, Mutonga D, Rao CY, Lederman ER, Schnabel D, Paweska JT, Katz M, Hightower A, Njenga MK, Feikin DR, Breiman RF. Risk factors for severe Rift Valley fever infection in Kenya, 2007. Am J Trop Med Hyg. 2010; 83(2 Suppl): 14-21. |
| 395868 | Zimbabwe | Anderson EC, Rowe LW. The prevalence of antibody to the viruses of bovine virus diarrhoea, bovine herpes virus 1, rift valley fever, ephemeral fever and bluetongue and to Leptospira sp in free-ranging wildlife in Zimbabwe. Epidemiol Infect. 1998; 121(2): 441-9. |

Appendix Figure 1: All extracted data, literature and EMPRES-i


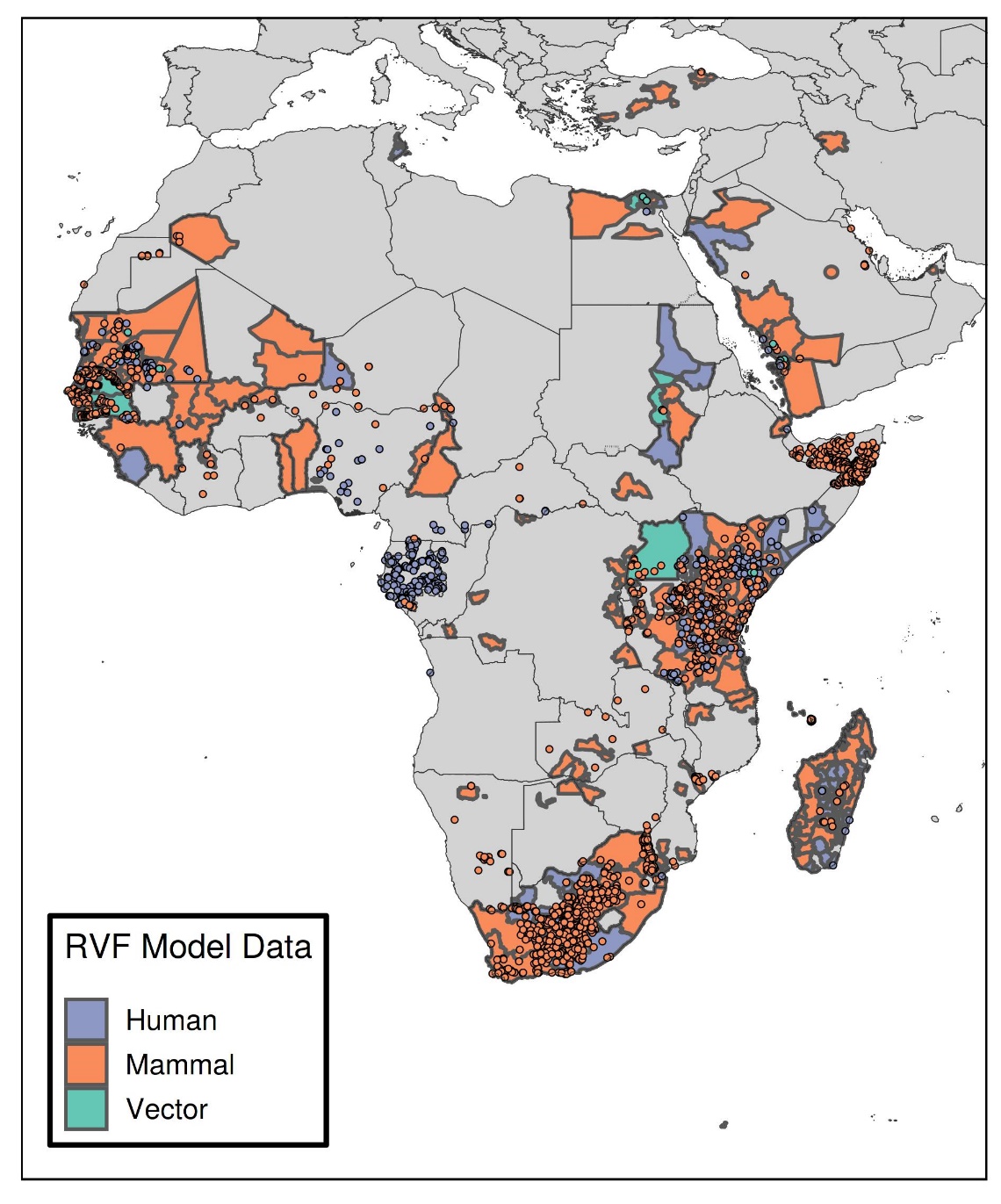


### 2.1.2 Information collected from extraction

For articles we included in our literature review, we extracted a variety of geographical and epidemiological information when available. We noted if the organism tested was a human, mammal, or vector, as well as if the subject was symptomatic when tested. We recorded if the subject had been diagnosed using PCR, a serological test, or neither, in which case the diagnostic method was flagged as “case report.” If the subject was diagnosed using serology, we noted if the serological testing was exploratory or if the intention was to diagnose a symptomatic patient.

### 2.1.3 Point and polygon data

When possible, we extracted geographical information as exact geographical coordinates, or “point data.” Sometimes the paper gave coordinates of where testing occurred, but often, they would show locations of detections as points on a map. When the data were represented this way, we digitised the map by anchoring borders on the map to analogous borders in a digital representation of the Earth in ArcGIS. We could then extract the coordinates to which the point referred.

If a paper referred to testing done in an administrative unit, such as a district or state, we used a digital representation of this unit as “polygon data.” In some instances, we also used custom sampling regions defined in papers as polygon data. In each of our 100 model bootstraps, we sampled a different point from within each polygon to represent that piece of data. Every piece of point data was included in every bootstrap, along with a single point from each polygon. To limit uncertainty introduced by polygon data, we excluded polygons from our analysis from which we couldn’t sample 1% of the area in our bootstraps. Since our model had 100 bootstraps, we only included polygons that had 10,000 pixels or fewer. Each pixel is 25 square kilometers, so the polygons that we excluded had areas greater than 25,000 square kilometers.

### 2.1.4 Date sampling

Sometimes, RVF studies were conducted over a range of dates and did not include the exact date of detections. In these cases, we recorded the range of dates associated with each detection. In each bootstrap of the model, we randomly sampled a month-year combination from this range. Some papers did not report information regarding the month or year of infection. In these cases, we still sampled a date. Of the 1,381 occurrences, nine did not include month information, and of these, three did not include year information. Since RVF occurrences happen in different hemispheres and many different climates, we aimed to sample a month-year combination in a representative way. For each of the detections with no year information, we created a distribution of years when RVF detections had occurred based on our records from the same country that did include date information. In the figure below, we show what this distribution of years looked like for a case in Mauritania with missing information. When a record did not include a month, we repeated a similar process. A monthly distribution for Mauritania is also shown below. If a paper included a year but not a month, or vice versa, we would only sample the information that was missing.

Appendix Figure 2: Example of year sampling distribution


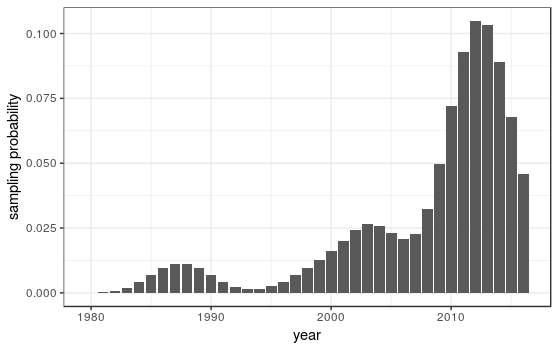


Appendix Figure 3: Example of month sampling distribution


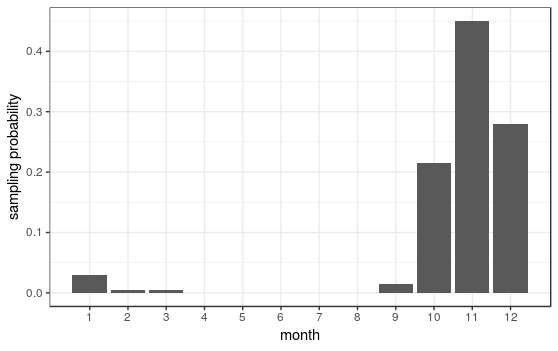


## 2.2 EMPRES-i processing

### 2.2.1 Locality quality processing

We downloaded all RVF occurrence records from the EMPRES-i database on October 3, 2018. Records that were marked with a locality quality of “Exact” and records obtained from the OIE were treated as point data. Records for which the GPS coordinates provided represented the centroid of an administrative unit were treated as a polygon record representing that polygon. Records for which the coordinates represented the centroid of a locality were handled on a case-by-case basis. If the locality was small enough, the record was treated as point data. If not, a custom polygon was made to encompass the region.

### 2.2.2 Date sampling

The EMPRES-i dataset includes the date each case was reported to the FAO. Most records also include a date on which the cases were observed, which was used as our temporal representation of the occurrence when available. When an observation date wasn’t available, which was less than 5% of the time, we made a range from which to sample the date in each bootstrap of our model based on the reporting date. The range was the interquartile range of the times between observation and reporting for all records that did include an observation date.

## 2.3 Deduplication of literature and EMPRES-i data

To remove occurrences that were reported in both the literature and the EMPRES-i database, we conducted a de-duplication process. We only removed data as a duplicate when their date range was either the same as or was totally encompassed by another date range corresponding to a similar geography. To de-duplicate point data across the multiple sources, we rounded latitude and longitude values to the second decimal place. This process helped to identify duplicated cases that might have been reported slightly differently. If the same polygon was reported by multiple sources, we removed it as a duplicate if the date was similar.

## 2.4 Model-specific data subsets

In addition to the modelling dataset shown above, we also ran three other models using different subsets of this data. We ran one model that used only the occurrence records from humans in our initial datasets, a model that only used occurrence records that were diagnosed using PCR, and a model that only used occurrence records that were reported with exact date information. We show the results for these models in Section 5.5 of this appendix.

# 3.0 Modelling framework and techniques

## 3.1 Background data simulation

### 3.1.1 Spatial sampling

We provided the model with disease absence, or background, points in addition to disease presence points. Given the difficulty of establishing disease absence in a geography, we sampled disease absence points from a range close to previous RVF detections. Sampling from this region based on occurrences allowed us to see greater contrast in areas close to previous RVF cases than if we had sampled background points from across the whole world.

To define this region, we combined all point data with the centroids of each piece of polygon data. We used a hierarchical clustering technique to group points into similar geographies. We selected the number of clusters by selecting the number that was greater than or equal to the number of Global Burden of Disease regions (Appendix Figure 4) represented in the data, which yielded the highest gap statistic, as described by Tibshirani and colleagues.^1^ We then found the maximum-minimum pairwise distance between the centroids of these clusters. We buffered the centroid of each cluster by this distance and took the overlapping geography that intersected with land to be our background sampling region. Each bootstrap sampled the same number of background points from this region as the amount of occurrence data provided to the bootstrap.

Appendix Figure 4: Global Burden of Disease (GBD) region definitions


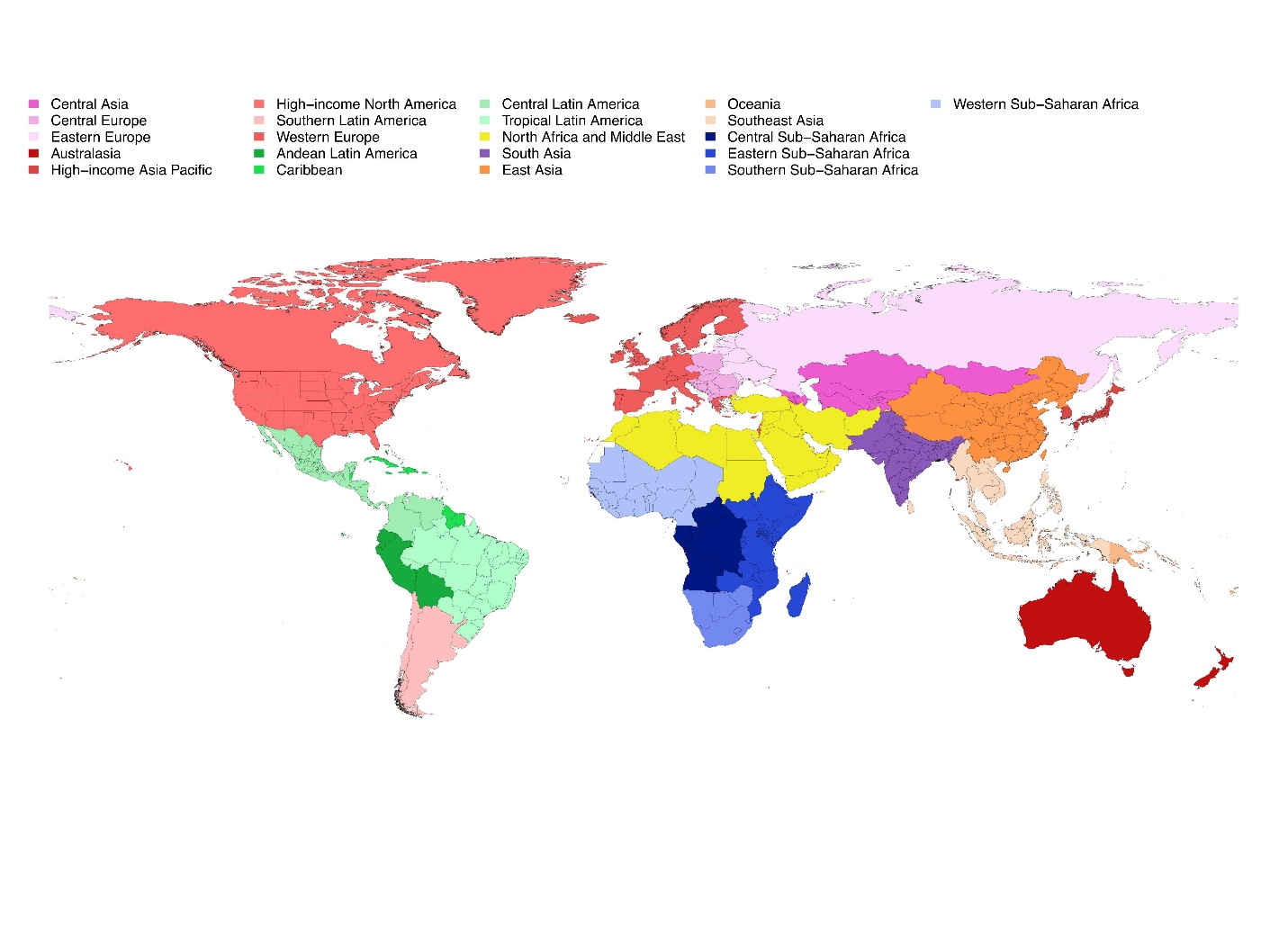


Appendix Figure 5: Datapoints geographically clustered with hierarchical clustering

We computed an optimal number of point clusters and grouped them using hierarchical clustering. The points, grouped into six clusters, are shown below.


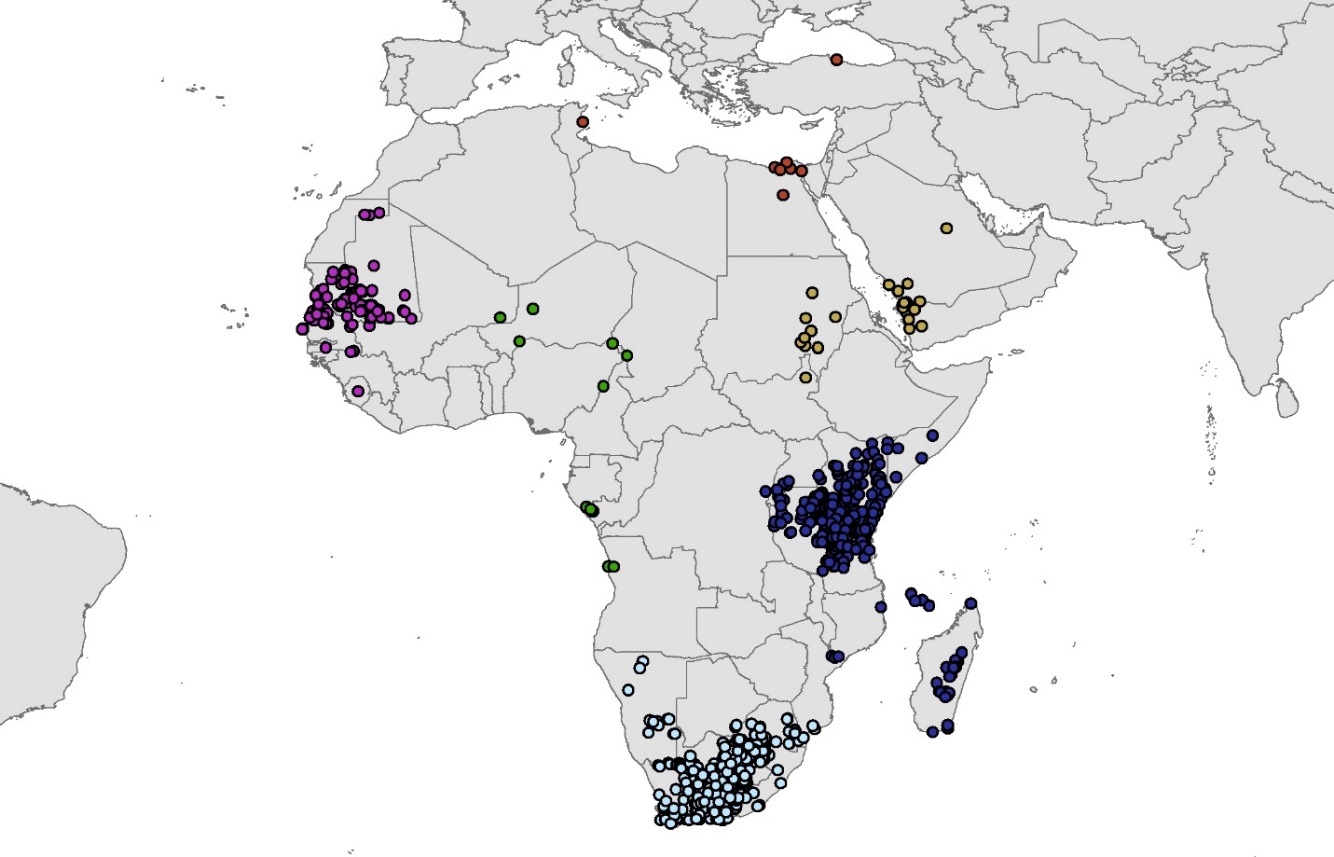


Appendix Figure 6: Buffered cluster centroids

We found the centroid of each cluster of points and buffered each cluster centroid by the maximum-minimum pairwise distance between the cluster centroids.


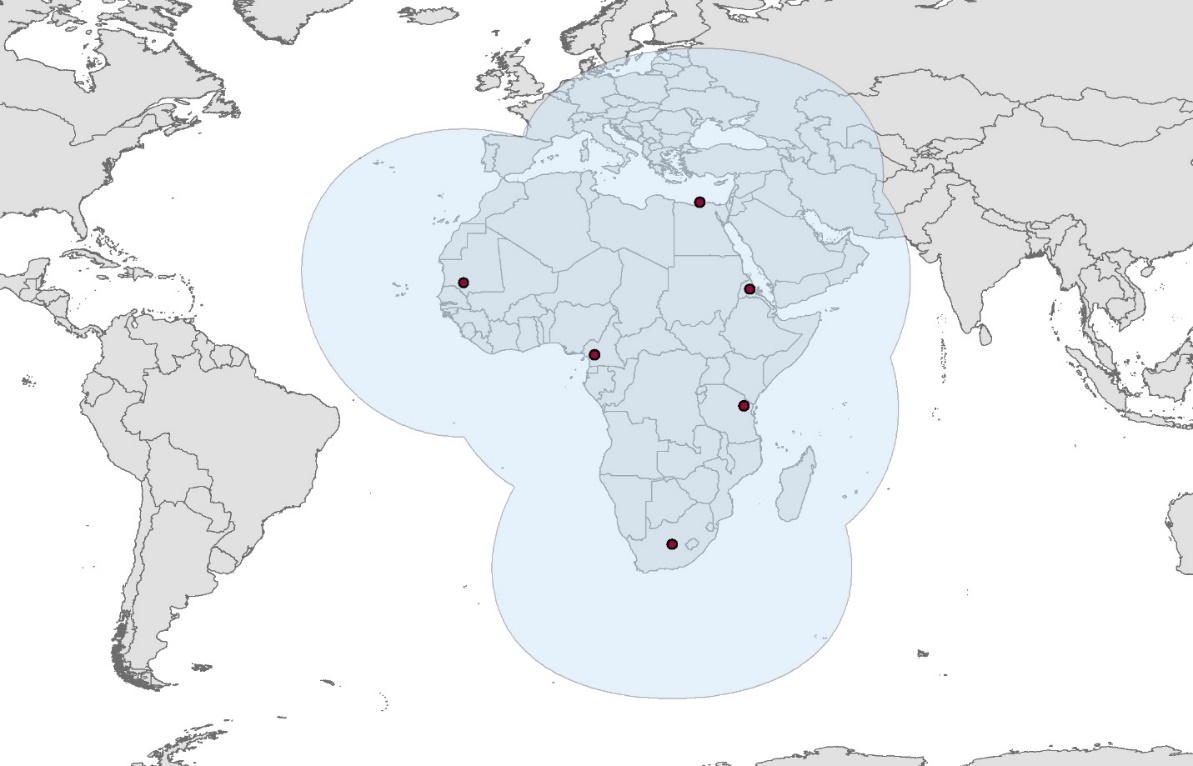


### 3.1.2 Date sampling

To simulate dates of background data, we sampled a year from a distribution representative of all years present in the occurrence data. We then sampled a month from an analogous distribution which used months represented in the occurrence data. We then extracted environmental covariates from this time period in the location that was spatially simulated as described in the section above.

## 3.2 Modelling with boosted regression trees

### 3.2.1 BRT methodology

To model regions environmentally suitable for RVF, we used a species distribution modelling framework. We selected boosted regression trees (BRTs) for this analysis because they have been shown to perform better than other techniques.^2^ BRTs form a sequence of trees that classify a region as suitable or not suitable based on provided covariates. Similar to boosting algorithms, later models are informed by previous trees. Trees are selected that make the best predictions, evaluated by their performance across a test-train split used by the model. An example of one of these trees is shown in Appendix Figure 7. Appendix Figure 8 shows the frequency of interactions between each pair of covariates included in the model.

The modelled relationships are then projected onto covariate values across space and time, regardless of whether occurrences have been reported in those times and places. Predictions are continuous, from 0 to 1, where a prediction of 1 means that covariates in a particular time and location exactly match the region in covariate space considered suitable by the model. Using these predictions, we can determine environmental suitability, which is a key factor in determining the possibility of disease presence in a given place and time. This suitability metric is one important component of determining a location’s risk profile.

Appendix Figure 7: Example of single classification tree

Each component tree of a boosted regression tree model makes a series of binary splits on the values of certain covariates.


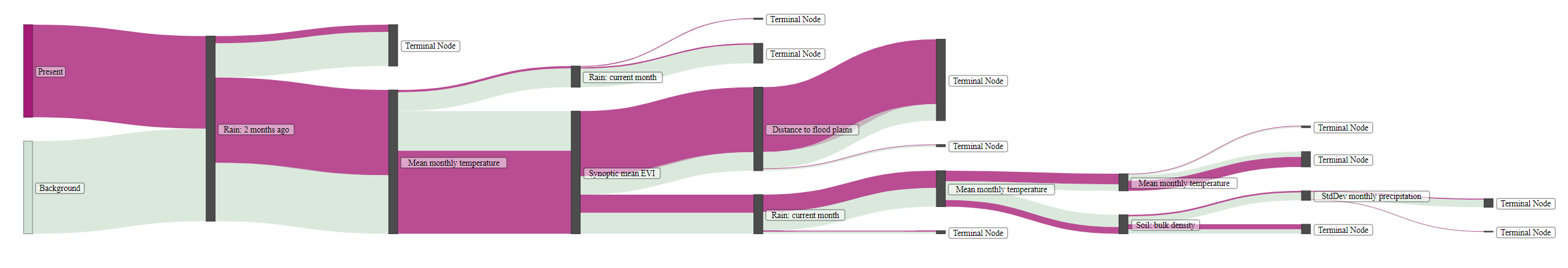


Appendix Figure 8: Frequency of interactions between covariates in classification trees

This plot shows, across all trees, the frequency at which a split on one covariate preceded a split on another covariate.


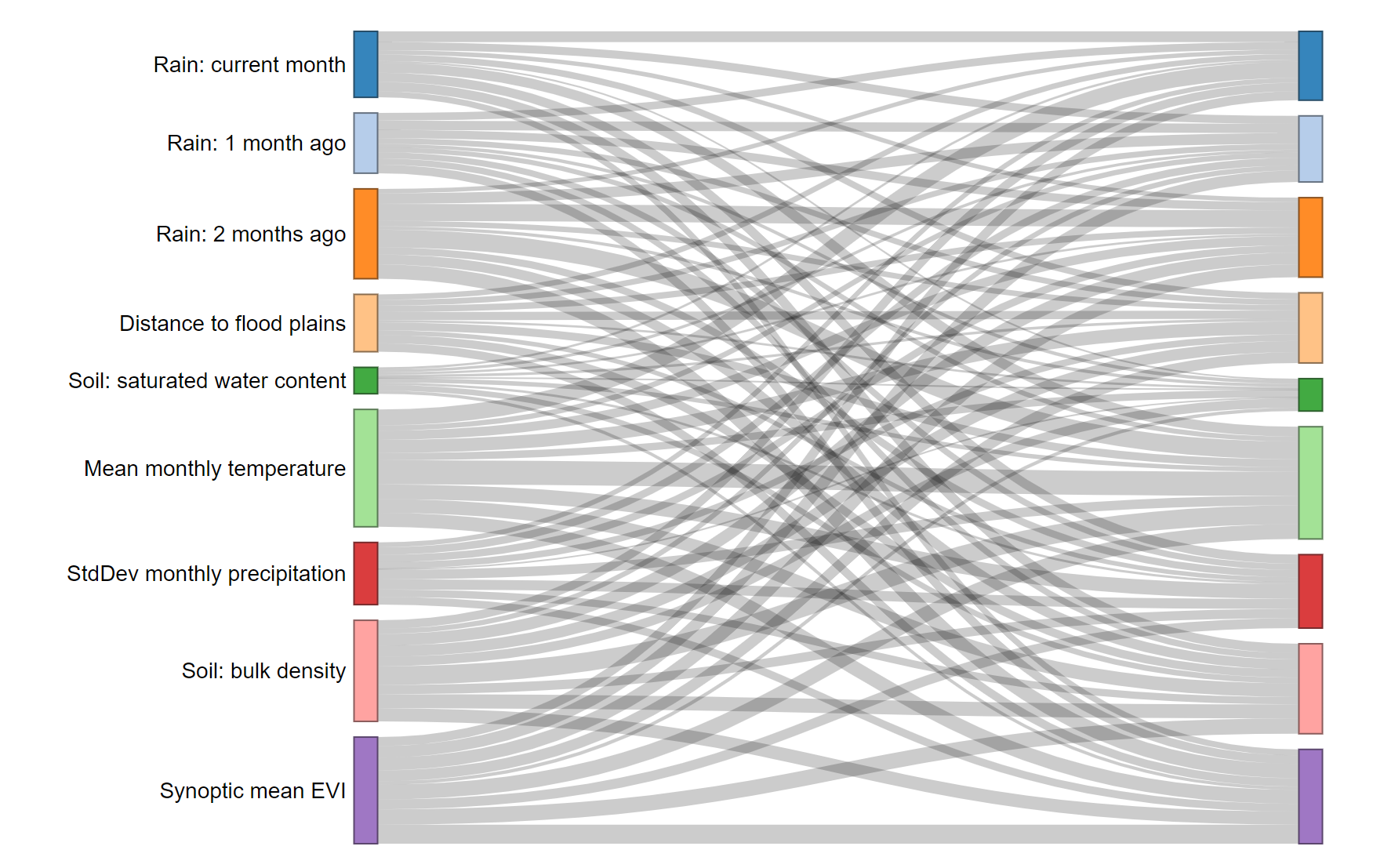


### 3.2.2 Tuning BRT hyper-parameters

BRT models have parameters that affect model behavior, namely the total number of trees used and the complexity and learning rate of these trees.^2,3^ Different combinations of these parameters must be tested to determine the set that leads to optimal model performance. We used Bayesian optimisation, implemented with Scikit-Optimize, to do this testing. With this method, we used an acquisition function to sequentially update the posterior distribution over the parameter space as we sampled different parameters. For each bootstrap, we would pick these parameters using 80% of the model data before running the model with the full dataset.

Appendix Table 3: Model hyperparameters

| **Experiment** | **Median (IQR) Interaction Depth** | **Median (IQR) Minimum observations in node** | **Median (IQR) number of trees** | **Median (IQR) Shrinkage** |
| --- | --- | --- | --- | --- |
| Full dataset | 10 (10 – 10) | 8 (6 – 9) | 2381 (1473 – 3049) | 0.011 (0.007 - 0.017) |
| Human-only dataset (Appendix Section 5.5.1) | 10 (8 – 10) | 8 (7 – 9) | 1344 (550.2 - 2421) | 0.021 (0.011 - 0.037) |
| PCR-only dataset (Appendix Section 5.5.2) | 9 (8 – 10) | 8 (7 –9) | 1443.5 (742.2 - 2556) | 0.020 (0.010 - 0.043) |
| Exact-date-only dataset (Appendix Section 5.5.3) | 10 (8 – 10) | 8 (7 – 9) | 2120 (1139 – 2916) | 0.009 (0.006 - 0.015) |

### 3.2.3 Model covariates

A distinguishing feature of RVF environmental epidemiology across the many climates where it has occurred is its tendency for occurrence when places have standing water, often after prolonged periods of heavy rainfall.^4,5^ This excess water can induce the hatching of transovarially infected mosquitoes and serve as a habitat for new generations and other species of mosquitoes that can become infected after biting hosts, continuing a cycle of transmission. The covariates for this model were selected to identify areas in space and time that are susceptible to flooding events and that could serve as suitable mosquito habitats.

To identify flood-susceptible regions in space and time, we used a suite of environmental covariates (Appendix Table 4). Using the GFPLAINS dataset, we created a distance to nearest floodplain covariate.^6^ For each month of data, we recorded the mean rainfall of that month as well as each of the previous two months to account for the prolonged rainfall that has preceded past RVF outbreaks.^5,7–9^ As others have reported, RVF outbreaks are associated with heavy, abnormal rainfall due to El Niño Southern Oscillation (ENSO) Events.^10^ We calculated the standard deviation of monthly precipitation across years for each of the 12 calendar months. This standard deviation covariate is meant to identify areas most susceptible to changes in precipitation due to ENSO and other climatic cycles. We used two soil covariates to account for two different mechanisms that can cause flooding.^11^ A soil density covariate represents a region’s tendency for infiltration excess flooding, which occurs when the rate of rainfall is greater than the rate at which it can be absorbed. Another covariate, soil saturated water content, represents propensity for saturation excess flooding, which occurs when soil cannot hold excess precipitation.

To detect patterns related to suitable mosquito habitats, we used temperature as a covariate, since some mosquitoes have been shown to prefer some temperatures.^12^ We also used an average Enhanced Vegetation Index (EVI) covariate, since a range of a similar covariates has previously been used to describe areas generally suitable for RVF^13^ and since increased vegetation has been correlated with increased mosquito suitability.^14^ This covariate was not computed for each month. It was created by averaging monthly EVI values across all months for years 2002–2016, which were the years for which we had complete datasets. We show the relative effect that each covariate had on the model, as well as how values of this covariate affected suitability predictions (Appendix Figure 9). Shown on these same plots is the distribution of occurrence data. Predictions in areas with data that aligned with the distributions had lower levels of uncertainty than predictions in areas that did not have covariate values that aligned with values associated with occurrence data.

Appendix Figure 9: Covariate effects (part 1 of 2)

The effects of each covariate on suitability predictions, as well as their relative influence on the model and the distribution of occurrence data in each covariate space, are shown below. The six covariates with the highest levels of relative influence are shown in this figure.


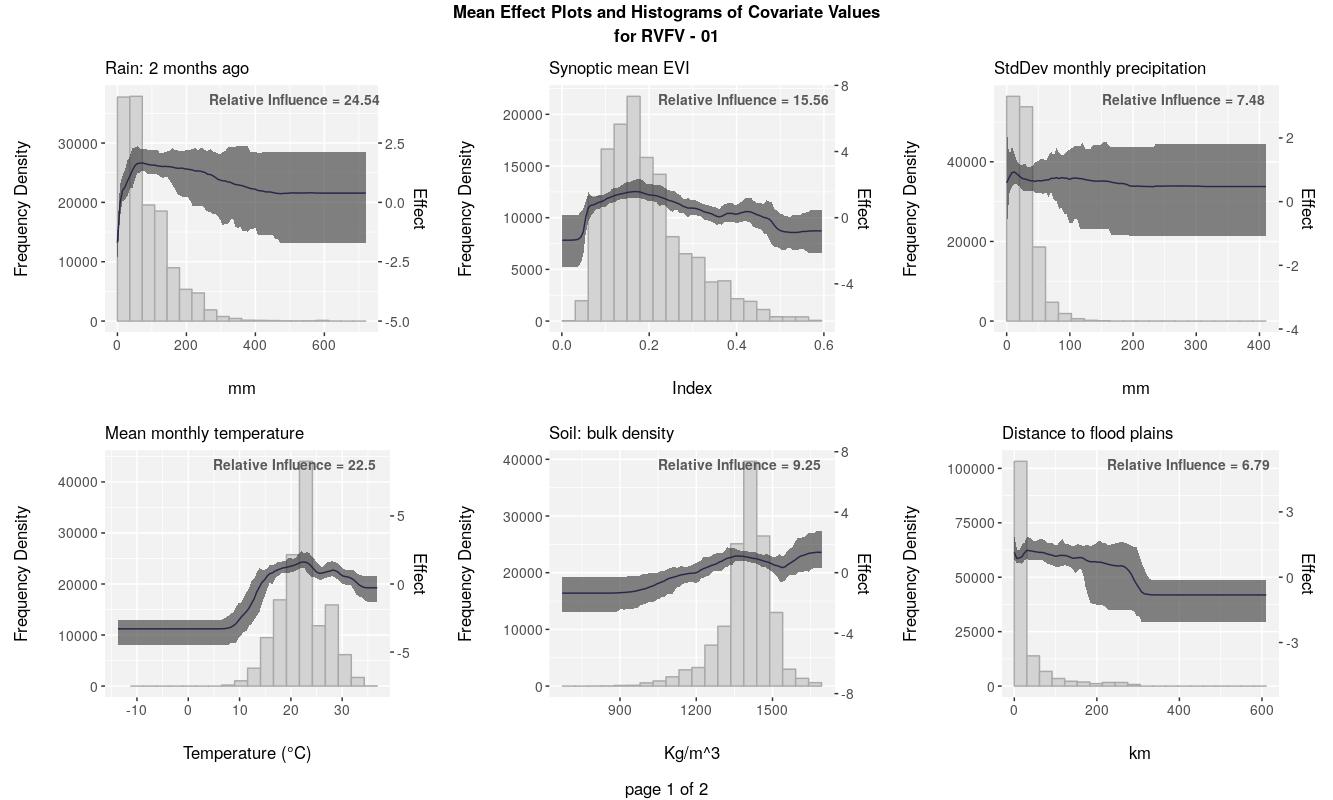


Appendix Figure 10: Covariate effects (part 2 of 2)

The effects of each covariate on suitability predictions, as well as their relative influence on the model and the distribution of occurrence data in each covariate space, are shown below. The three covariates with the lowest levels of relative influence are shown in this figure.


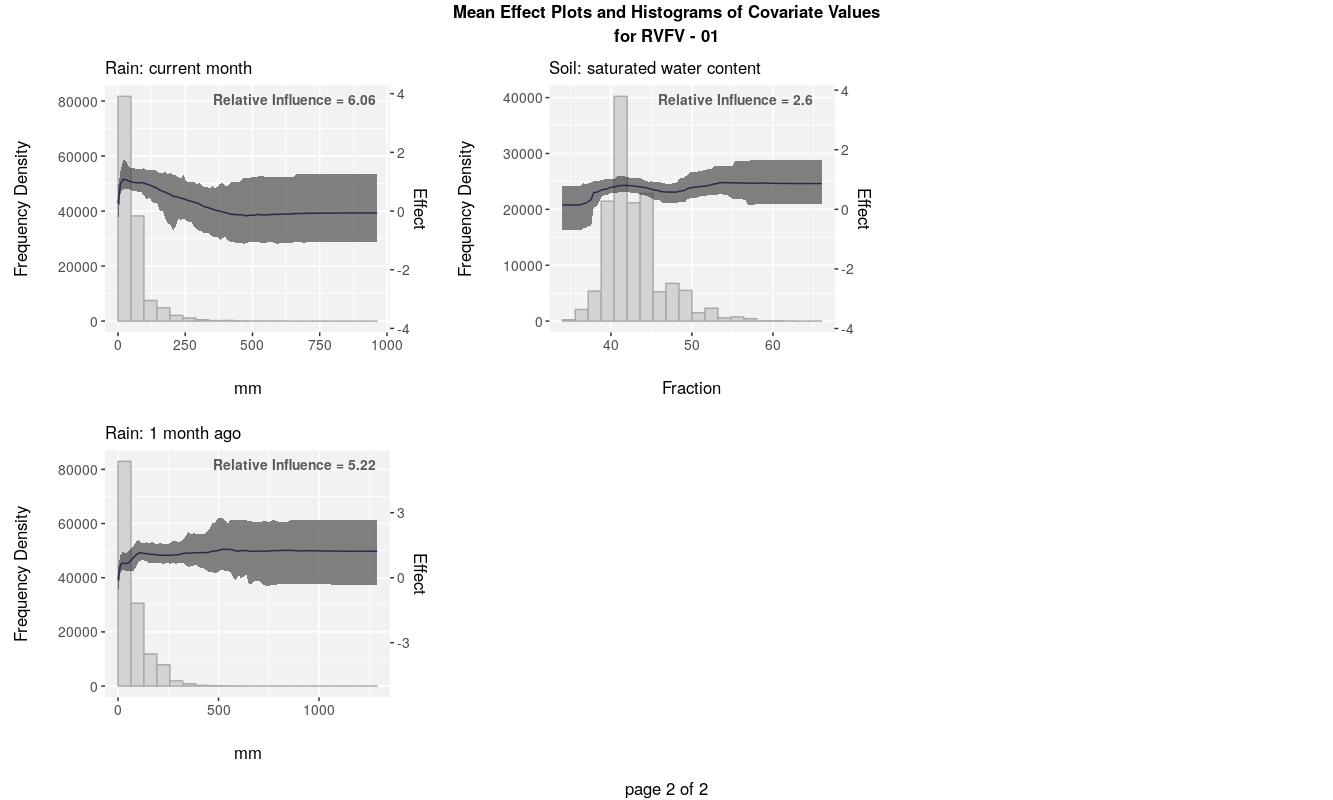


Appendix Table 4: Model covariates and citations

Listed are the covariates used in our boosted regression trees model along with their sources and citations where the datasets can be accessed. All data were processed to align with a 5 × 5-km pixel geographical representation of the earth.

| **Covariate** | **Source** | **Reference (raw data)** | **NID** |
| --- | --- | --- | --- |
| Monthly rainfall (current month) | Beck et al. 2017 | Beck, H.E., A.I.J.M. van Dijk, V. Levizzani, J. Schellekens, D.G. Miralles, B. Martens, & A. de Roo. MSWEP: 3-hourly 0.25 global gridded precipitation (1979-2015) by merging gauge, satellite, and reanalysis data. Hydrology and Earth System Sciences 21(1), 589-615 (2017).  Available at: https://data.princetonclimate.com/opendap | 419634 |
| Monthly rainfall (one month prior) |  |  |  |
| Monthly rainfall (two months prior) |  |  |  |
| Standard deviation of monthly precipitation across years |  |  |  |
| Distance to closest floodplain | NASA STRM Digital Elevation model (derived) | Nardi, F., Di Baldassarre, G., Vivoni, E.R. & Grimaldi, S. Data Descriptor: GFPLAIN250m, a global high-resolution dataset of Earth’s floodplains. Nature: Scientific Data, (2019).  Available at: https://figshare.com/articles/GFPLAIN250m/6665165/1 | 419339 |
| Saturated water content of soil | ISRIC | Hengl, T. et al. SoilGrids1km - Global Soil Information Based on Automated Mapping. PLOS ONE 9, e105992 (2014). Hengl, T. et al. Mapping Soil Properties of Africa at 250 m Resolution: Random Forests Significantly Improve Current Predictions. PLOS ONE 10, e0125814 (2015).  Available at: http://geonode.isric.org | 419642 |
| Bulk density of soil |  |  |  |
| Monthly mean temperature | CRUTS | Harris, I., Jones, P. d., Osborn, T. j. & Lister, D. h. Updated high-resolution grids of monthly climatic observations – the CRU TS3.10 dataset. Int. J. Climatol. 34, 623–642 (2014).  University of East Anglia. Climatic Research Unit TS v. 3.24 dataset. Available at: https://crudata.uea.ac.uk/cru/data/hrg/ | 281800 |
| Mean enhanced vegetation index over all months and years | MODIS | Schaaf, C. & Wang, Z. MCD43A1 MODIS/Terra+Aqua BRDF/Albedo Model Parameters Daily L3 Global - 500m V006. NASA EOSDIS Land Processes DAAC.  Available at: https://modis.gsfc.nasa.gov/data/dataprod/mod43.php | 422343 |

### 3.2.4 Multivariate environmental similarity surface (MESS) analysis

The covariate space occupied by the data onto which we make predictions is not necessarily all contained within the covariate space occupied by our modelled occurrence data. To assess which locations had covariate values that were contained in the space occupied by our occurrence data, we conducted a multivariate environmental similarity surface (MESS) analysis.^15^ For the geographical space for which we made predictions, this analysis quantifies how similar locations were in environmental covariate space to the space occupied by occurrence data. Positive values indicated that our model was interpolating predictions from occurrence data, while negative values indicated that the predictions were being extrapolated. We would have less confidence in extrapolated predictions, as these would be based on data dissimilar from that associated with our occurrence records. We conducted this analysis for all years across each month and report the percentage of years each pixel showed positive MESS values, which represented interpolation, in each month.

Appendix Figure 11: January multivariate environmental similarity surface analysis

For each month in every year of our analysis, we conducted a multivariate environmental similarity surface (MESS) analysis. This map shows, for this month, the percentage of years from 1995 to 2016 where MESS values were positive, indicating model interpolation.


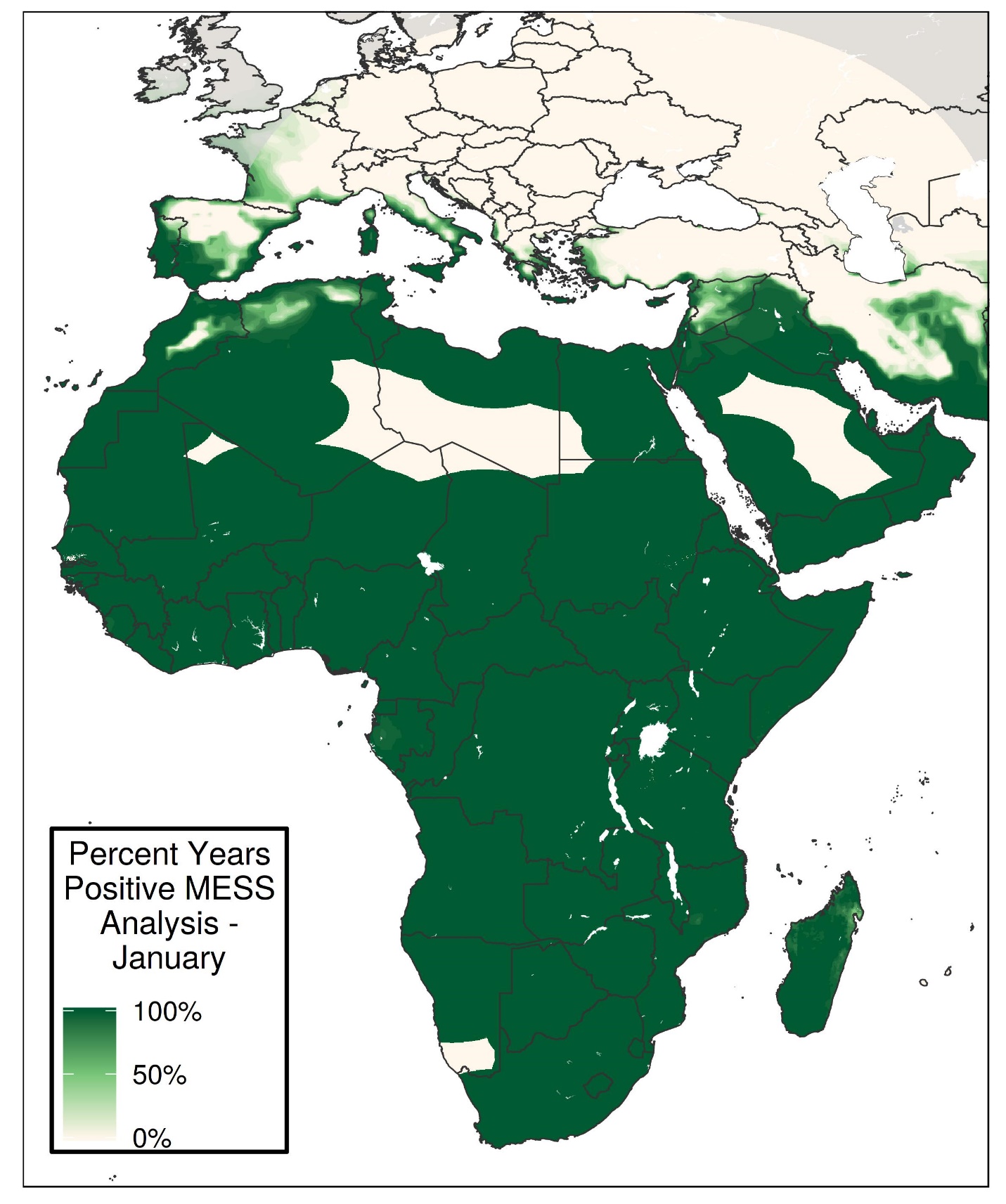


Appendix Figure 12: February multivariate environmental similarity surface

For each month in every year of our analysis, we conducted a multivariate environmental similarity surface (MESS) analysis. This map shows, for this month, the percentage of years from 1995 to 2016 where MESS values were positive, indicating model interpolation.


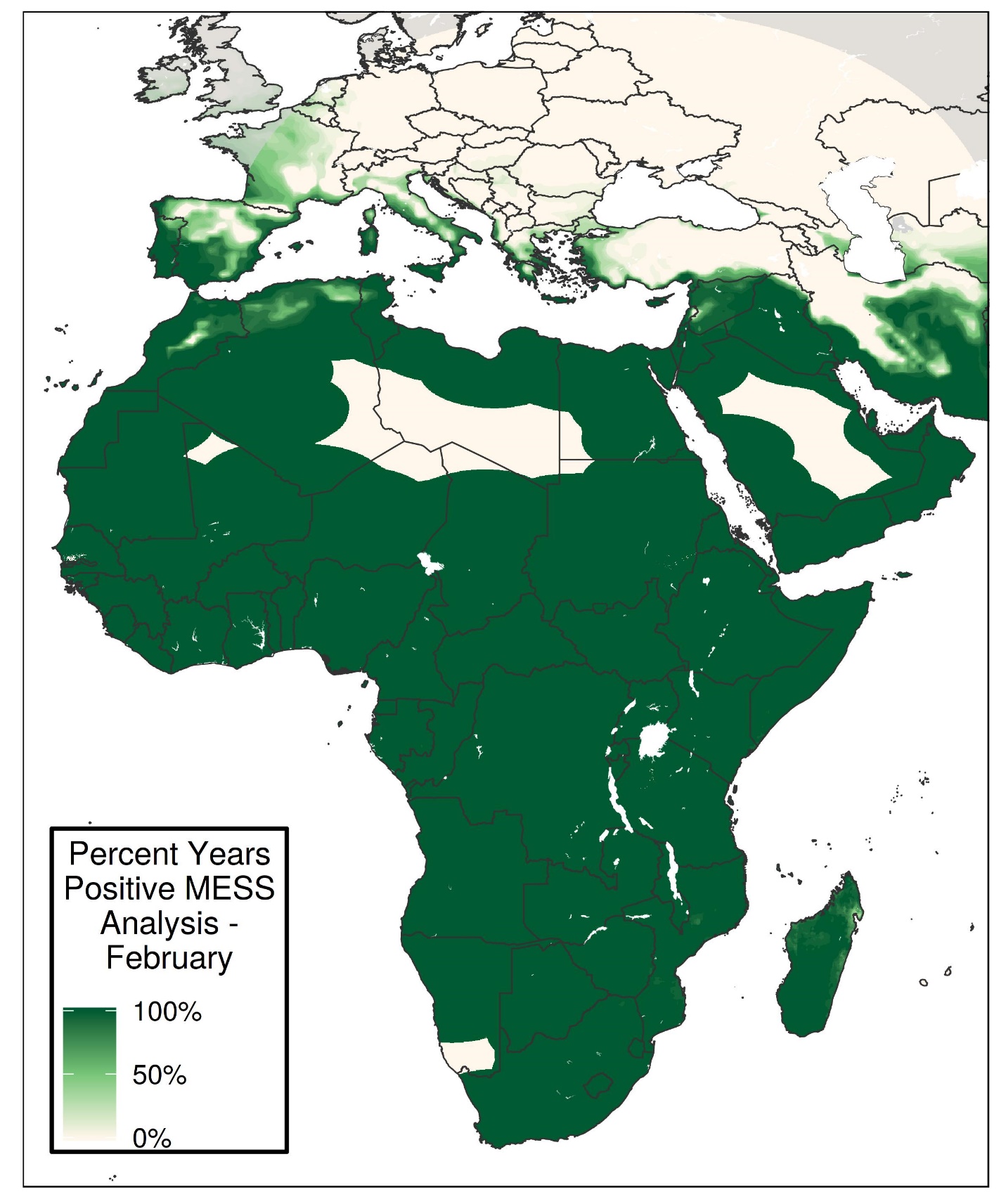


Appendix Figure 13: March multivariate environmental similarity surface analysis

For each month in every year of our analysis, we conducted a multivariate environmental similarity surface (MESS) analysis. This map shows, for this month, the percentage of years from 1995 to 2016 where MESS values were positive, indicating model interpolation.


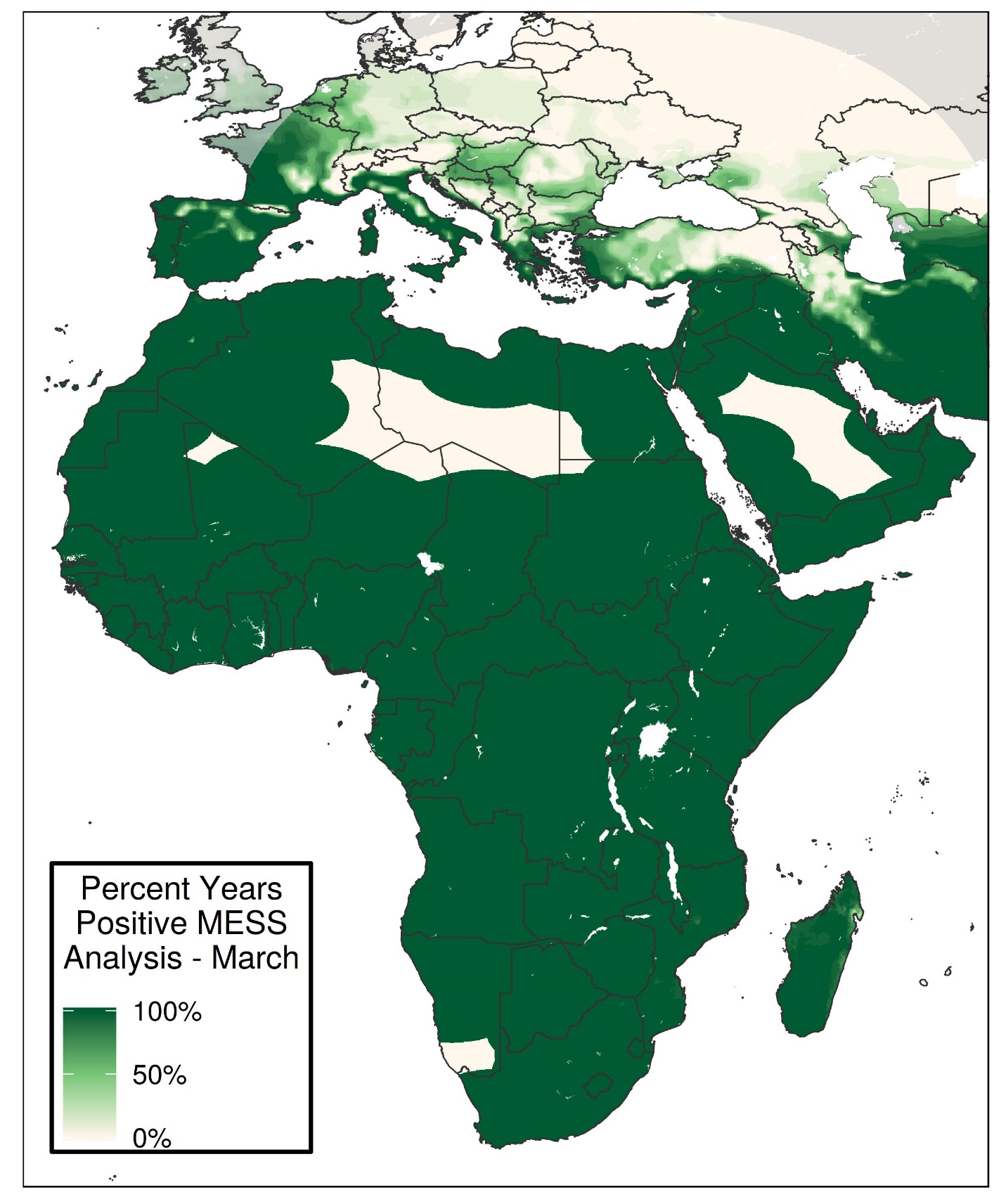


Appendix Figure 14: April multivariate environmental similarity surface analysis

For each month in every year of our analysis, we conducted a multivariate environmental similarity surface (MESS) analysis. This map shows, for this month, the percentage of years from 1995 to 2016 where MESS values were positive, indicating model interpolation.


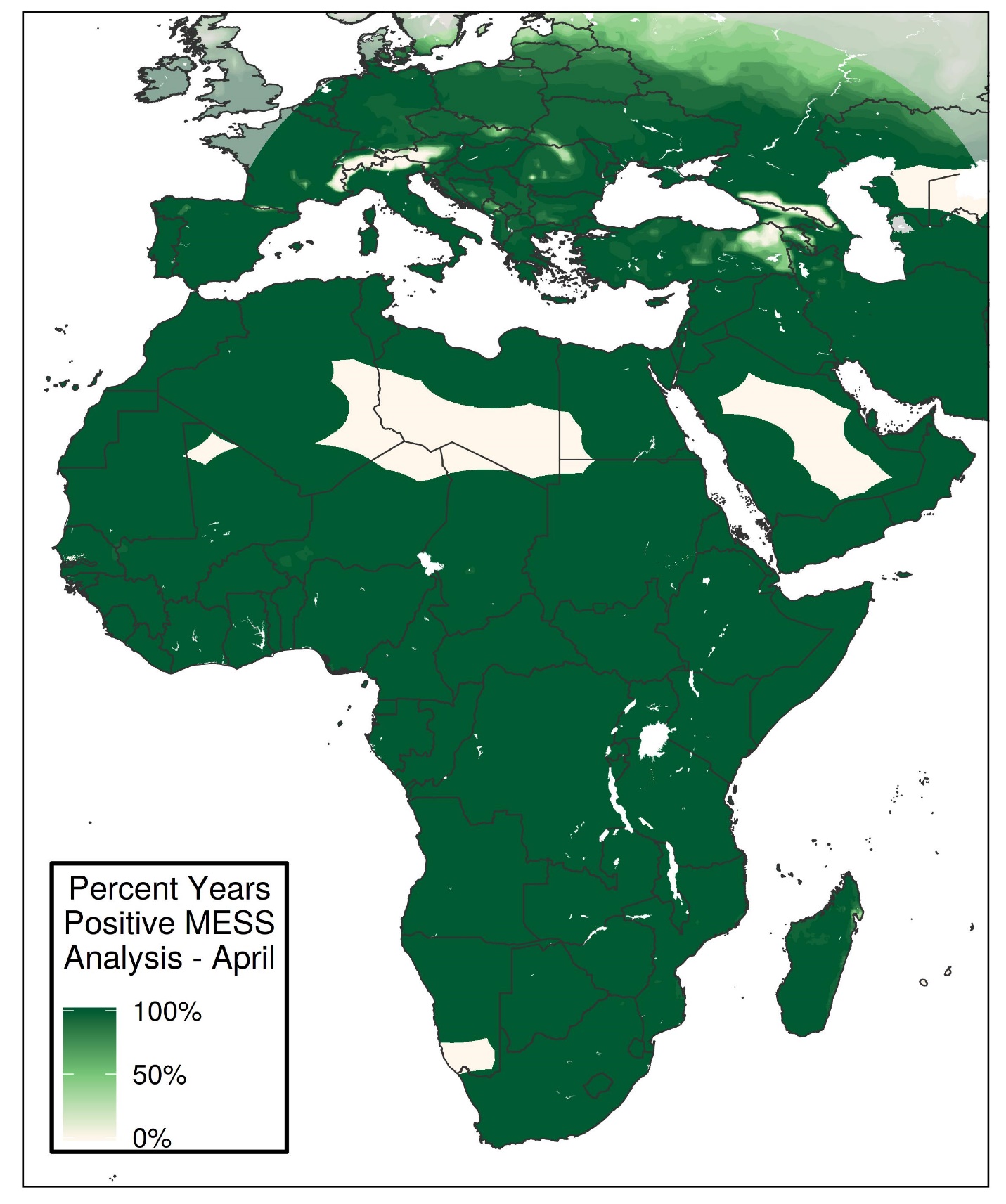


Appendix Figure 15: May multivariate environmental similarity surface analysis

For each month in every year of our analysis, we conducted a multivariate environmental similarity surface (MESS) analysis. This map shows, for this month, the percentage of years from 1995 to 2016 where MESS values were positive, indicating model interpolation.


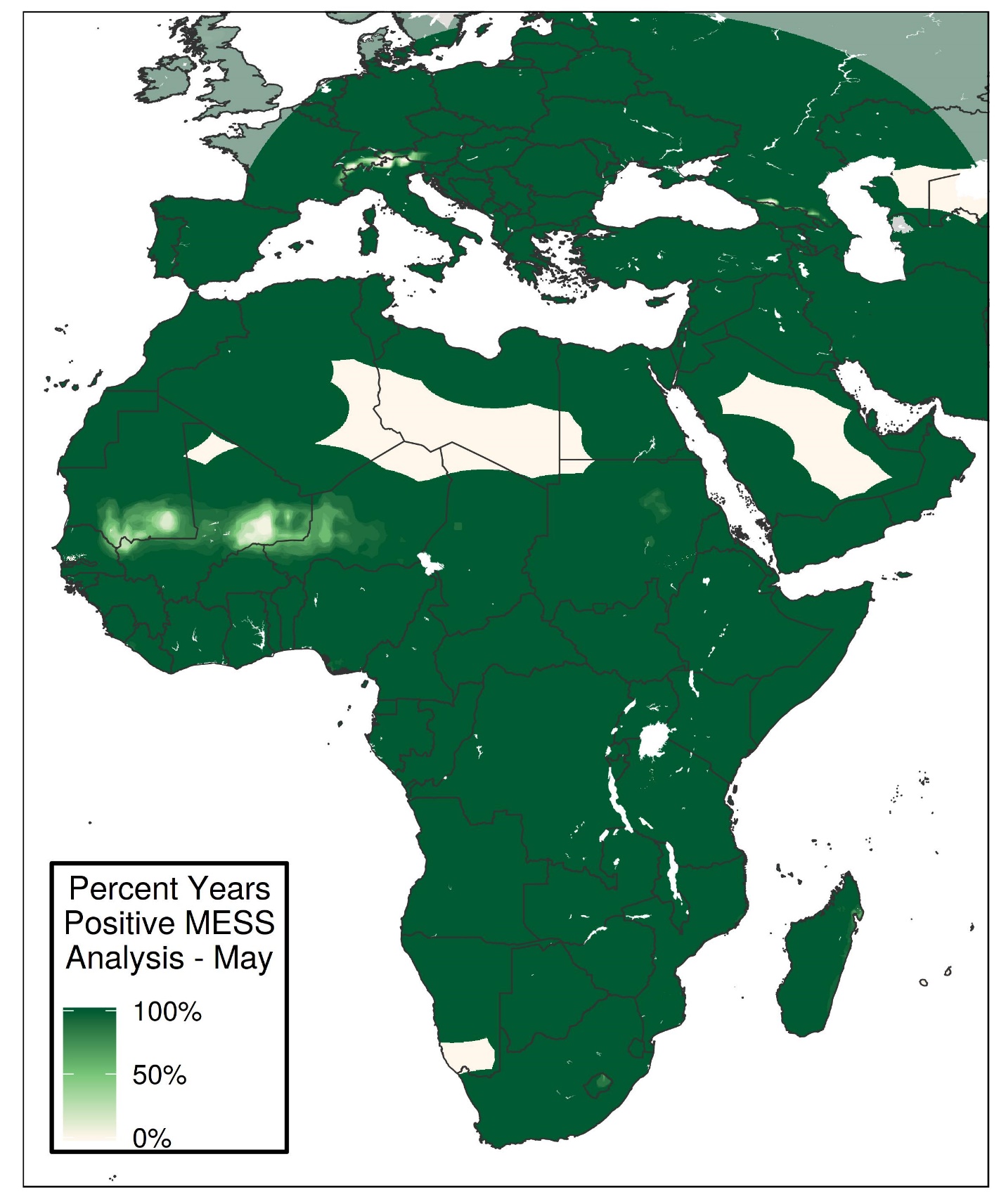


Appendix Figure 16: June multivariate environmental similarity surface analysis

For each month in every year of our analysis, we conducted a multivariate environmental similarity surface (MESS) analysis. This map shows, for this month, the percentage of years from 1995 to 2016 where MESS values were positive, indicating model interpolation.


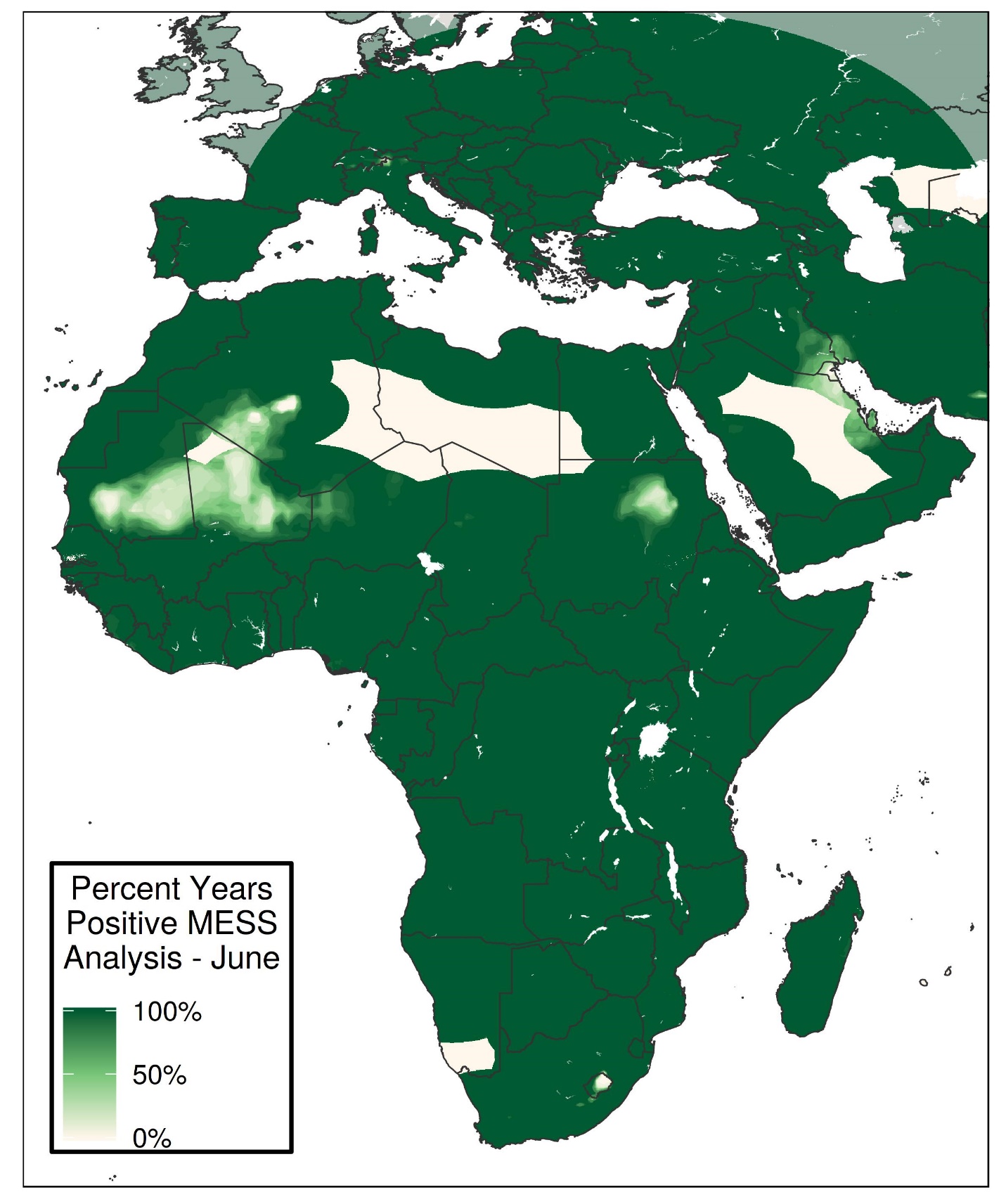


Appendix Figure 17: July multivariate environmental similarity surface analysis

For each month in every year of our analysis, we conducted a multivariate environmental similarity surface (MESS) analysis. This map shows, for this month, the percentage of years from 1995 to 2016 where MESS values were positive, indicating model interpolation.


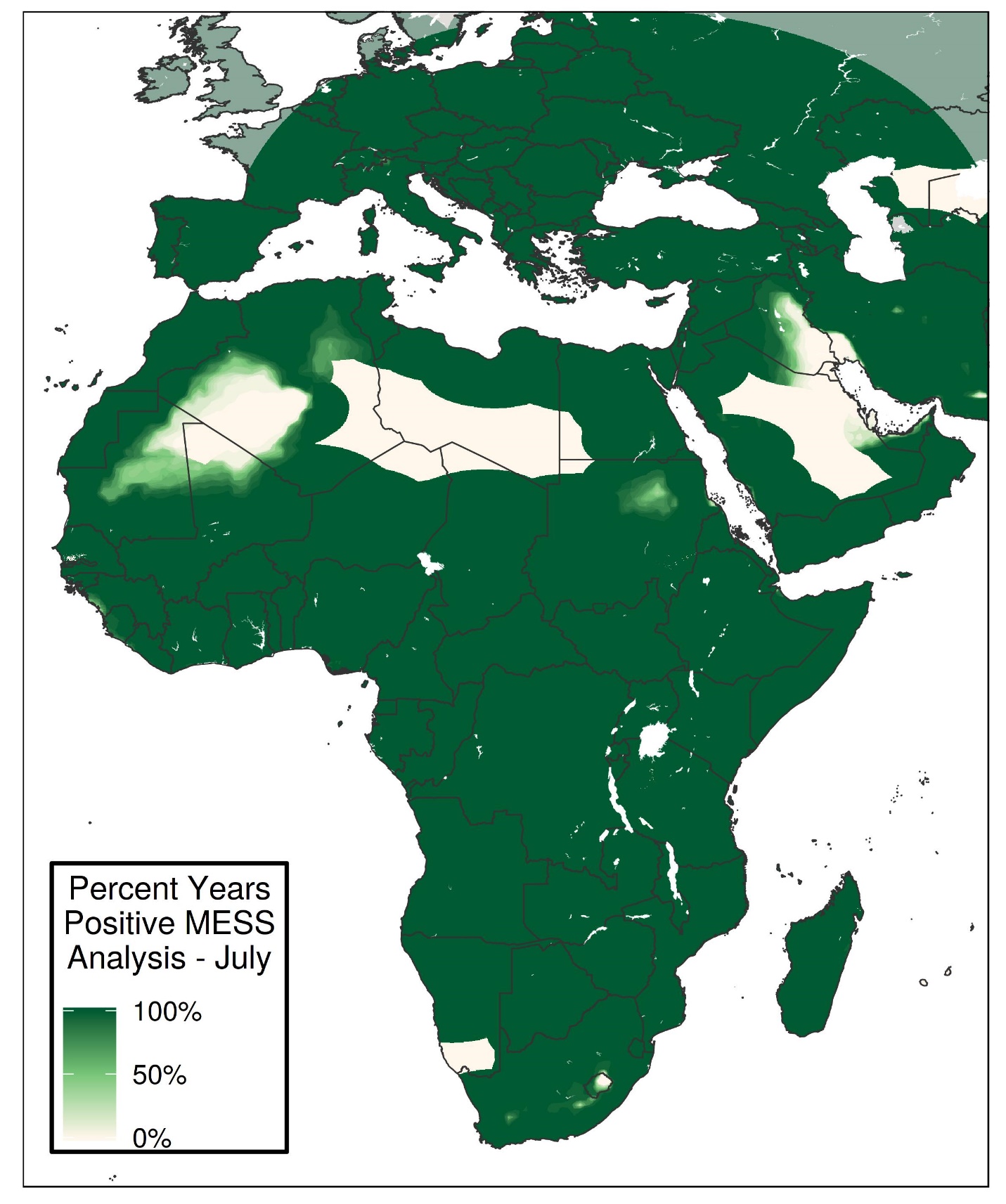


Appendix Figure 18: August multivariate environmental similarity surface analysis

For each month in every year of our analysis, we conducted a multivariate environmental similarity surface (MESS) analysis. This map shows, for this month, the percentage of years from 1995 to 2016 where MESS values were positive, indicating model interpolation.


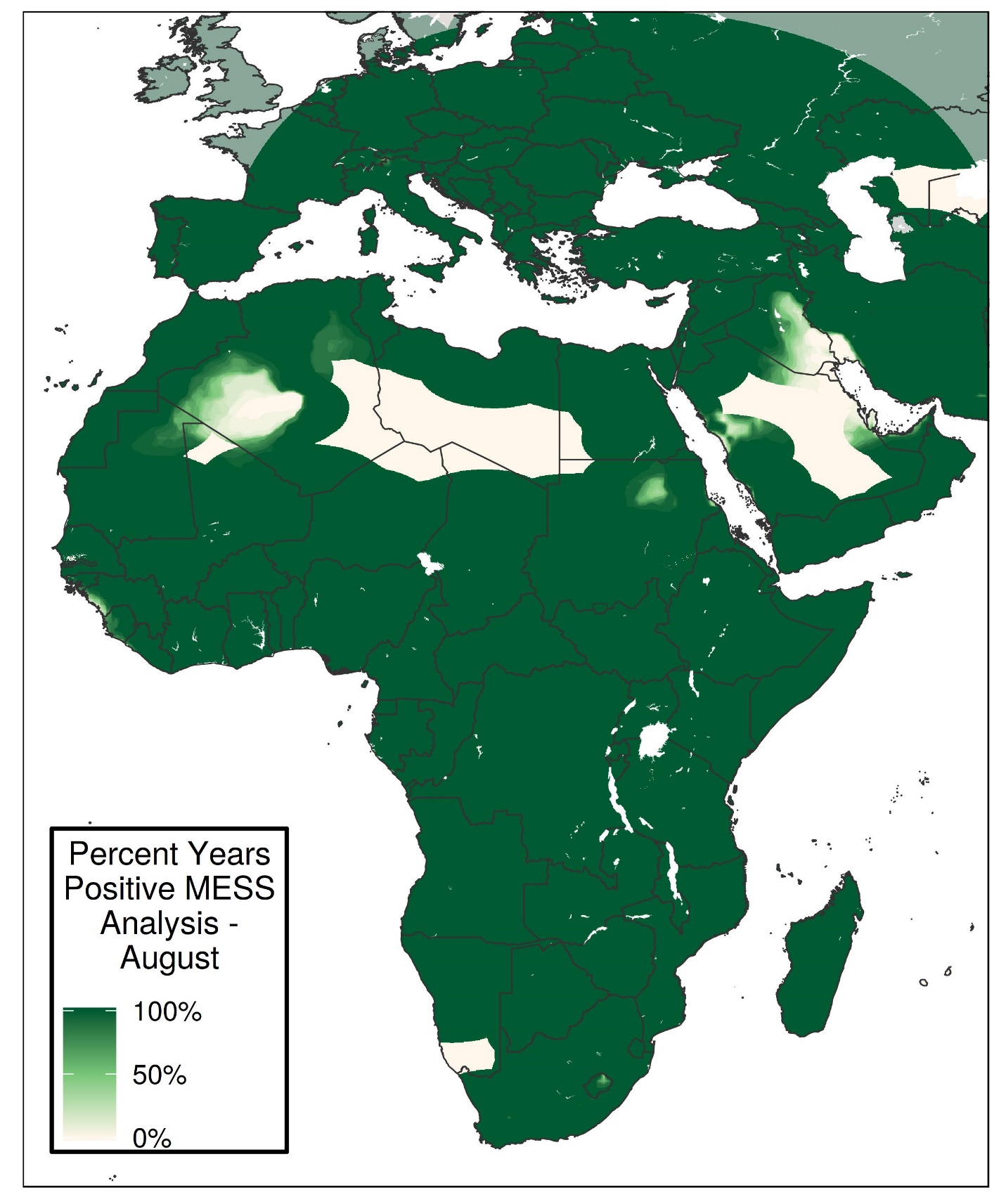


Appendix Figure 19: September multivariate environmental similarity surface analysis

For each month in every year of our analysis, we conducted a multivariate environmental similarity surface (MESS) analysis. This map shows, for this month, the percentage of years from 1995 to 2016 where MESS values were positive, indicating model interpolation.


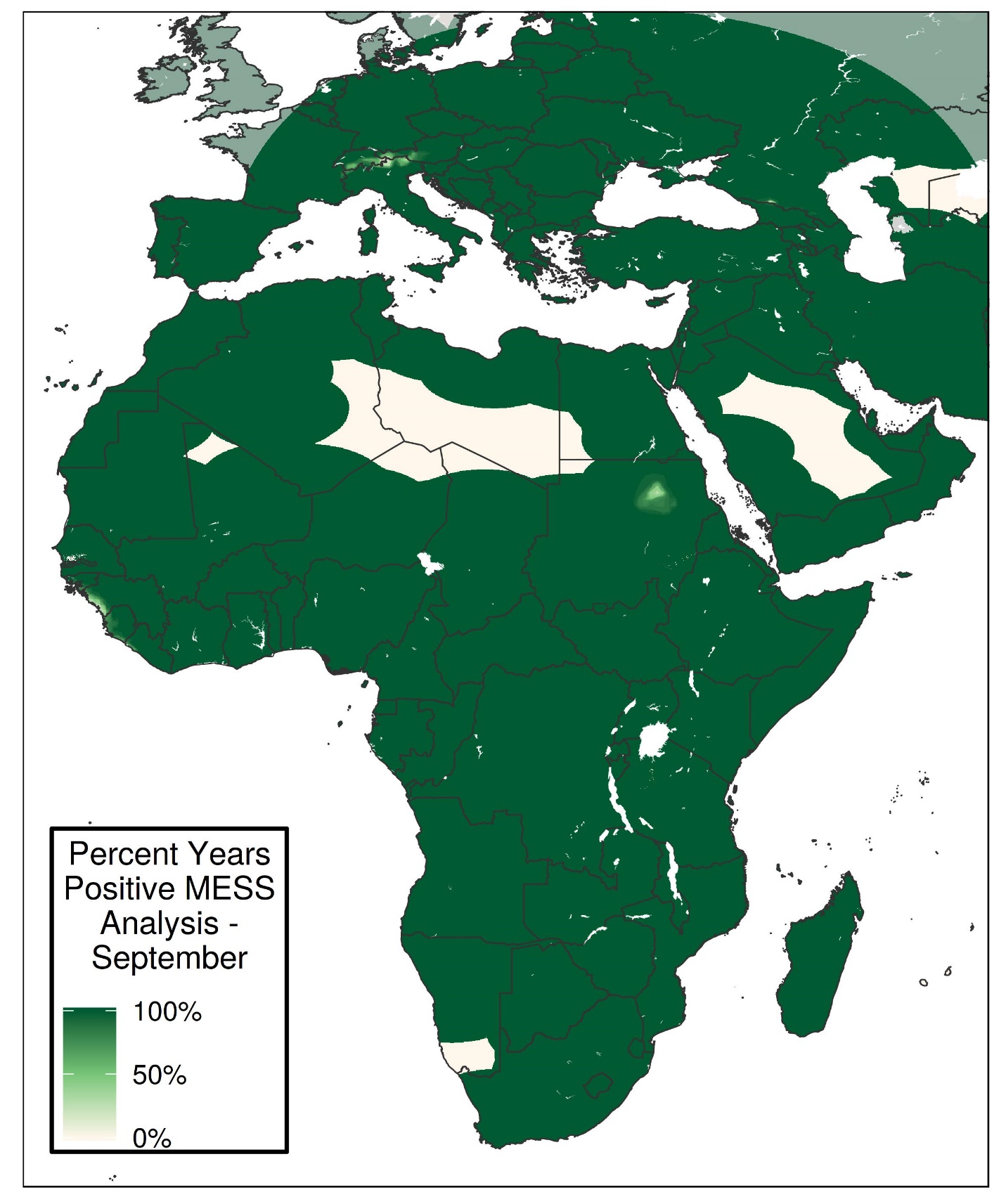


Appendix Figure 20: October multivariate environmental similarity surface analysis

For each month in every year of our analysis, we conducted a multivariate environmental similarity surface (MESS) analysis. This map shows, for this month, the percentage of years from 1995 to 2016 where MESS values were positive, indicating model interpolation.


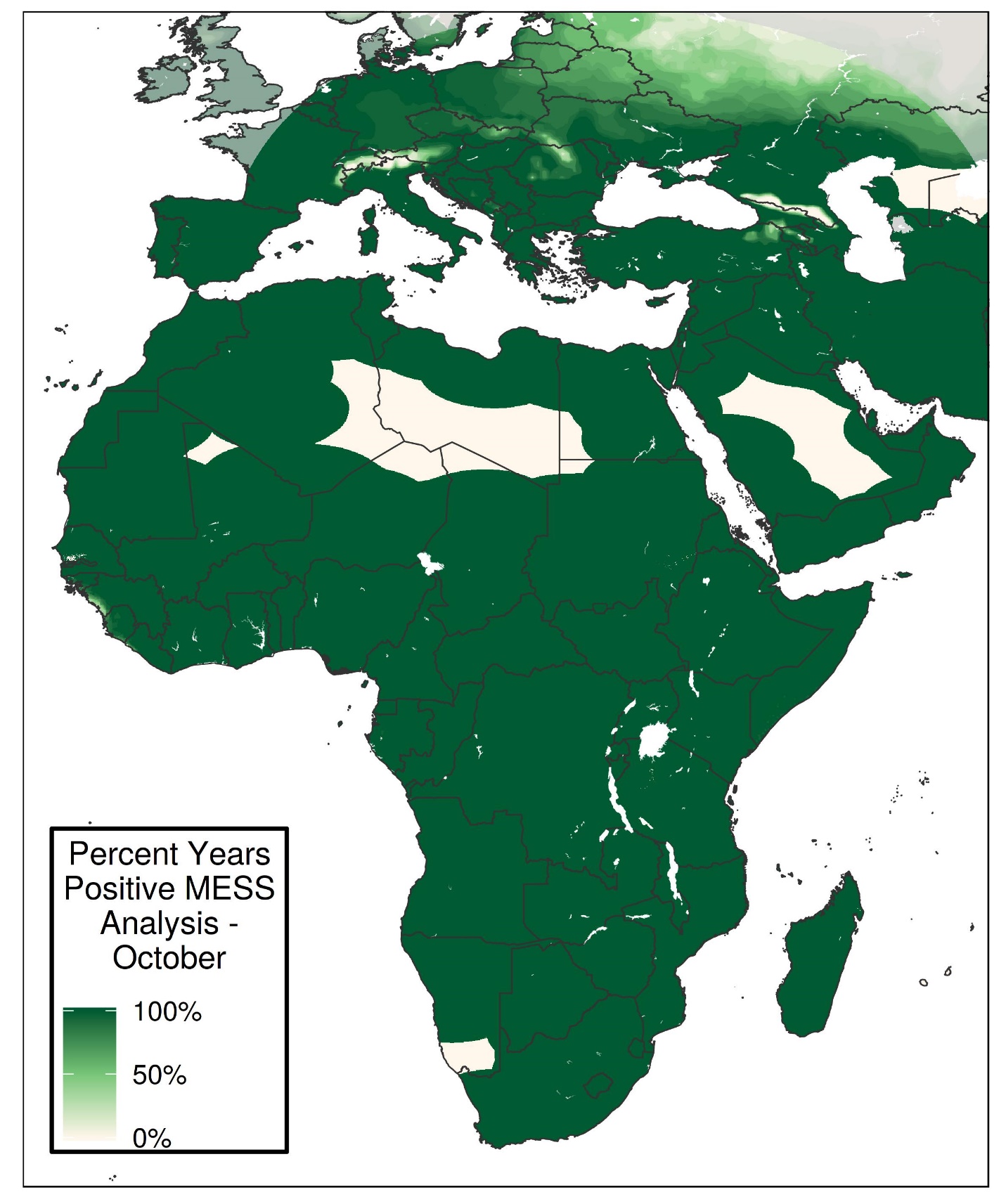


Appendix Figure 21: November multivariate environmental similarity surface analysis

For each month in every year of our analysis, we conducted a multivariate environmental similarity surface (MESS) analysis. This map shows, for this month, the percentage of years from 1995 to 2016 where MESS values were positive, indicating model interpolation.


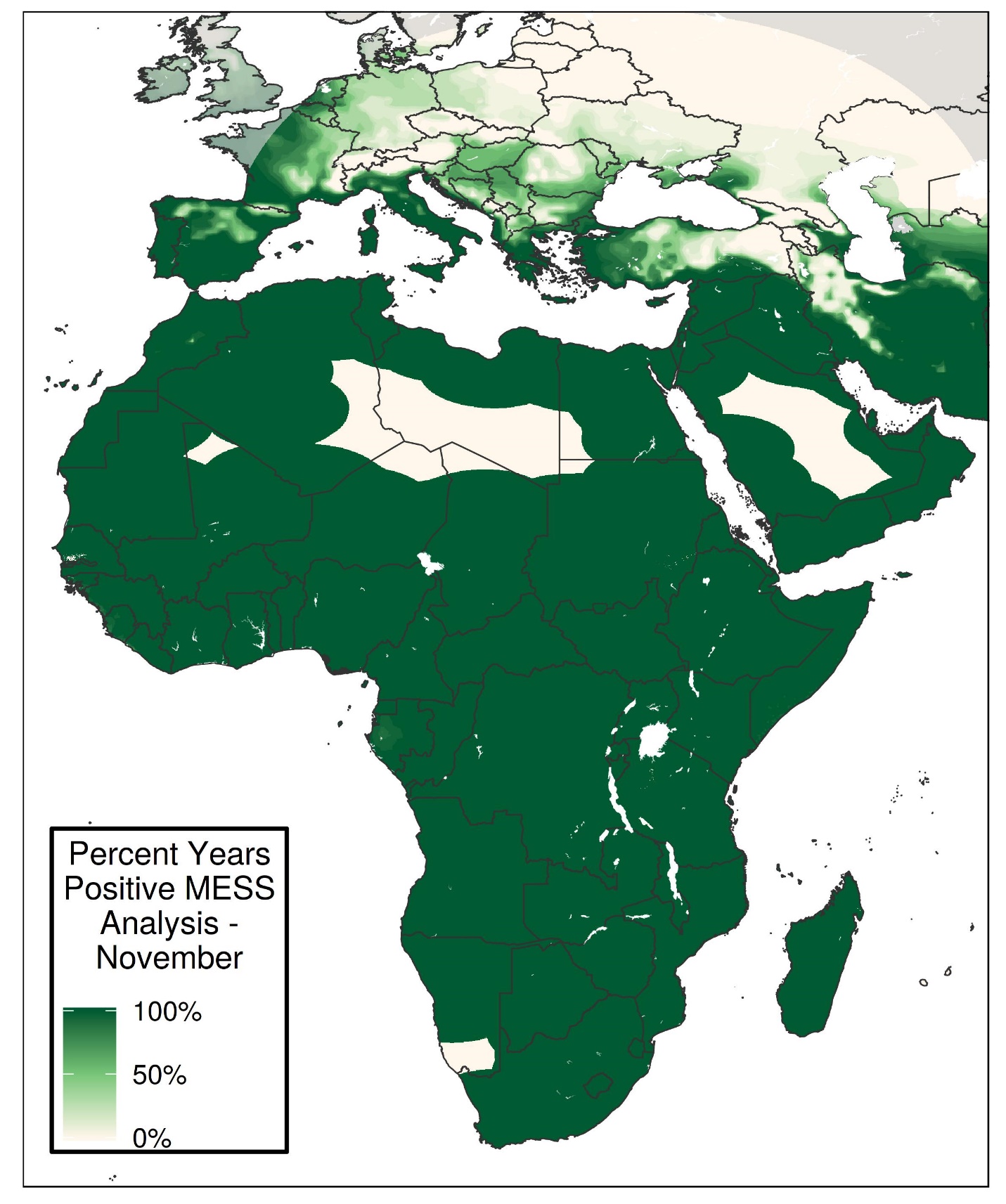


Appendix Figure 22: December multivariate environmental similarity surface analysis

For each month in every year of our analysis, we conducted a multivariate environmental similarity surface (MESS) analysis. This map shows, for this month, the percentage of years from 1995 to 2016 where MESS values were positive, indicating model interpolation.


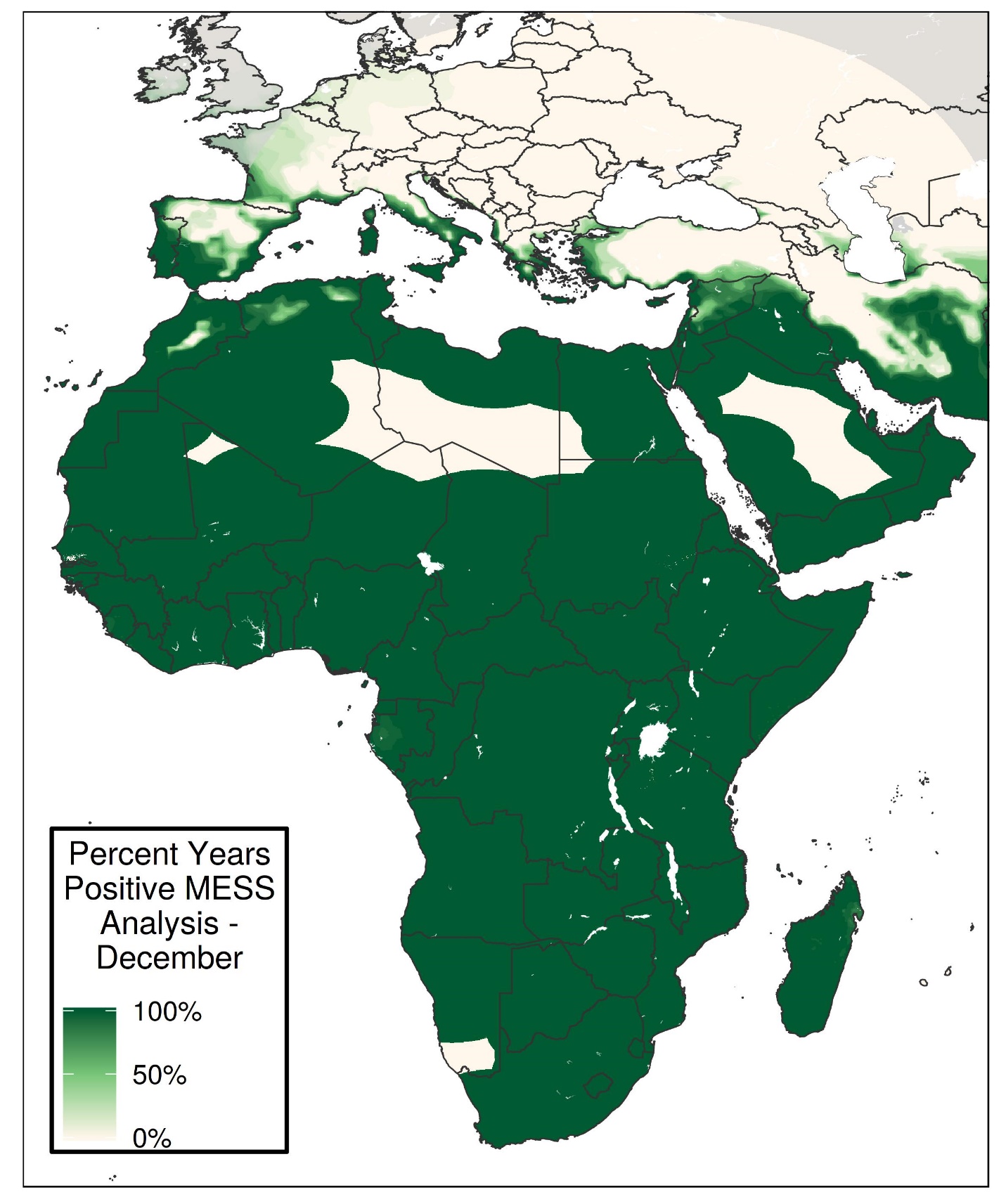


### 3.2.5 Evaluating model performance

To assess the performance of the internal machinery of our model, we calculated the area under the curve for each bootstrap (AUC_boot_). The bootstrap was evaluated on how well it could classify occurrence and background data using the datapoints’ associated covariate values without knowledge of the month or year in which they occurred. In contrast, once we had made predictions across all months for all 22 years, we averaged the suitability across years for each month and evaluated the predictive performance of these outputs by calculating the area under the curve for each monthly synoptic map (AUC_syn_). AUC_syn_ values for each month are shown below.

Appendix Table 5: Synoptic area under the curve values for monthly maps

The area under the curve is shown for each month. These values were calculated using the predictions associated with each background and occurrence record from each month and an optimised threshold for each month defined using these predictions.

| **Month** | **AUC_syn_** |
| --- | --- |
| January | 0.969 |
| February | 0.980 |
| March | 0.979 |
| April | 0.965 |
| May | 0.923 |
| June | 0.822 |
| July | 0.780 |
| August | 0.775 |
| September | 0.811 |
| October | 0.900 |
| November | 0.934 |
| December | 0.953 |

# 4.0 Results aggregation

## 4.1 Suitable months per year

We made suitability predictions for each pixel across our defined RVF range for every month between 1995 and 2016. Each one of these prediction maps was composed of values that ranged from 0 to 1. For each month-year combination, we determined an optimal threshold that minimised the Pythagorean distance between the point where sensitivity = 1 and (1 – specificity) = 0 when the two values were plotted against each other for different thresholds between 0 or 1 on a receiver operating curve (ROC). The data we used to calculate this threshold were all data from that month across all years. For example, we evaluated the January 2002 map using all data from January, whether background or occurrence. Once we determined the threshold, we applied it across all of our predictions for that month and year. Pixels with predicted values greater than or equal to the threshold were given values of 1, while those with lower values were assigned values of 0. Across all months and years, we added how many times each pixel was given a value of 1 and then divided this number by 22 (the total number of years) to determine the average number of suitable months per year.

Appendix Figure 23: Suitable months per year aggregation

A graphical representation of how we calculated average suitable months per year is shown.


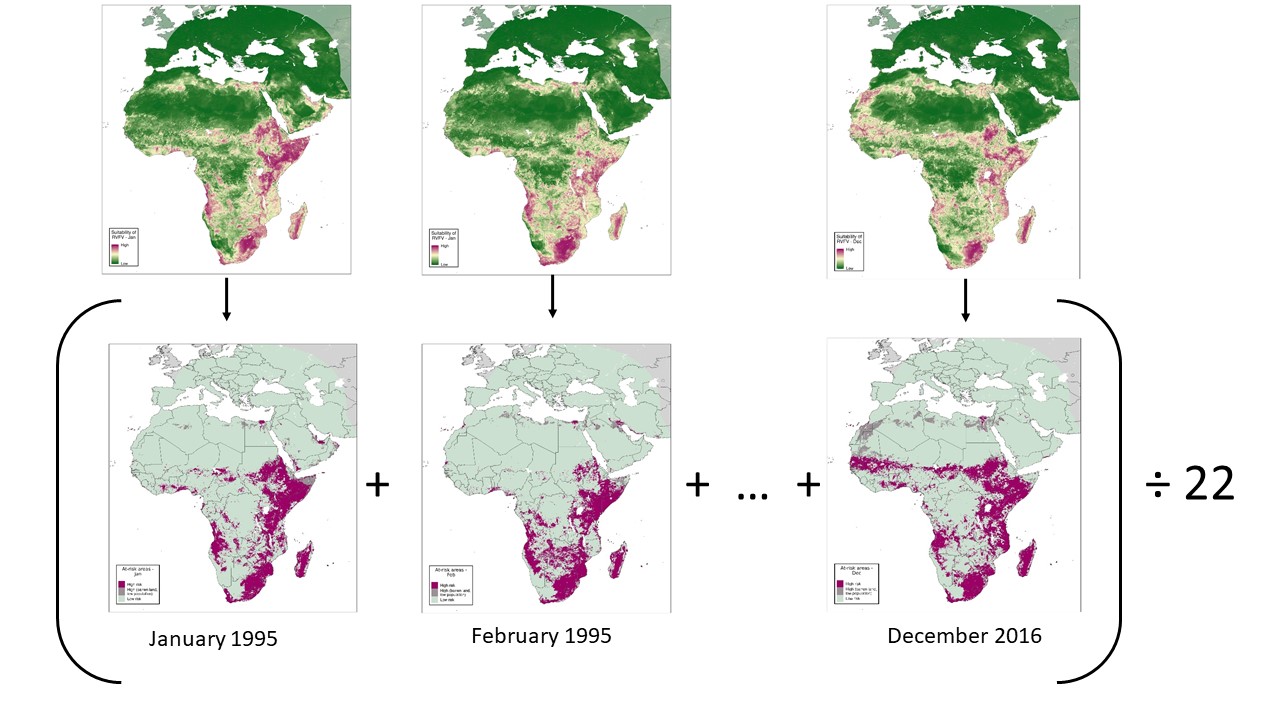


## 4.2 Mean suitability maps

For each of the 12 calendar months, we averaged the continuous suitability values for each pixel across all years. The resulting 12 maps still contain values ranging from 0 to 1.

Appendix Figure 24: Monthly suitability aggregation

A graphical representation of how we calculated mean monthly suitability is shown.


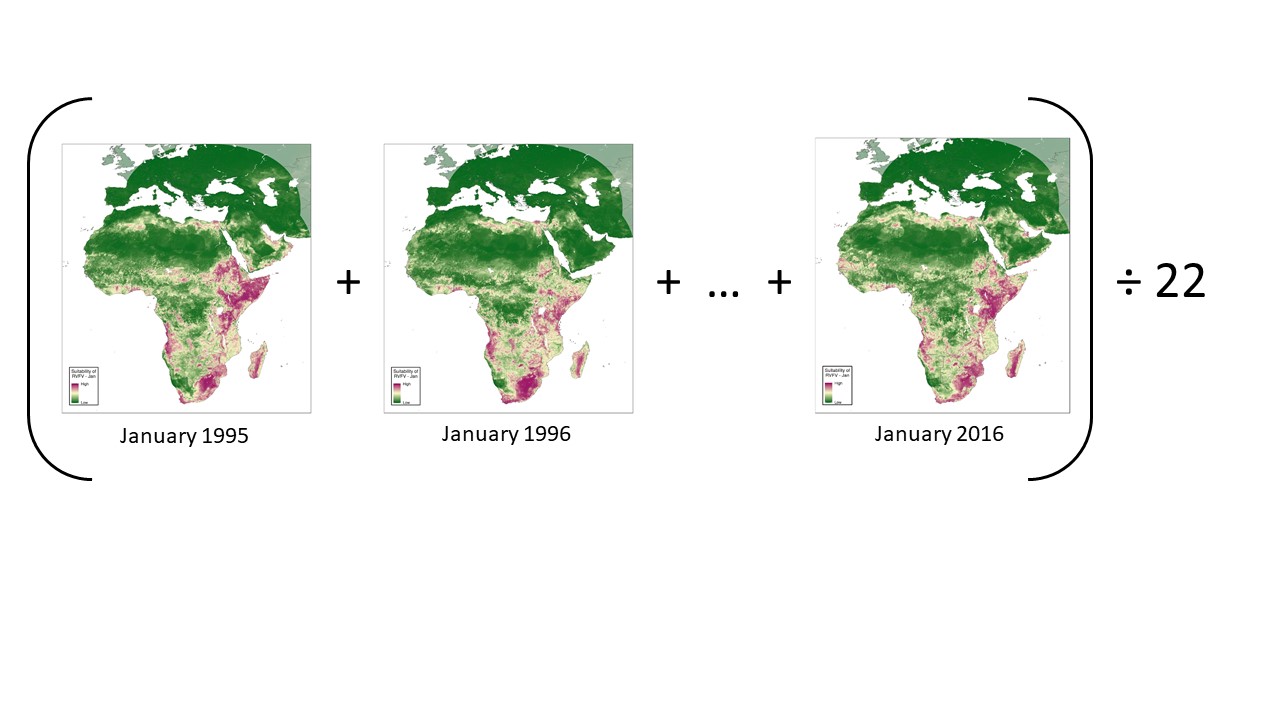


## 4.3 Mean uncertainty

We generated an uncertainty map for each month-year for which we made predictions. The uncertainty of each pixel was given as the difference between the 2.5th and the 97.5th percentiles of that pixel’s predicted values across all 100 model bootstraps. These uncertainty maps were averaged for each month across all years, similar to the mean suitability maps and are shown below.

Appendix Figure 25: Mean uncertainty aggregation


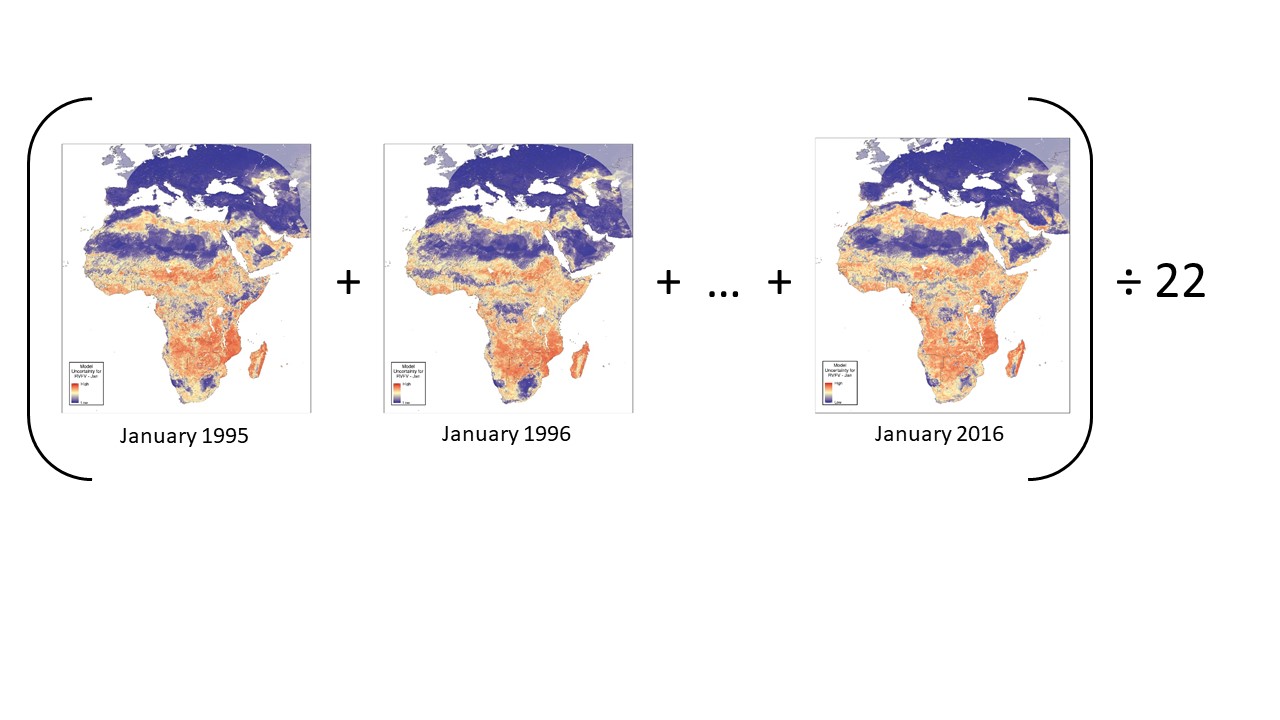


## 4.4 Synoptic binary maps, confidence intervals, and synoptic AUC

For each mean suitability map, we used all data from the map’s given month across all years to create an optimised threshold with a method like the one described in section 4.1. Using this optimised threshold, we converted the mean suitability maps into 12 monthly binary maps, where pixels could be either 0 or 1. We calculated AUC_syn_, described in section 3.2.4, using these binary predictions for occurrence and background data. For each month-year combination, we also produced two maps that represented percentile 2.5 and 97.5 of each pixel’s predictions across all 100 bootstraps. For each calendar month, these upper and lower confidence interval maps were averaged. We applied the optimised threshold, calculated above, to both of these new averaged maps to create binary maps of the upper and lower confidence intervals for each month.

In general, months with lower AUC_syn_ values had smaller amounts of data represented in the dataset. Because threshold values for individual month-year maps and synoptic monthly maps were made using all data from a given month, the thresholds in months with less data could be more volatile if more data were added, or if data were removed to test the model with a subset of data. The optimised thresholds calculated for each synoptic monthly map are shown below.

Appendix Figure 26: Occurrence data counts by month

The number of occurrence records included from each month in our modelled dataset are shown below.


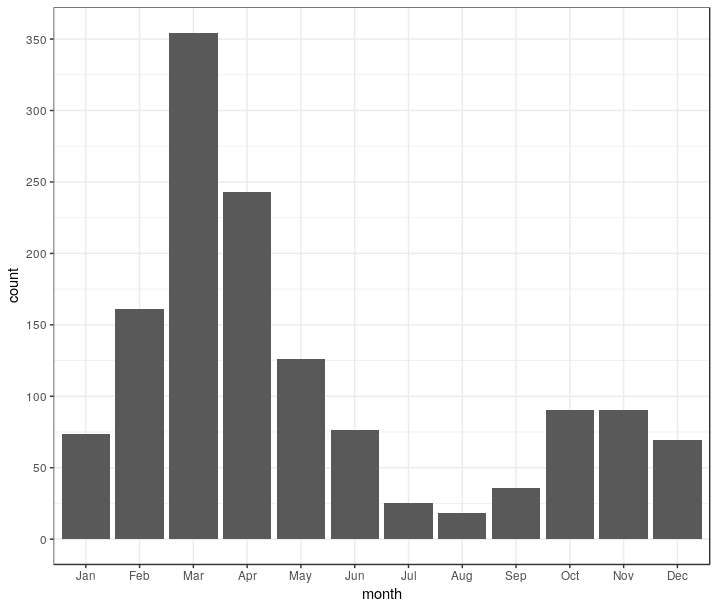


Appendix Figure 27: Optimised thresholds for monthly synoptic maps

Suitability values in all maps ranged from 0 to 1. Thresholds were calculated by optimising sensitivity and specificity values on a receiver operating curve for predictions made for occurrence and pseudoabsence points from the given month across all years of data. The thresholds calculated for each month are shown below.


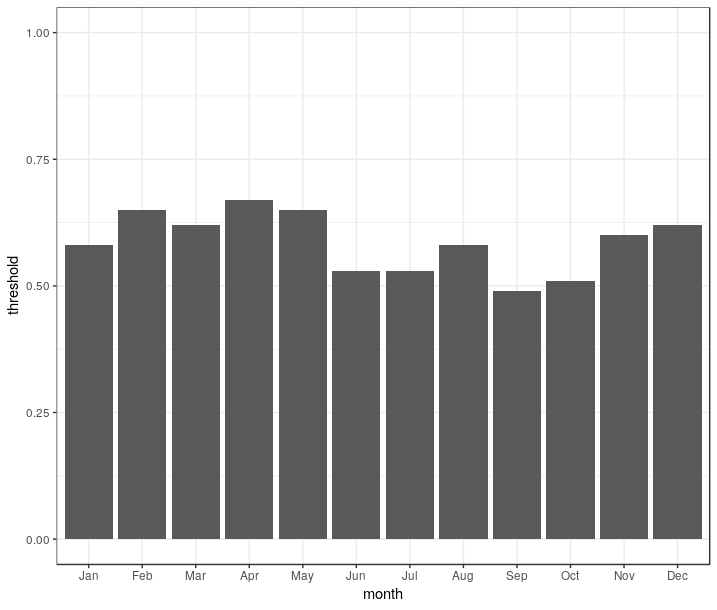


## 4.5 Spillover potential

To calculate the spillover potential across our defined range for each month between 1995 and 2016, we started with the month-year-specific binary prediction masks described in section 4.1. We then overlapped these binary suitability predictions with WorldPop population data^16^ to determine the number of people in a suitable geography, or the number of people “at risk,” in each second administrative unit, or “district,” for each month. We repeated this process to calculate the number of cattle, sheep, and goats at risk in each district using livestock population rasters.^17^ Maps of these livestock populations are shown (Appendix Figures 29-31).

We then followed a protocol similar to the one described by Pigott and colleagues 2017.^18^ We took the natural log of the number of people at risk in each district. We then found the minimum and maximum values of this log indicator across all districts, months, and years. Based on these maximum and minimum values, we scaled all values to be between 0 and 10. Next, in addition to measuring the absolute number of people at risk in each district, we calculated the proportion of the population at risk in each district. We then similarly scaled these values across all districts, months, and years to be between 0 and 10. We then took the geometric mean of these two 0-10 indicators to come up with a single value for humans between 0 and 10 for each district in each month-year. We repeated this process to obtain a similar measure for livestock (combined cattle, sheep, and goats) in these areas. The final spillover potential was given as the geometric mean of the human indicator and the livestock indicator.

Finally, we ranked each spillover value from all districts, months, and years from lowest to highest, not including districts with spillover values of 0 in a given month and year. Using these rankings, we determined the quintile that each value fell into and calculated spillover in each district in each month and year as its percentile ranking, binned into these quintiles. For each month, we then found the average quintile of each district across all years and report this value as a district’s synoptic spillover potential for a given month.

Supplemental Figure 28: Cattle population data

Here we show the cattle population data that were used as part of our spillover calculation.


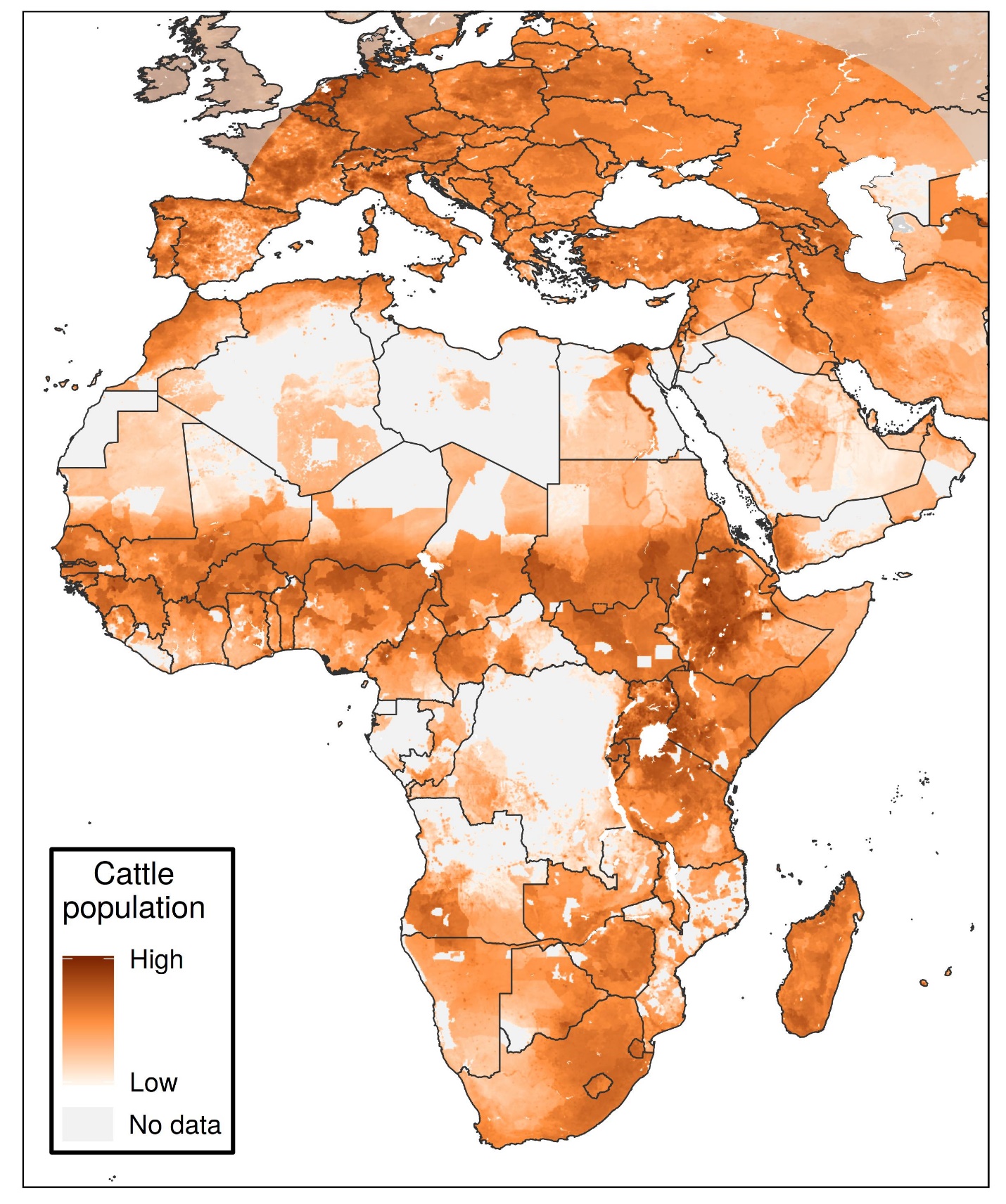


Supplemental Figure 29: Sheep population data

Here we show the sheep population data that were used as part of our spillover calculation.


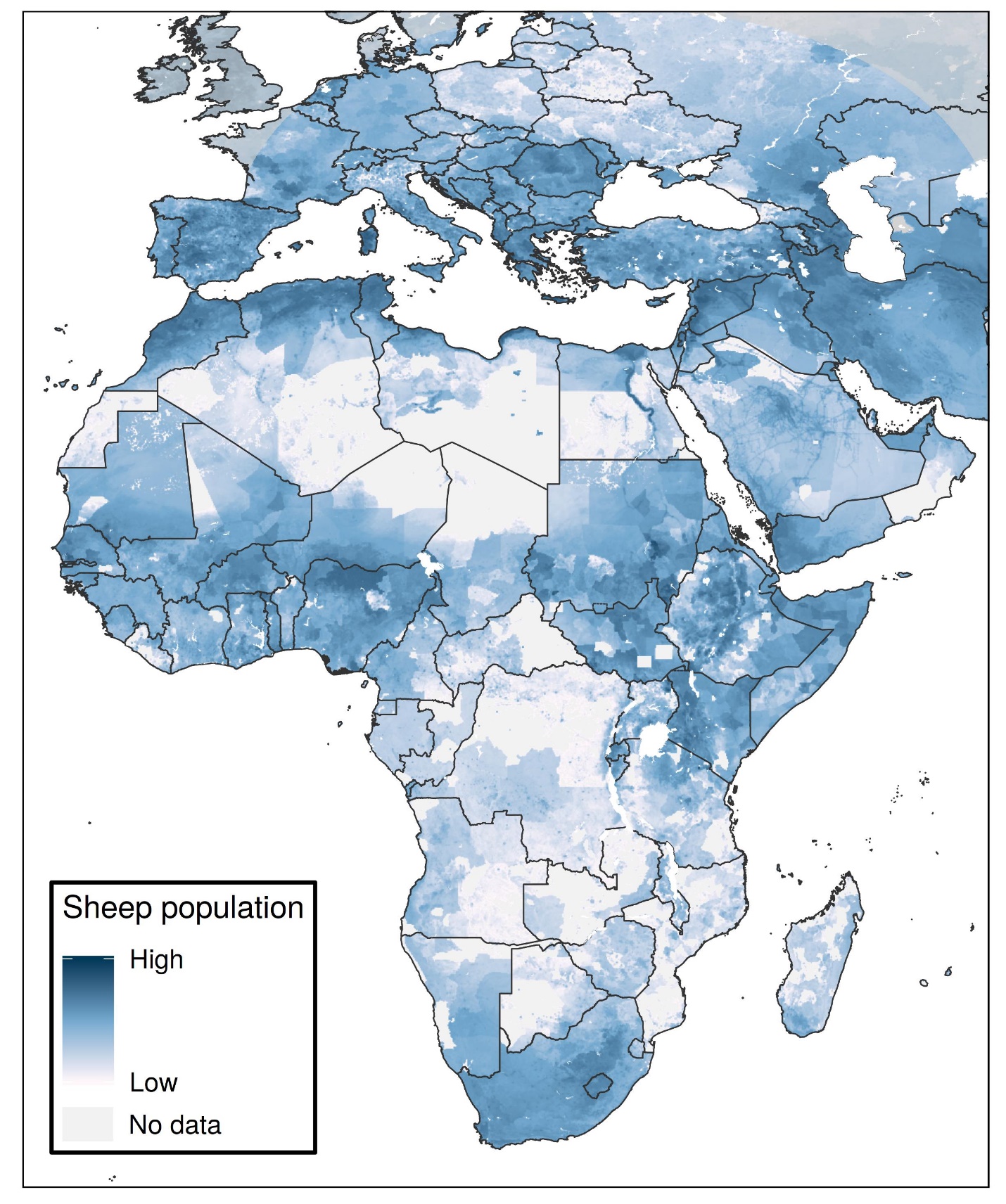


Supplemental Figure 30: Goat population data

Here we show the goat population data that were used as part of our spillover calculation.


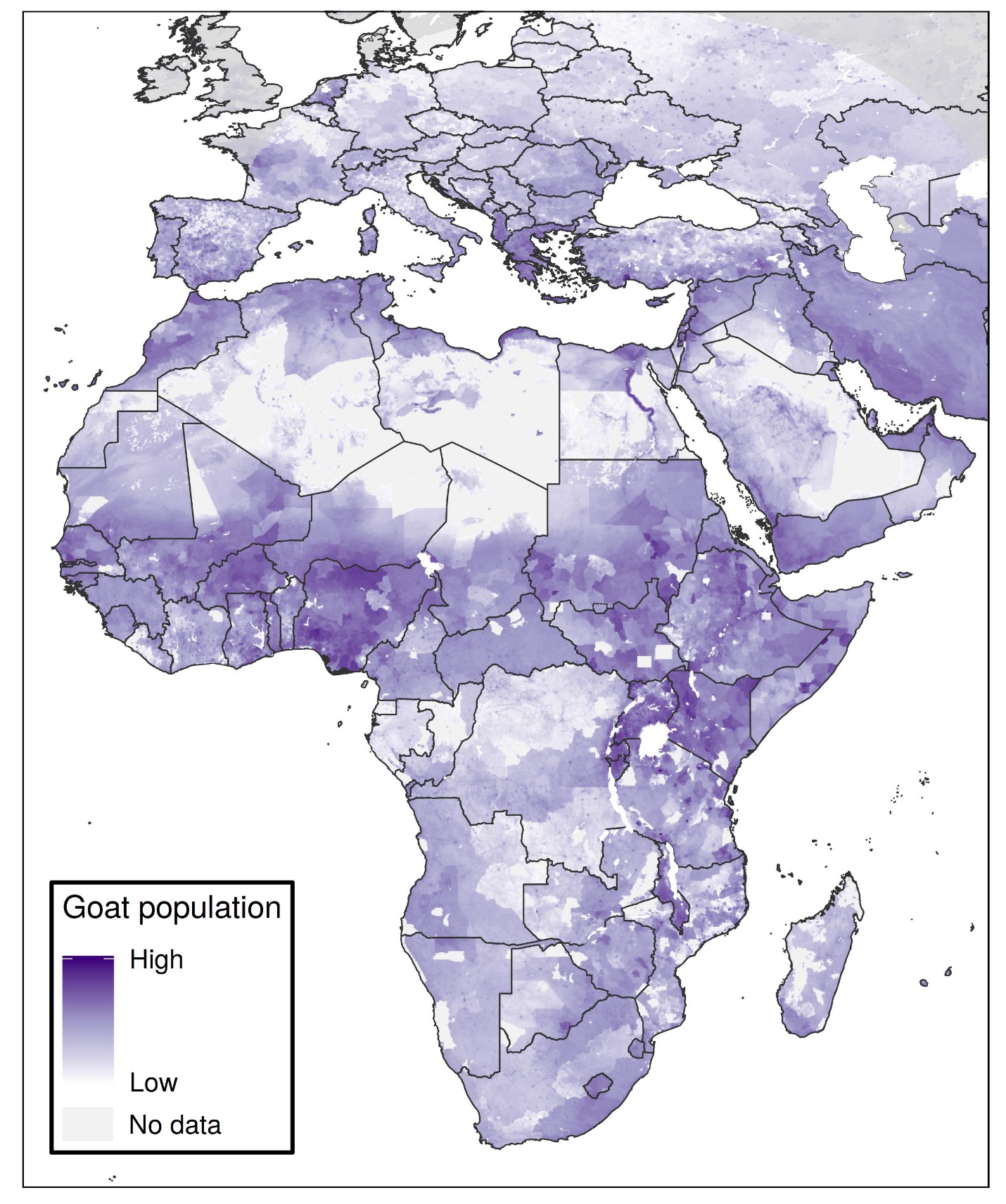


## 4.5 Mean spillover quintile

For each calendar month, we took the average of the spillover quintile value for each district across all years. We then bin these values between 0 and 5 and report this value as a synoptic indicator of spillover potential.

## 4.6 Months per year in top spillover quintile

Since districts in certain months and years with spillover values that fall into the highest quintile could be considered the highest-priority districts for responses, we calculated how many times per year a given district was in the top spillover quintile. Similar to the suitable months per year calculation described in section 4.1, we counted the number of times a district was in the top quintile over all months and years and then divided this number by 22, which was the total number of years included in our analysis, to find the average number of months per year a district was in the top quintile of spillover potential.

# 5.0 Supplementary results

## 5.1 Monthly mean suitability maps

In Figure 3 of the main text, we show mean suitability predictions for January, April, July, and October. Here we show these maps for all 12 months.

Appendix Figure 31: January mean environmental suitability

The predictions shown represent mean environmental suitability predictions for this month averaged across 1995–2016. Areas shown in purple are more suitable than those shown in green.


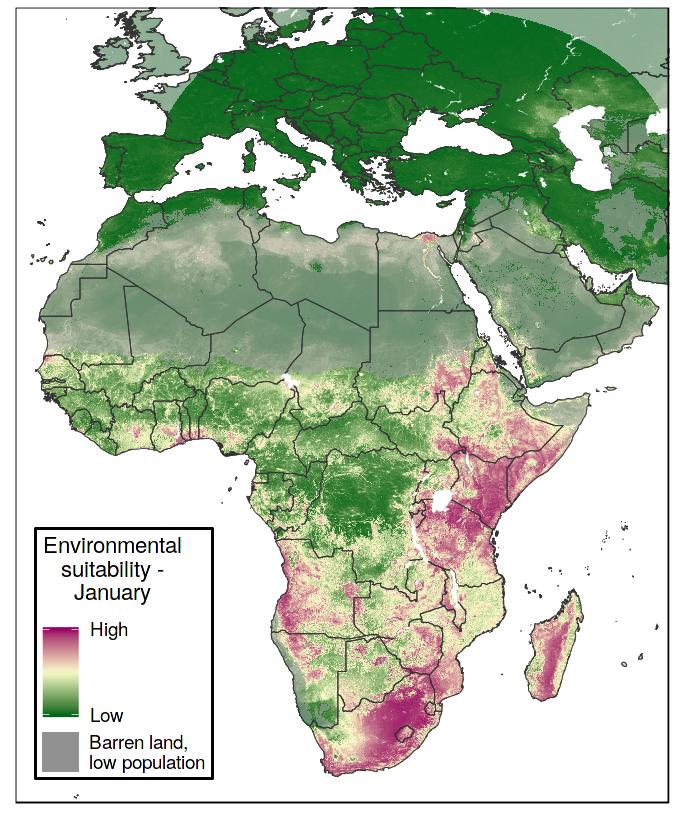


Appendix Figure 32: February mean environmental suitability

The predictions shown represent mean environmental suitability predictions for this month averaged across 1995–2016. Areas shown in purple are more suitable than those shown in green.


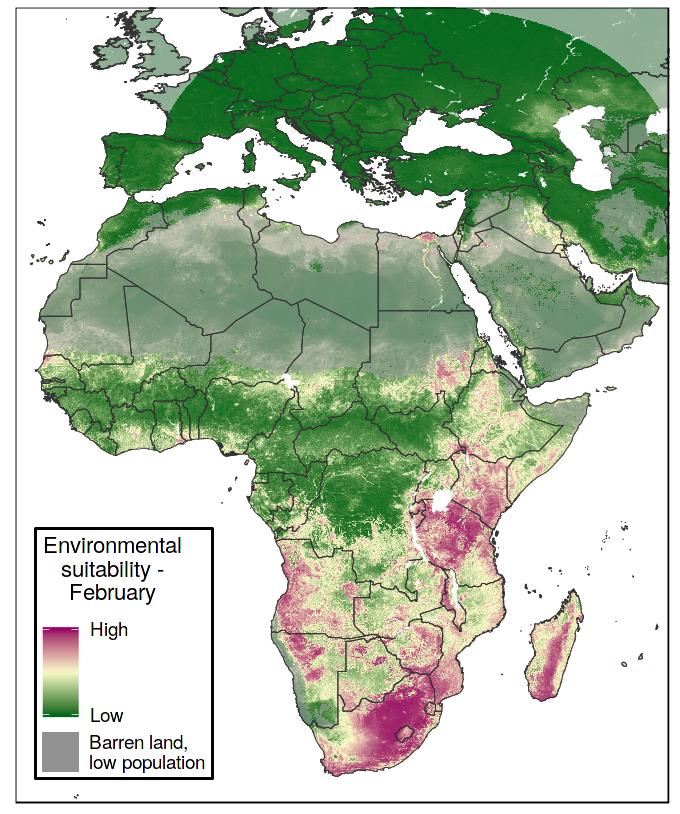


Appendix Figure 33: March mean environmental suitability

The predictions shown represent mean environmental suitability predictions for this month averaged across 1995–2016. Areas shown in purple are more suitable than those shown in green.


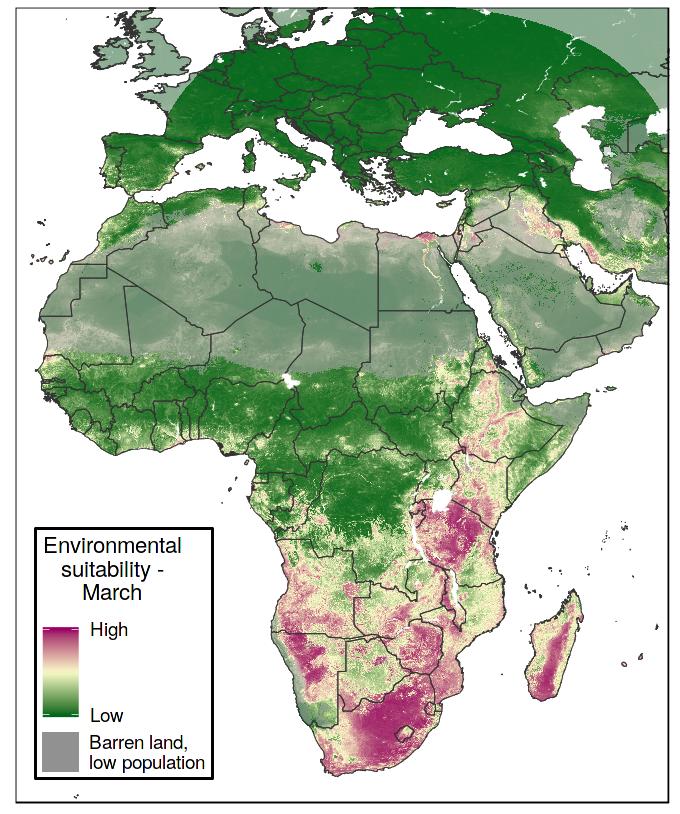


Appendix Figure 34: April mean environmental suitability

The predictions shown represent mean environmental suitability predictions for this month averaged across 1995–2016. Areas shown in purple are more suitable than those shown in green.


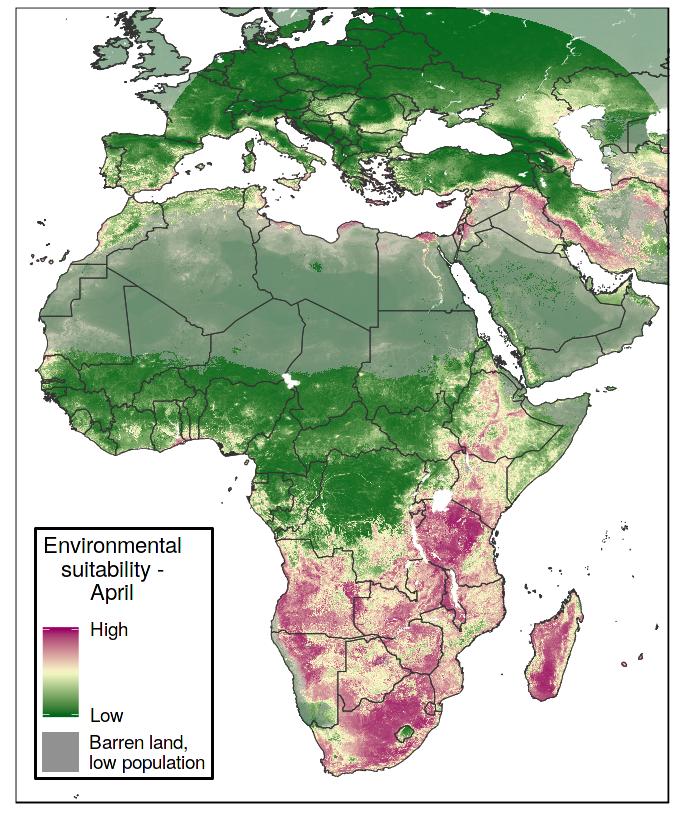


Appendix Figure 35: May mean environmental suitability

The predictions shown represent mean environmental suitability predictions for this month averaged across 1995–2016. Areas shown in purple are more suitable than those shown in green.


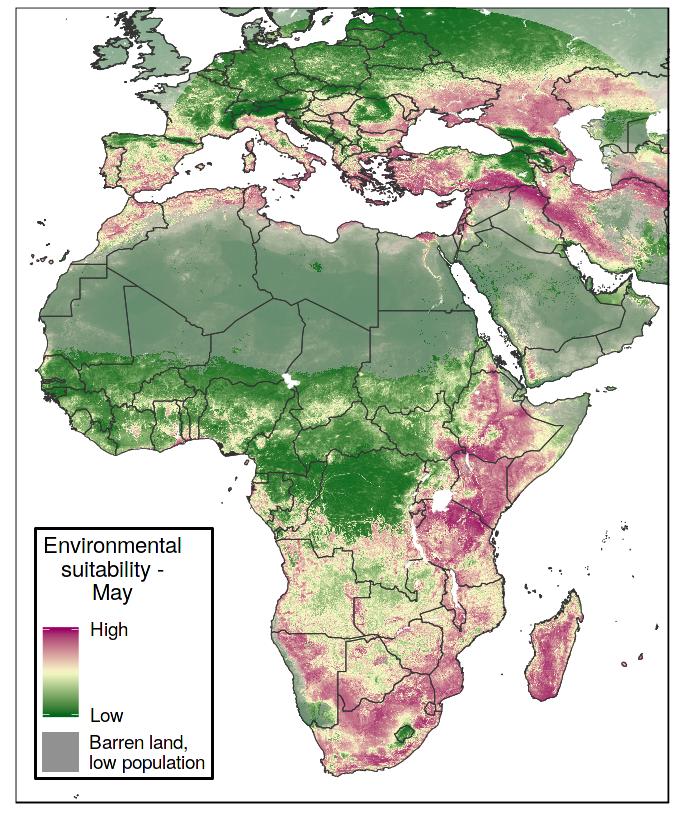


Appendix Figure 36: June mean environmental suitability

The predictions shown represent mean environmental suitability predictions for this month averaged across 1995–2016. Areas shown in purple are more suitable than those shown in green.


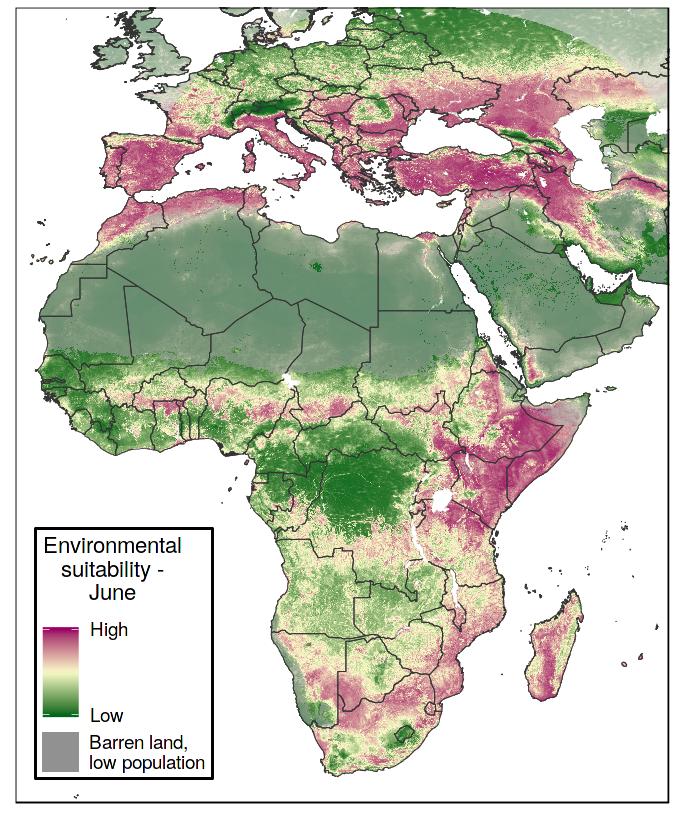


Appendix Figure 37: July mean environmental suitability

The predictions shown represent mean environmental suitability predictions for this month averaged across 1995–2016. Areas shown in purple are more suitable than those shown in green.


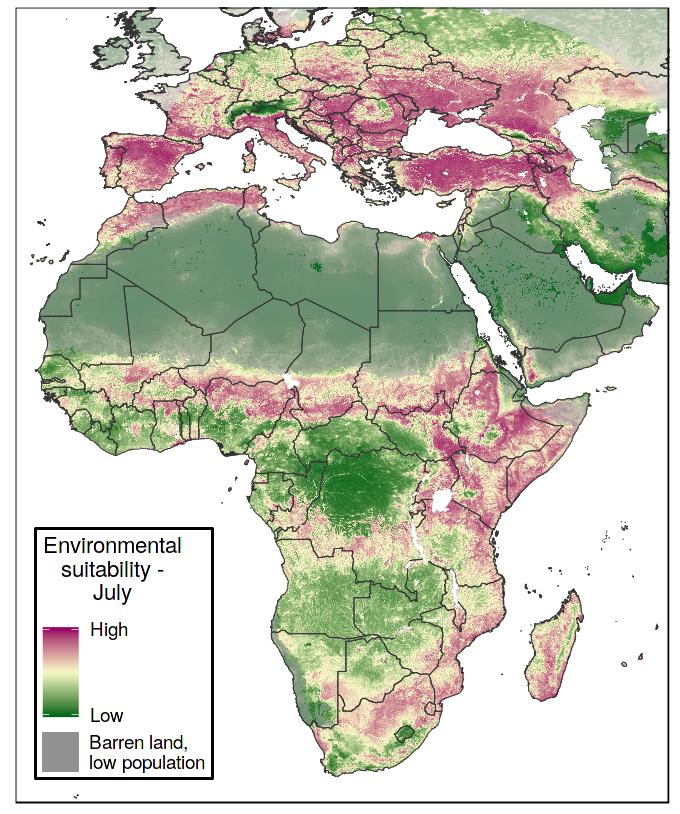


Appendix Figure 38: August mean environmental suitability

The predictions shown represent mean environmental suitability predictions for this month averaged across 1995–2016. Areas shown in purple are more suitable than those shown in green.


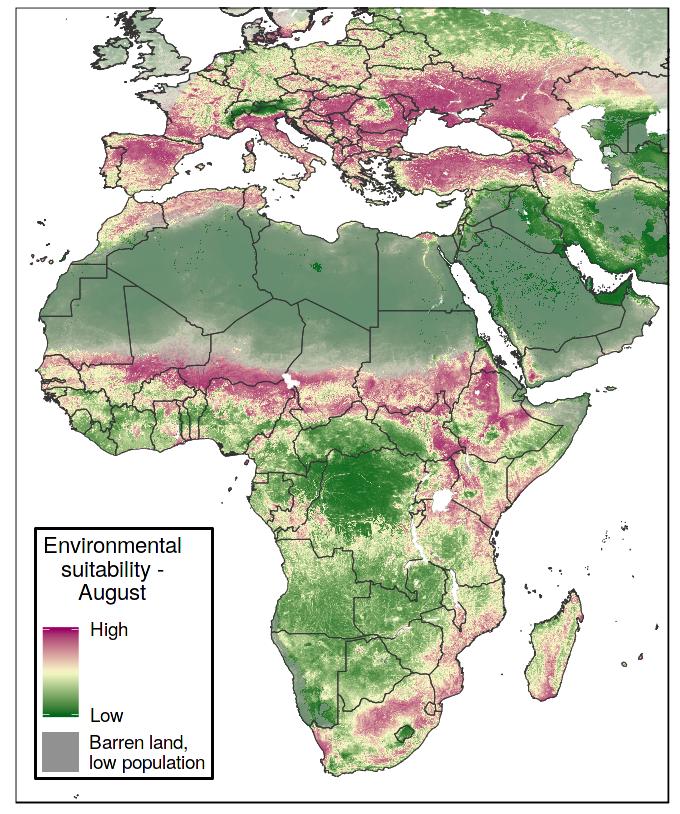


Appendix Figure 39: September mean environmental suitability

The predictions shown represent mean environmental suitability predictions for this month averaged across 1995–2016. Areas shown in purple are more suitable than those shown in green.


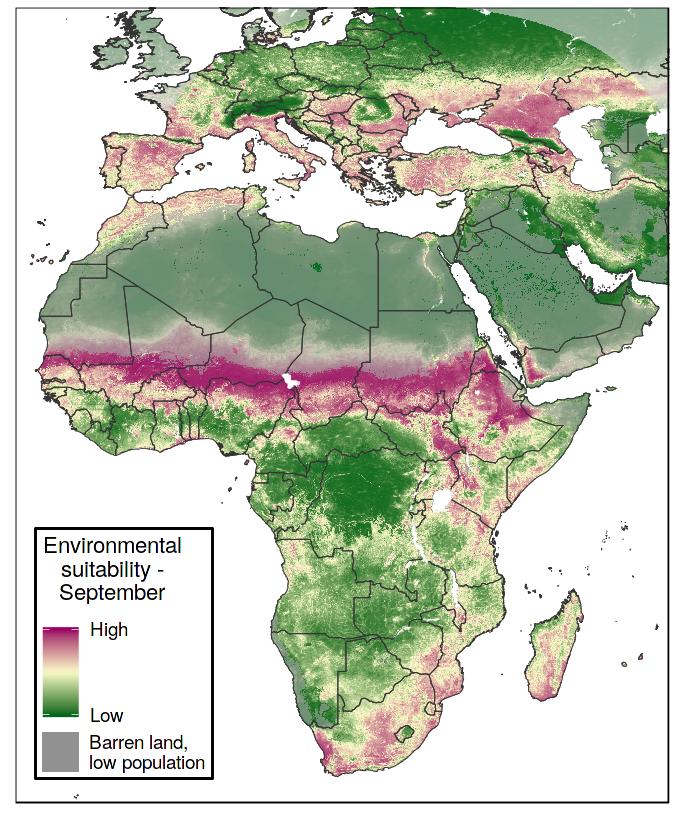


Appendix Figure 40: October mean environmental suitability

The predictions shown represent mean environmental suitability predictions for this month averaged across 1995–2016. Areas shown in purple are more suitable than those shown in green.


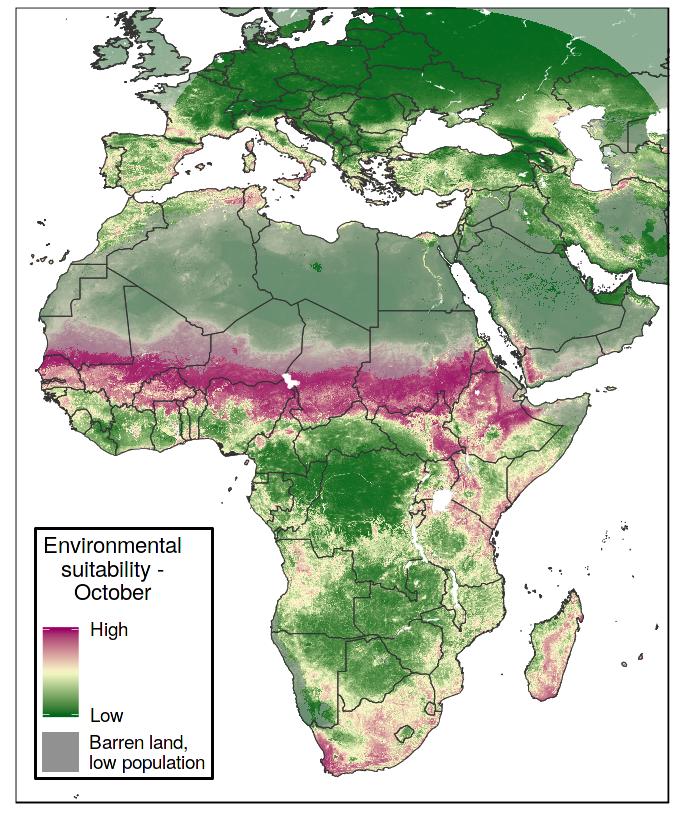


Appendix Figure 41: November mean environmental suitability

The predictions shown represent mean environmental suitability predictions for this month averaged across 1995–2016. Areas shown in purple are more suitable than those shown in green.


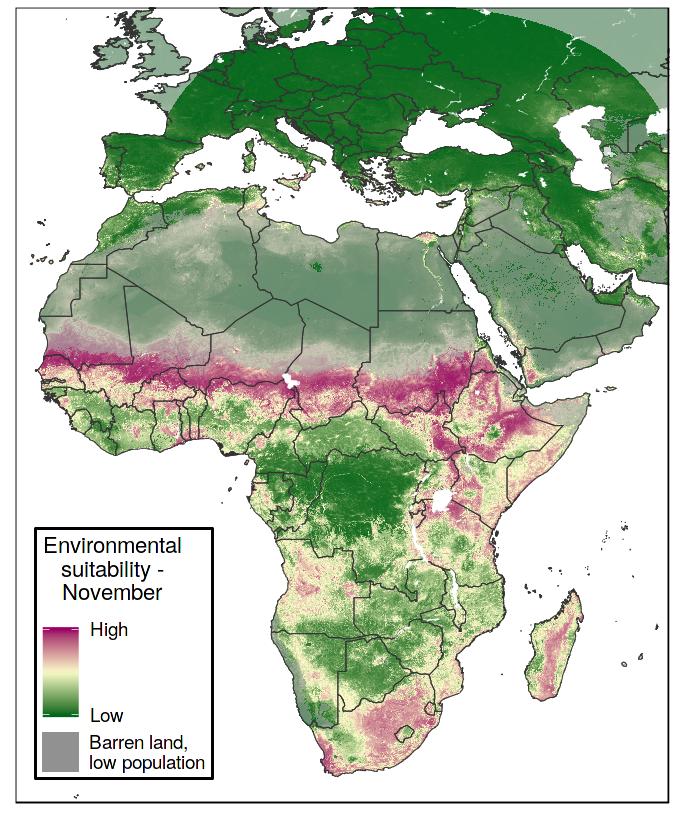


Appendix Figure 42: December mean environmental suitability

The predictions shown represent mean environmental suitability predictions for this month averaged across 1995–2016. Areas shown in purple are more suitable than those shown in green.


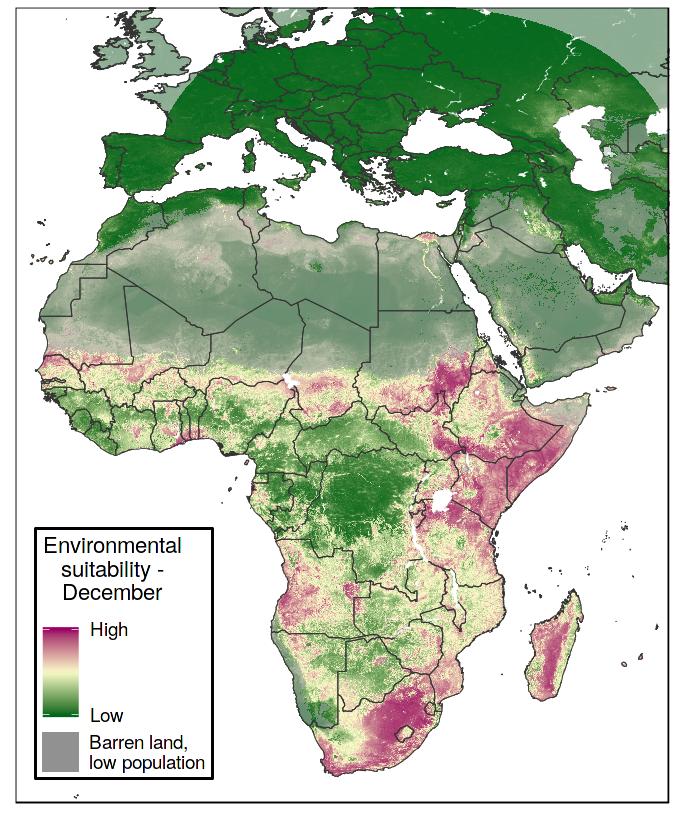


## 5.2 Monthly binary maps

As described in section 4.4 of this appendix, for each month, we converted the continuous predictions shown in section 5.1 into binary predictions. We also converted maps of upper and lower confidence intervals into binary predictions and report them below.

Appendix Figure 43: January binary prediction map with confidence intervals

We used the mean map for this month and all data from this month to calculate an optimised threshold. We used this threshold to turn the continuous predictions shown above into binary ones, where a location could be either suitable or not suitable. Suitable areas are shown in purple.


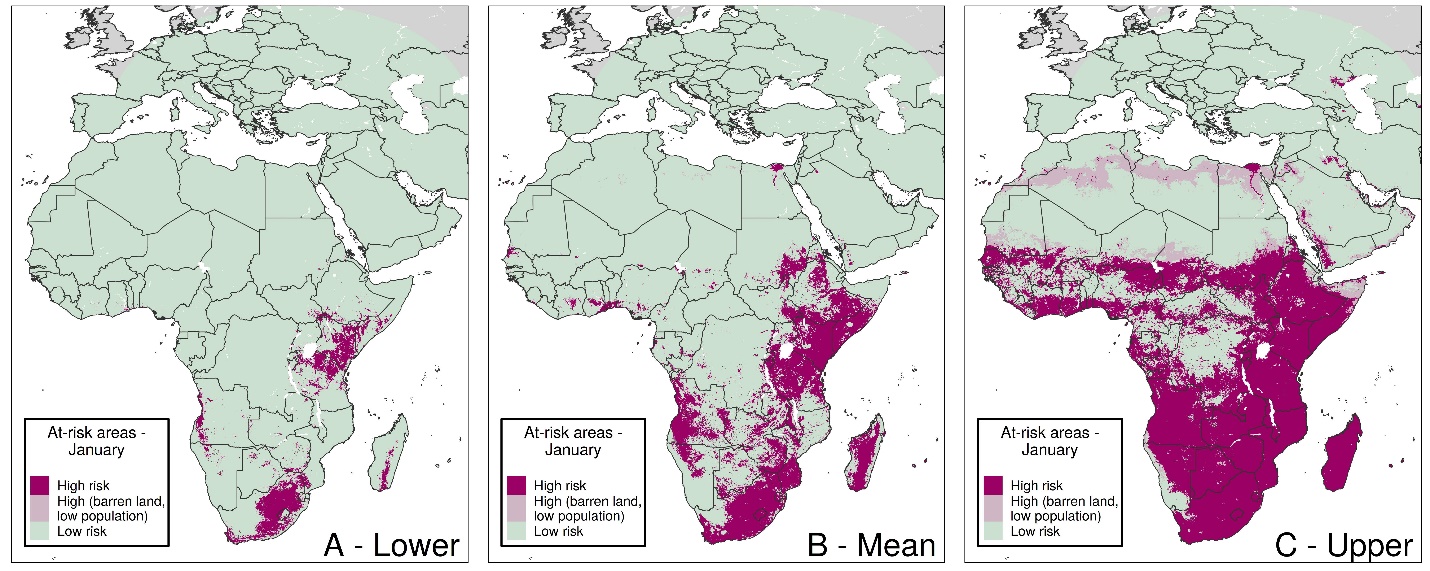


Appendix Figure 44: February binary prediction map with confidence intervals

We used the mean map for this month and all data from this month to calculate an optimised threshold. We used this threshold to turn the continuous predictions shown above into binary ones, where a location could be either suitable or not suitable. Suitable areas are shown in purple.


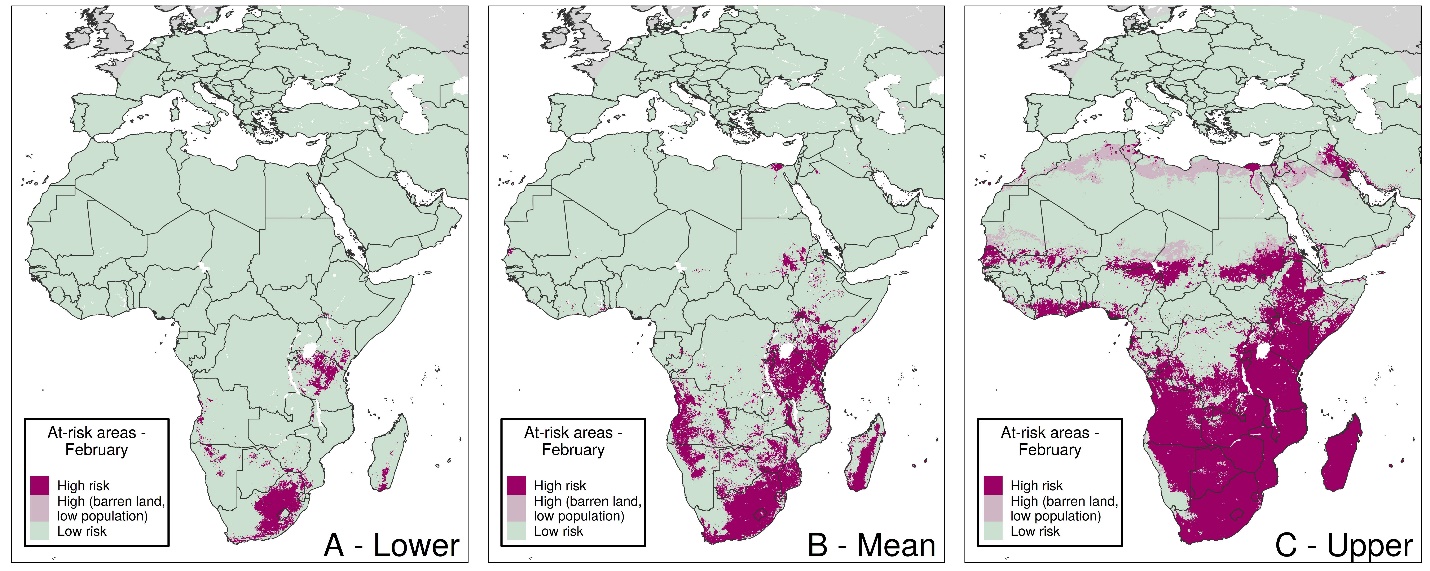


Appendix Figure 45: March binary prediction map with confidence intervals

We used the mean map for this month and all data from this month to calculate an optimised threshold. We used this threshold to turn the continuous predictions shown above into binary ones, where a location could be either suitable or not suitable. Suitable areas are shown in purple.


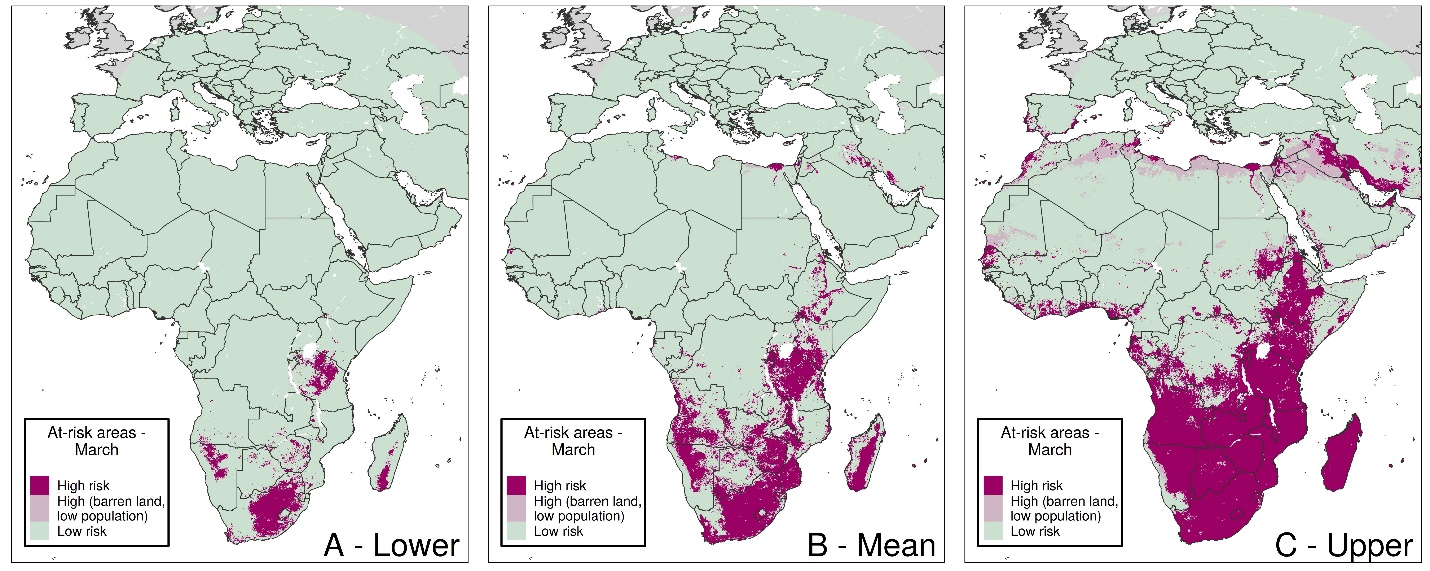


Appendix Figure 46: April binary prediction map with confidence intervals

We used the mean map for this month and all data from this month to calculate an optimised threshold. We used this threshold to turn the continuous predictions shown above into binary ones, where a location could be either suitable or not suitable. Suitable areas are shown in purple.


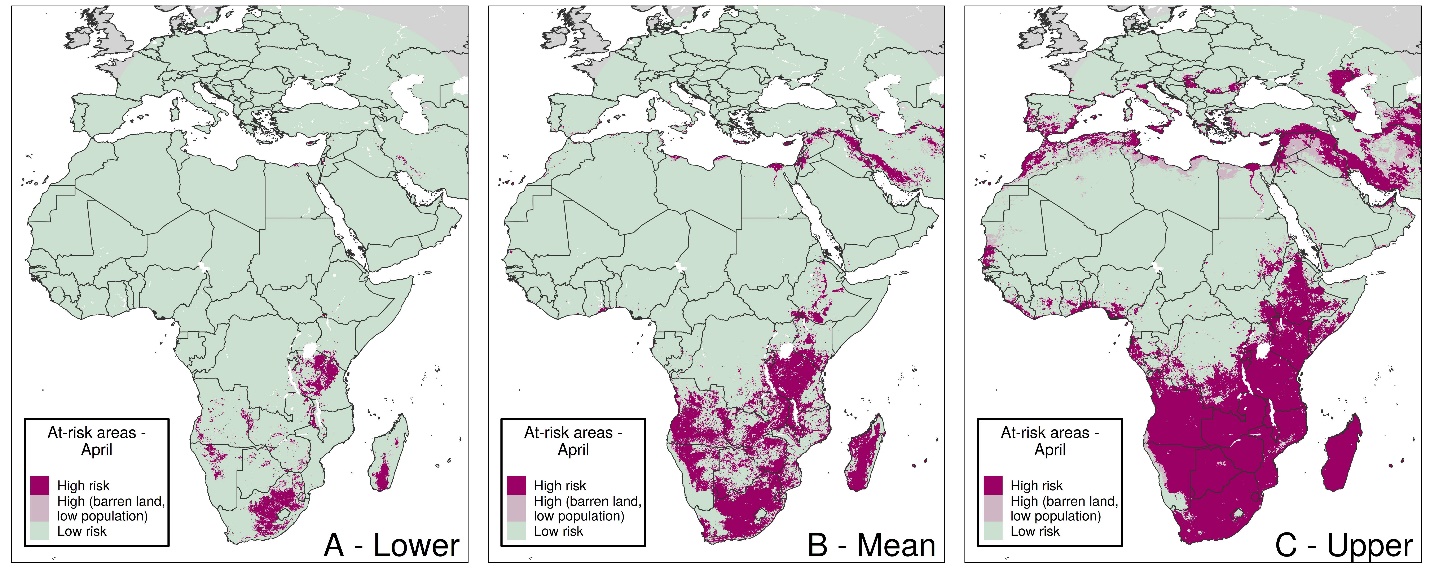


Appendix Figure 47: May binary prediction map with confidence intervals

We used the mean map for this month and all data from this month to calculate an optimised threshold. We used this threshold to turn the continuous predictions shown above into binary ones, where a location could be either suitable or not suitable. Suitable areas are shown in purple.


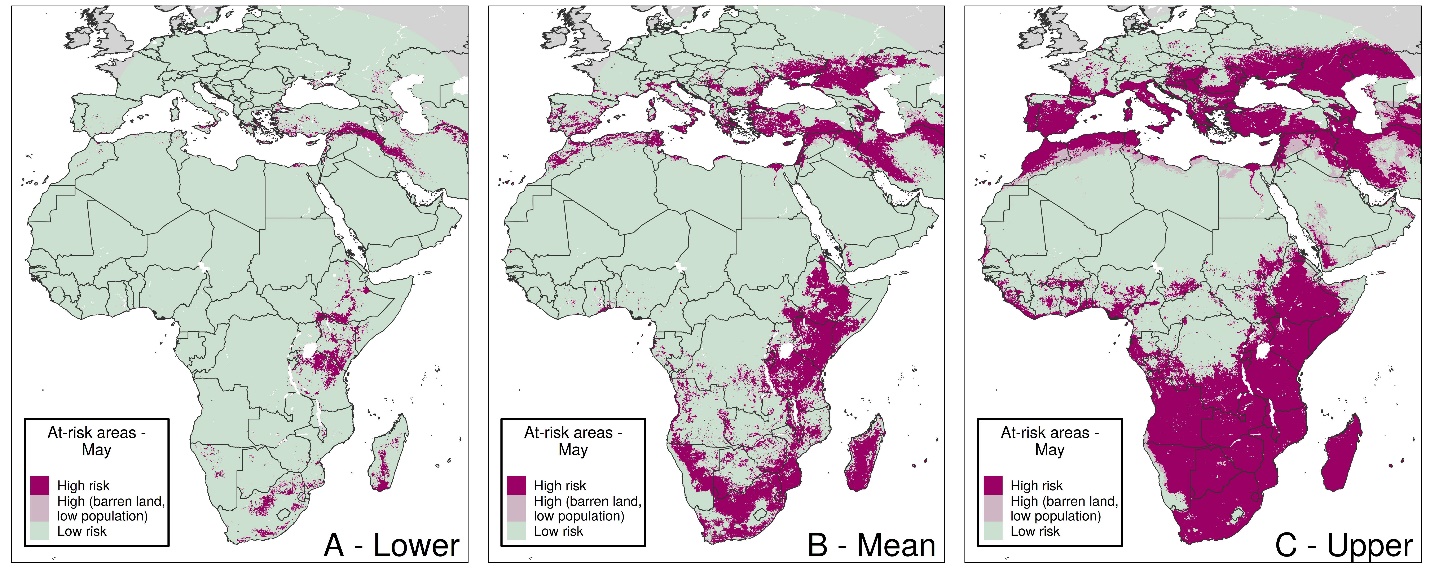


Appendix Figure 48: June binary prediction map with confidence intervals

We used the mean map for this month and all data from this month to calculate an optimised threshold. We used this threshold to turn the continuous predictions shown above into binary ones, where a location could be either suitable or not suitable. Suitable areas are shown in purple.


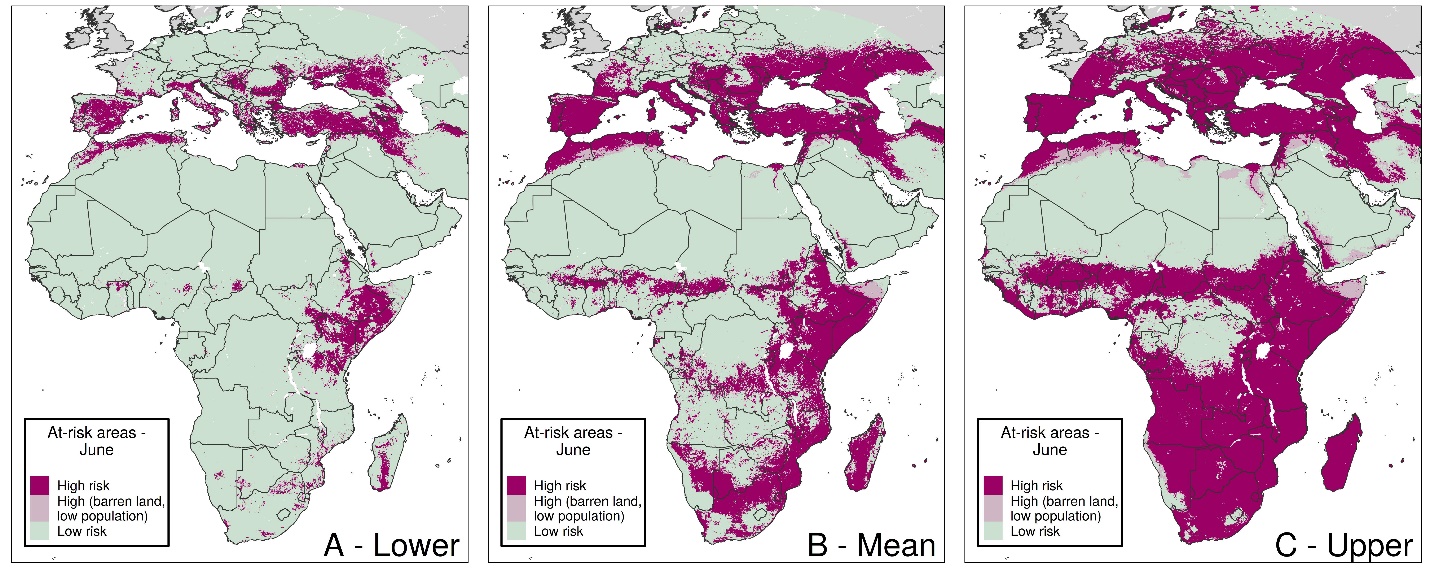


Appendix Figure 49: July binary prediction map with confidence intervals

We used the mean map for this month and all data from this month to calculate an optimised threshold. We used this threshold to turn the continuous predictions shown above into binary ones, where a location could be either suitable or not suitable. Suitable areas are shown in purple.


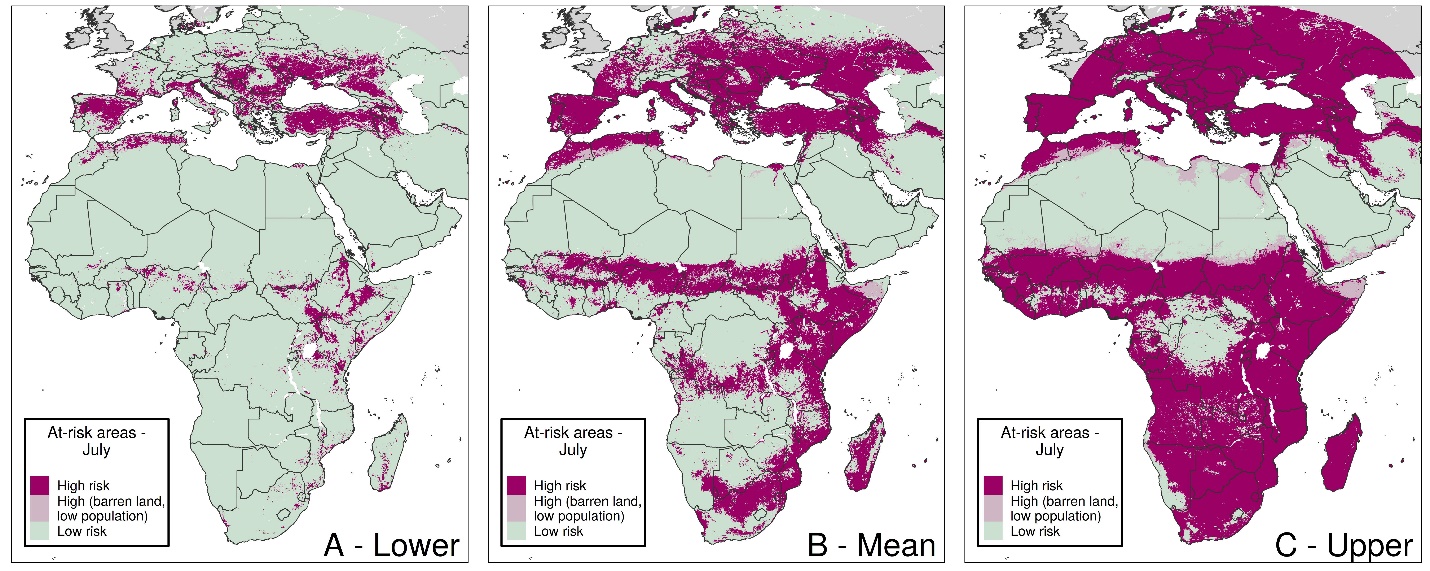


Appendix Figure 50: August binary prediction map with confidence intervals

We used the mean map for this month and all data from this month to calculate an optimised threshold. We used this threshold to turn the continuous predictions shown above into binary ones, where a location could be either suitable or not suitable. Suitable areas are shown in purple.


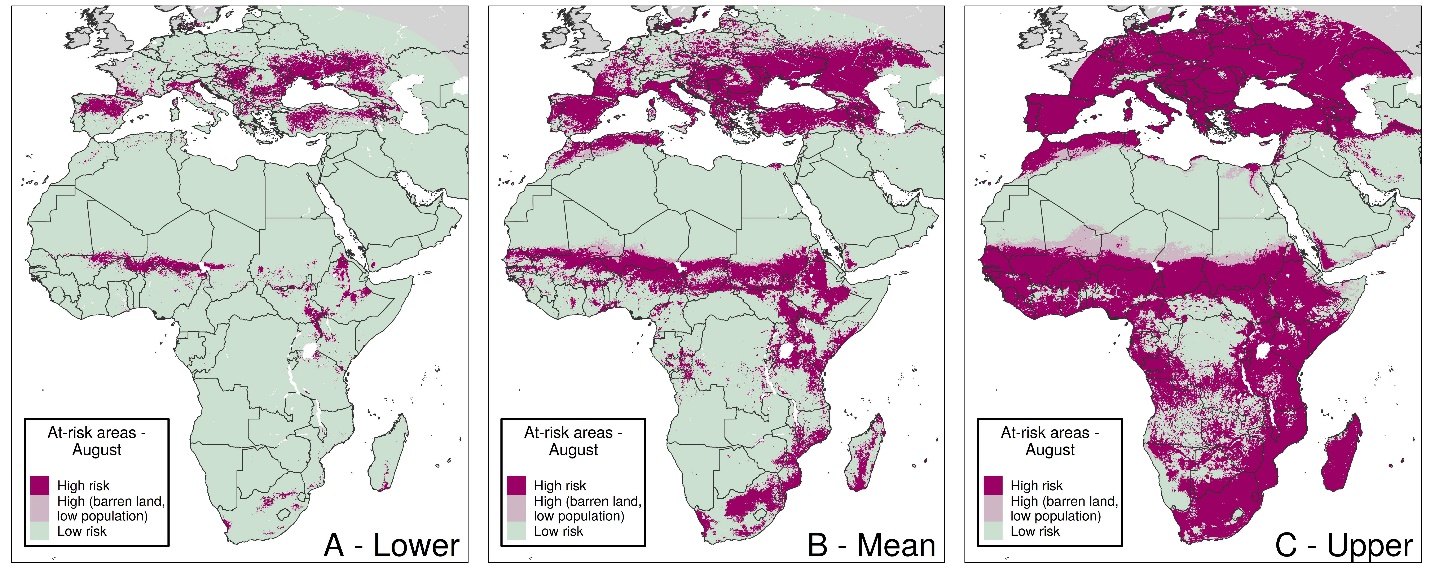


Appendix Figure 51: September binary prediction map with confidence intervals

We used the mean map for this month and all data from this month to calculate an optimised threshold. We used this threshold to turn the continuous predictions shown above into binary ones, where a location could be either suitable or not suitable. Suitable areas are shown in purple.


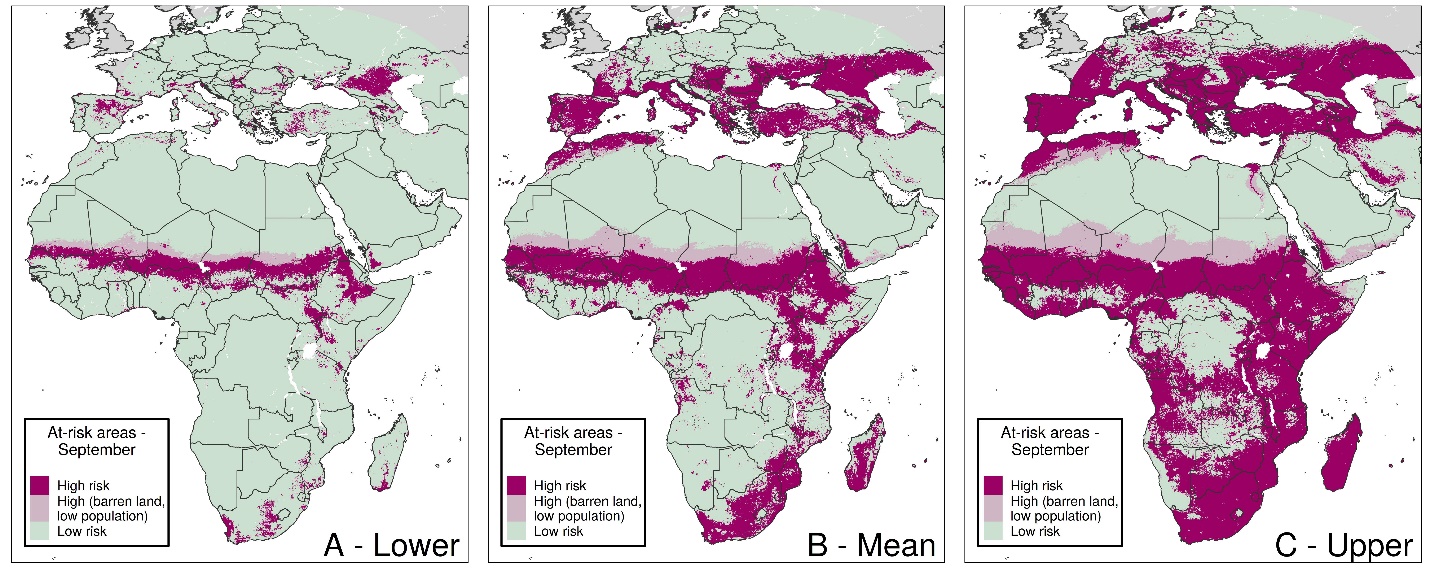


Appendix Figure 52: October binary prediction map with confidence intervals

We used the mean map for this month and all data from this month to calculate an optimised threshold. We used this threshold to turn the continuous predictions shown above into binary ones, where a location could be either suitable or not suitable. Suitable areas are shown in purple.


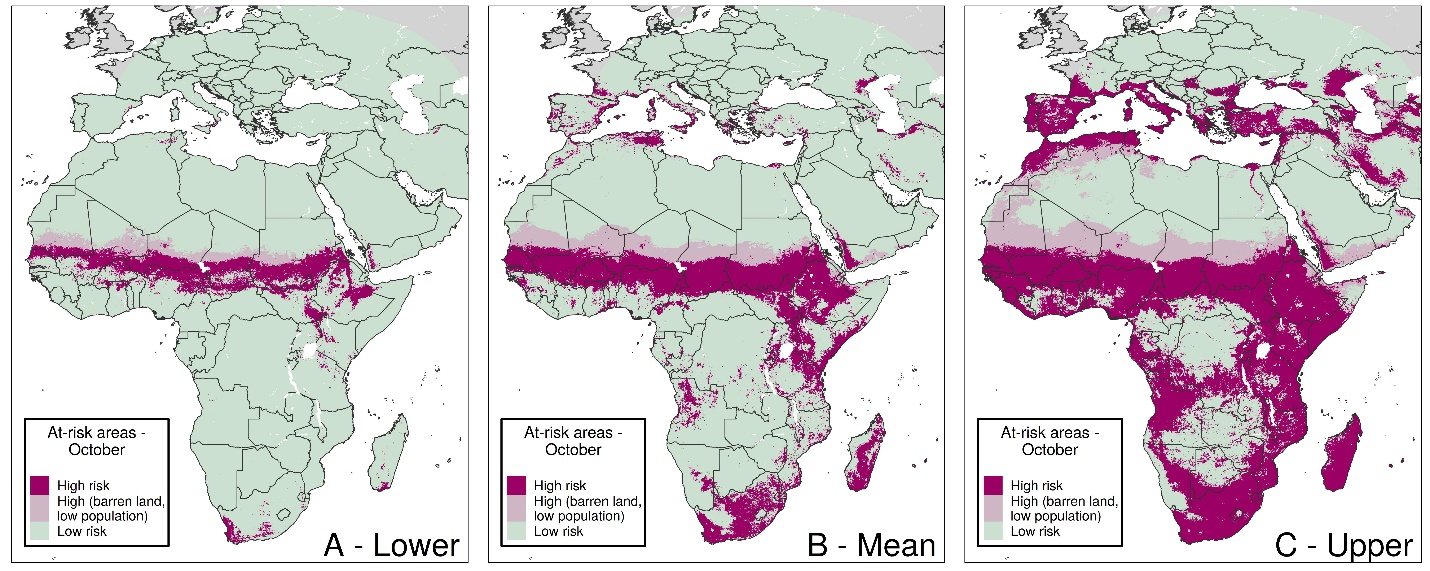


Appendix Figure 53: November binary prediction map with confidence intervals

We used the mean map for this month and all data from this month to calculate an optimised threshold. We used this threshold to turn the continuous predictions shown above into binary ones, where a location could be either suitable or not suitable. Suitable areas are shown in purple.


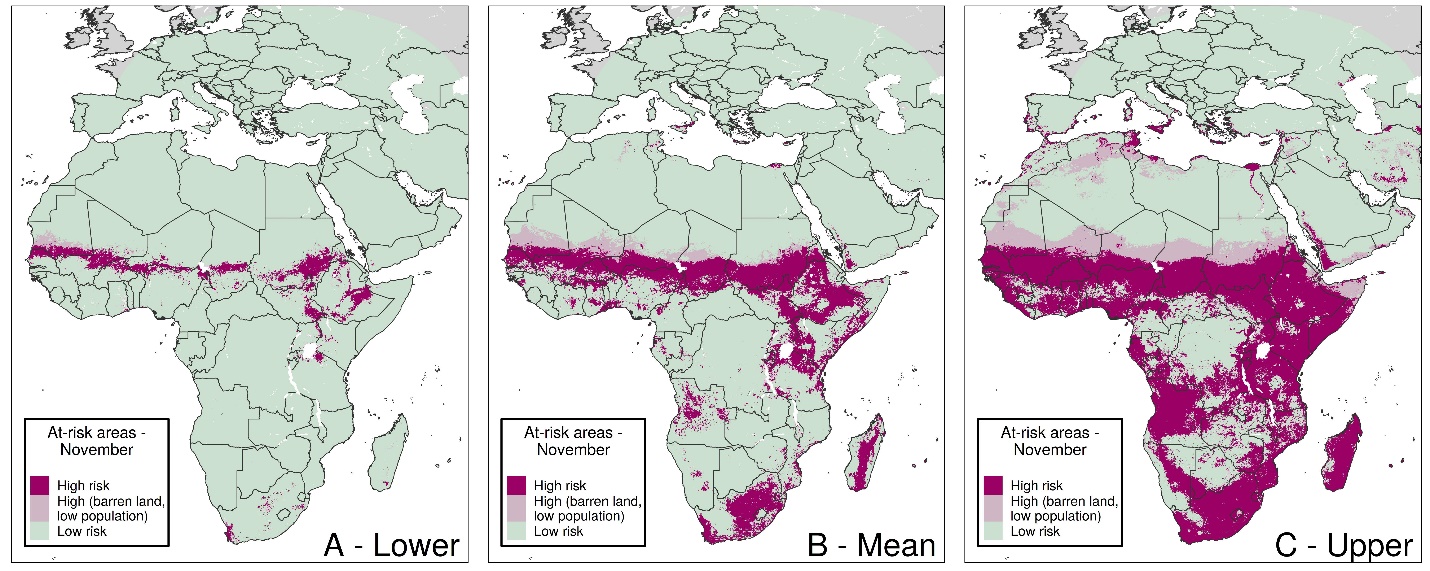


Appendix Figure 54: December binary prediction map with confidence intervals

We used the mean map for this month and all data from this month to calculate an optimised threshold. We used this threshold to turn the continuous predictions shown above into binary ones, where a location could be either suitable or not suitable. Suitable areas are shown in purple.


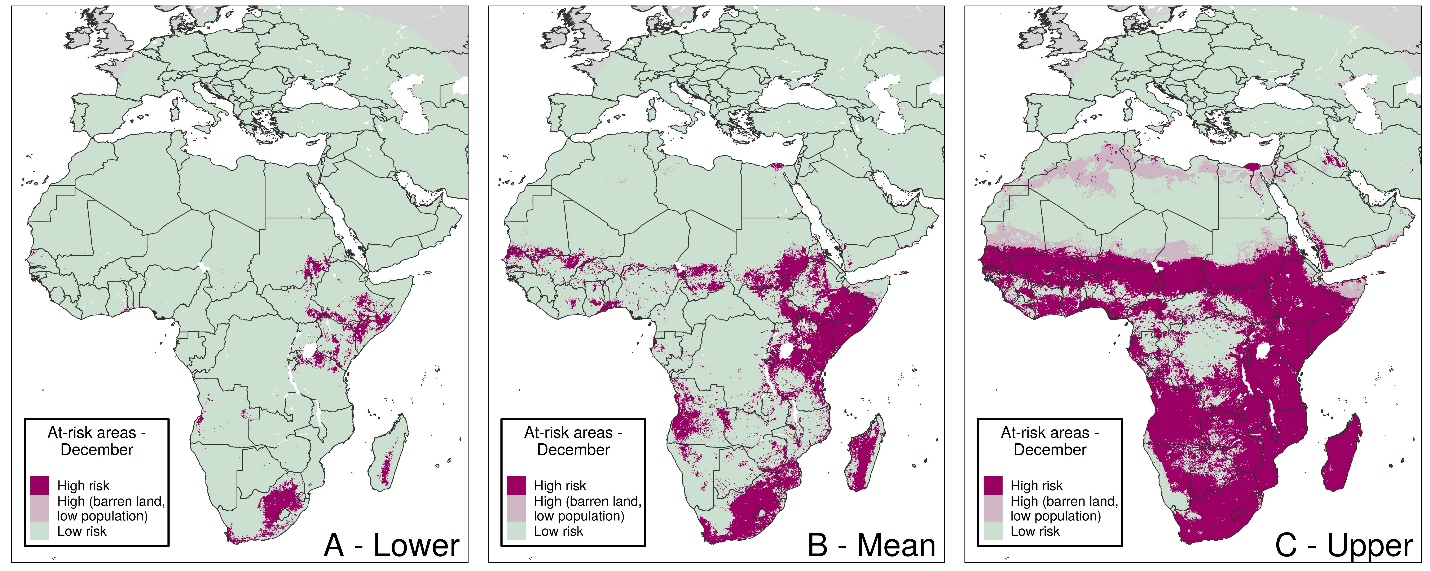


## 5.3 Monthly mean uncertainty maps

We calculated uncertainty for each month-year combination as the difference between percentiles 2.5 and 97.5 of the distribution of the 100 bootstrap predictions of suitability. We report the mean of this difference across all years as monthly uncertainty.

Appendix Figure 55: January mean uncertainty map

Here we show the difference between the upper and lower confidence intervals of suitability predictions for this month, averaged across all years. Red indicates higher uncertainty, and blue indicates lower.


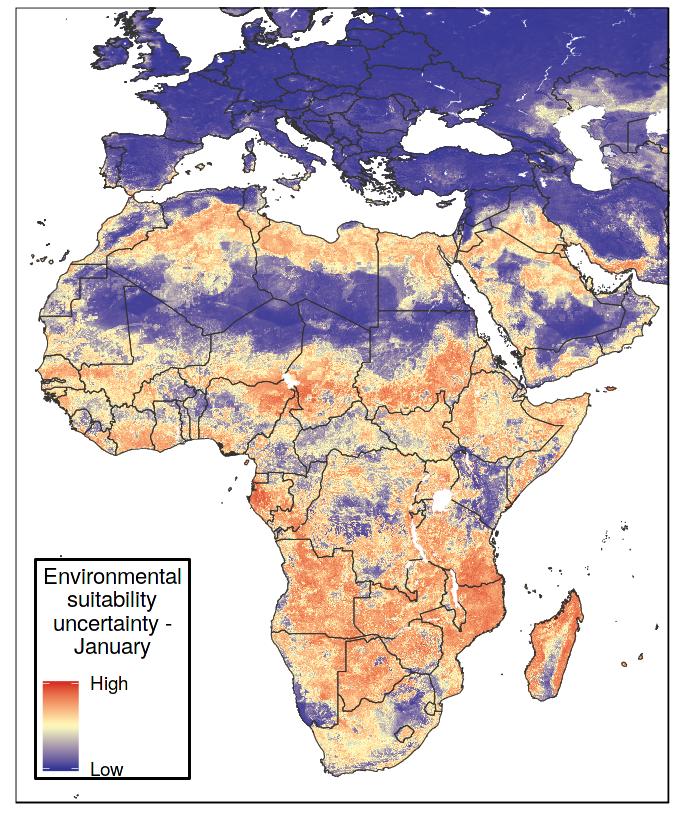


Appendix Figure 56: February mean uncertainty map

Here we show the difference between the upper and lower confidence intervals of suitability predictions for this month, averaged across all years. Red indicates higher uncertainty, and blue indicates lower.


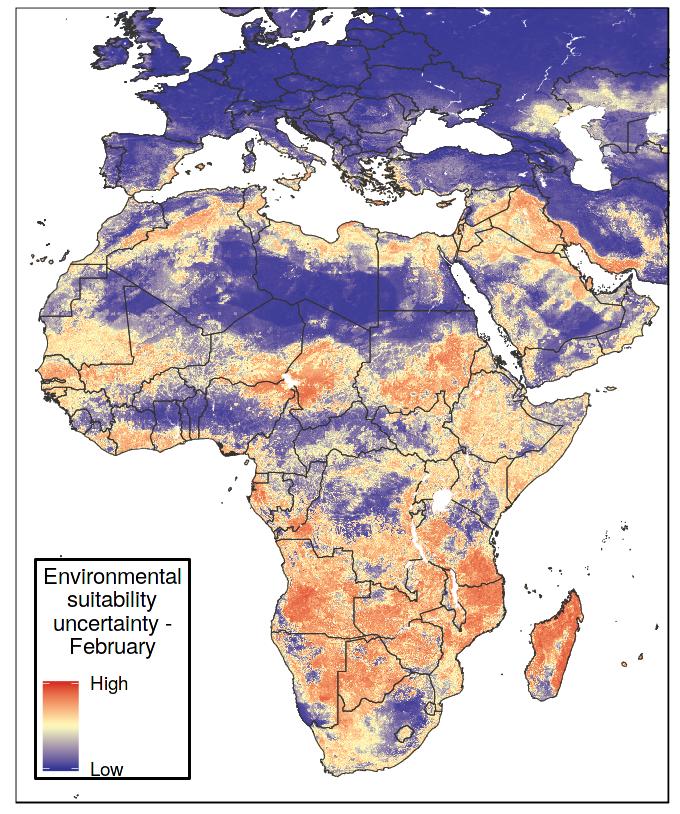


Appendix Figure 57: March mean uncertainty map

Here we show the difference between the upper and lower confidence intervals of suitability predictions for this month, averaged across all years. Red indicates higher uncertainty, and blue indicates lower.


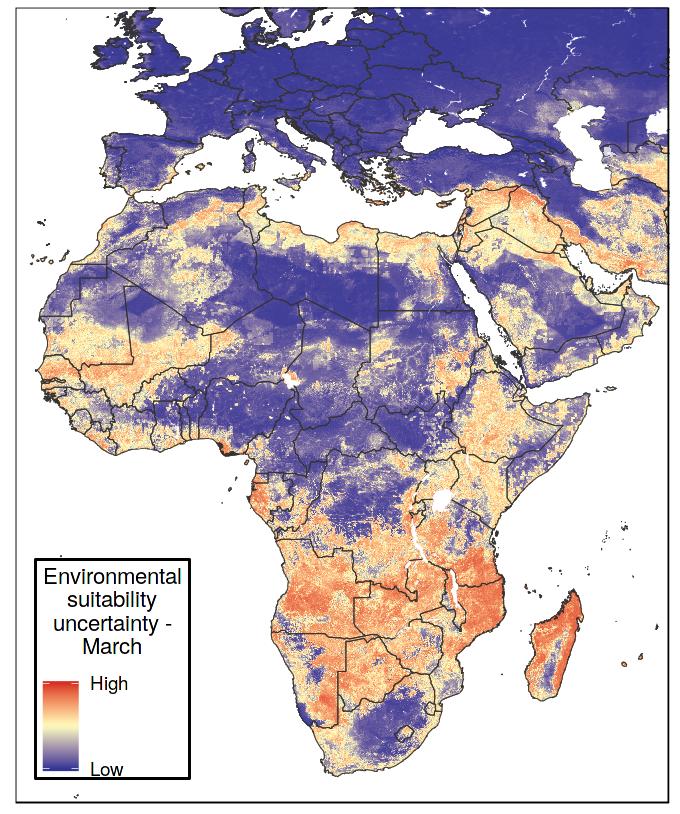


Appendix Figure 58: April mean uncertainty map

Here we show the difference between the upper and lower confidence intervals of suitability predictions for this month, averaged across all years. Red indicates higher uncertainty, and blue indicates lower.


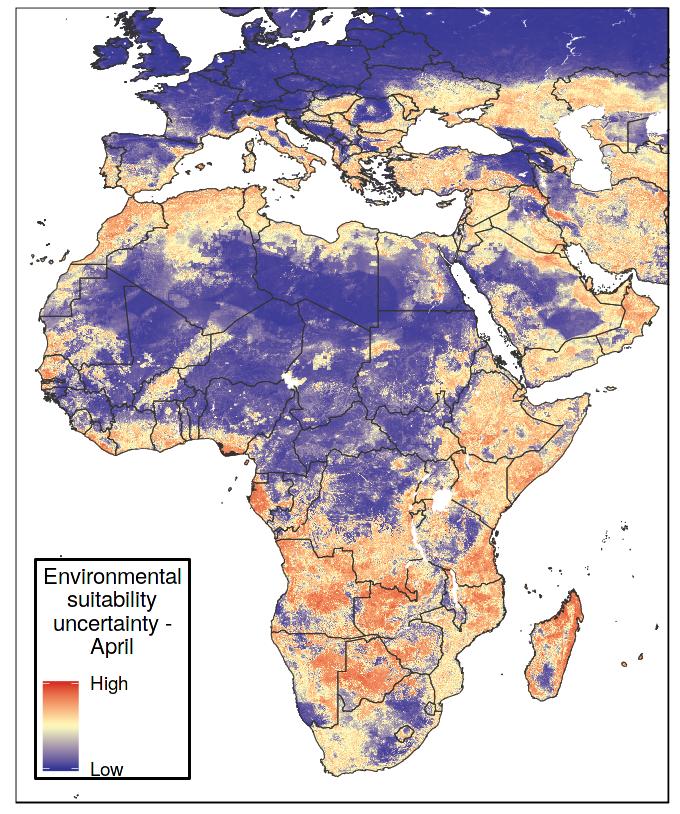


Appendix Figure 59: May mean uncertainty map

Here we show the difference between the upper and lower confidence intervals of suitability predictions for this month, averaged across all years. Red indicates higher uncertainty, and blue indicates lower.
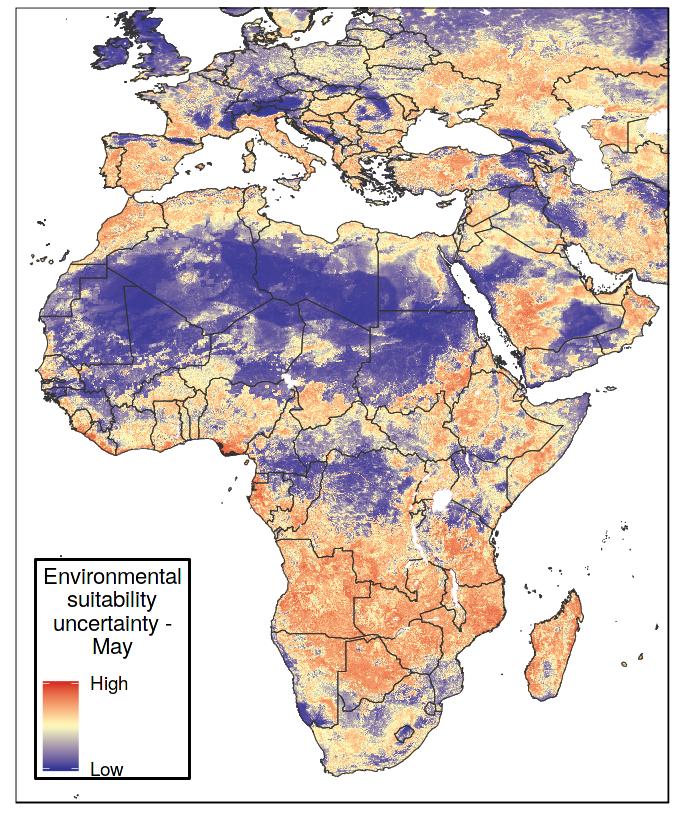


Appendix Figure 60: June mean uncertainty map

Here we show the difference between the upper and lower confidence intervals of suitability predictions for this month, averaged across all years. Red indicates higher uncertainty, and blue indicates lower.


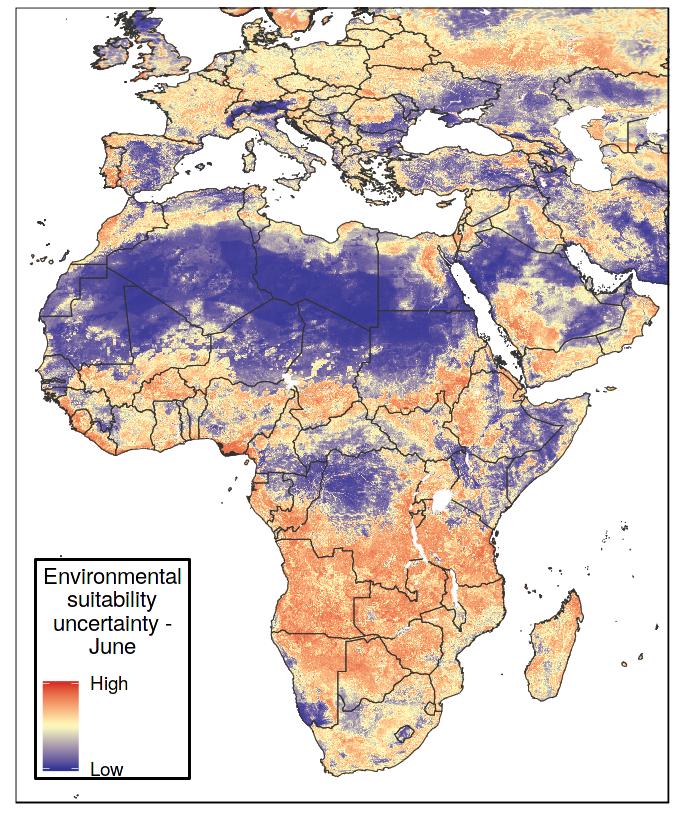


Appendix Figure 61: July mean uncertainty map

Here we show the difference between the upper and lower confidence intervals of suitability predictions for this month, averaged across all years. Red indicates higher uncertainty, and blue indicates lower.


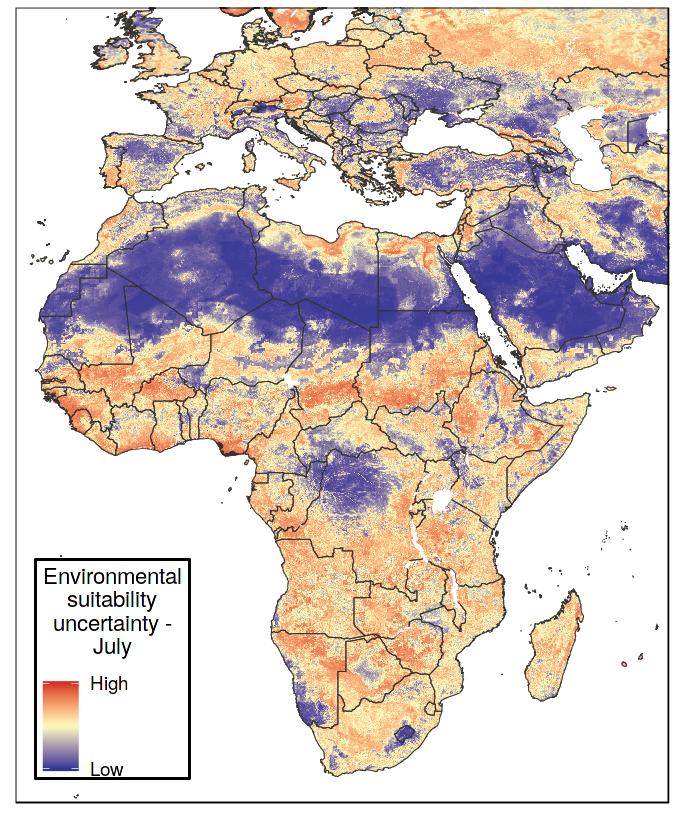


Appendix Figure 62: August mean uncertainty map

Here we show the difference between the upper and lower confidence intervals of suitability predictions for this month, averaged across all years. Red indicates higher uncertainty, and blue indicates lower.


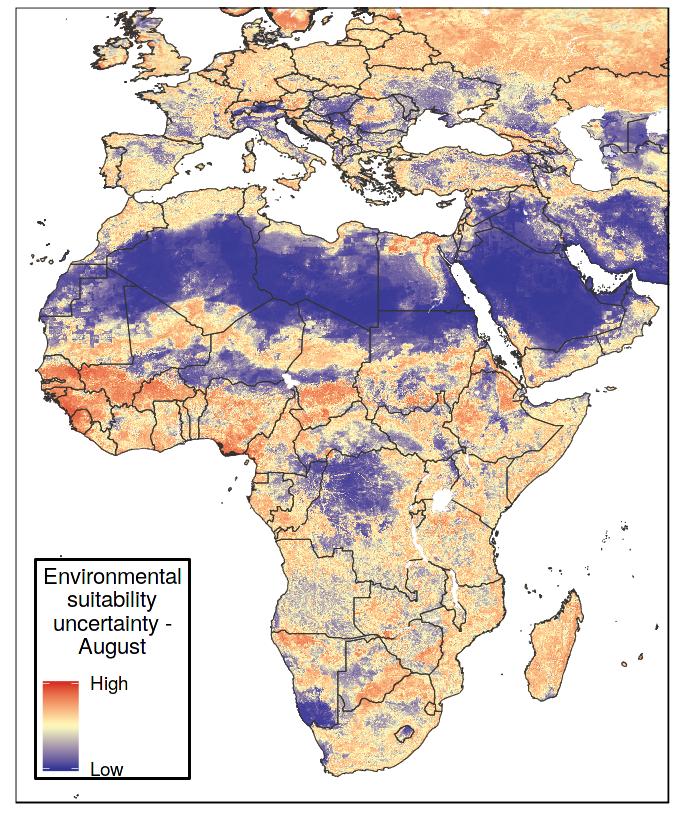


Appendix Figure 63: September mean uncertainty map

Here we show the difference between the upper and lower confidence intervals of suitability predictions for this month, averaged across all years. Red indicates higher uncertainty, and blue indicates lower.


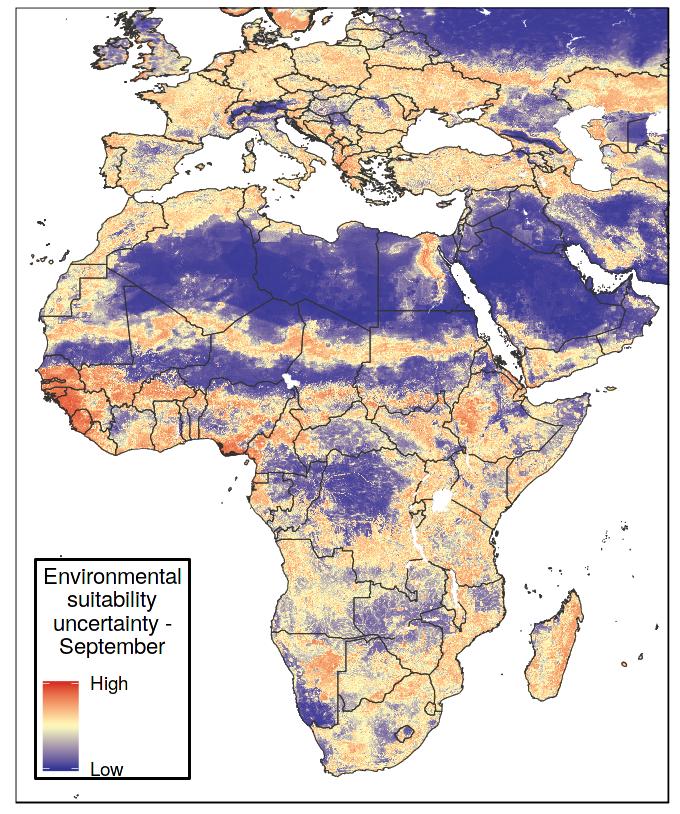


Appendix Figure 64: October mean uncertainty map

Here we show the difference between the upper and lower confidence intervals of suitability predictions for this month, averaged across all years. Red indicates higher uncertainty, and blue indicates lower.
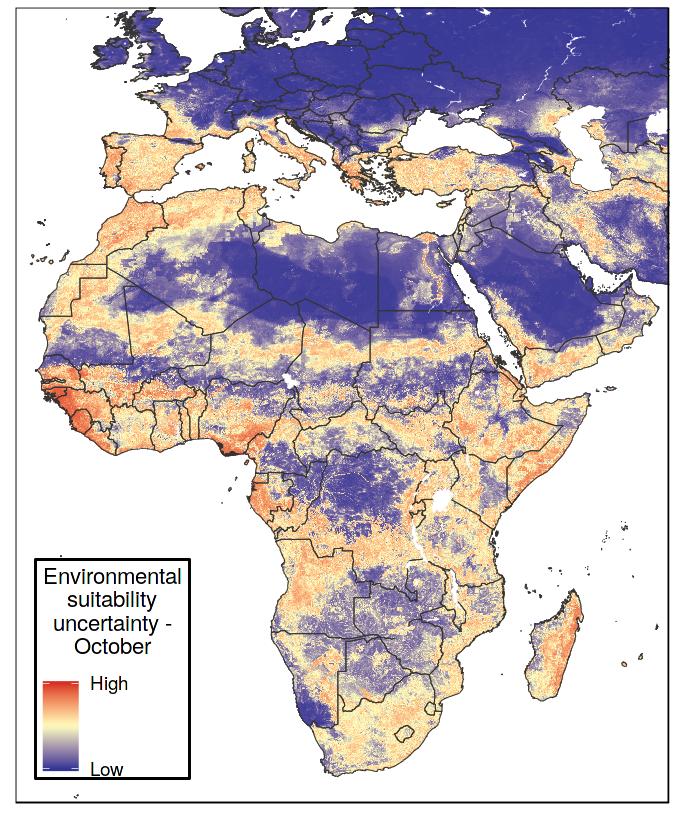


Appendix Figure 65: November mean uncertainty map

Here we show the difference between the upper and lower confidence intervals of suitability predictions for this month, averaged across all years. Red indicates higher uncertainty, and blue indicates lower.


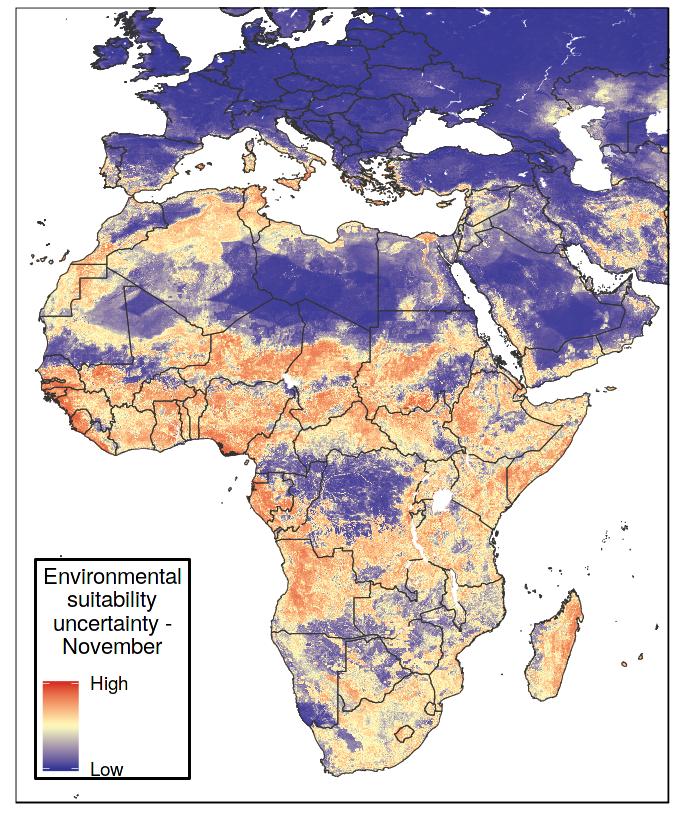


Appendix Figure 66: December mean uncertainty map

Here we show the difference between the upper and lower confidence intervals of suitability predictions for this month, averaged across all years. Red indicates higher uncertainty, and blue indicates lower.


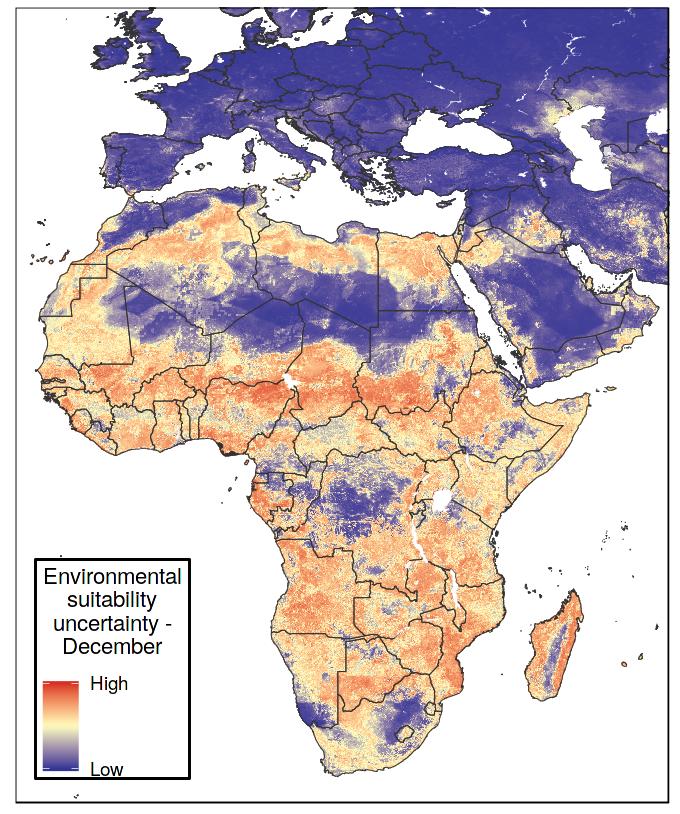


## 5.4 Monthly spillover maps

As described in section 4.5, we calculated spillover potential for each district in each month and year, then ranked all values across all districts, months, and years and binned these values into quintiles.

Appendix Figure 67: January mean spillover map

Each district’s average spillover quintile in this month across all years is shown, where dark purple indicates the highest average quintile.


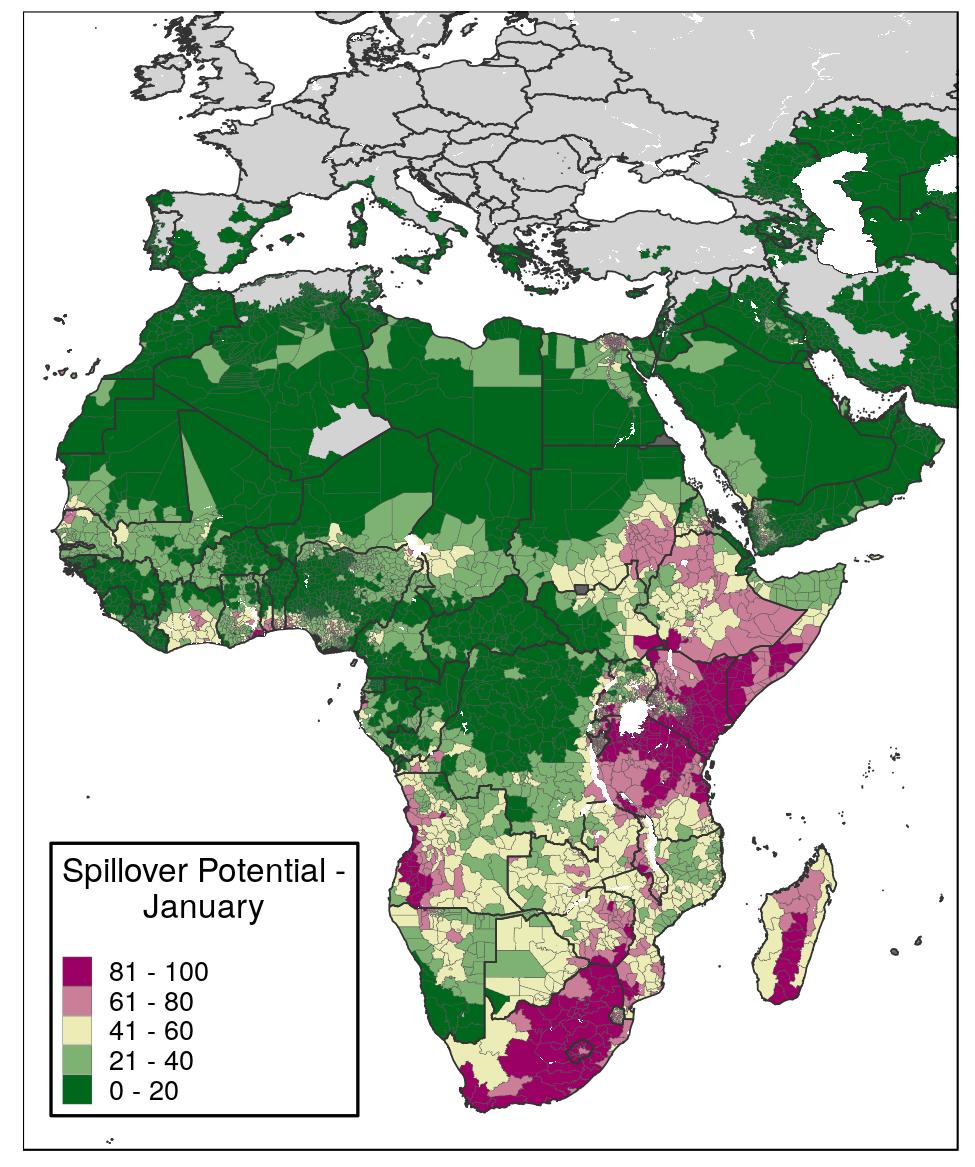


Appendix Figure 68: February mean spillover map

Each district’s average spillover quintile in this month across all years is shown, where dark purple indicates the highest average quintile.
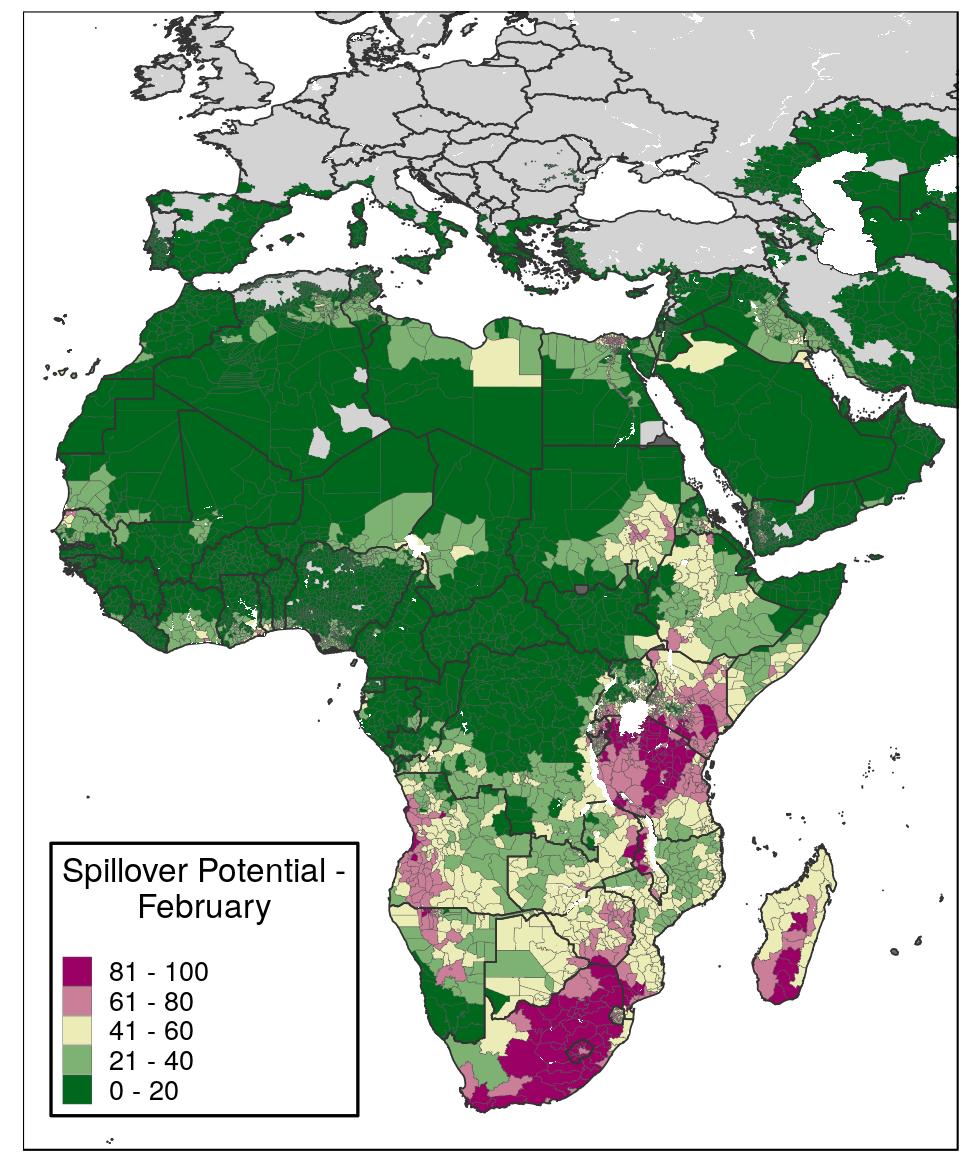


Appendix Figure 69: March mean spillover map

Each district’s average spillover quintile in this month across all years is shown, where dark purple indicates the highest average quintile.


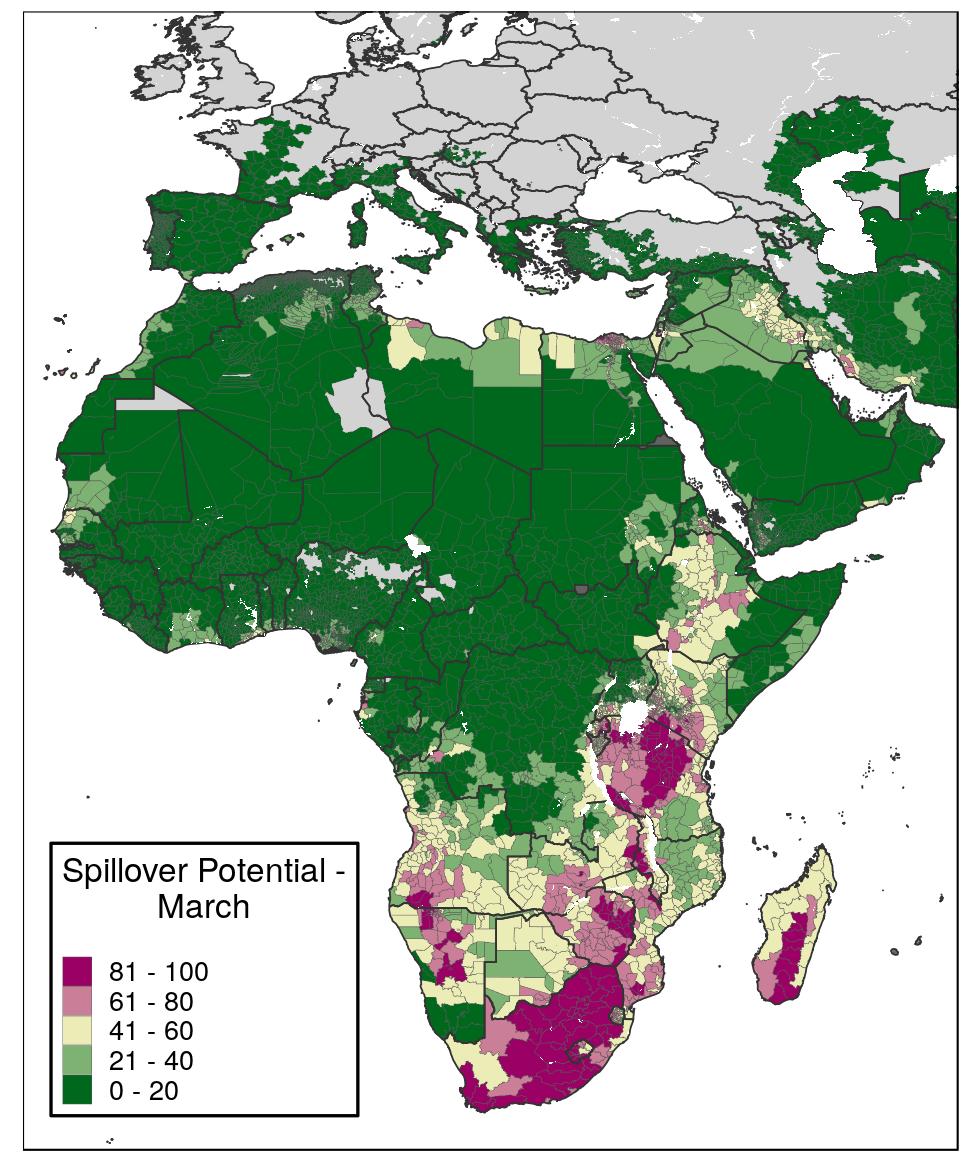


Appendix Figure 70: April mean spillover map

Each district’s average spillover quintile in this month across all years is shown, where dark purple indicates the highest average quintile.


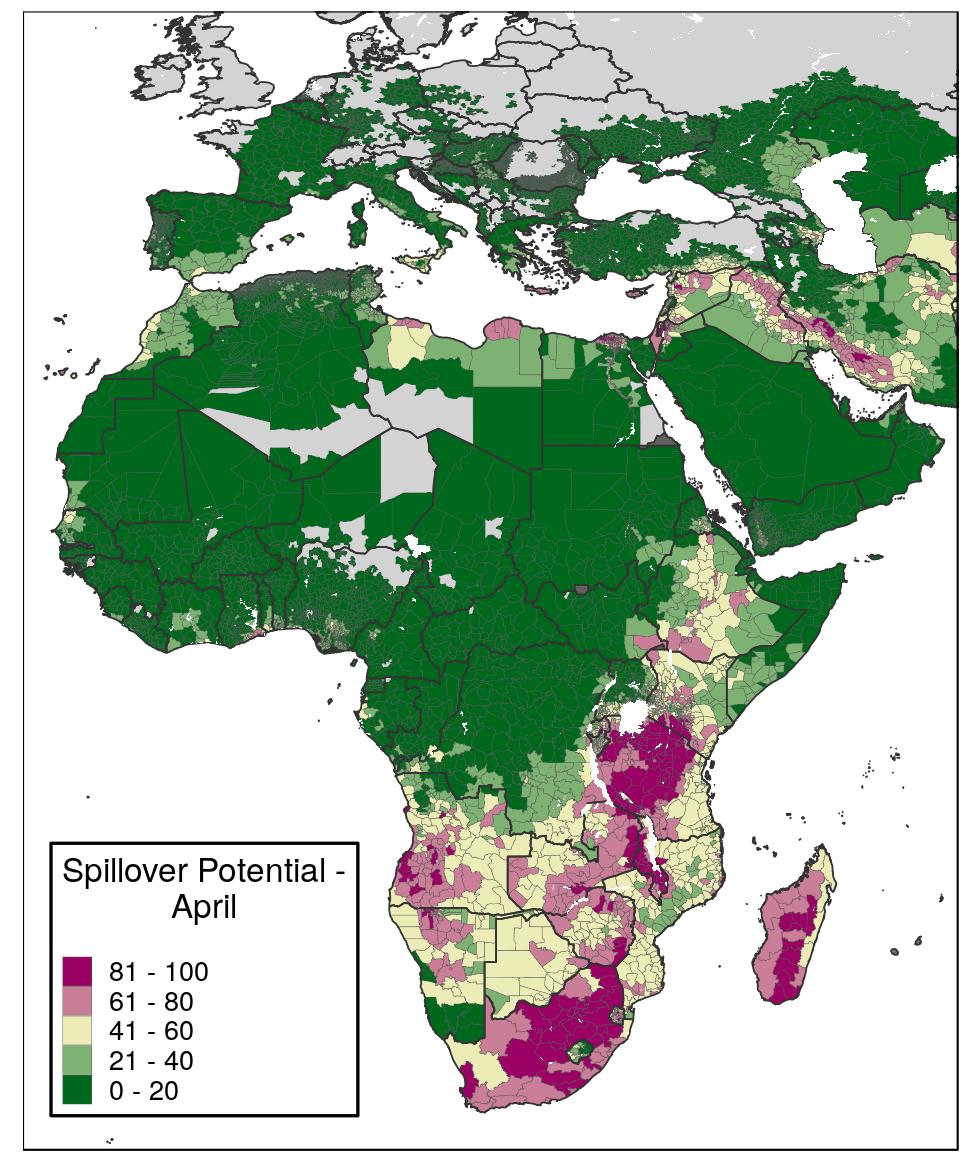


Appendix Figure 71: May mean spillover map

Each district’s average spillover quintile in this month across all years is shown, where dark purple indicates the highest average quintile.
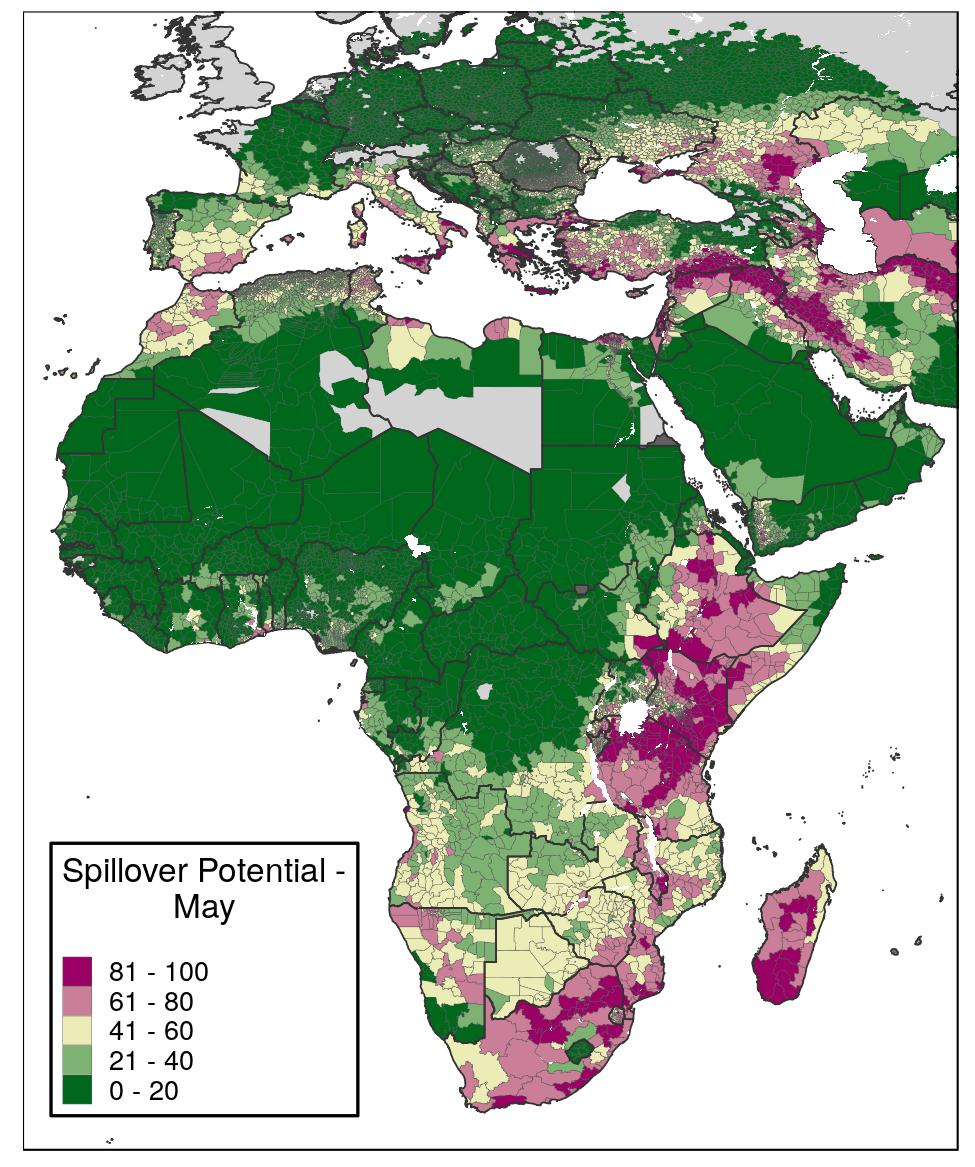


Appendix Figure 72: June mean spillover map

Each district’s average spillover quintile in this month across all years is shown, where dark purple indicates the highest average quintile.


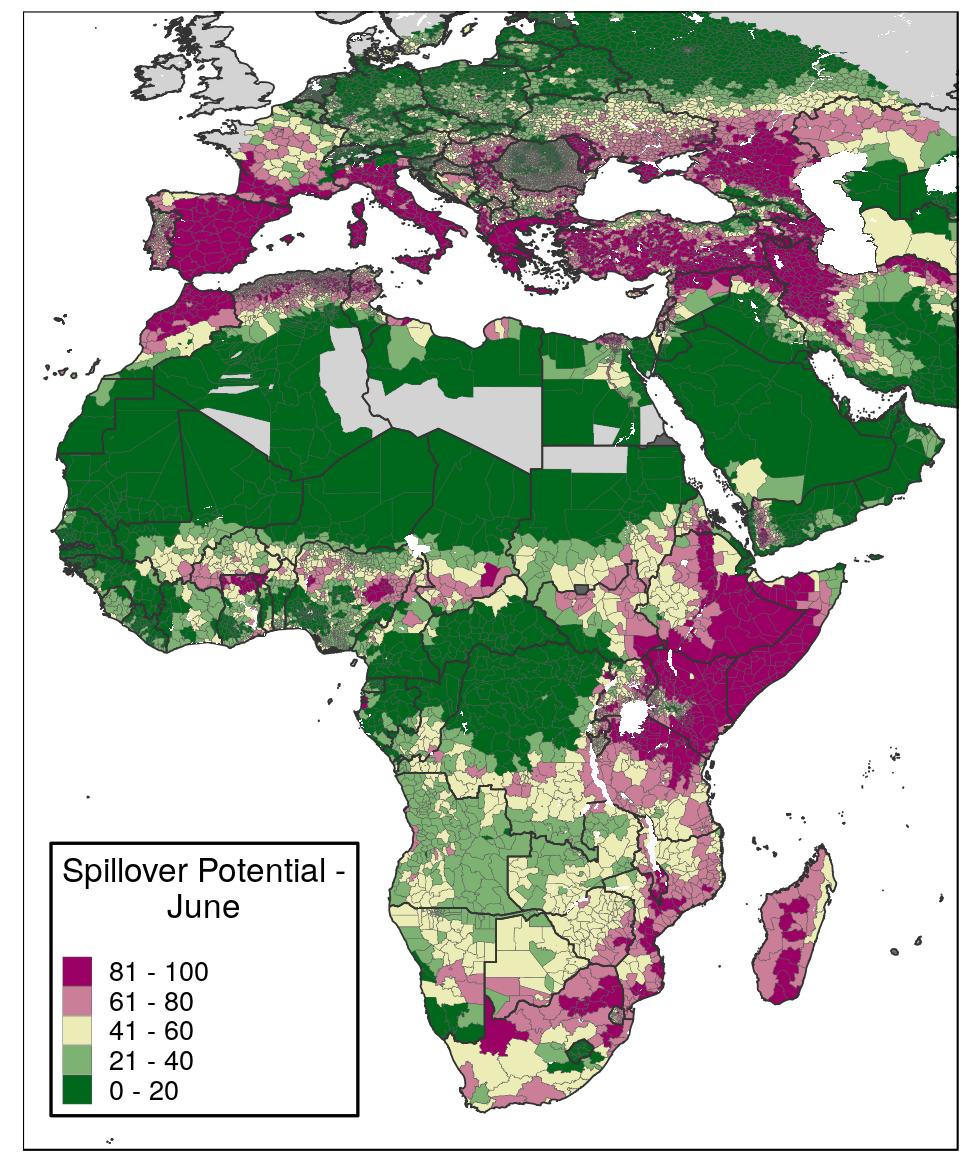


Appendix Figure 73: July mean spillover map

Each district’s average spillover quintile in this month across all years is shown, where dark purple indicates the highest average quintile.


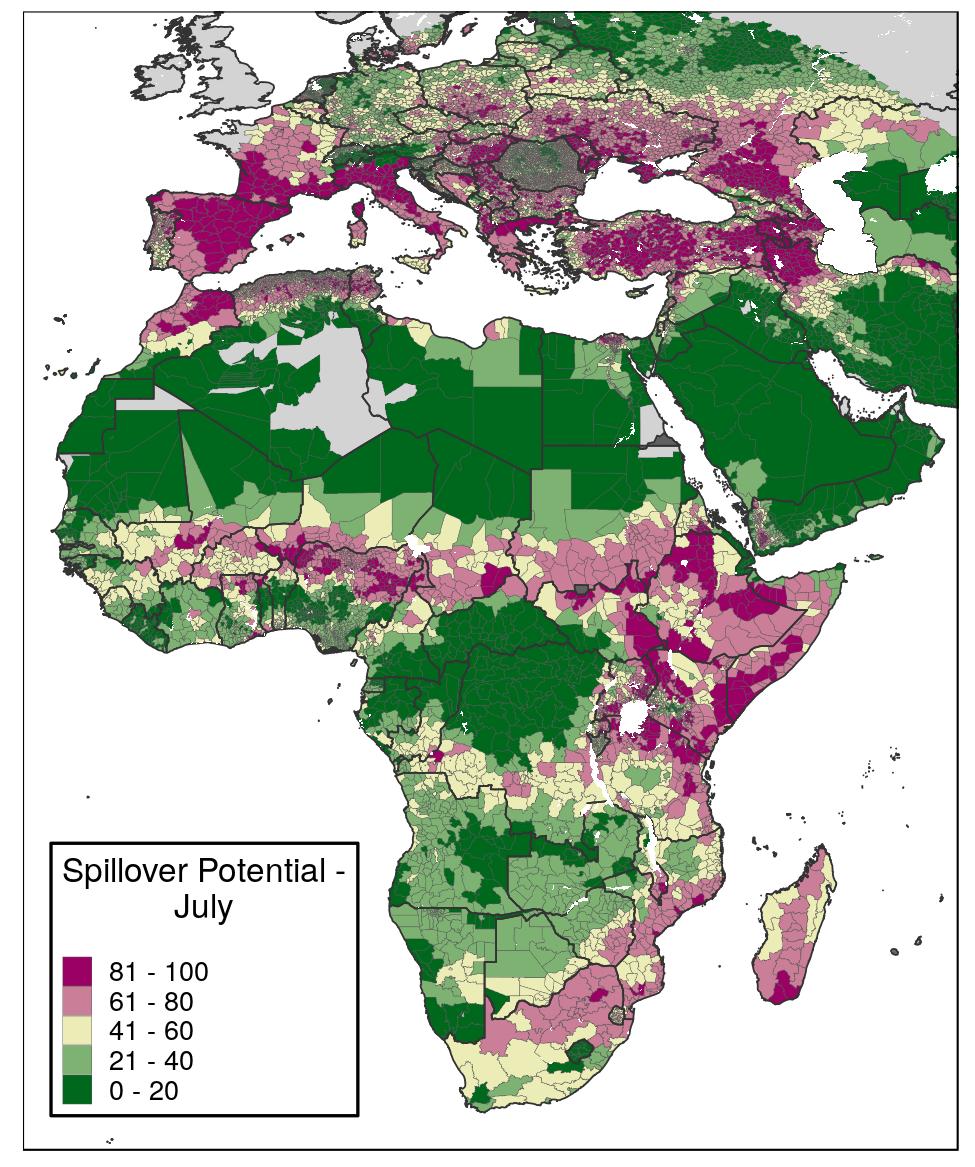


Appendix Figure 74: August mean spillover map

Each district’s average spillover quintile in this month across all years is shown, where dark purple indicates the highest average quintile.


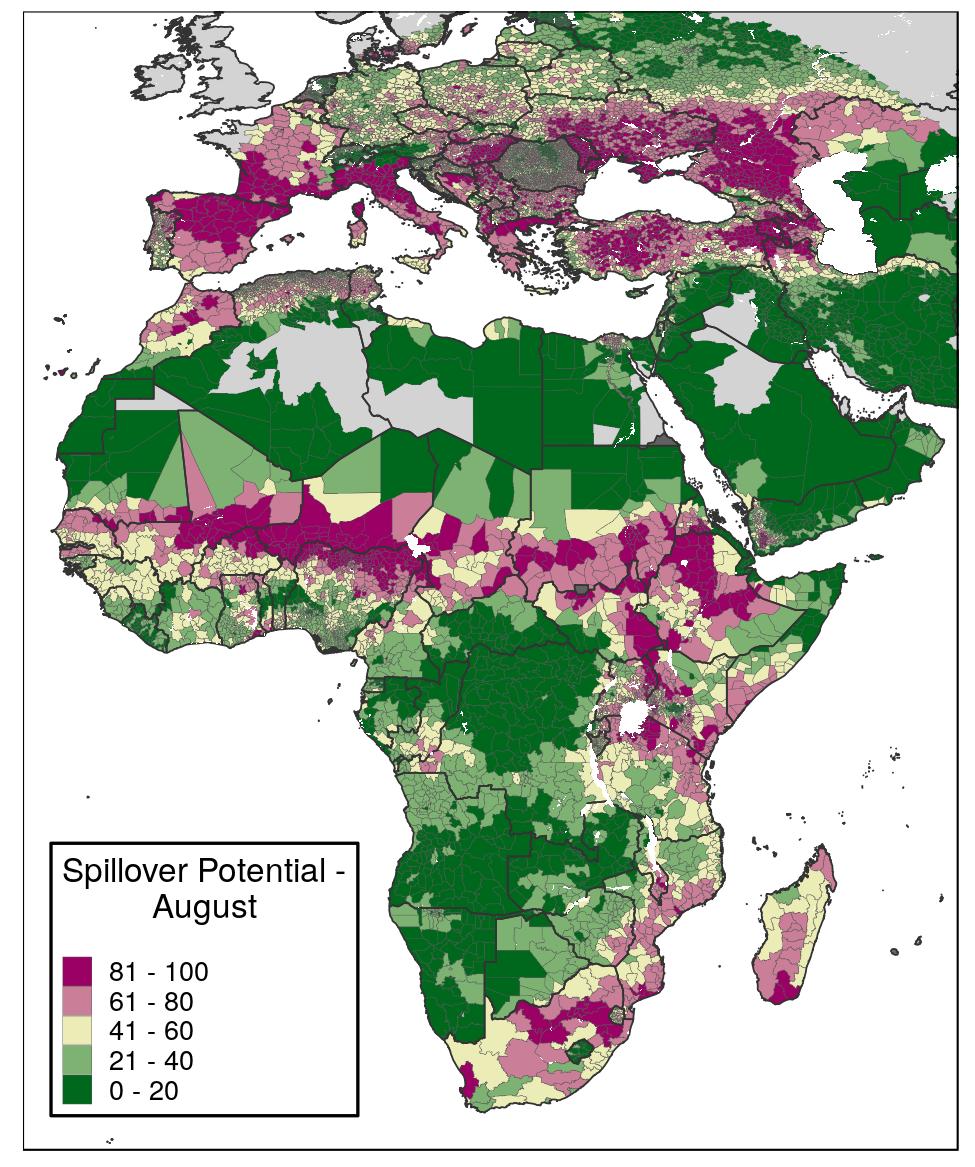


Appendix Figure 75: September mean spillover map

Each district’s average spillover quintile in this month across all years is shown, where dark purple indicates the highest average quintile.


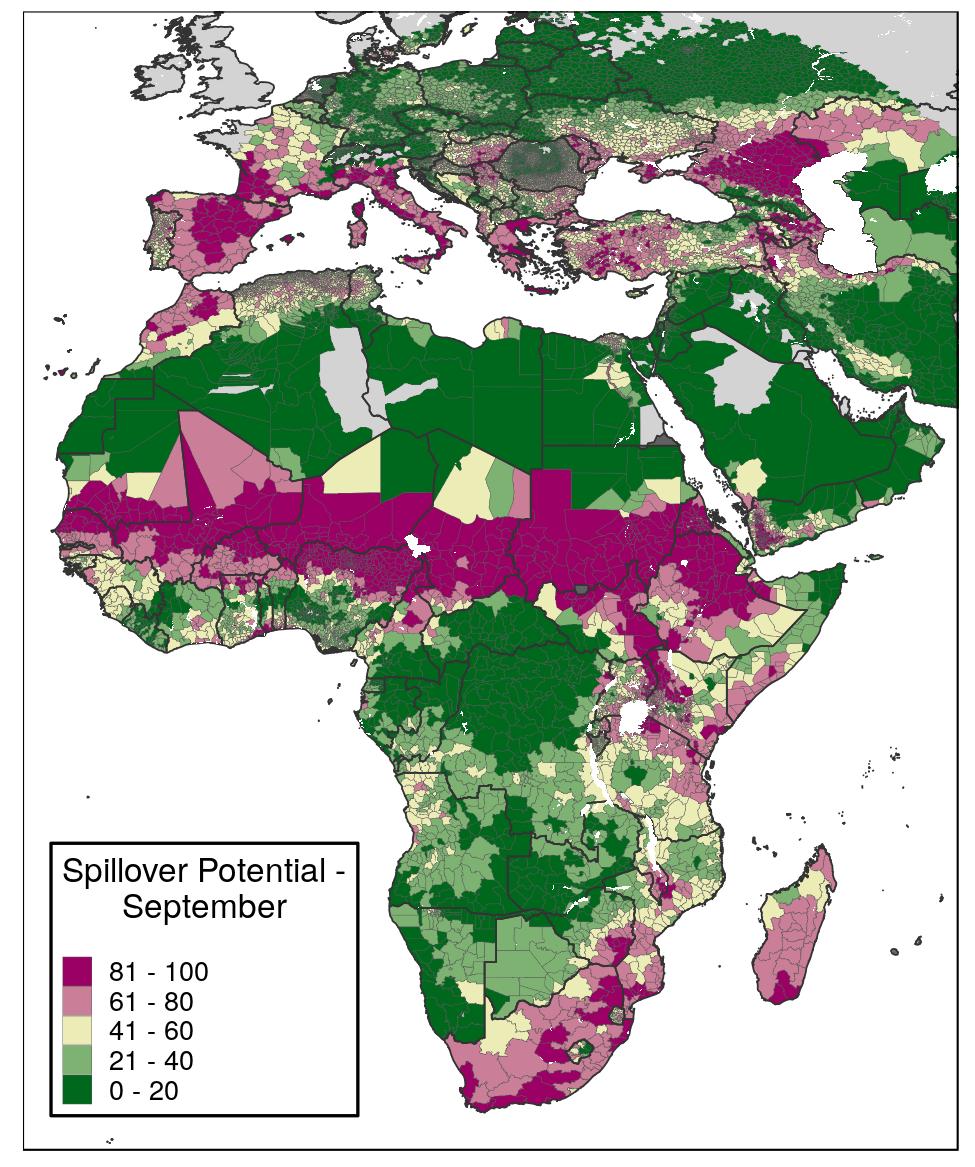


Appendix Figure 76: October mean spillover map

Each district’s average spillover quintile in this month across all years is shown, where dark purple indicates the highest average quintile.
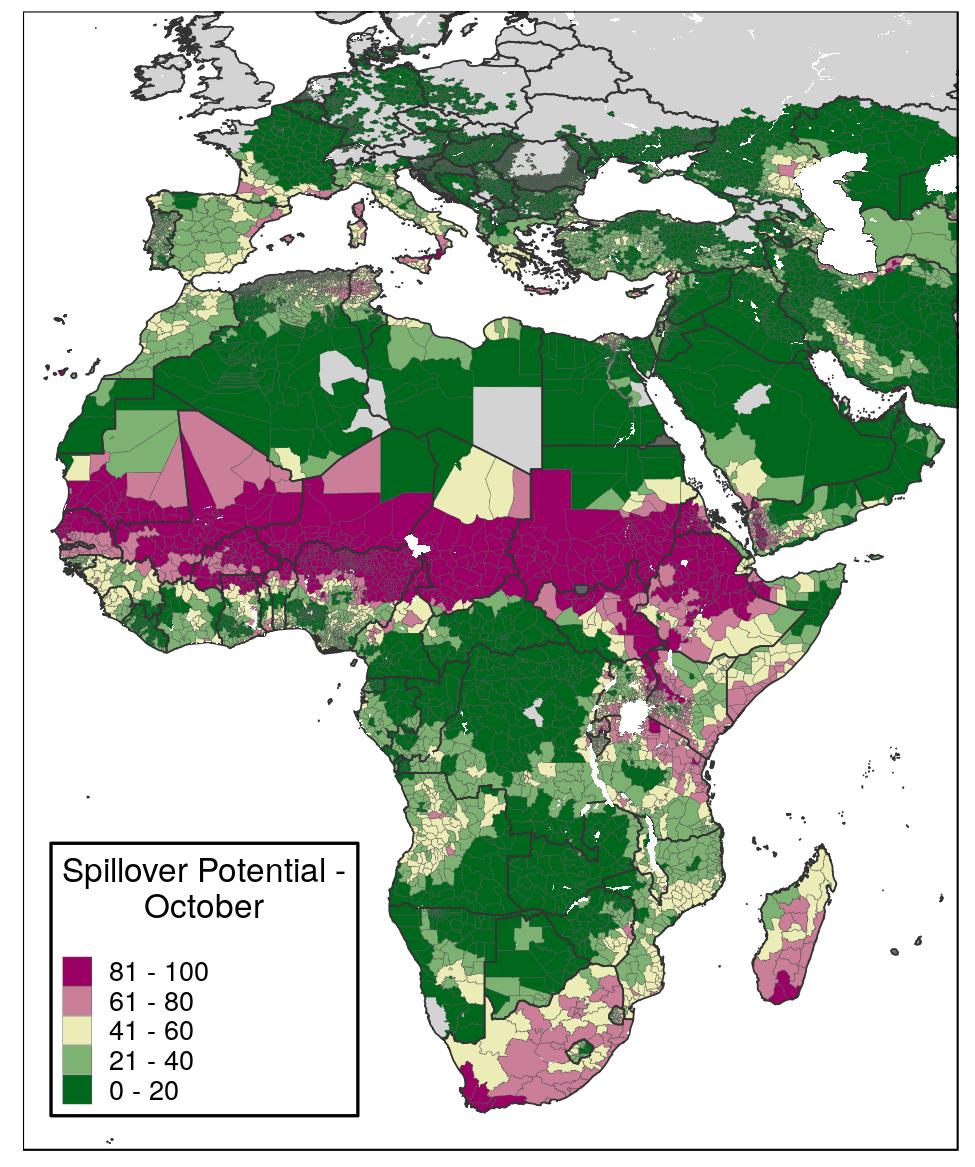


Appendix Figure 77: November mean spillover map

Each district’s average spillover quintile in this month across all years is shown, where dark purple indicates the highest average quintile.


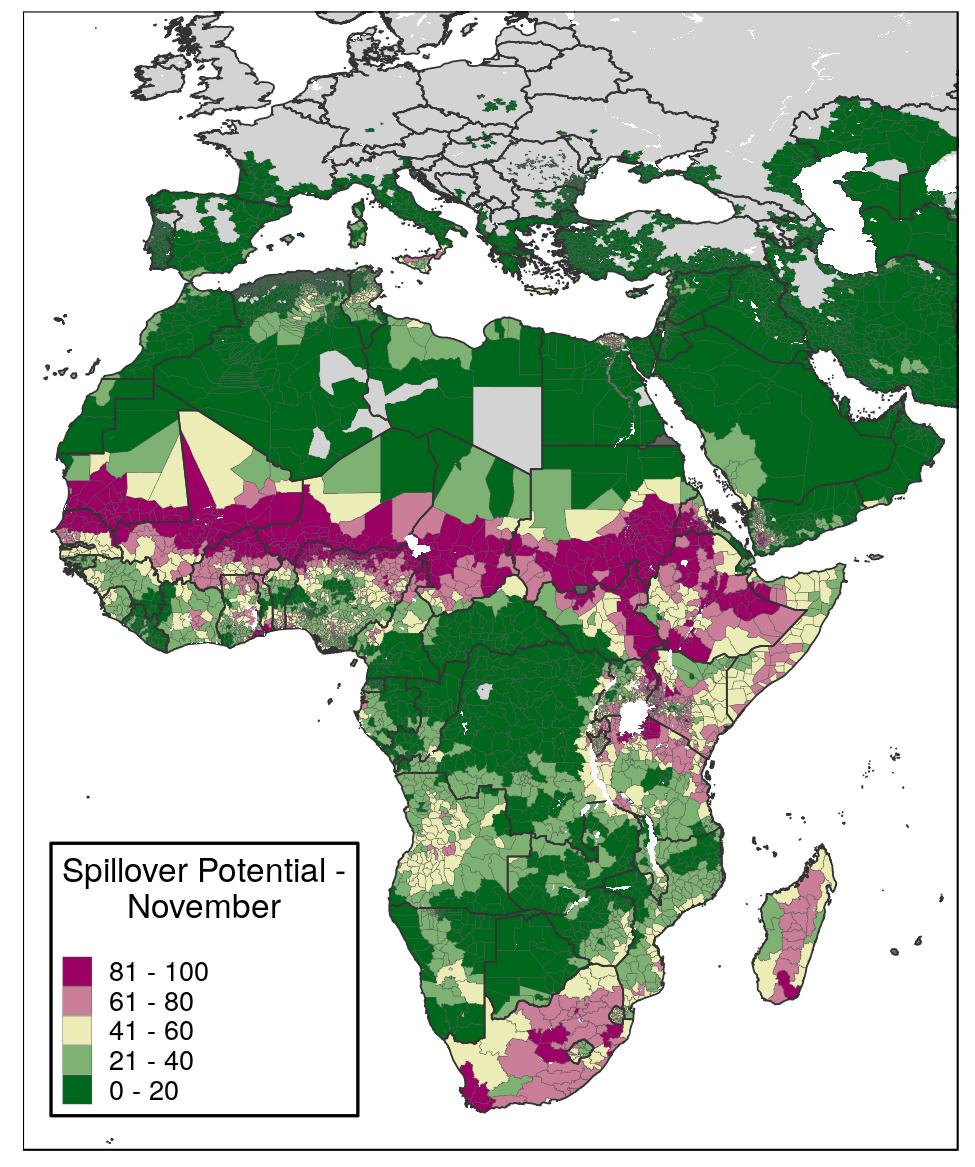


Appendix Figure 78: December mean spillover map

Each district’s average spillover quintile in this month across all years is shown, where dark purple indicates the highest average quintile.


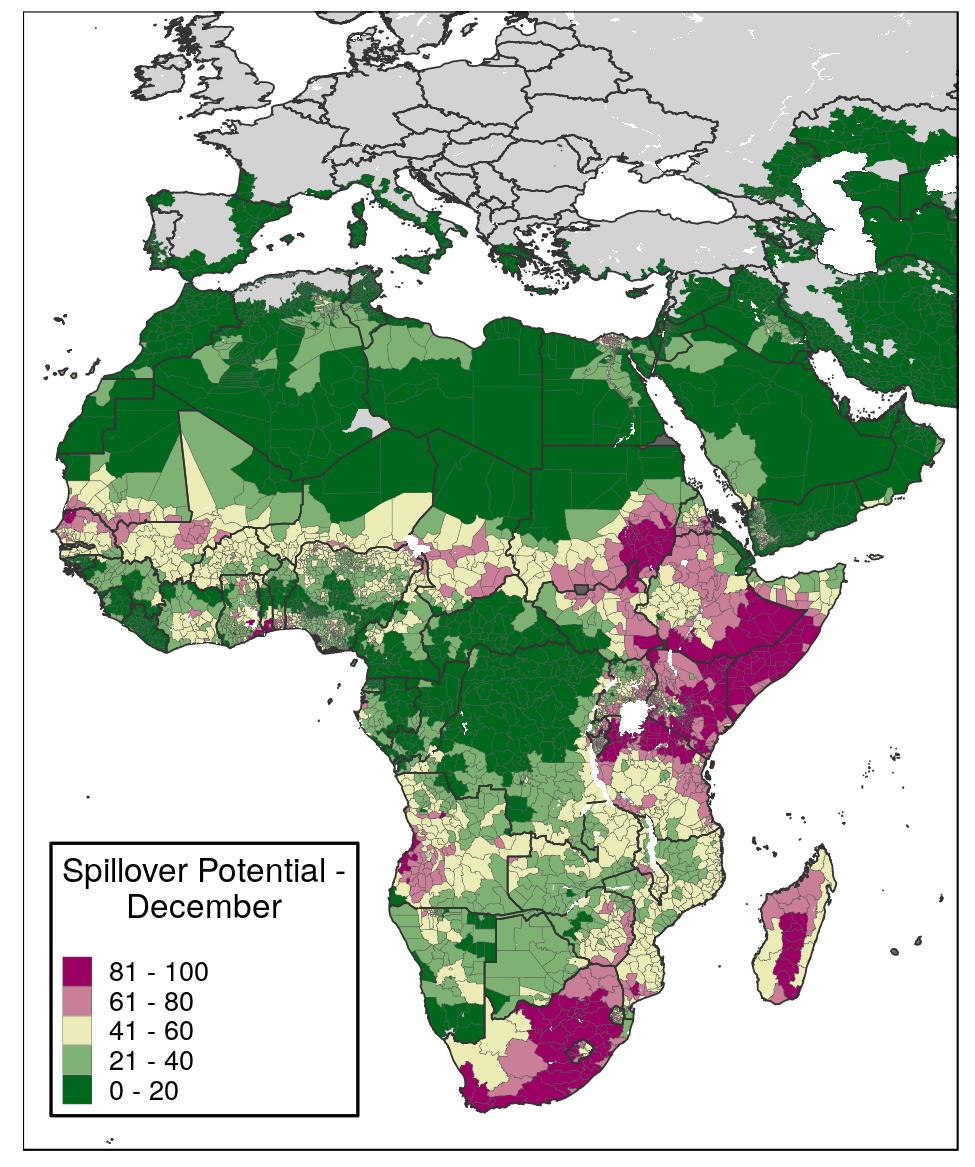


## 5.5 Supplementary experiments

### 5.5.1 Occurrence data from detections in humans

In this experiment, we subset our data to include only occurrences that were detected in humans, which left us with 298 occurrence records. Below we show the geographical distribution of the occurrence data included in this experiment and associated model predictions.

Appendix Figure 79: Map of symptomatic occurrences of Rift Valley Fever in humans


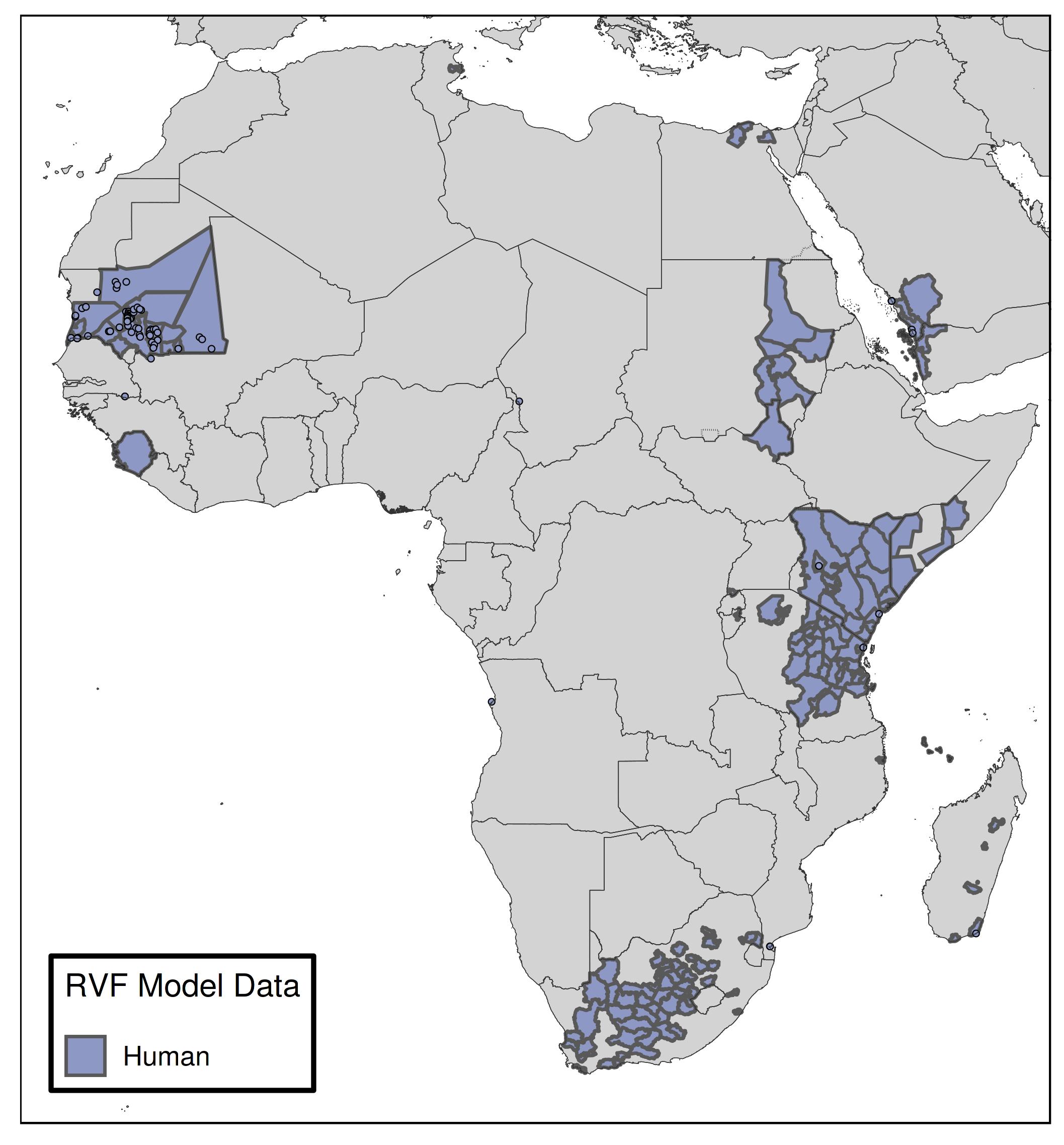


Appendix Figure 80: January binary suitability estimates and confidence intervals modelled with only human occurrence data

For the map of mean suitability predictions for this month, we calculated and applied an optimised threshold using the map and all predictions from occurrences and backgrounds from that month across all years of data (B) for the experiment. We then applied that threshold to maps of lower confidence interval (A) and upper confidence interval (C) predictions. Before having the threshold applied, lower confidence interval estimates for each pixel represent percentile 2.5 of all predictions for that pixel across all bootstraps, and upper confidence interval estimates represent percentile 97.5.


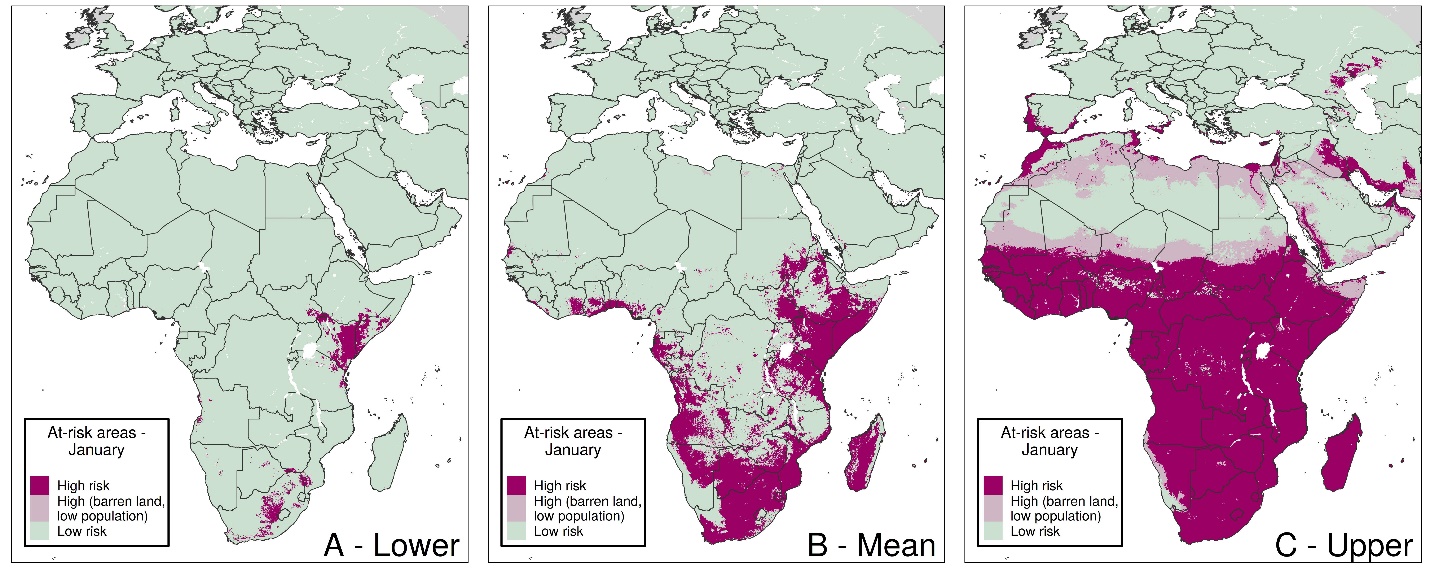


Appendix Figure 81: April binary suitability estimates and confidence intervals modelled with only human occurrence data

For the map of mean suitability predictions for this month, we calculated and applied an optimised threshold using the map and all predictions from occurrences and backgrounds from that month across all years of data (B) for the experiment. We then applied that threshold to maps of lower confidence interval (A) and upper confidence interval (C) predictions. Before having the threshold applied, lower confidence interval estimates for each pixel represent percentile 2.5 of all predictions for that pixel across all bootstraps, and upper confidence interval estimates represent percentile 97.5.


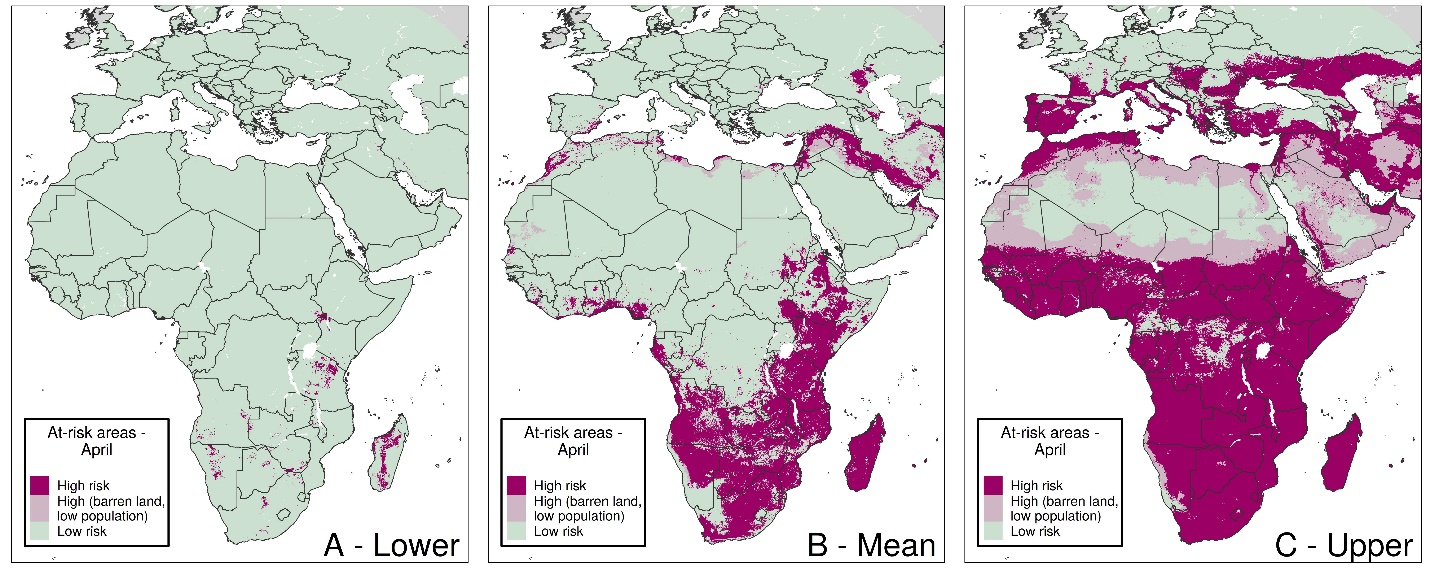


Appendix Figure 82: July binary suitability estimates and confidence intervals modelled with only human occurrence data

For the map of mean suitability predictions for this month, we calculated and applied an optimised threshold using the map and all predictions from occurrences and backgrounds from that month across all years of data (B) for the experiment. We then applied that threshold to maps of lower confidence interval (A) and upper confidence interval (C) predictions. Before having the threshold applied, lower confidence interval estimates for each pixel represent percentile 2.5 of all predictions for that pixel across all bootstraps, and upper confidence interval estimates represent percentile 97.5.


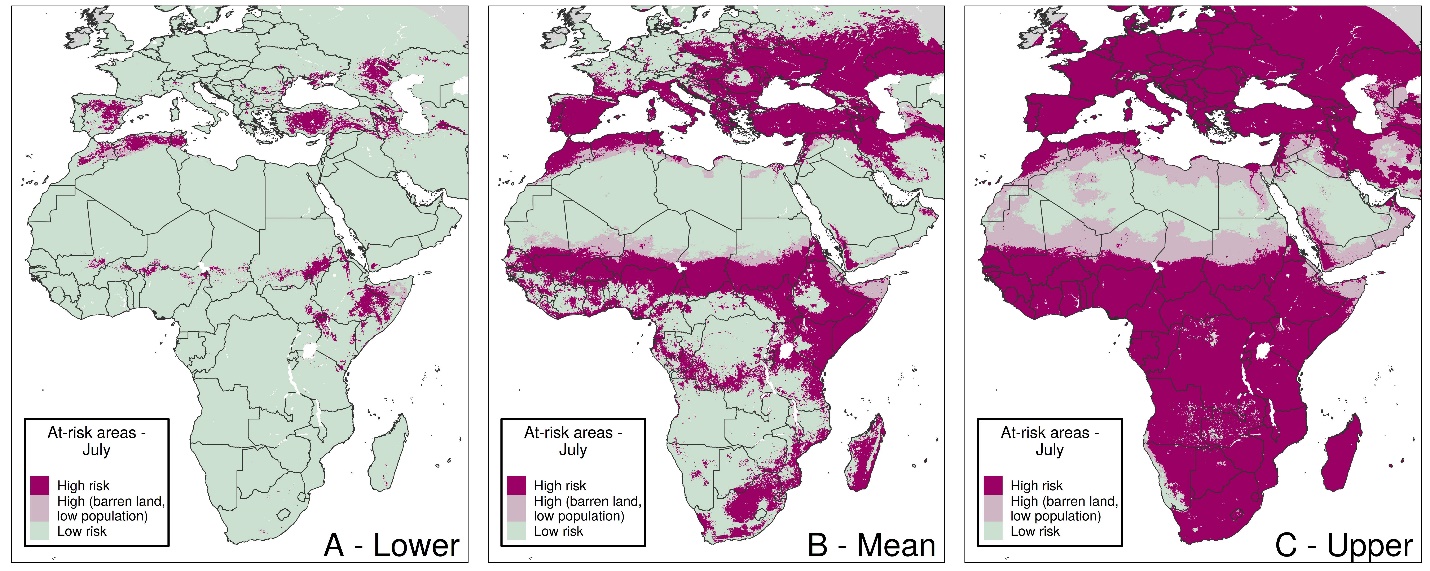


Appendix Figure 83: October binary suitability estimates and confidence intervals modelled with only human occurrence data

For the map of mean suitability predictions for this month, we calculated and applied an optimised threshold using the map and all predictions from occurrences and backgrounds from that month across all years of data (B) for the experiment. We then applied that threshold to maps of lower confidence interval (A) and upper confidence interval (C) predictions. Before having the threshold applied, lower confidence interval estimates for each pixel represent percentile 2.5 of all predictions for that pixel across all bootstraps, and upper confidence interval estimates represent percentile 97.5.


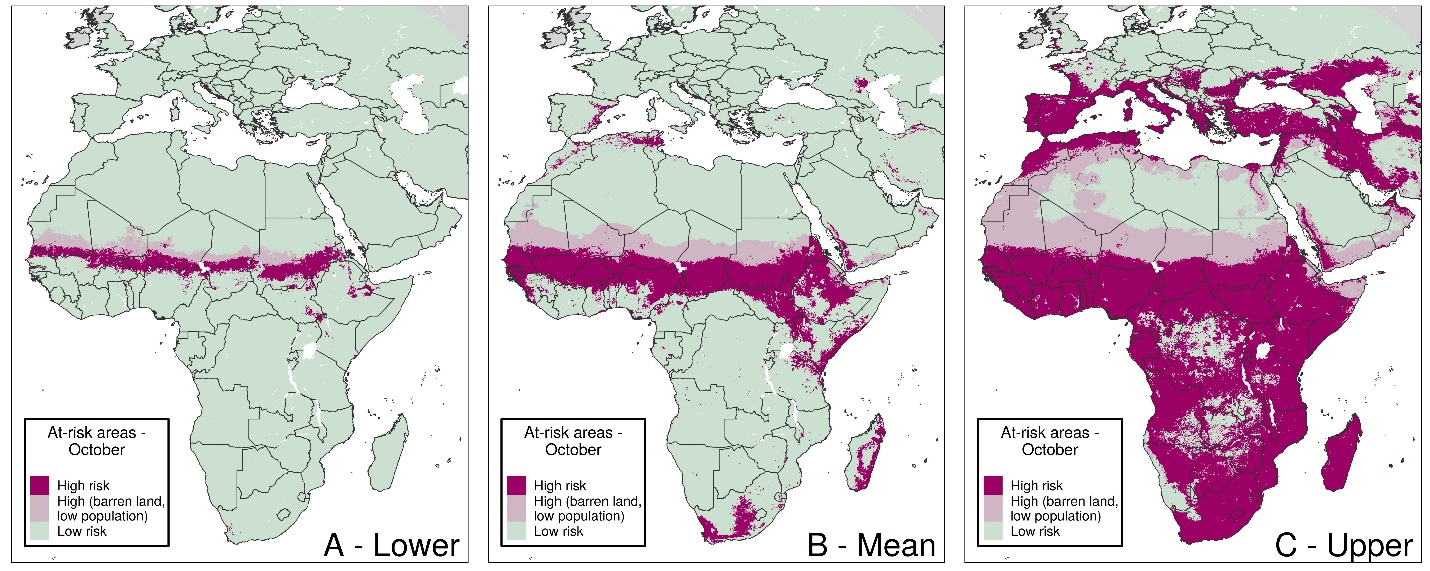


In general, mean map results from this experiment looked similar to results from the model that used the full dataset of symptomatic occurrences, even though the model was based on only 298 records instead of 1,381. Also, this model gave similar mean results even though many of the occurrence records were polygon data. This model showed a high degree of uncertainty, however, likely due to the much smaller dataset. This experiment demonstrates the importance of detecting RVF in mammal and vector species to gain understanding about disease distribution.

### 5.5.2 Occurrence data from detections diagnosed with PCR

In this experiment, we subset our data to include only occurrences that were detected using a PCR diagnostic, which left us with 199 occurrence records. It could be that more cases in our dataset were diagnosed with PCR. However, although we know that cases in the EMPRES-i databases are confirmed at regional FAO partner laboratories, we do not know which diagnostics were used to diagnose individual occurrences. Therefore, EMPRES-i data, which constituted the majority of our dataset, was not included in this experiment. Below we show the geographical distribution of the occurrence data included in this experiment and associated model predictions.

Appendix Figure 84: Map of Rift Valley Fever occurrences diagnosed with PCR


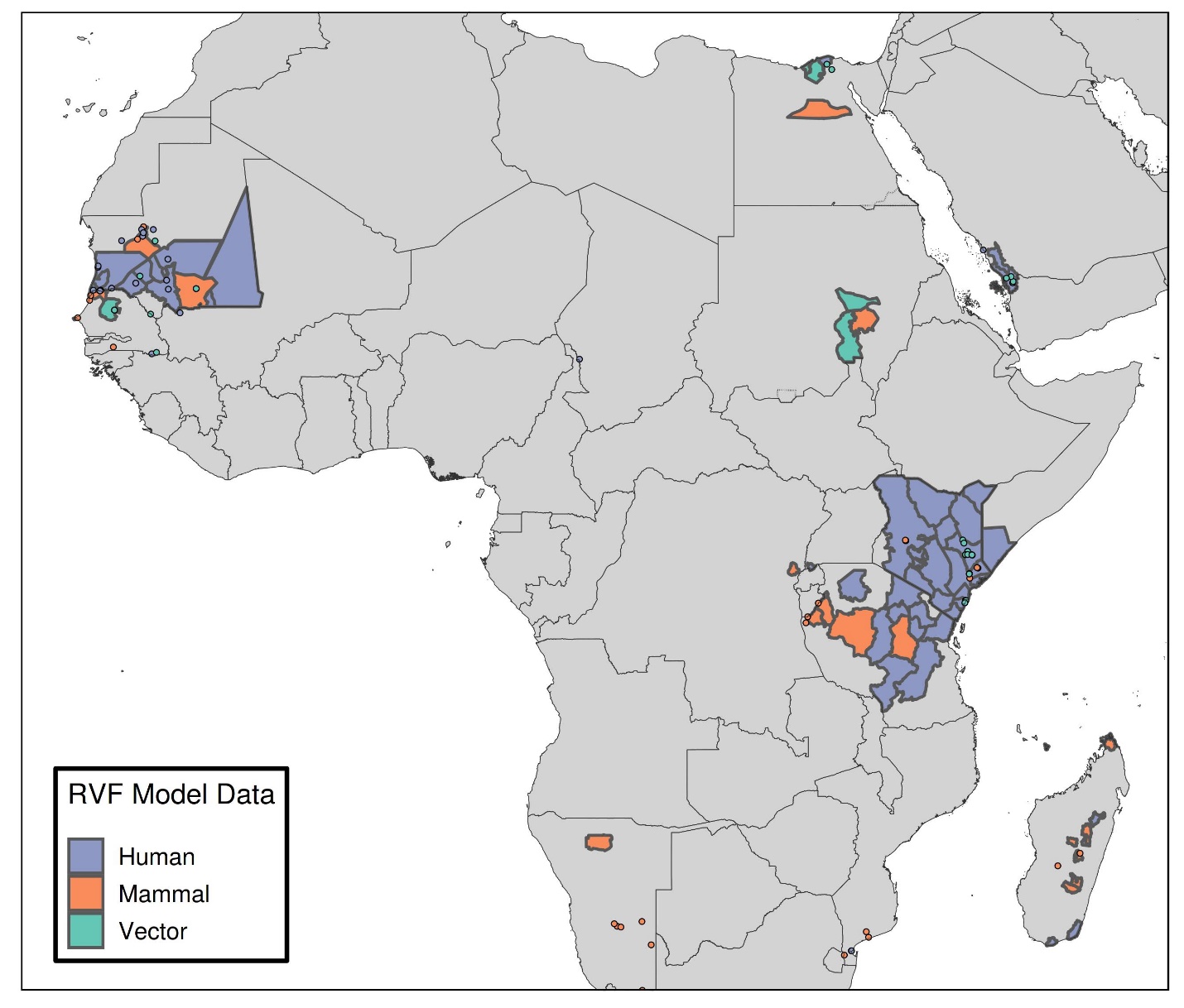


Appendix Figure 85: January binary suitability estimates and confidence intervals modelled with only occurrence data detected using PCR

For the map of mean suitability predictions for this month, we calculated and applied an optimised threshold using the map and all predictions from occurrences and backgrounds from that month across all years of data (B) for the experiment. We then applied that threshold to maps of lower confidence interval (A) and upper confidence interval (C) predictions. Before having the threshold applied, lower confidence interval estimates for each pixel represent percentile 2.5 of all predictions for that pixel across all bootstraps, and upper confidence interval estimates represent percentile 97.5.


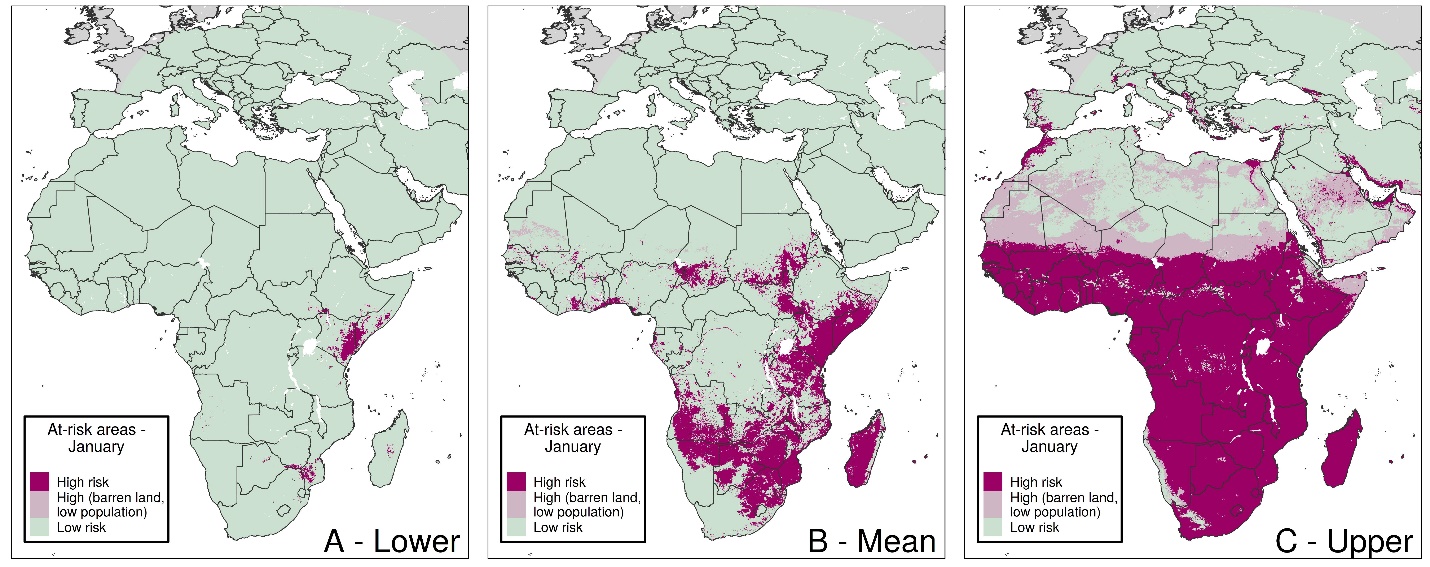


Appendix Figure 86: April binary suitability estimates and confidence intervals modelled with only occurrence data detected using PCR

For the map of mean suitability predictions for this month, we calculated and applied an optimised threshold using the map and all predictions from occurrences and backgrounds from that month across all years of data (B) for the experiment. We then applied that threshold to maps of lower confidence interval (A) and upper confidence interval (C) predictions. Before having the threshold applied, lower confidence interval estimates for each pixel represent percentile 2.5 of all predictions for that pixel across all bootstraps, and upper confidence interval estimates represent percentile 97.5.


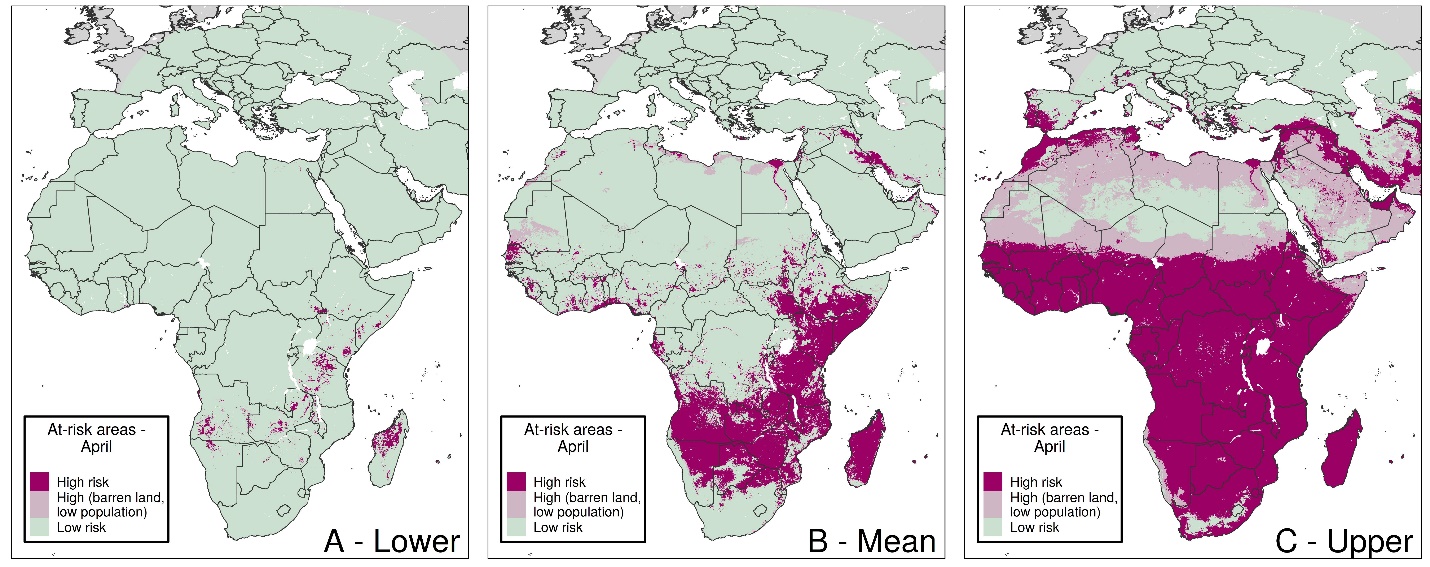


Appendix Figure 87: July binary suitability estimates and confidence intervals modelled with only occurrence data detected using PCR

For the map of mean suitability predictions for this month, we calculated and applied an optimised threshold using the map and all predictions from occurrences and backgrounds from that month across all years of data (B) for the experiment. We then applied that threshold to maps of lower confidence interval (A) and upper confidence interval (C) predictions. Before having the threshold applied, lower confidence interval estimates for each pixel represent percentile 2.5 of all predictions for that pixel across all bootstraps, and upper confidence interval estimates represent percentile 97.5.


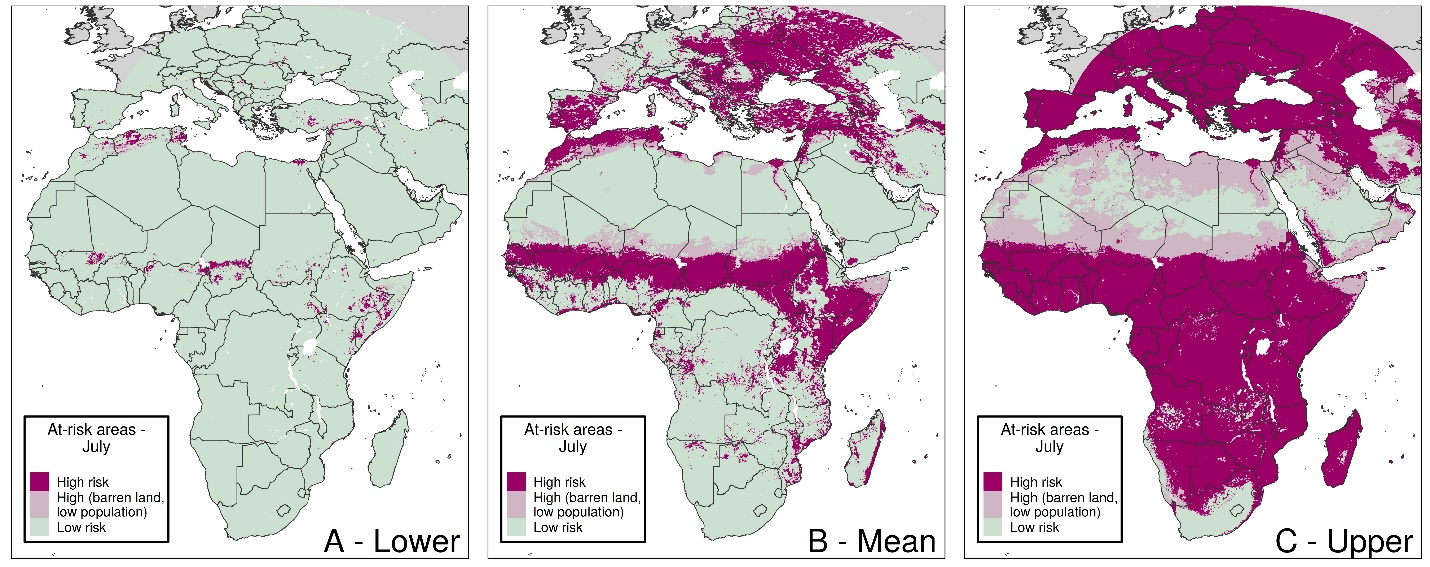


Appendix Figure 88: October binary suitability estimates and confidence intervals modelled with only occurrence data detected using PCR

For the map of mean suitability predictions for this month, we calculated and applied an optimised threshold using the map and all predictions from occurrences and backgrounds from that month across all years of data (B) for the experiment. We then applied that threshold to maps of lower confidence interval (A) and upper confidence interval (C) predictions. Before having the threshold applied, lower confidence interval estimates for each pixel represent percentile 2.5 of all predictions for that pixel across all bootstraps, and upper confidence interval estimates represent percentile 97.5.


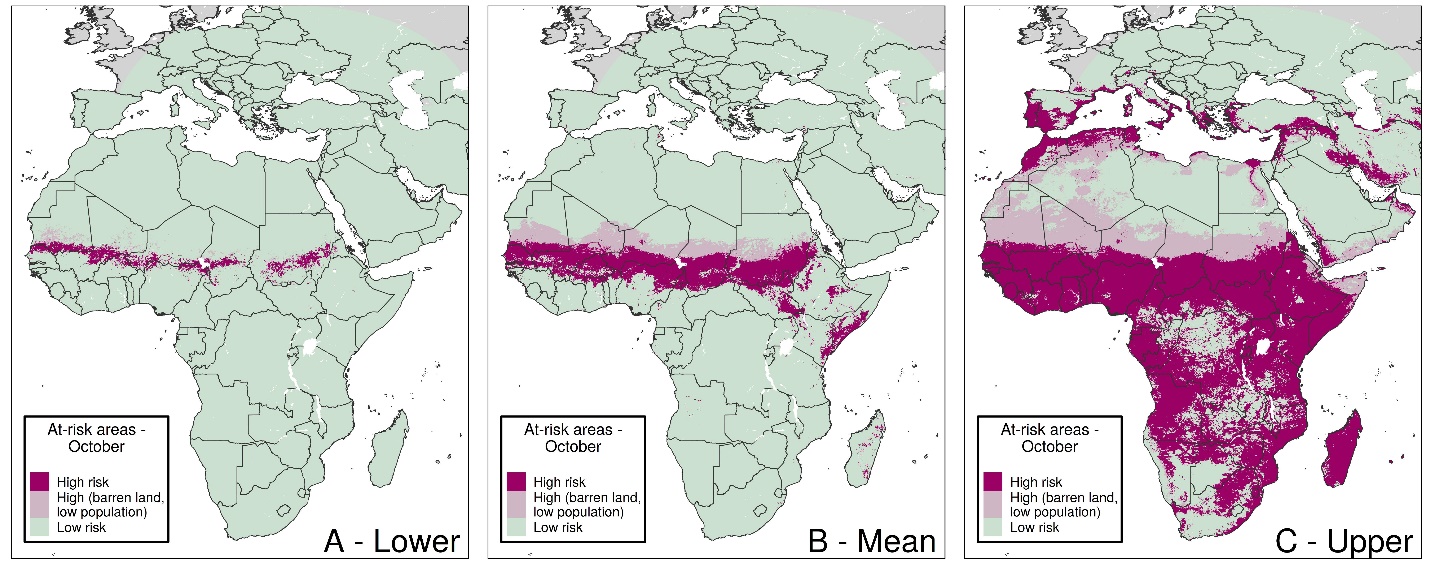


Running our model using only occurrence points that were detected by PCR simulates the effects of only including data from places with more advanced diagnostic capacity. Filtering the data in this way removed 1,182 of our 1,381 datapoints, including all datapoints from South Africa. While many predicted areas were still similar, this model removed much of South Africa from our mean predictions in certain months; however, it primarily greatly increased the uncertainty, such that the upper confidence interval of this experiment still included much of the country in the binary predictions.

### 5.5.3 Occurrence data with exact date information

In this experiment, we subset our data to include only occurrences that were reported with exact month-year date combinations, such that we did not have to sample a month and year from a date range for any occurrence record. This subset left us with 948 occurrence records. Below we show the geographical distribution of the occurrence data included in this experiment and associated model predictions.

Appendix Figure 89: Map of Rift Valley Fever occurrences reported with exact date information


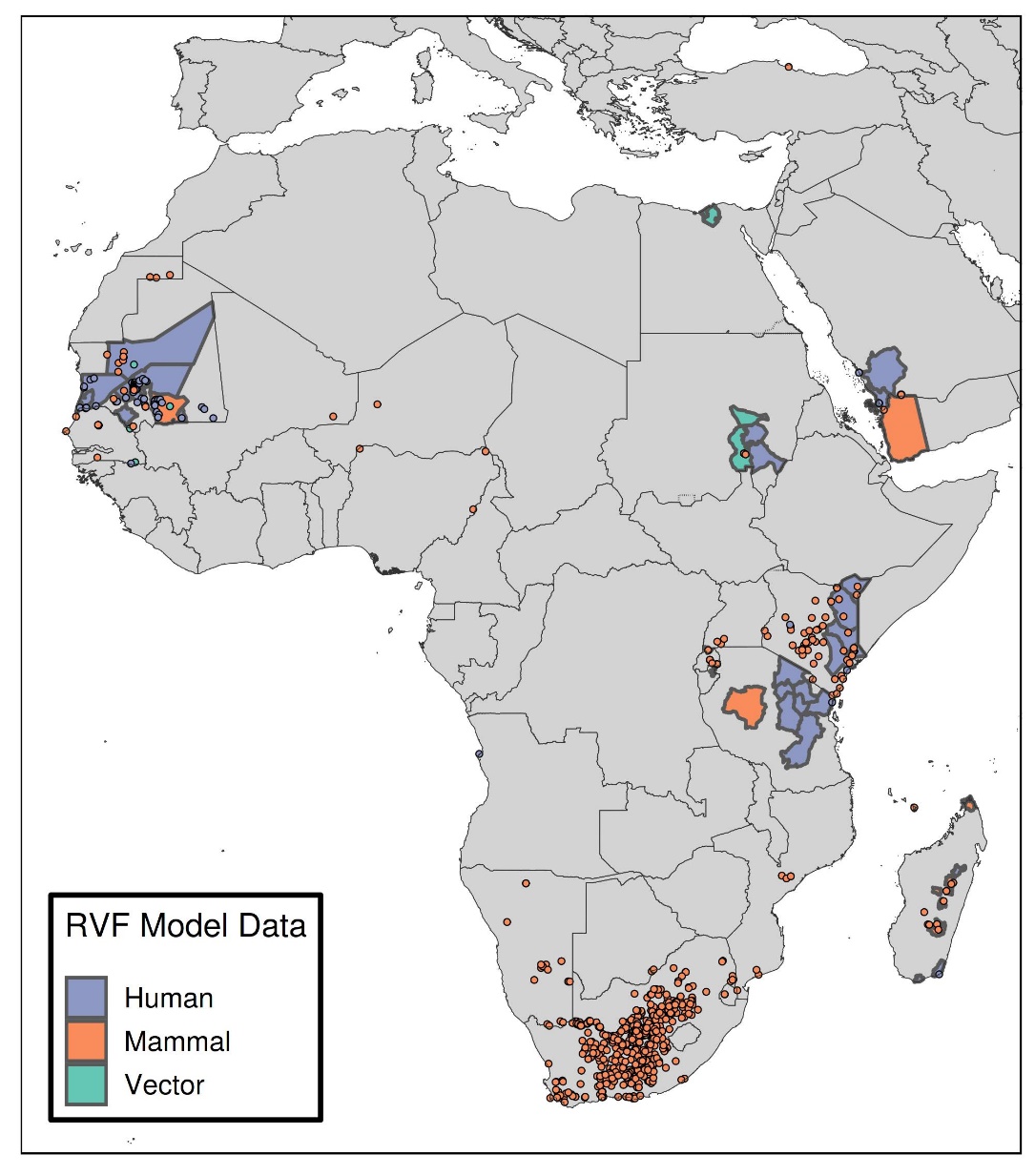


Appendix Figure 90: January binary suitability estimates and confidence intervals modelled with only occurrence data reported with exact date information

For the map of mean suitability predictions for this month, we calculated and applied an optimised threshold using the map and all predictions from occurrences and backgrounds from that month across all years of data (B) for the experiment. We then applied that threshold to maps of lower confidence interval (A) and upper confidence interval (C) predictions. Before having the threshold applied, lower confidence interval estimates for each pixel represent percentile 2.5 of all predictions for that pixel across all bootstraps, and upper confidence interval estimates represent percentile 97.5.


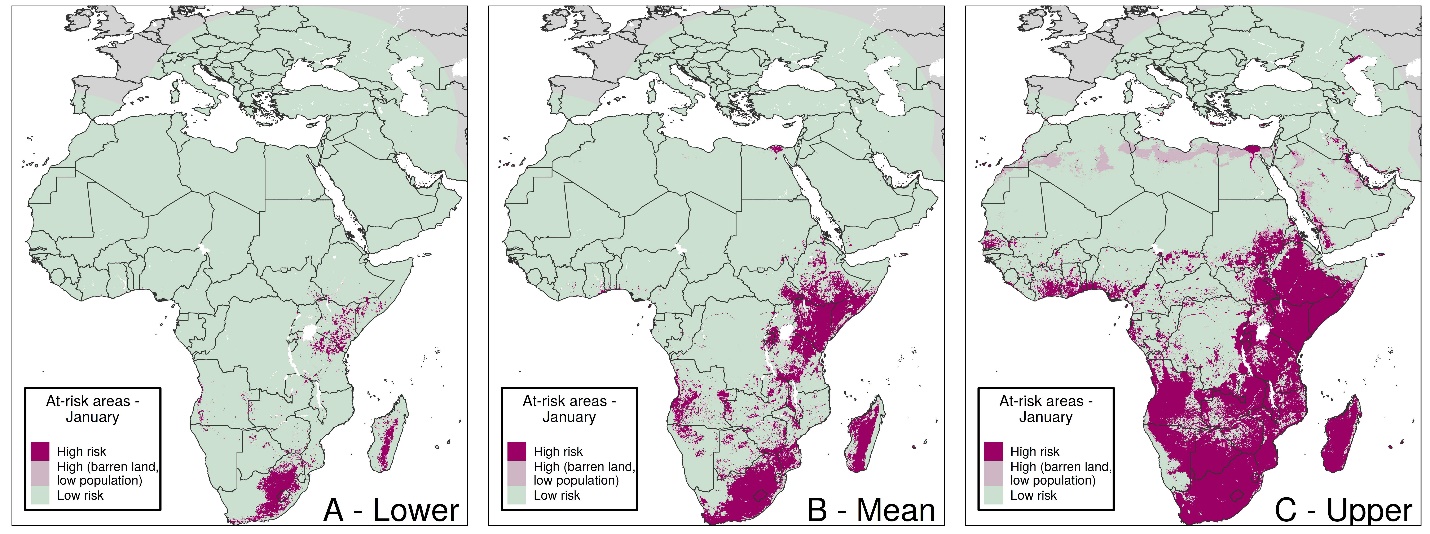


Appendix Figure 91: April binary suitability estimates and confidence intervals modelled with only occurrence data reported with exact date information

For the map of mean suitability predictions for this month, we calculated and applied an optimised threshold using the map and all predictions from occurrences and backgrounds from that month across all years of data (B) for the experiment. We then applied that threshold to maps of lower confidence interval (A) and upper confidence interval (C) predictions. Before having the threshold applied, lower confidence interval estimates for each pixel represent percentile 2.5 of all predictions for that pixel across all bootstraps, and upper confidence interval estimates represent percentile 97.5.


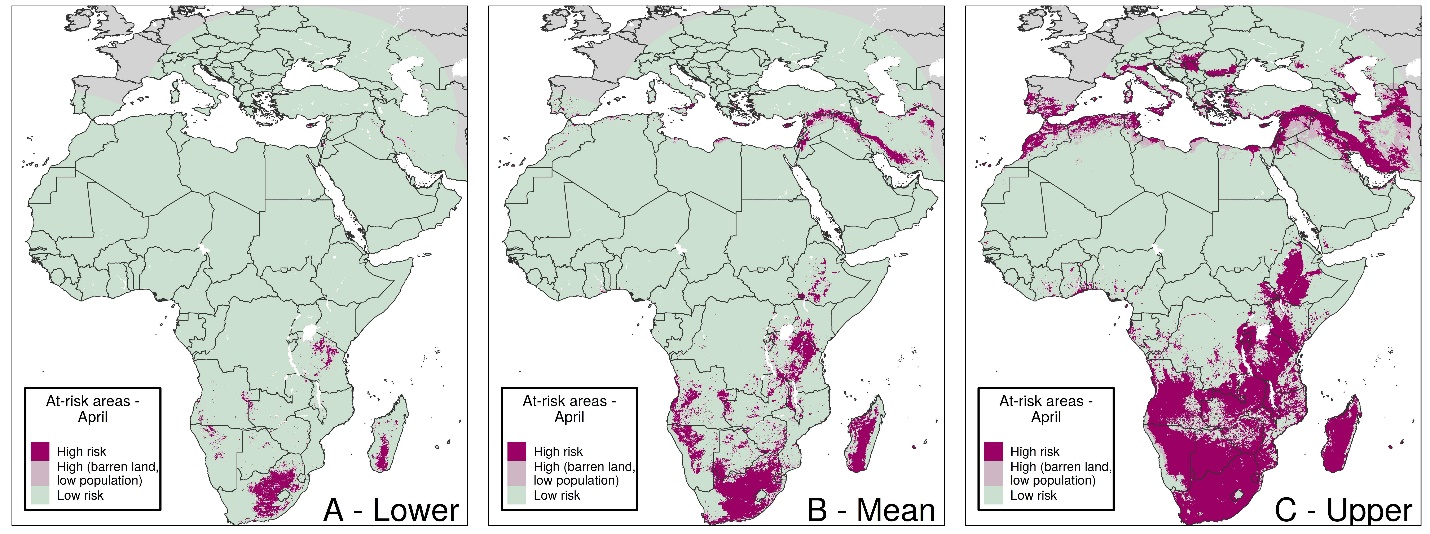


Appendix Figure 92: July binary suitability estimates and confidence intervals modelled with only occurrence data reported with exact date information

For the map of mean suitability predictions for this month, we calculated and applied an optimised threshold using the map and all predictions from occurrences and backgrounds from that month across all years of data (B) for the experiment. We then applied that threshold to maps of lower confidence interval (A) and upper confidence interval (C) predictions. Before having the threshold applied, lower confidence interval estimates for each pixel represent percentile 2.5 of all predictions for that pixel across all bootstraps, and upper confidence interval estimates represent percentile 97.5.


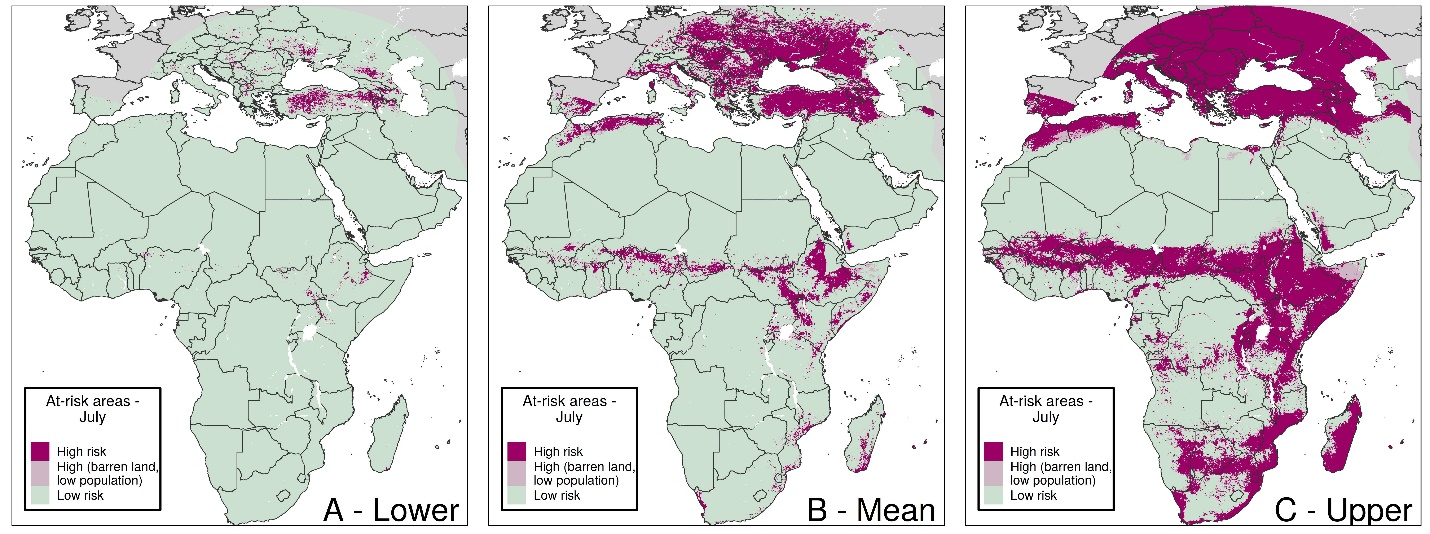


Appendix Figure 93: October binary suitability estimates and confidence intervals modelled with only occurrence data reported with exact date information

For the map of mean suitability predictions for this month, we calculated and applied an optimised threshold using the map and all predictions from occurrences and backgrounds from that month across all years of data (B) for the experiment. We then applied that threshold to maps of lower confidence interval (A) and upper confidence interval (C) predictions. Before having the threshold applied, lower confidence interval estimates for each pixel represent percentile 2.5 of all predictions for that pixel across all bootstraps, and upper confidence interval estimates represent percentile 97.5.


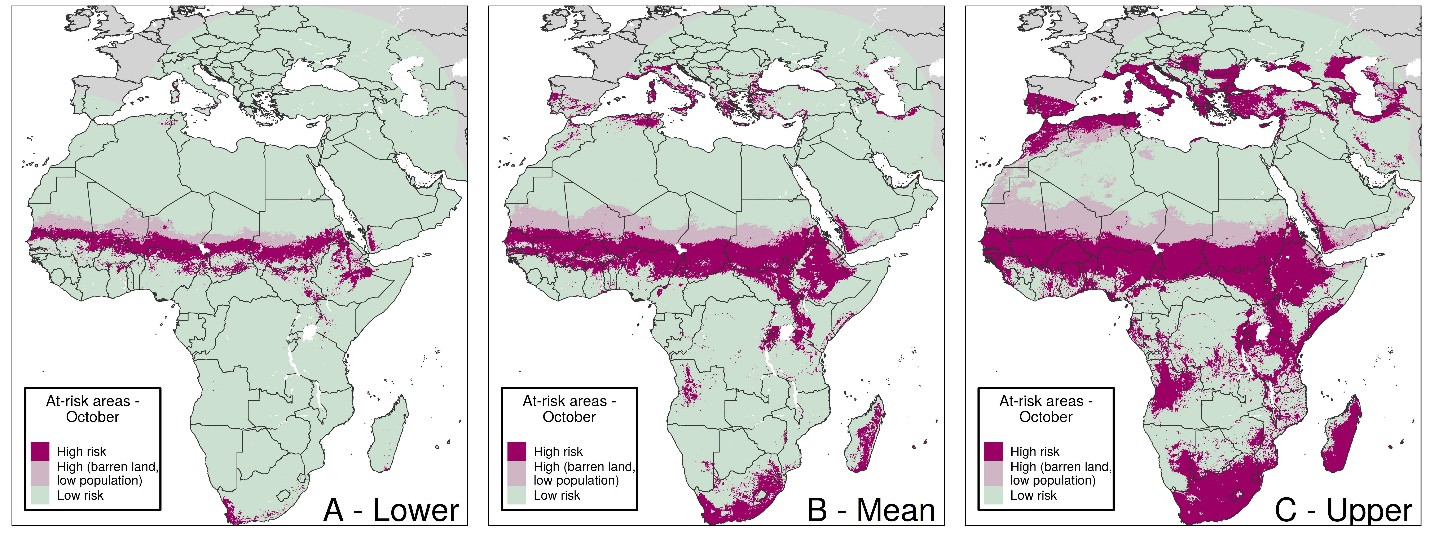


In this experiment, we sought to analyse how our model would change if we did not have to sample date information for any records. We kept most of our dataset in this experiment, since most EMPRES-i data were reported with exact date information. A notable difference in the mean outputs compared to the model with the full dataset is the removal of much of central and south Africa during the month of July. While we kept most of our data in this experiment subset, it left us with even fewer datapoints than we already had in the months surrounding July, as shown in the figure below. This experiments’ mean predictions for July do not predict an area of southern Namibia where an outbreak occurred in May-July of 2010,^7^ while our model built using the full dataset does. More locations are included in the upper confidence interval, but this experiment shows that important regions might be included as suitable during certain times as a result of including data with imprecise corresponding time information and sampling from within the range over multiple bootstraps.

Appendix Figure 94: Counts by month of records with exact date information

Shown below are counts of occurrence records from the experiment which included only records reported with exact date information.


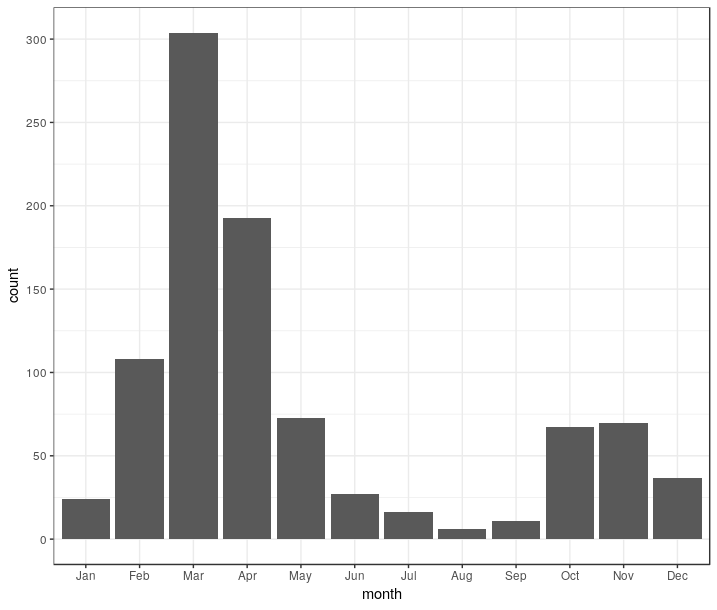


# 6.0 Supplementary Limitations

Studies have suggested that some mosquito species have a critical length of suitability before a sufficient population is developed to pose a threat,^19^ another factor that could be considered in individual locations, especially those with a small number of months predicted to be suitable. Our spillover maps did not incorporate vector populations because of the current lack of information on distributions of many of the vectors that can transmit RVF.

Spatial clustering is a concern for our model only if it corresponds to clustering in covariate space. As demonstrated by the multivariate environmental similarity surface (MESS) plots (Appendix Figures 11-22), our occurrence data covered a wide range of environmental conditions in covariate space, due in part to occurrences in the same area happening in different seasons. Because of this coverage, many predictions were still made by interpolating between occurrence data (Appendix Figures 11-22), though predictions in some locations in certain months were extrapolated.

Different regions have different climatic relationships with RVF,^8,20,21^ and while we believe our model captures many of these differences, region-specific analyses that account for seasonality should still be conducted.

# 7.0 Supplementary references

1 Tibshirani R, Walther G, Hastie T. Estimating the number of clusters in a data set via the gap statistic. *J R Stat Soc Ser B Stat Methodol* 2001; **63**: 411–23.

2 Elith J, Leathwick JR, Hastie T. A working guide to boosted regression trees. *J Anim Ecol* 2008; **77**: 802–13.

3 Friedman JH. Stochastic gradient boosting. *Comput Stat Data Anal* 2002; **38**: 367–78.

4 Linthicum KJ, Britch SC, Anyamba A. Rift Valley Fever: An Emerging Mosquito-Borne Disease. *Annu Rev Entomol* 2016; **61**: 395–415.

5 Davies FG, Linthicum KJ, James AD. Rainfall and epizootic Rift Valley fever. *Bull World Health Organ* 1985; **63**: 941–3.

6 Nardi F, Annis A, Di Baldassarre G, Vivoni ER, Grimaldi S. GFPLAIN250m, a global high-resolution dataset of Earth’s floodplains. *Sci Data* 2019; **6**: 180309.

7 Monaco F, Pinoni C, Cosseddu GM, *et al.* Rift Valley Fever in Namibia, 2010. *Emerg Infect Dis* 2013; **19**: 2025–7.

8 Caminade C, Ndione JA, Diallo M, *et al.* Rift Valley Fever Outbreaks in Mauritania and Related Environmental Conditions. *Int J Environ Res Public Health* 2014; **11**: 903–18.

9 Sindato C, Karimuribo ED, Pfeiffer DU, *et al.* Spatial and Temporal Pattern of Rift Valley Fever Outbreaks in Tanzania; 1930 to 2007. *PLoS ONE* 2014; **9**: e88897.

10 Linthicum KJ. Climate and Satellite Indicators to Forecast Rift Valley Fever Epidemics in Kenya. *Science* 1999; **285**: 397–400.

11 Alaoui A, Rogger M, Peth S, Blöschl G. Does soil compaction increase floods? A review. *J Hydrol* 2018; **557**: 631–42.

12 Rueda LM, Patel KJ, Axtell RC, Stinner RE. Temperature-Dependent Development and Survival Rates of Culex quinquefasciatus and Aedes aegypti (Diptera: Culicidae). *J Med Entomol* 1990; **27**: 892–8.

13 Anyamba A. Mapping Potential Risk of Rift Valley Fever Outbreaks in African Savannas Using Vegetation Index Time Series Data. ; : 10.

14 Linthicum KJ, Bailey CL, Davies FG, Tucker CJ. Detection of Rift Valley fever viral activity in Kenya by satellite remote sensing imagery. *Science* 1987; **235**: 1656–9.

15 Elith J, Kearney M, Phillips S. The art of modelling range-shifting species. *Methods Ecol Evol* 2010; **1**: 330–42.

16 Lloyd CT, Sorichetta A, Tatem AJ. High resolution global gridded data for use in population studies. *Sci Data* 2017; **4**: 1–17.

17 Gilbert M, Nicolas G, Cinardi G, *et al.* Global distribution data for cattle, buffaloes, horses, sheep, goats, pigs, chickens and ducks in 2010. *Sci Data* 2018; **5**: 180227.

18 Pigott DM, Deshpande A, Letourneau I, *et al.* Local, national, and regional viral haemorrhagic fever pandemic potential in Africa: a multistage analysis. *The Lancet* 2017; **390**: 2662–72.

19 Brady OJ, Golding N, Pigott DM, *et al.* Global temperature constraints on Aedes aegypti and Ae. albopictus persistence and competence for dengue virus transmission. *Parasit Vectors* 2014; **7**: 338.

20 Soti V, Chevalier V, Maura J, *et al.* Identifying landscape features associated with Rift Valley fever virus transmission, Ferlo region, Senegal, using very high spatial resolution satellite imagery. *Int J Health Geogr* 2013; **12**: 10.

21 Lancelot R, Béral M, Rakotoharinome VM, *et al.* Drivers of Rift Valley fever epidemics in Madagascar. *Proc Natl Acad Sci U S A* 2017; **114**: 938–43.
